# Genetic Diversity of *Rhanterium eppaposum* Oliv. Populations in Kuwait as Revealed by GBS

**DOI:** 10.3390/plants11111435

**Published:** 2022-05-27

**Authors:** Fadila Al Salameen, Nazima Habibi, Sami Al Amad, Bashayer Al Doaij

**Affiliations:** Environment and Life Science Research Centre, Kuwait Institute for Scientific Research, Safat 13109, Kuwait; fslamian@kisr.edu.kw (F.A.S.); samad@kisr.edu.kw (S.A.A.); bdoaij@kisr.edu.kw (B.A.D.)

**Keywords:** next-generation sequencing, single nucleotide polymorphisms, native plant, population structure, molecular diversity, desert species, genotyping by sequencing

## Abstract

Natural populations of *Rhanterium eppaposum* Oliv. (Arfaj), a perennial forage shrub, have depleted due to unethical human interventions and climate change in Kuwait. Therefore, there is an urgent need to conserve this native plant through the assessment of its genetic diversity and population structure. Genotyping by sequencing (GBS) has recently emerged as a powerful tool for the molecular diversity analysis of higher plants without prior knowledge of their genome. This study represents the first effort in using GBS to discover genome-wide single nucleotide polymorphisms (SNPs) of local *Rhanterium* plants to assess the genetic diversity present in landraces collected from six different locations in Kuwait. The study generated a novel set of 11,231 single nucleotide polymorphisms (SNPs) and indels (insertions and deletions) in 98 genotypes of *Rhanterium*. The analysis of molecular variance (AMOVA) revealed ~1.5% variation residing among the six populations, ~5% among the individuals within the population and 93% variation present within the populations (F_ST_ = 0.029; *p* = 0.0). Bayesian and UPGMA analyses identified two admixed clusters of the tested samples; however, the principal coordinates analysis returned the complete population as a single group. Mantel’s test returned a very weak correlation coefficient of r^2^ = 0.101 (*p* = 0.00) between the geographic and genetic distance. These findings are useful for the native species to formulate conservation strategies for its sustainable management and desert rehabilitation.

## 1. Introduction

Land degradation is an issue of global concern and poses a serious threat to flora biodiversity, soil fertility and food security in arid lands [1]. Anthropogenic activities, climate change and human population expansion have fueled it further and increased the vulnerability of desert ecosystems [2]. Global simulations predict that current landscapes will store 24% and 10% less carbon in plants and soil, respectively, if covered by their natural vegetation [3]. A substantial amount of carbon is accumulated by native vegetation, thus reducing the impacts of climate change. Indigenous flora is not only important to a region’s cultural identity but is also critical for environmental health concerning soil fertility, agricultural productivity and sustenance of biodiversity [4]. Besides this, it provides a unique niche for the dying flora and fauna [4]. Therefore, plants of genetically diverse wild species must be reintroduced on a large scale to achieve global terrestrial restoration targets [5].

Kuwait harbors an important genetic resource that contains a valuable gene pool of drought and salt tolerance. Nonetheless, Kuwaiti foliage has been severely degraded, and significant changes in vegetation communities have been observed since 1974 [6]. Further, the Gulf War of 1991 brought in enormous levels of oil pollution, badly affecting plantations in the entire country [7]. The natural plant genetic resources of Kuwait are at risk of extinction, so they must be conserved. With the signing of the Convention on Biological Diversity (CBD) in 1993, the nation developed a national biodiversity strategy [8]. This convention was intended to counteract the extinction of species and the loss of biodiversity. Not only does this refer to propagating dying species, but also to ensuring the survival of the maximum number of native species and maintaining their genetic diversity.

*Rhanterium epapposum*, Oliv. (Asteraceae), locally known as “Arfaj”, is the national plant of Kuwait [9] and grows in deep, sandy soils and shallow substrates. The plant reproduces sexually and exhibits cross-pollination. The seeds are blown short distances by winds or transported over long distances by zoochory [10]. The diploid set of chromosome numbers of this species was identified as twelve [11]. The plant is also valued for its essential oils that have been used to treat skin ailments and gastrointestinal disorders in folklore [12]. Camels and sheep graze on it as forage. The plant’s aesthetic appeal, flower color and potential to adapt to urban landscaping makes it an ideal candidate for desert rehabilitation [6]. Human interventions such as off-road driving, camping and expansion in urban areas [13] have affected the natural population of *Rhanterium*, thereby reducing it from 30.6% to 2.1% [14,15]. Previously common in southern Kuwait, the species now occurs only in protected areas such as Sulaibiya Field Station, military airbases, military camps and some restricted oil fields [16,17]. It is therefore necessary to understand the extent and distribution of the genetic variation within these populations, to devise sampling strategies that are efficient in capturing the genetic diversity for selection trials and to use the material in revegetation and rehabilitation projects that are intended for maintaining the biological diversity of this native plant in Kuwait.

Technological advancement has brought about a paradigm shift in the way of assessing genetic diversity. The conventional morphological markers have been replaced by the use of first-generation molecular markers such as simple sequence repeats (SSRs), amplified fragment length polymorphism (AFLP) and randomly amplified polymorphic DNA (RAPDs) [18,19,20]. Due to a further reduction in the costs of next-generation technologies, the second generation of molecular markers based on single nucleotide polymorphisms (SNPs) have now become more common [21]. Various experimental methods can be used to observe SNP markers in plants, but genotyping by sequencing is currently the most popular way to study SNPs in plants [22,23]. The GBS approach does not require prior knowledge of the genome, as it is based on the principle of genome reduction with restriction enzymes. It combines the process of marker discovery and genotyping, generating a large dataset of genome-wide SNPs, making it an ideal choice for plant genetic diversity analysis [23,24].

There are very few published studies on the genetic diversity and population structure of *Rhanterium eppaposum* in Kuwait, the Gulf Cooperation Council (GCC) and other tropical countries [9,13,14,15,25]. Our study aimed to identify genome-wide and high-quality SNPs in *R. eppaposum* populations to estimate genetic diversity and to identify genetic structures within the wild landraces growing in Kuwait. The findings provide a basis to formulate strategies for its conservation and rehabilitation.

## 2. Results

To the best of our knowledge, in this study, SNP markers were generated and used for the first time in the non-model species of *R. eppaposum* through the technique of GBS.

### 2.1. Genotyping and SNP Density

**Figure 1:**
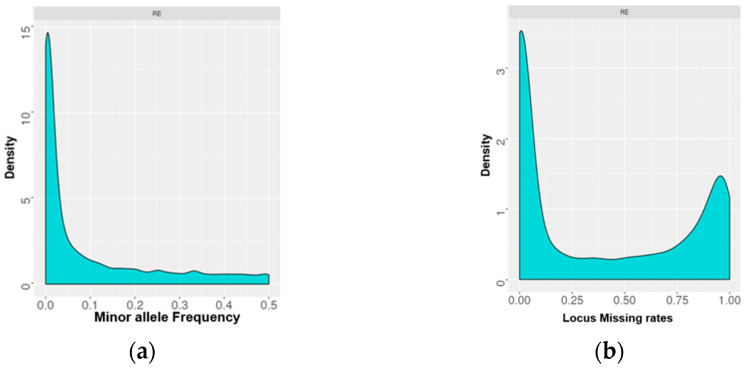
(**a**) Minor allele frequency and (**b**) locus missing rates in the genomes of *Rhanterium eppoposum* genotypes of Kuwait.

For the 99 samples, sequencing of the GBS library yielded 40 million raw reads with an average base quality of Q 30 (Figure S1). The sequencing coverage was estimated to be 10x. Combined variant calling files (.vcf) were generated, and a total of 38,199 genomic variants (34,109 SNPs and 2207 indels) were obtained. A high density of low-frequency variants was recorded in the samples tested in the present study (Figure 1a). The genomic missing rates were also recorded to be high (Figure 1b), and therefore, the locus missing rate of 0.6 was set as a cut-off to filter out low-quality variants. Samples with >5% missing genotypes, variants with missing calls in more than 5% of samples and variants with minimum allele frequency (MAF) < 0.01 were filtered. This removed 15,781 (41%) low-quality SNPs/indels and a single genotype. The total number of variants remained at 11,231 (10,568 SNPs and 753 indels). The single genotype was also excluded from further analysis.

**Figure 2:**
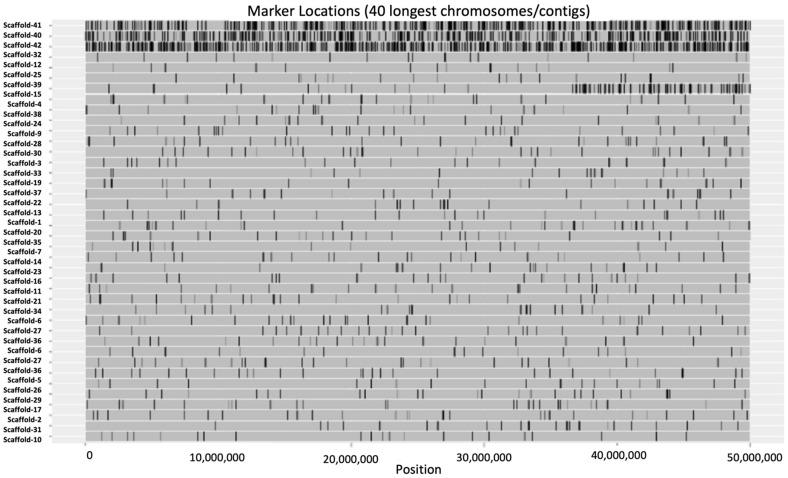
Variant density plot of *Rhanterium eppaposum* genotypes. Each row is represented by a scaffold of 50 Gb length. The black lines denote the SNPs and indels. The top three scaffolds were highest in variant density.

These variants were distributed on 44 scaffolds of 50 GB each (Figure 2). Maximum density was observed on Scaffold 42, followed by scaffolds 41 and 42. The variant density ranged from 66 (0.00132/Mbps) to 2527 (0.05134/Mbps) variants (Tables S1 and S2).

### 2.2. Molecular Diversity

A cut-off of 0.05 for the allowed level of variants for molecular diversity analysis further filtered the usable number of SNPs at each location. Maximum numbers were 8841 at Al Kabd, followed by 8838 at Al Salmi, 8583 at Om Qaser, 8547 at Al Maqwa, 8078 at SANR and 7536 at Mina Abdulla. Among these, approximately 26% were polymorphic at Al Salmi and Al Kabd, 21 % at SANR and Mina Abdulla, 16.5% at Om Qaser and 14% at Al Maqwa (Table 1).

There were more transition-type SNPs (28.40%) than transversion-type (19.5%), and the ratio of Ts/Tv was 1.5 (2351/1618). Substitutions were higher than the transitions and transversions. The observed theta (pi) was higher than the expected theta (S) at all the locations yielding a negative Tajima’s D (D < 0). From this, we interpret that there is an abundance of rare alleles in all the *Rhanterium* populations. In addition to this, the inbreeding coefficient (FS) ranged from 0.129 to 2.83 (mean −1.095 *p* = 0.00).

### 2.3. Genetic Differentiation

An AMOVA partitioning on this dataset revealed 93% of variance distributed within the populations, 4.8% among the individuals among the populations and a minimum of ~1.5% among the populations. An average F_ST_ of 0.02, F_IT_ of 0.06 and a F_IS_ of 0.05 at a confidence interval of 95% was obtained. The values of fixation indices were in agreement with molecular diversity indices (Table 2).

The genetic distance (F_ST_) was at a minimum (0.004) between Al Salmi and Al Kabd and a maximum (0.04) between Om Qaser and Mina Abdulla. Almost comparable F_ST_ of 0.032 and 0.033 were observed between SANR and Mina Abdulla and between Al Maqwa and Mina Abdulla (Table 3). The Mina Abdulla location exhibited an F_ST_ ~0.03 with Al Kabd and Al Salmi. Our results suggested Mina Abdulla is the farthest among all the locations.

### 2.4. Population Structure

**Figure 3:**
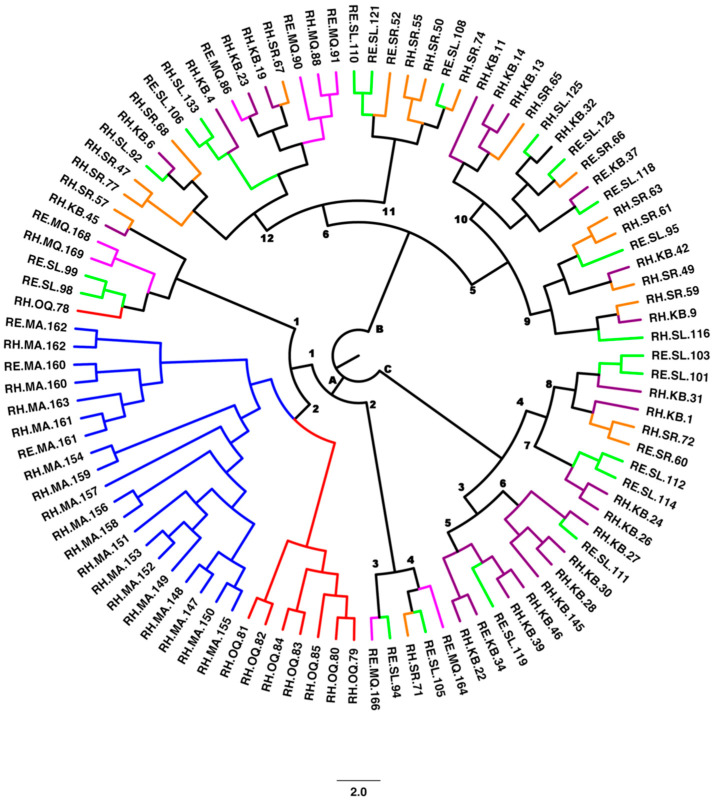
Cladogram showing population structure of 98 *Rhanterium* accessions sampled across six locations in Kuwait.

To establish phylogenetic relationships among the 98 genotypes, a cladogram was constructed using the UPGMA method and 10,568 SNPs (Figure 3). Cluster A with four sub-clusters, cluster B with five sub-clusters and cluster C with six sub-groups each were seen. All the clusters had overlapping populations from the six locations. Only Mina Abdulla populations existed as one combined group in cluster A.

**Figure 4:**
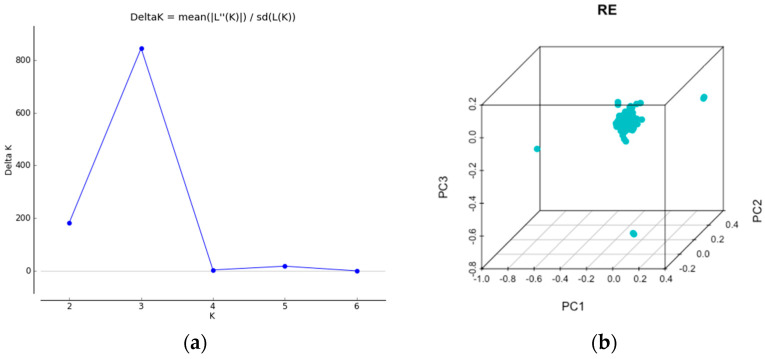

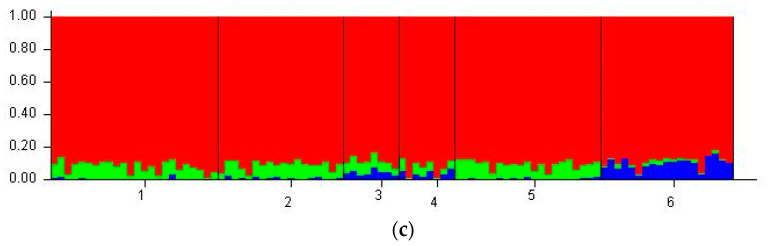
(**a**) Relationship between delta K and K obtained through Structure Harvester, (**b**) principal component analysis on 98 accessions of *Rhanterium,* (**c**) Bayesian model clustering, numbers 1–6 represent the six populations, and the colors depict three clusters.

The Bayesian approach of clustering by Evanno’s method demonstrated a clear peak at 3 (Figure 4a), indicating that three groups were distributed across all the *Rhanterium* populations. The PCA analysis plotted all the *Rhanterium* of Kuwait as a single collection (Figure 4b). The first three principal components explained 5.1% (PC1 = 1.8, PC2 = 1.7 and PC3 = 1.6) of the variance for *R. eppaposum*. A complete admixture of populations was observed in the STRUCTURE plot (Figure 4c).

**Figure 5:**
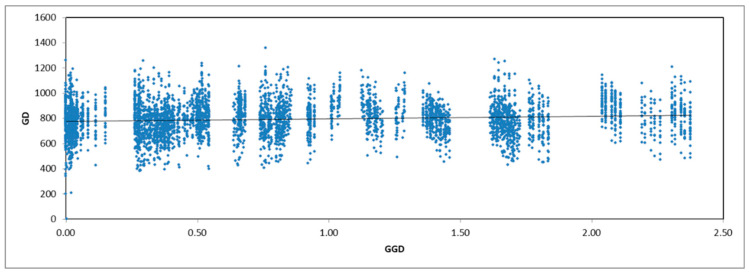
Mantel’s test graph of the genetic (GD) and geographic distances (GGD) among 98 landraces of *Rhanterium eppaposum*.

The clustering analysis was further supported by Mantel’s test. An r^2^ of 0.101 at *p* = 0.05 was obtained between the genetic and geographic distance, indicating that the populations were sparingly isolated by distance (Figure 5).

## 3. Discussion

This study reports the genome-wide characterization of *Rhanterium* for the first time in Kuwait. We were able to generate 10,568 SNPs from 98 genotypes and studied their genetic diversity. The model-based Bayesian clustering revealed the occurrence of three admixed clusters with only 1.5% variation residing among the populations. The high intra-population diversity is encouraging in terms of the presence of rare alleles rendering scope for further population expansion. The technique established will also help in studying the genetic diversity of other non-model species in Kuwait and abroad.

### 3.1. Genotyping by Sequencing

When a reference genome is lacking, de novo assembly of a rough genome at a lower coverage is desirable. In the present study, rough genomes based on the whole genome sequencing for one representative sample were used as a reference genome for subsequent alignments. Previous reports on the use of de novo contigs as a reference for SNP discovery and genotyping on non-model species are available [23,24,26]. Genome survey sequencing has been employed previously for a native tree of Kuwait to filter microsatellite motifs [19]. However, transcriptomes have also been used as a reference for genotyping non-model species avoiding the need for de novo DNA assembly with the advantage of finding SNP sites near selected targets [27]. Some other non-reference-based methods for GBS analysis include Stacks [28], TASSEL [29] and UNEAK [30]. Haplotag Software for Haplotype-Based GBS analysis is also optimized for use in an out-crossing and self-pollinating non-model plant species [24,31]. The in silico prediction of optimum restriction enzymes (PstI + BtgI) was also performed from the reference genome, setting a baseline to obtain at least 1000 filtered markers. To increase the genome coverage and reduce the levels of missing data, we employed two restriction enzymes to fragment genomic DNA. Some of the previous studies have demonstrated that double-enzyme digestion generates more consistent results among different individuals than single-enzyme digestion [24,32,33].

In the current study, haplotype-based FreeBayes was employed to call putatively polymorphic variants (specifically SNPs, indels, MNPs and composite insertion and substitution events) from the short-read aligned BAM files (Phred + 33) into a variant call format [34]. Using the double-digest approach and haplotype-based variant calling, we were able to order 11,231 good quality markers against the predicted 1000 markers deemed to be sufficient for downstream genetic diversity analysis. Freebayes [34] was applied to the 1000 Genomes Exon Targeted samples, using default parameters [35]. GBS has been attempted previously to study the genetic diversity of crested wheatgrass [36], forage grasses [37] and Rimth, native to Kuwait [15,22].

### 3.2. Molecular Diversity

In this study, the number of polymorphic SNPs in Al Kabd populations was the highest. This confirmed our previous predictions based on RAPD, SRAP and ISSR markers [9,13,14]. Higher diversity in these populations may provide new elite and desirable alleles facilitating genotypes for desert rehabilitation and revegetation [38]. Although a high number of variants were generated, only 59% of them fell above the cut-off of 0.6 (locus missing rates). This could be attributed to the naturally high levels of sequence diversity in wild accessions [29]. *Haloxylon salicornicum* growing in similar environments also depicted higher locus missing rates [15].

The average transition/transversion (Ts/Tv) ratio was found to be 1.5. The mutation of methylcytosine to uracil and eventually to thymine might result in transition abundance within a species [39]. In Eukaryotes, cytosine methylation in DNA is one of the most significant changes and is crucial for gene activity regulation and genomic integrity preservation. Plant development, differentiation and response to biotic and abiotic stresses are all influenced by DNA methylation and other epigenetic mechanisms [40]. *Rhanterium* populations naturally grow under stressed conditions; consequently, methylation events are expected to be high. The voluminous transitions and exceedingly repetitive sequences can be considered an evolutionary footprint of methylation events [40,41,42]. Analogous observations were recorded in Iranian wheat landraces [43] and wild accessions of *H. salicornicum* populations [15]. A highly repetitive genome was also recorded in *Acacia pachyceras* growing in Kuwait [19].

The negative Tajima’s D values are encouraging and suggestive of excessive low-frequency polymorphisms or rare alleles [44]. This could be attributed to a selective sweep event that might have resulted in the population expansion of the *Rhanterium* population in Kuwait. This was supported by the inbreeding coefficients, which indicate a higher number of observed heterozygotes within the populations [45].

### 3.3. Mixed Ancestry Is a Potential Cause for Low Genetic Diversity among Populations

The findings from a population structure analysis suggest that the large, genetically homogenous group of 98 genotypes of *Rhanterium* collected all over Kuwait is likely to originate from a common ancestor. The minor genetic differences are believed to be due to recombination events occurring during sexual reproduction [46]. These findings show that the majority of *Rhanterium* landraces are genetically similar, justifying the need to extend the diversity of future rehabilitation projects by integrating alternative genotypes from neighboring countries such as Saudi Arabia, UAE, Oman, Qatar, etc. In a similar study conducted with ISSR markers [9] and SRAP and RAPD markers [13], low genetic diversity among the populations was demonstrated. Likewise, the populations of *Haloxylon* also demonstrated a weak population structure in Kuwait [45]. Our analysis showed a high within-line genetic variation of tested *Rhanterium* populations, which agrees with studies on highly outcrossing species [47]. Overall, our genetic diversity results are following diversity studies of *Rhanterium* [9,13].

### 3.4. Geographic Origin Showed No Congruence with Genetic Structure

There was no clear congruence between genetic structure (clusters) and geographic origin in our analyses, i.e., no grouping was based on spatial origin. This was confirmed through the results of Mantel’s correlation test. No isolation by distance pattern was observed. Regular exchange of pollens, critters and reproductive material taking place between these spatially apart but outcrossing species is the only explanation that can be given for this in the present circumstances. Kuwait is a country with a small land area of 17,000 km^2^. The topography is flat with slightly uneven desert dunes. Frequent sandstorms and winds (*Toz*) are quite common. These features facilitate the easy dissemination of seeds to otherwise far areas [48]. The complete admixture of populations is thus justified, as stated by Sewell Wright [48]. These results were in congruence with other desert species of Kuwait [45].

We speculated differences in the population structure owing to the various soil types in which these populations are growing. The soil types of OmQaser, SANR, KABD, Al Maqwa, Mina Abdulla and Al Salmi were calcigypsids, haplocalcids, torripsaments, acquisalids and Petrogypsids, respectively [49]. It appears from our findings that the soil types are not influencing the *Rhanterium* community much in Kuwait. The phenotypic characterization of these populations, however, needs to be undertaken.

## 4. Materials and Methods

### 4.1. Plant Material and DNA Isolation

**Figure 6:**
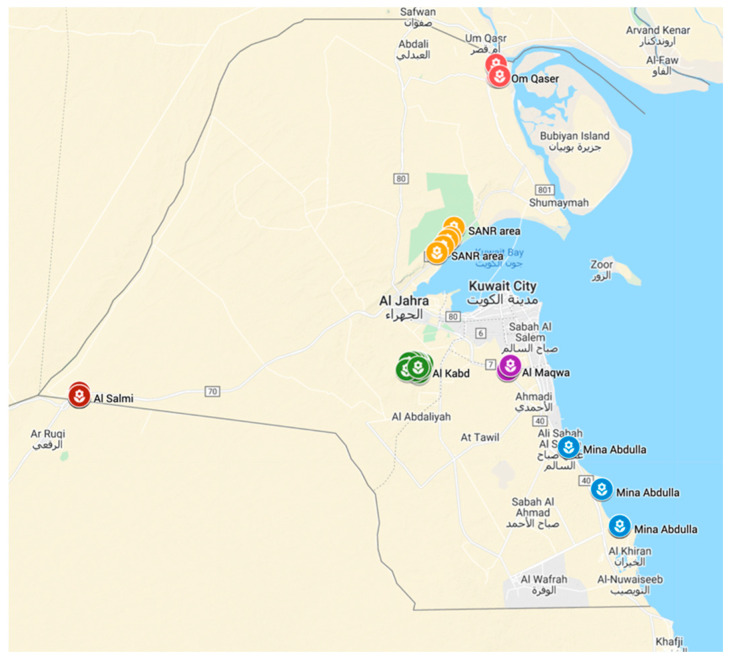
Map of Kuwait showing the distribution of six populations of *Rhanterium eppaposum.* Each population is color-coded.

A set of 99 Kuwaiti *Rhanterium* landraces were used in this study. They were collected from the areas of Al Kabd (n = 21), Sabah Al Ahmed Nature Reserve (SANR, n = 18), Om Qaser (n = 8), Al Maqwa (n = 8), Al Salmi (n = 24) and Mina Abdulla (n = 19). The GPS coordinates of each specimen were recorded and used to mark the locations on Google Maps (Figure 6). The GPS coordinates and soil types of these populations are provided in Table S3. The average annual precipitation ranges between 75 and 160 mm/yr. The temperature in Kuwait falls between 0 and 46 °C. Genomic DNA was extracted using a GenElute^TM^ Plant Genomic DNA Miniprep Kit (Sigma, St. Louis, MO, USA), as per the manufacturer’s instructions, from young leaves [15]. All the DNA samples were quantified through a Qubit fluorometer (Thermo Fisher Scientific, Carlsbad, CA, USA) employing the Qubit ^®^BR dsDNA Assay (Life Technologies, Inc., Grand Island, NY, USA).

### 4.2. GBS and SNP Calling

The GBS was performed at the University of Minnesota Genomic Centre (UMGC), USA. Genomic DNA was digested using the restriction enzymes PstI + BtgI (New England BioLabs, Inc., Ipswich, MA, USA), and barcoded adapters were ligated to each DNA sample using T4 ligase (New England BioLabs, Inc.). Dual indexed SBG libraries for R. eppaposum were pooled and loaded across 4 lanes of a 150 bp single read sequencing run on a NextSeq 550 (Illumina, San Deigo, CA, USA) instrument with a coverage of 10x generating ~40 M raw reads.

In-house-developed Gopher-pipelines by the University of Minnesota Informatics Institute in collaboration with the University of Minnesota Genomics Center and the Research Informatics Solutions (RIS) group at the University of Minnesota Supercomputing Institute were used for sequence alignment and variant calling (Supplementary File). Per-base and per-read quality score statistics were calculated for each fastq file through FastQC [50]. Mean quality scores for all libraries were ≥Q30. Sequence adapters were removed, and low-quality bases were trimmed using Trimmomatic [51]. Each sample was aligned to a reference genome using BWA, and the bam file was sorted and indexed. A representative sample (RH_KB_01) was chosen for paired-end, whole-genome sequencing at a coverage of 50x on an Illumina HiSeq 2500 [52]. Approximately 180–190 M raw reads with a GC content of 35% were assembled with Abyss 2.0.2, applying the “abyss-pe” command using a kmer size of 64 [53]. This was used as a reference to align the 99 GBS libraries. The Bayesian genetic variant detector FreeBayes v1.1.0 was used for joint SNP calling on all the samples [34]. Mantel’s test was run on geographic and genetic distances using GenAlEx6.5 software [54].

### 4.3. Data Analysis

The analysis of molecular variance (AMOVA) was used to partition the genetic variation into inter- and intra-gene pool diversities using Arlequin V3.5 software [55]. For the analysis of population structure, a model-based Bayesian cluster analysis was performed using STRUCTURE version 2.3.4 [56]. The structure analysis was run 10 times for each K value (K = 1 to 7) using a burn-in period of 1000 steps and 10,000 MC steps and an admixture model. All parameters were set to default values recommended by the manufacturer. The probability of best fit into each number of assumed clusters (K) was estimated by an ad hoc statistic ΔK based on the rate of change in the log probability of data between consecutive K values [57]. A cladogram based on the similarity between the SNPs was constructed employing the UPGMA algorithm [13].

## 5. Conclusions

With the application of GBS, it has been possible to generate 10,568 SNP markers for diversity analysis of *Rhanterium* in Kuwait. The variation residing among these populations was found to be 1.5%. Further analysis grouped the assayed samples into three genetic clusters with admixed populations. These results can enhance genotype selection for increased genetic variation and for desert rehabilitation. The findings in this study can also aid in the application of GBS in the characterization of non-model plants with complex genomes.

## Figures and Tables

**Table 1:** A summary of SNPs, indels, transitions and transversions in 98 genotypes of *R. eppaposum.*

	Al Kabd	SANR	Om Qaser	Al MAqwa	Al Salmi	Mina Abdulla	Mean
**No. of Loci**	10,568	10,568	10,568	10,568	10,568	10,568	10,568
**No. of usable loci**	8841	8078	8583	8547	8838	7536	8279
**Polymorphic loci**	2278	1730	1422	1188	2283	1587	3936
**# Transitions**	1362	1063	872	725	1368	970	2351
**# Transversions**	927	680	552	467	927	622	1618
**# Substitutions**	2289	1743	1424	1192	2295	1592	3969
**# Ts. sites**	1358	1061	872	723	1363	969	2345
**# Tv. sites**	927	680	552	467	927	622	1618
**No. of substitution sites**	2278	1730	1422	1188	2283	1587	3936
**Ts/Tv**	1.46	1.56	1.57	1.47	1.47	1.55	1.45
**Theta (S)**	610.022 ± 196.21	502.972 ± 173.39	548.429 ± 235.35	458.18 ± 196.68	634.56 ± 210.62	454.06 ± 154.50	763.223 ± 184.03
**Theta (pi)**	430.148 ± 212.30	396.288 ± 198.73	490.42 ± 267.94	398.57 ± 217.83	463.02 ± 230.03	374.97 ± 187.43	192.107 ± 192.10
**Tajima’s D (p)**	−1.196 (0.09)	−0.908 (0.19)	−0.581 (0.31)	−0.715 (0.26)	−1.123 (0.12)	−0.737 (0.24)	−1.616 (0.02)
**FS (p)**	0.129 (0.35)	0.769 (0.42)	2.83 (0.57)	2.62 (0.56)	0.573 (0.36)	0.568 (0.39)	1.095 (0.00)

**Table 2:** Variations within, between and among the populations of *Rhanterium eppaposum* as observed by analysis of molecular variance (AMOVA).

Source of Variation	d.f.	Sum of Squares	Variance Components	Variation (%)
Among populations	5	4901.21	9.65 Va	1.45
Among individuals within populations	87	59,994.72	31.97 Vb	4.79
Within individuals	93	58,184.50	625.63 Vc	93.76
Total	185	123,080.43	667.27	

**Fixation Index F_ST_= 0.014 (*p* ≥ 0.05); F_IT_of 0.06 (*p* ≤ 0.05); F_IS_ of 0.05(*p* ≤ 0.05)**. Significance test at 10,100 permutations.

**Table 3:** Pairwise genetic distances (F_ST_) among six populations of *Rhanterium* in Kuwait.

	Al Kabd	SANR	Om Qaser	Al Maqwa	Al Salmi	Mina Abdulla
**Al Kabd**	0.00000					
**SANR**	0.00569	0.00000				
**Om Qaser**	0.01722	0.02398	0.00000			
**Al Maqwa**	0.00544	0.00965	0.02474	0.00000		
**Al Salmi**	**0.00401**	0.00614	0.02227	0.01068	0.00000	
**Mina Abdulla**	0.02726	0.03259	**0.03940**	0.03320	0.02969	0.00000

## Data Availability

All data generated or analyzed during this study are included in this published article. The raw DNA sequencing data are available on the NCBI portal under the accession number PRJNA669035 (SAMN16430163 to SAMN16430261).

## Supplementary Materials

The following supporting information can be downloaded at: https://www.mdpi.com/article/10.3390/plants11111435/s1. Figure S1: Phred Quality of 99 samples of *Rhanterium eppaposum* sequenced on an Illumina platform; Table S1: SNP/Indel counts per scaffolds; Table S2: Position of SNPs and Indels on each scaffold and Table S3: GPS coordinates and soil types of *Rhanterium* populations growing in Kuwait.

## References

[1] Stringer L. (2008). Reviewing the International Year of Deserts and Desertification 2006: What contribution towards combating global desertification and implementing the United Nations Convention to Combat Desertification?. J. Arid Environ..

[2] Yirdaw E., Tigabu M., Monge Monge A.A. (2017). Rehabilitation of degraded dryland ecosystems–review. Silva Fenn..

[3] Bondeau A., Smith P.C., Zaehle S., Schaphoff S., Lucht W., Cramer W., Gerten D., LOTZE-CAMPEN H., Müller C., Reichstein M. (2007). Modelling the role of agriculture for the 20th century global terrestrial carbon balance. Glob. Change Biol..

[4] Morton S., Cullen P., Bourne G., Cristofani P., Possingham H., Young M.D. (2002). Sustaining Our Natural Systems and Biodiversity. https://publications.csiro.au/rpr/pub?list=BRO&pid=procite:78417c7a-70a5-4ffd-ae4d-6b695b8c3a16.

[5] Nevill P.G., Tomlinson S., Elliott C.P., Espeland E.K., Dixon K.W., Merritt D.J. (2016). Seed production areas for the global restoration challenge. Ecol. Evol..

[6] Omar S.A., Bhat N. (2008). Alteration of the Rhanterium epapposum plant community in Kuwait and restoration measures. Int. J. Environ. Stud..

[7] Böer B., Sargeant D. (1998). Desert perennials as plant and soil indicators in Eastern Arabia. Plant Soil.

[8] CBD (1992). Convention on biological diversity. U. N. Treaty Ser..

[9] Al Salameen F., Habibi N., Al Amad S., Kumar V., Dashti J., Talebi L., Al Doaij B. (2020). Genetic diversity analysis of *Rhanterium eppaposum* Oliv. by ISSRs reveals a weak population structure. Curr. Plant Biol..

[10] Brown G.M. (2001). Vegetation Ecology and Biodiversity of Degraded Desert Areas in North-Eastern Arabia. Habilitation Thesis.

[11] Malallah G., Al-Dosari M., Murin A. (2001). Determination of chromosome numbers in Kuwaiti flora II. THAISZIA-KOSICE-.

[12] Demirci B., Yusufoglu H.S., Tabanca N., Temel H.E., Bernier U.R., Agramonte N.M., Alqasoumi S.I., Al-Rehaily A.J., Başer K.H.C., Demirci F. (2017). Rhanterium epapposum Oliv. essential oil: Chemical composition and antimicrobial, insect-repellent and anticholinesterase activities. Saudi Pharm. J..

[13] Al-Salameen F., Al-Amad S., Al-Hashash H. (2014). Determination of genetic variation of Rhanterium epapposum in Kuwait desert using RAPD and SRAP DNA-based markers. Kuwait J. Sci..

[14] Habibi N., Salameen A. Role of ISSR markers for conservation of *Rhanterium eppaposum* Oliv. Proceedings of the International Symposium and Workshop on Native Seeds in Restoration of Dryland Ecosystems.

[15] Fadila A.S., Nazima H., Vinod K., Sami A.A., Leena T., Bashayer A.D., Jamal D. (2018). Genetic Characterization of Haloxylon Salicornicum and Rhanterium Eppaposum Native Plant Species of Kuwait by DNA Markers.

[16] Omar S.A., Misak R., King P., Shahid S.A., Abo-Rizq H., Grealish G., Roy W. (2001). Mapping the vegetation of Kuwait through reconnaissance soil survey. J. Arid Environ..

[17] Zaman S., Padmesh S., Tawfiq H. (2010). Germination ecology of Rhanterium epapposum olive. Am. J. Appl. Sci..

[18] Habibi N., Rahman M.H., Al Salameen F. (2020). Synoptic Overview on Application of Molecular Genetic Markers in Acacia. Res. J. Biotechnol..

[19] Habibi N., Al Salameen F., Rahman M., Kumar V., Al Amad S., Shajan A., Zakir F., Razzack N.A., Tinwala W.H. (2022). Draft Genome Sequence and SSR mining data of Acacia pachyceras Schwartz. Data Brief.

[20] Mathur P., Habibi N., Chittora M., Purohit S. (2013). Molecular Analysis of Variability among Genotypes of *Abrus precatorius* L. with Different Seed Coat Colours Using RAPD and ISSR Markers. Ind. J. Biotechnol..

[21] Davey J.W., Hohenlohe P.A., Etter P.D., Boone J.Q., Catchen J.M., Blaxter M.L. (2011). Genome-wide genetic marker discovery and genotyping using next-generation sequencing. Nat. Rev. Genet..

[22] Habibi N. DNA Marker Technology for Conservation of Plant Genetic Resources in Kuwait. Proceedings of the 13th International Conference on Development of Drylands Converting Dryland Areas from Grey into Green.

[23] Elshire R.J., Glaubitz J.C., Sun Q., Poland J.A., Kawamoto K., Buckler E.S., Mitchell S.E. (2011). A robust, simple genotyping-by-sequencing (GBS) approach for high diversity species. PLoS ONE.

[24] Peterson G.W., Dong Y., Horbach C., Fu Y.-B. (2014). Genotyping-by-sequencing for plant genetic diversity analysis: A lab guide for SNP genotyping. Diversity.

[25] Abdullah M.T. (2017). Conserving the Biodiversity of Kuwait through DNA Barcoding the Flora.

[26] Deschamps S., Llaca V., May G.D. (2012). Genotyping-by-sequencing in plants. Biology.

[27] Salazar J.A., Pacheco I., Shinya P., Zapata P., Silva C., Aradhya M., Velasco D., Ruiz D., Martínez-Gómez P., Infante R. (2017). Genotyping by sequencing for SNP-based linkage analysis and identification of QTLs linked to fruit quality traits in Japanese plum (*Prunus salicina* Lindl.). Front. Plant Sci..

[28] Catchen J., Hohenlohe P.A., Bassham S., Amores A., Cresko W.A. (2013). Stacks: An analysis tool set for population genomics. Mol. Ecol..

[29] Glaubitz J.C., Casstevens T.M., Lu F., Harriman J., Elshire R.J., Sun Q., Buckler E.S. (2014). TASSEL-GBS: A high capacity genotyping by sequencing analysis pipeline. PLoS ONE.

[30] Lu F., Lipka A.E., Glaubitz J., Elshire R., Cherney J.H., Casler M.D., Buckler E.S., Costich D.E. (2013). Switchgrass genomic diversity, ploidy, and evolution: Novel insights from a network-based SNP discovery protocol. PLoS Genet..

[31] Tinker N.A., Bekele W.A., Hattori J. (2016). Haplotag: Software for haplotype-based genotyping-by-sequencing analysis. G3 Genes Genomes Genet..

[32] Poland J.A., Brown P.J., Sorrells M.E., Jannink J.-L. (2012). Development of high-density genetic maps for barley and wheat using a novel two-enzyme genotyping-by-sequencing approach. PLoS ONE.

[33] Shirasawa K., Ban T., Nagata N., Murakana T. (2019). Impact of genomics on Capsicum breeding. The Capsicum Genome.

[34] Garrison E., Marth G. (2012). Haplotype-based variant detection from short-read sequencing. arXiv.

[35] Warden C.D., Adamson A.W., Neuhausen S.L., Wu X. (2014). Detailed comparison of two popular variant calling packages for exome and targeted exon studies. PeerJ.

[36] Baral K., Coulman B., Biligetu B., Fu Y.-B. (2018). Genotyping-by-sequencing enhances genetic diversity analysis of crested wheatgrass [*Agropyron cristatum* (L.) Gaertn.]. Int. J. Mol. Sci..

[37] Oliveira F.A.d. (2017). Estudos Genéticos-Genômicos em Gramíneas Forrageiras Tropicais dos Gêneros Urochloa e Paspalum: Genetic-Genomic Studies in Tropical Forage Grasses of the Genus Urochloa and Paspalum.

[38] Trethowan R.M., Mujeeb-Kazi A. (2008). Novel germplasm resources for improving environmental stress tolerance of hexaploid wheat. Crop Sci..

[39] Coulondre C., Miller J.H., Farabaugh P.J., Gilbert W. (1978). Molecular basis of base substitution hotspots in Escherichia coli. Nature.

[40] Kalinka A., Achrem M., Poter P. (2017). The DNA methylation level against the background of the genome size and t-heterochromatin content in some species of the genus *Secale* L.. PeerJ.

[41] Buckler E., Holtsford T.P. (1996). Zea ribosomal repeat evolution and substitution patterns. Mol. Biol. Evol..

[42] Feldman M., Levy A.A. (2012). Genome evolution due to allopolyploidization in wheat. Genetics.

[43] Alipour H., Bihamta M.R., Mohammadi V., Peyghambari S.A., Bai G., Zhang G. (2017). Genotyping-by-sequencing (GBS) revealed molecular genetic diversity of Iranian wheat landraces and cultivars. Front. Plant Sci..

[44] Schmidt D., Pool J. (2002). The effect of population history on the distribution of the Tajima’s D statistic. Popul. Engl. Ed..

[45] Al Salameen F., Habibi N., Kumar V., Al Amad S., Dashti J., Talebi L., Al Doaij B. (2018). Genetic diversity and population structure of Haloxylon salicornicum moq. in Kuwait by ISSR markers. PLoS ONE.

[46] Mezard C. (2006). Meiotic recombination hotspots in plants. Biochem. Soc. Trans..

[47] Hamrick J. (1989). Isozymes and the analysis of genetic structure in plant populations. Isozymes in Plant Biology.

[48] Wright S. (1917). The average correlation within subgroups of a population. J. Wash. Acad. Sci..

[49] Omar S.A., Shahid S.A. (2013). Reconnaissance Soil survey for the state of Kuwait. Developments in Soil Classification, Land Use Planning and Policy Implications.

[50] Andrews S. (2010). FastQC: A quality control tool for high throughput sequence data. Available online. Retrieved May.

[51] Bolger A.M., Lohse M., Usadel B. (2014). Trimmomatic: A flexible trimmer for Illumina sequence data. Bioinformatics.

[52] Al Salameen F., Habibi N., Al Amad S., Al Doaij B. (2021). Data on draft genome assembly and annotation of Haloxylon salicornicum Moq. Data Brief.

[53] Jackman S.D., Vandervalk B.P., Mohamadi H., Chu J., Yeo S., Hammond S.A., Jahesh G., Khan H., Coombe L., Warren R.L. (2017). ABySS 2.0: Resource-efficient assembly of large genomes using a Bloom filter. Genome Res..

[54] Peakall R., Smouse P.E. (2006). GENALEX 6: Genetic analysis in Excel. Population genetic software for teaching and research. Mol. Ecol. Notes.

[55] Excoffier L., Lischer H.E. (2010). Arlequin suite ver 3.5: A new series of programs to perform population genetics analyses under Linux and Windows. Mol. Ecol. Resour..

[56] Pritchard J.K., Stephens M., Donnelly P. (2000). Inference of population structure using multilocus genotype data. Genetics.

[57] Evanno G., Regnaut S., Goudet J. (2005). Detecting the number of clusters of individuals using the software STRUCTURE: A simulation study. Mol. Ecol..

