# Supplementary material for: Genetic Diversity of Rhanterium eppaposum Oliv. Populations in Kuwait as Revealed by GBS
Source: Plants (Basel). 2022 May 27;11(11):1435. doi: 10.3390/plants11111435 (PMC9183190; doi:10.3390/plants11111435)
Supplement: Supplementary file 1 [file plants-11-01435-s001.zip › Supplementary Figure S1.pdf]

Reads per sample Plots

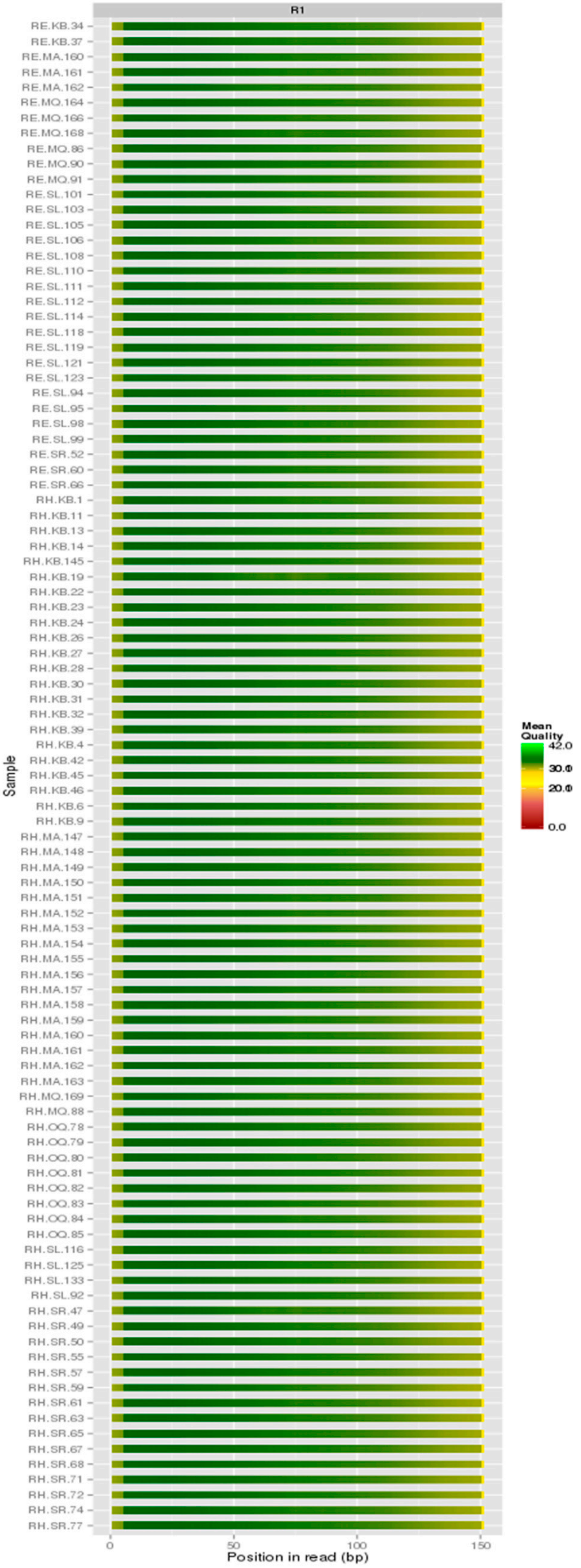

Figure S1: Phred Quality of 99 samples of *Rhanterium eppaposum* sequenced on an Illumina platform
