# Supplementary material for: Genetic Diversity of Rhanterium eppaposum Oliv. Populations in Kuwait as Revealed by GBS
Source: Plants (Basel). 2022 May 27;11(11):1435. doi: 10.3390/plants11111435 (PMC9183190; doi:10.3390/plants11111435)
Supplement: Supplementary file 1 [file plants-11-01435-s001.zip › Supplementary Table S1-S2.pdf]

Table S1: SNP/Indel counts per scaffolds

Scaffold 1- 111
Scaffold 10- 123
Scaffold 11-103
Scaffold 12-68
Scaffold 13-126
Scaffold 14- 119
Scaffold 15- 132
Scaffold 16- 134
Scaffold 17-112
Scaffold 18-79
Scaffold 19- 91
Scaffold 2- 160
Scaffold 20- 87
Scaffold 21- 143
Scaffold 22-118
Scaffold 23- 106
Scaffold 24-116
Scaffold 25- 104
Scaffold 26- 103
Scaffold 27-68
Scaffold 28- 147
Scaffold 29- 149
Scaffold 3- 74
Scaffold 30- 95
Scaffold 31- 144
Scaffold 32- 88
Scaffold 33- 94
Scaffold 34- 127
Scaffold 35- 113
Scaffold 36- 131
Scaffold 37- 114
Scaffold 38- 105
Scaffold 39- 516
Scaffold 4- 98
Scaffold 40-1847
Scaffold 41- 2064
Scaffold 42- 2527
Scaffold 5- 159
Scaffold 6- 110
Scaffold 7- 95
Scaffold 8- 66

Scaffold 9- 166

Table S2: Position of SNPs and Indels on each scaffold

SCAFF	POS	ID	REF	ALT	FILTER
Scaffold-1	2057075			T	A
Scaffold-1	2057085			C	G
Scaffold-1	2057109			A	C
Scaffold-1	2057110			TTGTTACAAGGCAGCAGAAGGCTG	TTGTTACAAGGCAGCAAAATGCTG,TAGTTACAAGGCAGCAGAAGGCTG
Scaffold-1	2057136			G	A
Scaffold-1	2057139			G	T
Scaffold-1	2894025			C	T
Scaffold-1	2894040			A	T
Scaffold-1	2894043			A	C
Scaffold-1	2894046			G	T,A
Scaffold-1	2894051			T	C
Scaffold-1	2894054			A	T
Scaffold-1	2894058			C	T,G
Scaffold-1	2894084			G	A
Scaffold-1	3032266			C	A
Scaffold-1	3032366			G	T
Scaffold-1	3032478			C	A
Scaffold-1	4913106			G	A
Scaffold-1	4913154			A	G
Scaffold-1	4913235			T	C
Scaffold-1	8488948			A	G
Scaffold-1	8489055			G	A
Scaffold-1	8489063			T	C
Scaffold-1	8489068			C	T
Scaffold-1	9415367			G	C
Scaffold-1	9415377			T	C
Scaffold-1	9415384			A	G
Scaffold-1	9415397			C	T
Scaffold-1	9415414			A	G
Scaffold-1	9415424			T	A
Scaffold-1	9415438			T	G
Scaffold-1	9415443			T	A
Scaffold-1	12950941			C	A
Scaffold-1	12950959			C	T
Scaffold-1	12950966			C	T
Scaffold-1	12950993			A	G
Scaffold-1	12951016			C	G

Scaffold-1	12951023	A	C
Scaffold-1	14339308	A	C
Scaffold-1	14339314	A	G
Scaffold-1	14339318	T	G
Scaffold-1	14339328	T	C
Scaffold-1	14339347	A	G
Scaffold-1	14339363	T	C
Scaffold-1	14339373	CA	CG
Scaffold-1	16101043	T	C
Scaffold-1	16598127	ATACT	AT
Scaffold-1	17837399	C	T
Scaffold-1	17837411	G	A
Scaffold-1	18654607	T	A
Scaffold-1	18654642	T	C
Scaffold-1	18654655	C	T
Scaffold-1	18654684	A	G
Scaffold-1	20019706	A	G
Scaffold-1	20019712	CATTAATTAC	CATTAC
Scaffold-1	20019762	T	C
Scaffold-1	20019763	TC	TCCGGC
Scaffold-1	20019806	TCAG	CCAG,CCAT,TTAG
Scaffold-1	20019826	A	G
Scaffold-1	21122840	G	T
Scaffold-1	21122863	C	A
Scaffold-1	23050188	G	A
Scaffold-1	23050208	A	C
Scaffold-1	23050274	C	T
Scaffold-1	26795734	C	T
Scaffold-1	26795736	T	C
Scaffold-1	26795778	G	A
Scaffold-1	26795779	G	A
Scaffold-1	28160112	C	T
Scaffold-1	28160124	T	C
Scaffold-1	28160130	G	A
Scaffold-1	28160160	A	G
Scaffold-1	28160179	A	G
Scaffold-1	28160196	A	G
Scaffold-1	28160199	G	T
Scaffold-1	28601841	G	T
Scaffold-1	28601907	G	T
Scaffold-1	28601909	A	C
Scaffold-1	28601923	A	G
Scaffold-1	29611923	T	C
Scaffold-1	29611947	T	C

Scaffold-1	31104608	A	G
Scaffold-1	31585523	A	G
Scaffold-1	36385477	C	T
Scaffold-1	36385488	C	G
Scaffold-1	36385499	T	A
Scaffold-1	36385504	G	A
Scaffold-1	36385529	C	T
Scaffold-1	36385550	T	C
Scaffold-1	36385572	TAGGAC	TAGGGC,TAAGAC,CAGGAC,CTGGAC,TAGAAC
Scaffold-1	36385598	G	A
Scaffold-1	36385599	ATTA	ATC
Scaffold-1	36385605	C	T
Scaffold-1	43152962	G	C
Scaffold-1	43152977	G	A
Scaffold-1	43153008	C	A
Scaffold-1	43153026	A	G
Scaffold-1	43153027	T	C
Scaffold-1	43153040	G	A
Scaffold-1	44041230	G	T
Scaffold-1	44041248	AAAGG	AAAGT
Scaffold-1	44041253	AAGAGAG	AAGAGAA,AAGGGAA
Scaffold-1	44041265	T	A
Scaffold-1	44041281	G	A
Scaffold-1	44041288	GTTTTTTA	GTTTTTTTTTA
Scaffold-1	44041303	G	A
Scaffold-1	44041308	GGA	AGC
Scaffold-1	47570305	CGATTTCTTGAGAG	CGATTTCTGGAGAG,CG
Scaffold-1	47570336	G	A
Scaffold-1	49980725	CG	CA
Scaffold-10	1220600	A	G
Scaffold-10	1220627	A	G
Scaffold-10	2004441	A	G
Scaffold-10	2004514	A	C
Scaffold-10	3186523	C	A
Scaffold-10	3186567	GCA	GA
Scaffold-10	3635848	C	A
Scaffold-10	3635951	C	A
Scaffold-10	5673438	T	G
Scaffold-10	8456792	C	T
Scaffold-10	8456809	G	T
Scaffold-10	8456828	C	A
Scaffold-10	8456835	T	A
Scaffold-10	8456858	A	T
Scaffold-10	8456864	G	T

Scaffold-10	8456874		G	T
Scaffold-10	8914506		G	A
Scaffold-10	8914511		A	G
Scaffold-10	8914519		C	T
Scaffold-10	8914533		C	T
Scaffold-10	8914541		G	T
Scaffold-10	8914597		G	A
Scaffold-10	8938817		G	A
Scaffold-10	8938862		T	A
Scaffold-10	8938886		G	A
Scaffold-10	8938895		C	A
Scaffold-10	8938901		G	C
Scaffold-10	8938925		A	T
Scaffold-10	8938937		A	G
Scaffold-10	11354752		A	C
Scaffold-10	11354773		G	C
Scaffold-10	11354777		G	T
Scaffold-10	11354796		T	C
Scaffold-10	11354804		G	A
Scaffold-10	11354811		A	G
Scaffold-10	11354818		C	T
Scaffold-10	11354820		G	A
Scaffold-10	11354835		T	C
Scaffold-10	11354845		G	T
Scaffold-10	11354848		TGG	TG
Scaffold-10	11354859		G	A
Scaffold-10	11354868		G	T
Scaffold-10	11354874		C	T
Scaffold-10	20698703		C	T
Scaffold-10	20698709		C	T
Scaffold-10	20698726		C	T
Scaffold-10	20698763		A	G
Scaffold-10	20698771		G	A
Scaffold-10	21494856		A	C
Scaffold-10	21494863		A	G
Scaffold-10	21494867		C	T
Scaffold-10	21494868		C	T
Scaffold-10	21494880		T	G
Scaffold-10	21494881		C	A
Scaffold-10	21494886	TCCA	TCCC,TCTC	
Scaffold-10	21494905		C	T
Scaffold-10	21494907		TTTA	CTTG
Scaffold-10	21494923		C	T
Scaffold-10	21494926	CTGTA		CTGTC,CTGCC,CTCTC,CAGTC,CTGAC,CTGCA

Scaffold-10	21494931		AAAA	GAAA
Scaffold-10	21494941		G	T
Scaffold-10	21494955		G	C
Scaffold-10	22369550		C	T
Scaffold-10	22369638		G	A
Scaffold-10	22864474		A	G
Scaffold-10	22864487		A	G
Scaffold-10	22864512		A	G
Scaffold-10	22864533		C	G
Scaffold-10	24001650		T	A
Scaffold-10	26933017		A	T
Scaffold-10	26933059		C	T
Scaffold-10	26933061		A	G
Scaffold-10	26933081		T	C
Scaffold-10	26973556		C	T
Scaffold-10	26973638		A	C
Scaffold-10	29102427		C	A
Scaffold-10	29102437		T	C
Scaffold-10	29102531	CACACATA		CA
Scaffold-10	29102543		C	T
Scaffold-10	29102545		C	G
Scaffold-10	30368719		G	A
Scaffold-10	30368765	CTA	CTTA,CTC	
Scaffold-10	30368785	CAGC	CAGAGC	
Scaffold-10	30368820		G	C
Scaffold-10	30368829		C	T
Scaffold-10	30368837		AA	GG
Scaffold-10	30368858		G	A
Scaffold-10	33039738		C	T
Scaffold-10	33039741		A	G
Scaffold-10	33039795		CA	CG
Scaffold-10	33039821		G	A
Scaffold-10	33590241		G	C
Scaffold-10	33590275		A	G
Scaffold-10	33590281		A	G
Scaffold-10	33590288		T	C
Scaffold-10	33590326		A	T
Scaffold-10	38828594		T	G
Scaffold-10	38828616		C	A
Scaffold-10	38828629		CG	CA,CAA
Scaffold-10	38828687		G	A
Scaffold-10	42928568		G	A
Scaffold-10	42928596		A	C
Scaffold-10	42928610		CAC	CAA

Scaffold-10	42928625	G	A
Scaffold-10	42928630	A	T
Scaffold-10	42928632	GAAT	GAAG
Scaffold-10	42928636	CTTC	CTTA
Scaffold-10	42928641	G	A
Scaffold-10	42928648	TTTG	TTTA
Scaffold-10	42928661	C	T
Scaffold-10	42928666	G	A
Scaffold-10	42928668	G	A
Scaffold-10	42928672	T	A
Scaffold-10	42928673	C	A
Scaffold-10	42928676	C	A
Scaffold-10	42928685	G	A
Scaffold-10	42928686	C	T
Scaffold-10	42928687	G	A
Scaffold-10	42928691	G	A
Scaffold-10	45191071	C	T
Scaffold-10	45191105	C	T
Scaffold-10	45191141	C	T
Scaffold-10	45191144	G	A
Scaffold-11	3812131	T	A
Scaffold-11	3812164	T	C
Scaffold-11	5343220	G	A
Scaffold-11	5343253	A	G
Scaffold-11	7488372	A	T
Scaffold-11	7488445	T	G
Scaffold-11	8995707	TT	TAT
Scaffold-11	8995767	T	C
Scaffold-11	8995787	G	A
Scaffold-11	9555156	A	C
Scaffold-11	9555165	T	C
Scaffold-11	9555211	T	A
Scaffold-11	9555241	G	A
Scaffold-11	9555267	A	C
Scaffold-11	10970473	A	T
Scaffold-11	10970496	C	A
Scaffold-11	10970537	A	G
Scaffold-11	22281931	G	A
Scaffold-11	22281949	T	G
Scaffold-11	22281961	G	C
Scaffold-11	22708163	G	A
Scaffold-11	22708189	A	G
Scaffold-11	22708210	A	C
Scaffold-11	22708251	G	C

Scaffold-11	24390374		A	G	
Scaffold-11	24390378		G	T	
Scaffold-11	24390401		TT	GC	
Scaffold-11	24390464		T	C	
Scaffold-11	24551825		G	A	
Scaffold-11	24551834		G	A	
Scaffold-11	24551863		C	T	
Scaffold-11	24551873		T	A	
Scaffold-11	24551944		T	G	
Scaffold-11	24551968		T	C	
Scaffold-11	24604626		T	C	
Scaffold-11	24604645	CGTTATTCCTTCTTTG			CG
Scaffold-11	24604673		A	G	
Scaffold-11	24604676		T	C	
Scaffold-11	24604702		G	C	
Scaffold-11	32729920		G	A	
Scaffold-11	32729947		A	G	
Scaffold-11	32729955		C	T	
Scaffold-11	32729960		T	C	
Scaffold-11	32729984		AG	TC	
Scaffold-11	32730036		T	C	
Scaffold-11	33157696		G	A	
Scaffold-11	33157711		C	T	
Scaffold-11	33157723		T	G	
Scaffold-11	33157735		C	A	
Scaffold-11	33157756		A	C	
Scaffold-11	33157757		T	C	
Scaffold-11	33157765		G	A	
Scaffold-11	33157796		A	C	
Scaffold-11	33157821		G	A	
Scaffold-11	33242882		C	T	
Scaffold-11	33242965		C	T	
Scaffold-11	33242983		C	T	
Scaffold-11	33494989		T	G	
Scaffold-11	33494992		C	T	
Scaffold-11	33495002		G	A	
Scaffold-11	33495010		C	A	
Scaffold-11	33495011		C	T	
Scaffold-11	33495041		A	T,G	
Scaffold-11	33495046	ATT	AT		
Scaffold-11	33495051		T	C	
Scaffold-11	33495060		C	A	
Scaffold-11	33495061		T	A	
Scaffold-11	35292280		G	C	

Scaffold-11	35339822		TG	AT
Scaffold-11	35339824		G	C
Scaffold-11	35339841		T	G
Scaffold-11	35339868		C	A
Scaffold-11	35339872		T	C
Scaffold-11	35339879	TGAT	AGAT,AGAG	
Scaffold-11	35339918		C	A
Scaffold-11	35853524		T	A
Scaffold-11	35853535		G	A
Scaffold-11	35853556		G	A
Scaffold-11	35853579		G	A
Scaffold-11	35853589		T	C
Scaffold-11	35853611		A	G
Scaffold-11	37331668		C	T
Scaffold-11	37331683		C	T
Scaffold-11	37331688		A	G
Scaffold-11	37331708		A	G
Scaffold-11	37331727		A	G
Scaffold-11	37430775		T	C
Scaffold-11	37430808		C	A
Scaffold-11	38146734		T	A
Scaffold-11	42417040		A	C
Scaffold-11	42417043	CGAACTTT		
	TGAACTTT,TAAACTTT,CAAACTTT,CGAACTTA,CGATCTTT			
Scaffold-11	42417070		C	T
Scaffold-11	42417084		C	T
Scaffold-11	42417124		G	T,A
Scaffold-11	42417127		G	A
Scaffold-11	42417132		G	T
Scaffold-11	42417146		C	T
Scaffold-11	42417147		G	A
Scaffold-11	42417148		G	A
Scaffold-11	47468361		G	A
Scaffold-11	47468371		C	T
Scaffold-11	47468420		G	T
Scaffold-11	47468471		T	C
Scaffold-12	2078831		T	C
Scaffold-12	4971195		T	A,G
Scaffold-12	4971240		T	C
Scaffold-12	4971295		T	C
Scaffold-12	4971296		G	A
Scaffold-12	5957561		C	A
Scaffold-12	5957582		G	A
Scaffold-12	6043040		A	G

Scaffold-12	6043094		G	A	
Scaffold-12	6043095		G	A	
Scaffold-12	6043110		T	G	
Scaffold-12	12833527		G	T	
Scaffold-12	12833615		A	G	
Scaffold-12	12866879		T	C	
Scaffold-12	12866957		G	A	
Scaffold-12	12866995		C	A	
Scaffold-12	15079193		T	C	
Scaffold-12	15079205		A	G	
Scaffold-12	15079227		G	T	
Scaffold-12	15079271		G	C	
Scaffold-12	24428339		G	A	
Scaffold-12	24428345		G	C	
Scaffold-12	24428351		G	T	
Scaffold-12	24428358		T	C	
Scaffold-12	24428382	AAA	AAG,GAG		
Scaffold-12	24428393		G	A	
Scaffold-12	26466181		G	A	
Scaffold-12	26466243	TAG	TAA		
Scaffold-12	26466265		A	G	
Scaffold-12	26466267		A	C	
Scaffold-12	27139532		T	A	
Scaffold-12	30419146		A	T	
Scaffold-12	30419172	TTAA	ATAA,ATAT		
Scaffold-12	30419196	AGA	ATA		
Scaffold-12	30419210	TTGCT		ATGCT,TTACT	
Scaffold-12	30419217		T	C	
Scaffold-12	30419223		C	T	
Scaffold-12	30419232		A	G	
Scaffold-12	30524624		T	C	
Scaffold-12	30524653	AAAG	AAAA,CAAA		
Scaffold-12	30524661		C	A	
Scaffold-12	30524701		G	T	
Scaffold-12	32537829		C	A	
Scaffold-12	32537847		A	C	
Scaffold-12	33902577		T	C	
Scaffold-12	33902608		T	C	
Scaffold-12	34871833	TTAATAATAATAATAAG			TTAATAATAAAAATAAG
Scaffold-12	43928364		A	T	
Scaffold-12	43928428		C	T	
Scaffold-12	44208321	CAGT	CAGG		
Scaffold-12	44208330	CCCGG		CCCAA	
Scaffold-12	44208348		G	A	

Scaffold-12	44208353		G	T	
Scaffold-12	44208358		C	A	
Scaffold-12	44208368	CAGT	CAGA		
Scaffold-12	44208372	AG	AA		
Scaffold-12	44208396		T	C	
Scaffold-12	44208400		G	A	
Scaffold-12	44208403		C	T	
Scaffold-12	44208407	TAGA	CAGC		
Scaffold-12	44208414	TTTAT	TTAAT		
Scaffold-12	44208420		G	A	
Scaffold-12	44208421	GATCC		AATC	
Scaffold-12	44208429	GAGGT		AAGGC	
Scaffold-12	44208435		G	T	
Scaffold-12	44208442		C	T	
Scaffold-12	44208447		C	T	
Scaffold-12	44208448		T	C	
Scaffold-13	2589865		C	T	
Scaffold-13	2589886		G	A	
Scaffold-13	4680019		GTCGC		GTAGC
Scaffold-13	4680071		C	T	
Scaffold-13	4680092		TGGA	TGGG	
Scaffold-13	4680107		G	A	
Scaffold-13	4680130		G	C	
Scaffold-13	4680169		G	A	
Scaffold-13	4680174		G	A	
Scaffold-13	4680182		G	T	
Scaffold-13	4777589		A	T	
Scaffold-13	4777603		A	G	
Scaffold-13	4777636		T	C	
Scaffold-13	5019832		G	T	
Scaffold-13	5308346		A	G	
Scaffold-13	5308354		GCCTTT		GCCTTG,GCCT,TCCTTG
Scaffold-13	5308360		TGT	TGG	
Scaffold-13	5308394		G	T,A	
Scaffold-13	5308402		T	G	
Scaffold-13	6583448		T	C	
Scaffold-13	6583470		G	A	
Scaffold-13	6583495		A	G	
Scaffold-13	6583512		C	T	
Scaffold-13	6583532		T	G	
Scaffold-13	9080190		A	C	
Scaffold-13	11731304		T	C	
Scaffold-13	11954694		G	C	
Scaffold-13	11954695		A	G	

Scaffold-13	11954722		G	A
Scaffold-13	11954730		C	A
Scaffold-13	11954738		C	T
Scaffold-13	11954743		A	G
Scaffold-13	11954748		T	G
Scaffold-13	11954768		A	G
Scaffold-13	19303569		A	G
Scaffold-13	19303574		G	A
Scaffold-13	19303593		G	A
Scaffold-13	19303601		G	A
Scaffold-13	19303647		G	A
Scaffold-13	19303665		C	T
Scaffold-13	19303669		C	T
Scaffold-13	19303674		T	G
Scaffold-13	19303690		T	G
Scaffold-13	27407275		G	C
Scaffold-13	27407297	GTTTA		GTTTG
Scaffold-13	27407326	ATAAT	ATAAA,ATAAC	
Scaffold-13	27407374		ATC	TTC
Scaffold-13	28311219		T	C
Scaffold-13	28311280		A	T
Scaffold-13	28311317		T	A
Scaffold-13	32008688		C	T
Scaffold-13	32008730		T	C
Scaffold-13	34301093	ATGCTT		ATGCAT
Scaffold-13	34579238		T	C
Scaffold-13	34579249	ATC	AC	
Scaffold-13	34579262		G	A
Scaffold-13	34579301	GA	AG,AA	
Scaffold-13	34579339		T	C
Scaffold-13	35832102		G	A
Scaffold-13	35832174		A	C
Scaffold-13	36686662	CCC	CCA,GCA	
Scaffold-13	36765800		T	C
Scaffold-13	36765801		G	A
Scaffold-13	36765810		A	G
Scaffold-13	36765816		C	G
Scaffold-13	36765830	TGG	GGA	
Scaffold-13	36765837		A	G
Scaffold-13	36765845	ACCAA		ACCAG,GCCAG,ACTAA
Scaffold-13	36765852		T	A
Scaffold-13	36765859	AGAT	GGAC,AGAC	
Scaffold-13	36765881		T	A
Scaffold-13	36765884		C	T

Scaffold-13	36765902		C	G	
Scaffold-13	36765903		T	A	
Scaffold-13	36765910		A	C	
Scaffold-13	36765914	TA	TCA		
Scaffold-13	36765918		T	C	
Scaffold-13	36765924		C	T	
Scaffold-13	36765929		C	G	
Scaffold-13	36765932		G	A	
Scaffold-13	37393198		C	T	
Scaffold-13	37393211		T	C	
Scaffold-13	39818279		T	G	
Scaffold-13	39818299		C	A	
Scaffold-13	40413276		A	G	
Scaffold-13	40413280		C	G,T	
Scaffold-13	40413337		T	A	
Scaffold-13	40413349		A	G	
Scaffold-13	40413355		T	G	
Scaffold-13	40413385		T	C	
Scaffold-13	40413401		G	T	
Scaffold-13	41072785	AAA	CAA,CAC		
Scaffold-13	41429217	TGT	TT		
Scaffold-13	41429221	CA	TA		
Scaffold-13	41429234		T	A	
Scaffold-13	41429248	TCC	TCG		
Scaffold-13	41429261		C	T	
Scaffold-13	41429269	CCAAG		CCAAA	
Scaffold-13	41429274	ATG	ATA		
Scaffold-13	41429291		G	A	
Scaffold-13	41429293	GAAA	GAAG		
Scaffold-13	41429297	CTCCTTTCCA			CTCCTGTCCA
Scaffold-13	41429325		C	T	
Scaffold-13	41429336		TC	TT	
Scaffold-13	41429340		G	A	
Scaffold-13	41887846		G	A	
Scaffold-13	41887856		C	A	
Scaffold-13	41887948	TAAAAAAAAT			TAAAAAAAT
Scaffold-13	41887967		G	A	
Scaffold-13	42724840		G	C	
Scaffold-13	42724842		A	G	
Scaffold-13	42724905		T	C	
Scaffold-13	45231148		T	A	
Scaffold-13	45231155		G	A	
Scaffold-13	45231184		G	A	
Scaffold-13	45231240		C	T	

Scaffold-13	48458590	A	T
Scaffold-13	49898854	C	A
Scaffold-13	49898858	C	A
Scaffold-13	49898881	A	C,T
Scaffold-13	49898893	C	G
Scaffold-13	49898908	T	C
Scaffold-13	49898960	C	T
Scaffold-13	49898961	G	A
Scaffold-13	49898973	G	A
Scaffold-13	49898974	T	G
Scaffold-14	416321	G	C
Scaffold-14	416323	C	A
Scaffold-14	416343	G	A
Scaffold-14	416421	G	A
Scaffold-14	801245	G	T
Scaffold-14	801316	C	A
Scaffold-14	801333	C	A
Scaffold-14	801345	C	T
Scaffold-14	2109950	G	C
Scaffold-14	2109956	GTGG	ATGG
Scaffold-14	2109977	G	T
Scaffold-14	2110001	A	G
Scaffold-14	2110045	T	C
Scaffold-14	6106775	T	C
Scaffold-14	6106799	A	T
Scaffold-14	6106818	GCA	ACA,ACC
Scaffold-14	6106829	G	T
Scaffold-14	7051657	AGTC	GGTG
Scaffold-14	7051701	T	C
Scaffold-14	7051723	G	A
Scaffold-14	7051733	G	A
Scaffold-14	7051739	AT	AC
Scaffold-14	7051760	C	T
Scaffold-14	7051763	G	C
Scaffold-14	7051774	G	A
Scaffold-14	14064268	A	T
Scaffold-14	14064288	C	T
Scaffold-14	14064297	CTAAC	CTAAT
Scaffold-14	14064311	TGT	TT,TAT
Scaffold-14	14064314	GAA	GAG
Scaffold-14	14064340	T	C
Scaffold-14	14326147	C	T
Scaffold-14	14326206	G	C
Scaffold-14	14326227	G	T

Scaffold-14	14326265	A	G
Scaffold-14	14326291	T	G
Scaffold-14	14595373	A	T
Scaffold-14	14595431	G	A
Scaffold-14	14595458	C	G
Scaffold-14	14595464	T	C
Scaffold-14	14595517	A	G
Scaffold-14	14595593	GCTAAG	TCTACT
Scaffold-14	14595610	C	A
Scaffold-14	20395370	A	G
Scaffold-14	20395391	C	T
Scaffold-14	20395400	A	C
Scaffold-14	20395424	A	T
Scaffold-14	20395470	G	A
Scaffold-14	20395471	G	A
Scaffold-14	20518389	A	G
Scaffold-14	24713487	T	C
Scaffold-14	24713513	C	A
Scaffold-14	24713555	G	C
Scaffold-14	24713563	A	G
Scaffold-14	25755237	A	G
Scaffold-14	25755346	T	A
Scaffold-14	25755352	T	G
Scaffold-14	25755374	G	T
Scaffold-14	26698364	GCA	ACA,ACG
Scaffold-14	26698413	CAA	CAAA
Scaffold-14	26698421	C	G
Scaffold-14	26698455	G	C
Scaffold-14	27068800	A	T
Scaffold-14	27068871	G	C
Scaffold-14	33006425	AAAACAAACA	AAAACAAACAAACAAACA
Scaffold-14	33006444	T	C
Scaffold-14	33006448	T	C
Scaffold-14	33006460	AAGAGAGAGAGAGG	AAGAGAGAGAGAGAGG,AAGAGAGAGAGAGAGAGG
Scaffold-14	33006486	G	T
Scaffold-14	33006499	TAGCAAAGCAAAGCAAA	TAGCAAA,TAGCAAAGCAAAGGAAA
Scaffold-14	34350115	C	T
Scaffold-14	34350130	G	T
Scaffold-14	34350195	G	T
Scaffold-14	34350222	C	T
Scaffold-14	34460939	A	G
Scaffold-14	36063202	C	T

Scaffold-14	36063229	G	T
Scaffold-14	37090656	T	C
Scaffold-14	37090710	C	T
Scaffold-14	37090790	G	T
Scaffold-14	37133086	T	A
Scaffold-14	37133121	T	C
Scaffold-14	37133122	T	C
Scaffold-14	37133123	G	T
Scaffold-14	38141830	T	C
Scaffold-14	38141844	C	A
Scaffold-14	38141881	A	T
Scaffold-14	39567559	A	G
Scaffold-14	39567565	T	C
Scaffold-14	40013029	T	G
Scaffold-14	40013036	C	G
Scaffold-14	40013068	T	C
Scaffold-14	40013085	T	C
Scaffold-14	40013095	A	T
Scaffold-14	40013136	T	G
Scaffold-14	40013155	G	A
Scaffold-14	40583952	A	G
Scaffold-14	40583984	C	A
Scaffold-14	41555312	T	C
Scaffold-14	48900534	A	C
Scaffold-14	48900538	T	C
Scaffold-14	48900541	C	T
Scaffold-14	48900577	T	G
Scaffold-14	48900614	T	A
Scaffold-14	48900615	A	G
Scaffold-14	48900621	T	G
Scaffold-14	49935277	G	A
Scaffold-14	49935286	CGGAG	AGGAG
Scaffold-14	49935294	T	G
Scaffold-14	49935322	C	T
Scaffold-14	49935328	C	A
Scaffold-14	49935334	G	A
Scaffold-14	49935336	CAC	CAA
Scaffold-14	49935339	ATAAG	ATAAA
Scaffold-14	49935371	TTTTG	TTTTA
Scaffold-14	49935376	AGG	AGT
Scaffold-14	49935379	CCTG	CCTA
Scaffold-14	49935383	G	A
Scaffold-14	49935386	GTT	GTC
Scaffold-15	1919420	T	C

Scaffold-15	1919428	A	C
Scaffold-15	1919517	T	A
Scaffold-15	2118530	T	A
Scaffold-15	2118531	TGTTC	TGCTC
Scaffold-15	2118552	G	C
Scaffold-15	2118556	AGTTC	AGTTT,AGATT
Scaffold-15	2118566	G	A
Scaffold-15	2118575	T	C
Scaffold-15	2118582	G	A
Scaffold-15	2118589	GCA	GAA,GCG
Scaffold-15	2118600	C	T
Scaffold-15	2118601	A	G
Scaffold-15	2118609	G	A,T
Scaffold-15	5931442	G	A
Scaffold-15	5931468	G	T
Scaffold-15	5931480	ATTTTTTTTG	ATTATTTTG,ATTTTTTTG
Scaffold-15	5931494	A	G
Scaffold-15	5931531	A	G,T
Scaffold-15	7543766	C	T
Scaffold-15	7698324	T	C
Scaffold-15	7698446	C	A
Scaffold-15	7698462	A	C
Scaffold-15	13457939	T	C
Scaffold-15	13457947	T	G
Scaffold-15	13457989	GGGTC	GGATC
Scaffold-15	13457997	G	T
Scaffold-15	13458018	T	C
Scaffold-15	16391721	C	T
Scaffold-15	16391733	C	T
Scaffold-15	16391750	T	C
Scaffold-15	16391773	G	C
Scaffold-15	16391832	A	T
Scaffold-15	16391837	A	T
Scaffold-15	17746038	C	T
Scaffold-15	17746062	A	T
Scaffold-15	17746090	C	T
Scaffold-15	17746095	CAG	CAA,CAAA
Scaffold-15	18311389	GATG	TATG
Scaffold-15	18311437	G	A
Scaffold-15	18311442	CCCAA	CCCAG
Scaffold-15	18311484	GACA	GACG
Scaffold-15	18311507	AC	GC
Scaffold-15	18363751	T	C
Scaffold-15	18363758	C	T

Scaffold-15	20729699	C	A
Scaffold-15	20729756	C	A
Scaffold-15	20729758	T	A
Scaffold-15	20729762	C	A
Scaffold-15	20769735	A	G
Scaffold-15	20769767	A	G
Scaffold-15	20769777	C	T
Scaffold-15	20769791	G	A
Scaffold-15	20769795	G	A
Scaffold-15	20769817	C	T
Scaffold-15	20769828	T	C
Scaffold-15	21903090	A	G
Scaffold-15	21903092	A	T
Scaffold-15	21903094	T	C
Scaffold-15	21903133	C	A
Scaffold-15	24493770	CC	CTT
Scaffold-15	24493820	T	A
Scaffold-15	25806066	C	T
Scaffold-15	26149903	T	A
Scaffold-15	29211261	A	G
Scaffold-15	29211287	G	A
Scaffold-15	29211294	T	G
Scaffold-15	29211314	GCA	GCCA
Scaffold-15	29211332	C	G
Scaffold-15	29211344	G	T
Scaffold-15	29407266	C	G
Scaffold-15	29407273	A	G
Scaffold-15	29407279	C	T
Scaffold-15	29407317	A	C
Scaffold-15	29407319	CCC	ACC,CAC
Scaffold-15	29407324	A	G
Scaffold-15	29407333	C	T
Scaffold-15	29407373	T	A
Scaffold-15	29407390	T	C
Scaffold-15	29407395	A	G
Scaffold-15	32801343	C	T
Scaffold-15	32801369	C	T
Scaffold-15	32801394	G	A
Scaffold-15	32801401	T	C
Scaffold-15	32801416	T	C
Scaffold-15	34607852	A	G
Scaffold-15	34607866	A	G
Scaffold-15	34607914	A	G
Scaffold-15	34607944	T	C

Scaffold-15	34607961	C	A	
Scaffold-15	41029694	A	G	
Scaffold-15	41819427	G	A	
Scaffold-15	41819461	A	T	
Scaffold-15	41819485	G	C	
Scaffold-15	41819534	G	A	
Scaffold-15	43063837	T	C	
Scaffold-15	43063841	G	T	
Scaffold-15	43063873	T	G	
Scaffold-15	43063889	C	G	
Scaffold-15	43063898	C	G	
Scaffold-15	43063912	C	G	
Scaffold-15	43063961	A	T	
Scaffold-15	43063965	CAA	CA	
Scaffold-15	43063973	T	A	
Scaffold-15	43063987	C	T	
Scaffold-15	43063988	G	A	
Scaffold-15	43063995	A	T	
Scaffold-15	43064009	G	A	
Scaffold-15	43064018	A	C	
Scaffold-15	43064027	A	C,G	
Scaffold-15	43064056	CAGAAGAT		CT
Scaffold-15	45106651	T	A	
Scaffold-15	45106653	A	G	
Scaffold-15	45106661	G	T	
Scaffold-15	45106669	TAAA	TATA	
Scaffold-15	45106689	T	C	
Scaffold-15	45106725	A	G	
Scaffold-15	45106726	A	G	
Scaffold-15	45106745	T	G	
Scaffold-15	45106755	A	T	
Scaffold-15	45106765	G	A	
Scaffold-15	48737035	T	C	
Scaffold-15	48737040	A	T	
Scaffold-15	48737066	CCT	CCC	
Scaffold-15	48737069	T	C	
Scaffold-15	48737087	C	T	
Scaffold-15	48737104	C	T	
Scaffold-15	48737118	A	C	
Scaffold-15	48737127	C	T	
Scaffold-15	48737132	G	A	
Scaffold-15	48737133	G	A	
Scaffold-15	48737135	G	A	
Scaffold-16	348902	A	G	

Scaffold-16	348933	G	A	
Scaffold-16	348964	C	T	
Scaffold-16	1053783	T	C	
Scaffold-16	1053789	G	A	
Scaffold-16	1053820	T	A	
Scaffold-16	1053826	G	A	
Scaffold-16	1116867	T	C	
Scaffold-16	1116873	GT	GA,AA	
Scaffold-16	1116875	GCCA	GCCG	
Scaffold-16	1116885	A	T	
Scaffold-16	1116894	CTGAA		TTGAG
Scaffold-16	1116903	T	G	
Scaffold-16	1116904	G	A	
Scaffold-16	1116947	G	C	
Scaffold-16	3518893	C	A	
Scaffold-16	3518896	G	A	
Scaffold-16	3518903	G	A	
Scaffold-16	3518947	G	A	
Scaffold-16	5374965	C	G	
Scaffold-16	5374978	T	C	
Scaffold-16	5374980	T	C	
Scaffold-16	5374997	C	A	
Scaffold-16	5375022	G	A	
Scaffold-16	5375031	T	A	
Scaffold-16	5375036	A	C	
Scaffold-16	5375066	C	T	
Scaffold-16	5375067	G	A	
Scaffold-16	5375079	C	G	
Scaffold-16	5375102	A	T	
Scaffold-16	7225981	G	A	
Scaffold-16	7226035	C	G	
Scaffold-16	8199856	A	T	
Scaffold-16	9502018	T	C	
Scaffold-16	9502043	C	A	
Scaffold-16	9502071	G	T	
Scaffold-16	11900692	C	A,T	
Scaffold-16	11900697	A	G	
Scaffold-16	11900698	C	T	
Scaffold-16	11900718	C	T	
Scaffold-16	11900724	A	G	
Scaffold-16	11900760	G	T	
Scaffold-16	12510775	G	A	
Scaffold-16	12510812	T	C	
Scaffold-16	12510825	C	A	

Scaffold-16	14167980	A	C
Scaffold-16	14167985	G	A
Scaffold-16	14167990	C	A
Scaffold-16	14168010	C	T
Scaffold-16	14168016	GTC	ATT
Scaffold-16	14168030	C	T
Scaffold-16	14168032	C	T
Scaffold-16	14168040	T	G,A
Scaffold-16	14168045	GCTT	GCTC,ACTC
Scaffold-16	14168049	CTG	CTT
Scaffold-16	14168054	C	A
Scaffold-16	14168060	T	C
Scaffold-16	14168067	G	A
Scaffold-16	14168079	C	T
Scaffold-16	14168085	T	C
Scaffold-16	14168087	G	A
Scaffold-16	14168089	G	C,A
Scaffold-16	14639883	C	A
Scaffold-16	14639900	G	C
Scaffold-16	14639928	A	G
Scaffold-16	14639931	T	G
Scaffold-16	14639986	C	G
Scaffold-16	16034174	A	G
Scaffold-16	16034209	C	A
Scaffold-16	16034219	GTC	ATT
Scaffold-16	16034230	A	T
Scaffold-16	16034234	AAGC	CAGT
Scaffold-16	16034286	A	C
Scaffold-16	18864731	A	C
Scaffold-16	23552918	G	A
Scaffold-16	27404649	G	A
Scaffold-16	27404654	TGCTG	TGCTC,CGCTG
Scaffold-16	27404684	C	T
Scaffold-16	27404691	T	A
Scaffold-16	27404709	CTCATCATCAA	CTCATTATCAA,CTCATCAA
Scaffold-16	27404734	TCG	TCCG
Scaffold-16	27977250	G	A
Scaffold-16	28899106	T	C
Scaffold-16	28899141	AG	AT
Scaffold-16	28899202	A	T
Scaffold-16	31771859	T	G
Scaffold-16	31771897	T	C
Scaffold-16	37243501	A	G
Scaffold-16	37243528	A	G

Scaffold-16	37243617	T	C
Scaffold-16	37243618	C	T
Scaffold-16	38274886	T	A
Scaffold-16	38274901	C	G
Scaffold-16	38274902	T	A
Scaffold-16	38274907	C	T
Scaffold-16	38274914	A	G
Scaffold-16	38274930	C	T
Scaffold-16	38274958	ATT	GTT
Scaffold-16	38274977	A	G
Scaffold-16	39319088	ACC	ACT
Scaffold-16	42698896	G	A
Scaffold-16	42698905	C	T
Scaffold-16	42698913	G	T
Scaffold-16	42698920	C	T
Scaffold-16	42698925	C	T
Scaffold-16	42698942	AGAAGCTTAA	AGAAGCTTTG
Scaffold-16	42698973	G	T
Scaffold-16	42698989	ATT	GTT
Scaffold-16	42698996	GGCTTGGT	GGTCTGGA
Scaffold-16	42699008	CG	TA
Scaffold-16	42699019	C	G
Scaffold-16	42699021	G	A
Scaffold-16	44241292	T	A
Scaffold-16	44241304	T	C
Scaffold-16	44241336	T	G
Scaffold-16	44241344	G	A
Scaffold-16	44241359	C	T
Scaffold-16	44241362	T	C
Scaffold-16	44241369	C	G
Scaffold-16	45065935	G	A
Scaffold-16	45065938	C	T
Scaffold-16	45065948	G	A
Scaffold-16	45065952	C	T
Scaffold-16	45065961	AGCAG	GGCAG
Scaffold-16	45065999	AT	GT
Scaffold-16	45066017	AA	AG
Scaffold-16	45066027	G	A
Scaffold-16	45066030	T	C
Scaffold-16	45066037	C	T
Scaffold-16	46330899	G	A
Scaffold-16	46330918	TAAAAAAAC	TAAAAAAAAC
Scaffold-16	46330965	T	C
Scaffold-16	46330968	A	G

Scaffold-16	46330971	T	C	
Scaffold-17	168326	C	G	
Scaffold-17	168332	T	C	
Scaffold-17	168355	A	G	
Scaffold-17	2534402	G	C	
Scaffold-17	2534487	G	A	
Scaffold-17	2534498	T	C	
Scaffold-17	2534541	C	G	
Scaffold-17	2534547	A	T	
Scaffold-17	2534625	G	C	
Scaffold-17	2534640	T	C	
Scaffold-17	2821061	T	A	
Scaffold-17	2821090	A	C	
Scaffold-17	2821144	T	A	
Scaffold-17	5179304	A	G	
Scaffold-17	5179311	ATA	AA	
Scaffold-17	5179320	A	G	
Scaffold-17	5179329	A	G	
Scaffold-17	5179334	C	T	
Scaffold-17	5179339	CTTTTTTA		CTTTTTTTA,TTTTTTTA
Scaffold-17	5179371	C	A	
Scaffold-17	5179424	A	G	
Scaffold-17	7466694	G	T	
Scaffold-17	7466703	A	C	
Scaffold-17	7466724	A	G	
Scaffold-17	7466738	C	T	
Scaffold-17	10810247	T	C	
Scaffold-17	10995242	G	T	
Scaffold-17	11338244	T	G	
Scaffold-17	11338259	T	G	
Scaffold-17	11338276	C	A	
Scaffold-17	11338303	G	A	
Scaffold-17	11338341	GGTT	AGTC	
Scaffold-17	13927537	CCAGGG		CCAGGC
Scaffold-17	13927543	CTCC	CTCA	
Scaffold-17	13927557	C	G	
Scaffold-17	13927563	G	A	
Scaffold-17	13927599	T	C	
Scaffold-17	15925276	C	G	
Scaffold-17	15925316	A	T	
Scaffold-17	15925321	A	G	
Scaffold-17	16397198	C	G	
Scaffold-17	16397311	A	G	
Scaffold-17	22580662	A	G	

Scaffold-17	22580710	G	A
Scaffold-17	22580739	GAT	GT
Scaffold-17	22580746	C	T
Scaffold-17	22765795	G	A
Scaffold-17	22765800	C	G
Scaffold-17	32238750	G	A
Scaffold-17	32238758	A	G
Scaffold-17	32238771	A	T
Scaffold-17	32238801	C	A
Scaffold-17	32238808	G	A
Scaffold-17	32462316	G	A
Scaffold-17	32462341	A	T
Scaffold-17	32462396	G	C
Scaffold-17	32462407	T	C
Scaffold-17	33336821	AAAGA	AAAGAAGA
Scaffold-17	33336834	A	G
Scaffold-17	33336867	T	C
Scaffold-17	33375389	T	G,A
Scaffold-17	33375407	T	A
Scaffold-17	33375412	T	C
Scaffold-17	33725675	A	T
Scaffold-17	33725711	C	A
Scaffold-17	33725716	A	G
Scaffold-17	33725717	T	C
Scaffold-17	33725746	C	A
Scaffold-17	33725796	G	C
Scaffold-17	34143684	C	T
Scaffold-17	34143704	T	C
Scaffold-17	34143705	GTTAGTCATTCCGTCG	GTTAGTCATTCCGTCTTAGTCATTCCGTCG
Scaffold-17	34143774	T	A
Scaffold-17	35551006	TTCAGGCC	TCCAGGCC,TTCAGGCA
Scaffold-17	35551020	C	T
Scaffold-17	35551028	CCAAC	CCAAT
Scaffold-17	35551040	GT	GC,AC
Scaffold-17	35551051	T	C
Scaffold-17	35551068	A	G
Scaffold-17	35551069	T	C
Scaffold-17	35551078	G	A
Scaffold-17	35551088	CGAC	CGAT
Scaffold-17	35736949	C	T
Scaffold-17	35736966	GCCACCACCAC	GTCACCACCAC,GCCGCCACCAC
Scaffold-17	35736978	GG	AC,GC
Scaffold-17	35736980	C	T

Scaffold-17	35737005	A	G	
Scaffold-17	35737017	C	A	
Scaffold-17	36263472	G	A	
Scaffold-17	38028039	T	C	
Scaffold-17	38418151	A	G	
Scaffold-17	38418213	C	T	
Scaffold-17	38418222	T	A	
Scaffold-17	38418239	T	C	
Scaffold-17	39253109	C	T	
Scaffold-17	39253110	C	T	
Scaffold-17	39253152	C	T	
Scaffold-17	39253158	G	A	
Scaffold-17	39253163	C	T	
Scaffold-17	39317307	G	A	
Scaffold-17	39317328	T	C	
Scaffold-17	39317353	A	T	
Scaffold-17	39317402	T	C	
Scaffold-17	46650251	CTCTA		CTGTA
Scaffold-17	49390533	T	C	
Scaffold-17	49390591	T	A	
Scaffold-17	49390598	C	A	
Scaffold-17	49390600	T	C	
Scaffold-17	49390607	T	C	
Scaffold-17	49390625	T	C	
Scaffold-17	49390644	C	A	
Scaffold-17	49390659	C	T	
Scaffold-18	1480471	T	C	
Scaffold-18	1480474	CGTCGGA		CGTCGGT
Scaffold-18	1480486	TTCGT		TTCGA
Scaffold-18	1480502	A	G	
Scaffold-18	1480505	G	C,A	
Scaffold-18	7048719	C	A	
Scaffold-18	7048735	C	T	
Scaffold-18	7048756	G	A	
Scaffold-18	7048777	C	A	
Scaffold-18	7048789	C	T	
Scaffold-18	7048798	A	G	
Scaffold-18	8428064	C	T	
Scaffold-18	8428093	A	G	
Scaffold-18	8657300	C	T	
Scaffold-18	8657319	G	T	
Scaffold-18	8657324	C	T	
Scaffold-18	8657342	A	T	
Scaffold-18	8657349	G	T	

Scaffold-18	8657406	GG	GTT
Scaffold-18	8657420	A	T
Scaffold-18	9081536	A	G
Scaffold-18	9081541	G	T
Scaffold-18	9081566	T	A
Scaffold-18	9081593	T	G
Scaffold-18	9081608	A	G
Scaffold-18	9081614	T	C
Scaffold-18	9081661	A	G
Scaffold-18	9600483	T	A
Scaffold-18	9600523	C	T
Scaffold-18	9600562	G	T
Scaffold-18	9600669	T	C
Scaffold-18	9600712	A	C
Scaffold-18	11754768	AG	AGTTGGTATTAGCGTG
Scaffold-18	11754810	G	A
Scaffold-18	11754823	A	T
Scaffold-18	20428542	C	A
Scaffold-18	20428577	T	G
Scaffold-18	20428621	G	A
Scaffold-18	20428643	G	T
Scaffold-18	20428661	G	A
Scaffold-18	21799338	G	A
Scaffold-18	21799369	G	T
Scaffold-18	24043537	T	G
Scaffold-18	24043543	CCCT	CCCC,TCCC
Scaffold-18	24043553	T	C
Scaffold-18	24043648	A	G
Scaffold-18	24043664	G	A
Scaffold-18	39602880	G	C
Scaffold-18	39602890	A	T
Scaffold-18	39602957	A	G
Scaffold-18	40618592	A	T
Scaffold-18	40618638	C	A
Scaffold-18	42422028	C	T
Scaffold-18	42422043	C	A
Scaffold-18	42422046	G	T
Scaffold-18	42529915	G	A
Scaffold-18	42529926	CA	TT
Scaffold-18	42529949	T	G
Scaffold-18	42529984	C	G
Scaffold-18	42529990	G	A
Scaffold-18	42530005	CGCT	CGCC,AGCC
Scaffold-18	42530024	C	T

Scaffold-18	42530031		G	A
Scaffold-18	42530044		T	C
Scaffold-18	43253033		A	G
Scaffold-18	43253036		C	G
Scaffold-18	43253042		G	C
Scaffold-18	43253065		T	A
Scaffold-18	43253078		A	T
Scaffold-18	43253083		C	T
Scaffold-18	43253100		C	G
Scaffold-18	43253115		A	G
Scaffold-18	44581252		G	T
Scaffold-18	44581294		G	A
Scaffold-18	44581315		T	C
Scaffold-18	47137970		G	C
Scaffold-18	47927618		AAAGAAGG	AAAGG
Scaffold-18	47927632		TAGGGGTGTGTGTTCT	TAGGGGCGTGTGTTCT
Scaffold-18	47927669		G	A
Scaffold-19	35608	C	T	
Scaffold-19	35655	G	C	
Scaffold-19	6151313		A	G
Scaffold-19	6151376		G	T
Scaffold-19	11058009		G	T
Scaffold-19	11058035		G	A
Scaffold-19	11058054		C	T
Scaffold-19	11058075		G	A
Scaffold-19	11058085		C	T
Scaffold-19	11058101		C	T
Scaffold-19	11058112		C	T
Scaffold-19	12386982		A	G
Scaffold-19	12387029		C	A
Scaffold-19	12387051		A	G
Scaffold-19	12387061		A	G,C
Scaffold-19	13462064		C	T
Scaffold-19	13462082		G	A
Scaffold-19	13462093		A	G
Scaffold-19	13462098		A	T
Scaffold-19	13462110		A	G
Scaffold-19	13462116		CCCA	CCCG
Scaffold-19	13462140		C	T
Scaffold-19	13462169		AATT	CAT
Scaffold-19	14720344		T	A
Scaffold-19	14720401		G	A
Scaffold-19	14720428		C	T
Scaffold-19	14720430		G	C

Scaffold-19	14720438	T	G
Scaffold-19	14720454	T	G
Scaffold-19	14720457	C	T
Scaffold-19	18554895	G	T
Scaffold-19	22424504	CGGTT	GGGTT,GGGTC
Scaffold-19	22424523	A	C
Scaffold-19	22424578	G	A
Scaffold-19	22424588	T	C
Scaffold-19	22424596	A	G
Scaffold-19	22424614	G	A
Scaffold-19	23161888	A	C
Scaffold-19	23221076	G	A
Scaffold-19	23221090	A	G
Scaffold-19	23221096	G	A
Scaffold-19	26080677	T	G
Scaffold-19	26080693	GTG	GTA
Scaffold-19	26080706	CTT	CTTT
Scaffold-19	26080722	A	G
Scaffold-19	27407663	A	T
Scaffold-19	27407687	T	C
Scaffold-19	27407691	CTTTTTTTTTC	CTTTTTTTTC,CTTTTTTTC
Scaffold-19	27407742	T	C
Scaffold-19	36199177	T	C
Scaffold-19	36199214	T	A
Scaffold-19	36199271	G	T
Scaffold-19	36199292	G	T,A
Scaffold-19	43820796	T	C
Scaffold-19	43820820	A	G
Scaffold-19	43820823	T	C
Scaffold-19	43820850	C	T
Scaffold-19	43820851	C	T
Scaffold-19	43820901	A	T
Scaffold-19	43855545	GTA	GTC,GAC
Scaffold-19	43855558	G	A
Scaffold-19	43855583	C	T
Scaffold-19	44223682	A	G
Scaffold-19	44223697	G	A,C
Scaffold-19	44223750	A	G
Scaffold-19	44223752	T	G
Scaffold-19	46032722	A	T
Scaffold-19	46032738	G	C
Scaffold-19	46032742	G	T
Scaffold-19	46032778	A	G
Scaffold-19	46032792	T	C

Scaffold-19	46032827	C	T
Scaffold-19	46236475	A	G
Scaffold-19	46236476	C	G
Scaffold-19	46236481	A	C
Scaffold-19	46236486	G	C
Scaffold-19	46236497	AC	CC
Scaffold-19	46236503	TCAAAATCC	CCAAAATCC,CCGAAGTCT
Scaffold-19	46236513	CAA	CA
Scaffold-19	46236525	G	A
Scaffold-19	46236533	T	A
Scaffold-19	46236534	TGA	TGGA
Scaffold-19	46236562	G	T
Scaffold-19	48551183	A	C
Scaffold-19	48551203	G	A
Scaffold-19	48551241	C	A
Scaffold-19	48551247	C	T
Scaffold-19	48551257	T	C
Scaffold-19	48551265	A	G
Scaffold-19	48551274	G	A
Scaffold-19	48551278	A	C
Scaffold-2	600588	C	T
Scaffold-2	600609	A	T
Scaffold-2	600617	T	G
Scaffold-2	600639	GA	GT,CT
Scaffold-2	935977	A	G
Scaffold-2	935981	C	G
Scaffold-2	936000	A	T
Scaffold-2	936016	A	C
Scaffold-2	936047	ATTTT	ATGTT
Scaffold-2	936097	T	A
Scaffold-2	1718256	T	A
Scaffold-2	1718257	C	G
Scaffold-2	1718278	A	G
Scaffold-2	1718284	T	C
Scaffold-2	1718287	ACC	AC
Scaffold-2	1718374	T	C
Scaffold-2	1718378	T	G
Scaffold-2	1718394	ACTC	ACTT
Scaffold-2	1718398	AGAAAAT	AGAAACG,AAAAACG
Scaffold-2	1718405	CT	CC
Scaffold-2	1718421	G	A
Scaffold-2	1718441	C	T
Scaffold-2	1718477	CA	AG
Scaffold-2	1727760	C	T

Scaffold-2	1727786	G	T
Scaffold-2	1727815	G	A
Scaffold-2	1727838	G	T
Scaffold-2	1727864	TCA	TCG
Scaffold-2	1727890	T	C
Scaffold-2	1727895	C	A
Scaffold-2	4864499	G	A
Scaffold-2	4864579	C	T
Scaffold-2	4864603	G	C
Scaffold-2	4864621	A	T
Scaffold-2	7462075	ATCAA	ATCAG
Scaffold-2	7462162	T	C
Scaffold-2	7462178	T	C
Scaffold-2	9202040	A	G
Scaffold-2	9202103	TACTG	TATTG,AACTT
Scaffold-2	9202117	C	A
Scaffold-2	9202121	C	G
Scaffold-2	9202122	G	T
Scaffold-2	9202127	T	G
Scaffold-2	9202130	T	A
Scaffold-2	9202152	G	C
Scaffold-2	9202158	T	A
Scaffold-2	10307478	G	A
Scaffold-2	10307481	GCTG	ACTG,ACTA
Scaffold-2	10307486	T	C
Scaffold-2	10307492	A	T
Scaffold-2	10307498	C	T
Scaffold-2	10307508	A	T
Scaffold-2	10307558	G	T
Scaffold-2	10307564	T	A
Scaffold-2	15193080	A	G
Scaffold-2	15193086	T	A
Scaffold-2	15193102	A	T
Scaffold-2	15193106	A	G
Scaffold-2	15193117	C	T
Scaffold-2	15193141	TAT	TAC,AAC
Scaffold-2	15193153	A	C
Scaffold-2	15193171	T	A
Scaffold-2	15193175	G	C
Scaffold-2	15945125	A	C
Scaffold-2	15945187	C	A
Scaffold-2	15945195	ATCTCTCTCTCA	ATCTCTCTCTCACA
Scaffold-2	15945220	T	A
Scaffold-2	15945227	T	A

Scaffold-2	17070600	ATCGCC	ATCACA
Scaffold-2	17070606	G	A
Scaffold-2	17070620	T	C
Scaffold-2	17070626	G	A
Scaffold-2	17070639	G	T
Scaffold-2	17070641	CAGAC	CAGAA
Scaffold-2	17070646	GCTTTC	TCTTCT
Scaffold-2	17070670	T	A
Scaffold-2	17070676	CGTTGA	CGTTGG
Scaffold-2	17070682	G	A
Scaffold-2	17070690	T	C
Scaffold-2	17070693	T	C
Scaffold-2	17070696	G	A
Scaffold-2	17070697	G	A
Scaffold-2	17070698	CC	AG
Scaffold-2	17070702	G	A
Scaffold-2	17070708	TA	TG
Scaffold-2	17070714	T	A
Scaffold-2	17070716	G	A
Scaffold-2	17070725	C	A
Scaffold-2	17070728	A	G
Scaffold-2	19328198	C	T
Scaffold-2	19328207	A	T
Scaffold-2	23330404	T	C
Scaffold-2	23330406	A	T
Scaffold-2	24196029	G	C
Scaffold-2	24376145	G	A
Scaffold-2	26857363	A	G
Scaffold-2	26857381	T	A
Scaffold-2	26857389	AGC	CGT,AGT
Scaffold-2	26857398	GTT	GTTT
Scaffold-2	26966890	T	C
Scaffold-2	26966891	T	A
Scaffold-2	26966901	C	A
Scaffold-2	26966902	C	A
Scaffold-2	26966908	T	C
Scaffold-2	26966911	A	T
Scaffold-2	26966930	C	T
Scaffold-2	26966944	A	G
Scaffold-2	26966950	GCCA	ACCG
Scaffold-2	26966964	G	T
Scaffold-2	26966971	C	T
Scaffold-2	27414889	C	T
Scaffold-2	27414935	A	G

Scaffold-2	27414957	G	T
Scaffold-2	27414961	C	A
Scaffold-2	34252768	T	A
Scaffold-2	34252779	T	A
Scaffold-2	34542981	C	A
Scaffold-2	34542986	A	G
Scaffold-2	34543061	CAA	CA
Scaffold-2	34906449	C	T
Scaffold-2	34906456	G	T
Scaffold-2	34906508	T	A
Scaffold-2	34906522	A	G
Scaffold-2	34906533	T	C
Scaffold-2	34906540	A	G
Scaffold-2	34906548	G	C
Scaffold-2	36742347	G	T
Scaffold-2	36742361	T	A
Scaffold-2	36742437	A	G
Scaffold-2	41170698	C	T
Scaffold-2	41170737	A	T
Scaffold-2	42223719	C	T
Scaffold-2	43737245	ATTGTGCA	AA
Scaffold-2	43737262	T	C
Scaffold-2	43737281	G	C
Scaffold-2	43737291	G	A
Scaffold-2	43737301	T	G
Scaffold-2	43737328	C	T
Scaffold-2	45754626	A	G
Scaffold-2	45754628	C	G
Scaffold-2	45754642	G	A
Scaffold-2	45754656	C	T
Scaffold-2	45754667	C	G
Scaffold-2	45754669	A	C
Scaffold-2	45754684	G	A
Scaffold-2	45754717	G	T
Scaffold-2	45754738	T	A
Scaffold-2	45754746	G	A
Scaffold-2	48762939	G	T
Scaffold-2	48762991	A	T
Scaffold-2	48763004	T	A
Scaffold-2	48763020	GT	GAT
Scaffold-2	48763032	A	C
Scaffold-2	48763043	C	G
Scaffold-2	49800709	CCAA	TCAG
Scaffold-2	49800735	A	G

Scaffold-2	49800742	G	T	
Scaffold-2	49800748	CATTG		TATTA
Scaffold-2	49800762	T	C	
Scaffold-2	49800778	G	A	
Scaffold-20	558706	T	A	
Scaffold-20	3582891	G	T	
Scaffold-20	3582893	T	G	
Scaffold-20	3582900	G	T	
Scaffold-20	3582909	G	A	
Scaffold-20	3582927	T	C	
Scaffold-20	3582939	G	C	
Scaffold-20	3582961	TT	TTTTGGAAT	
Scaffold-20	3583029	G	A	
Scaffold-20	3583030	T	A	
Scaffold-20	3968125	GTACG		CTACA
Scaffold-20	3968133	A	G	
Scaffold-20	3968139	C	T	
Scaffold-20	3968142	C	T	
Scaffold-20	3968144	A	G	
Scaffold-20	3968150	C	A	
Scaffold-20	3968166	AT	AC	
Scaffold-20	3968181	CTC	CTT	
Scaffold-20	3968184	TATG	TATA	
Scaffold-20	3968189	G	T	
Scaffold-20	3968201	C	T	
Scaffold-20	3968204	C	A	
Scaffold-20	3968213	G	A	
Scaffold-20	3968218	C	T	
Scaffold-20	3968225	A	T	
Scaffold-20	3968226	CGGTT		CGTTG
Scaffold-20	3968231	C	A	
Scaffold-20	3968238	C	T	
Scaffold-20	3968240	G	A	
Scaffold-20	4850567	C	T	
Scaffold-20	4850571	A	T	
Scaffold-20	4850590	G	C	
Scaffold-20	4850593	A	T	
Scaffold-20	4850600	TAT	TT	
Scaffold-20	4850605	G	A	
Scaffold-20	4850620	AAGC	AAGT,GAGT	
Scaffold-20	4850640	C	T,G	
Scaffold-20	4850673	G	A	
Scaffold-20	5112116	G	A	
Scaffold-20	5986196	AA	ACCATCTGA	

Scaffold-20	6555059	A	G	
Scaffold-20	6555095	C	T	
Scaffold-20	6555096	C	A	
Scaffold-20	6555105	AGTA	AGTC,GGTC	
Scaffold-20	6555116	C	A	
Scaffold-20	6555127	C	A	
Scaffold-20	17024916	T	A	
Scaffold-20	17024930	T	A	
Scaffold-20	17024962	CT	TT,TA	
Scaffold-20	19966383	T	C	
Scaffold-20	28666137	C	T	
Scaffold-20	28666147	G	A	
Scaffold-20	28666166	C	T	
Scaffold-20	28666167	C	A	
Scaffold-20	28666209	C	T	
Scaffold-20	31235804	T	A	
Scaffold-20	31235812	A	C	
Scaffold-20	31235826	C	A	
Scaffold-20	31235835	C	A	
Scaffold-20	31235891	T	G	
Scaffold-20	31235893	C	T	
Scaffold-20	31235908	G	A	
Scaffold-20	32330754	C	T	
Scaffold-20	32330760	T	C	
Scaffold-20	32330814	GTCCA		GCCCA
Scaffold-20	34083290	T	G	
Scaffold-20	41582092	G	T	
Scaffold-20	41582134	A	G	
Scaffold-20	41582171	A	C	
Scaffold-20	43631602	C	T	
Scaffold-20	43631652	CC	TT	
Scaffold-20	43631663	C	T	
Scaffold-20	43633507	A	T	
Scaffold-20	47941763	G	A	
Scaffold-20	47941767	C	T	
Scaffold-20	47941780	T	C	
Scaffold-20	47941788	A	G	
Scaffold-20	47941802	T	G	
Scaffold-20	47941810	G	A	
Scaffold-20	47941811	T	A	
Scaffold-20	47941812	G	C	
Scaffold-20	47941820	A	C	
Scaffold-20	47941825	A	T	
Scaffold-20	47941863	A	T	

Scaffold-20	47941864		C	A	
Scaffold-20	47941884		C	T	
Scaffold-20	47941892		T	C	
Scaffold-21	61035	C	G		
Scaffold-21	61053	C	T		
Scaffold-21	61054	A	T		
Scaffold-21	1332754		T	A	
Scaffold-21	1332846		TTTC	TTTT,ATTT	
Scaffold-21	2512879		C	T	
Scaffold-21	2512882		C	A	
Scaffold-21	2512943		T	A	
Scaffold-21	2512965		T	G	
Scaffold-21	2512986		T	C	
Scaffold-21	2513000		C	T	
Scaffold-21	4117440		C	T	
Scaffold-21	4458190		T	C	
Scaffold-21	8006091		G	T	
Scaffold-21	8006099		G	A	
Scaffold-21	8006104		G	A	
Scaffold-21	8006112		A	C	
Scaffold-21	8006121		GC	GA	
Scaffold-21	8006128		C	T	
Scaffold-21	8006137		T	C	
Scaffold-21	8006142		GAAGG		GAAGA,AAAGA
Scaffold-21	8006160		G	A	
Scaffold-21	8006161		T	C	
Scaffold-21	8006199		A	G	
Scaffold-21	11230174		T	A	
Scaffold-21	11230181		G	C	
Scaffold-21	11230196		T	C	
Scaffold-21	11230219		A	C	
Scaffold-21	11230237		G	C	
Scaffold-21	11230246		G	T	
Scaffold-21	13835056		T	A	
Scaffold-21	13835086		G	A	
Scaffold-21	13835100		A	G	
Scaffold-21	13835118		G	T	
Scaffold-21	13835119		G	A	
Scaffold-21	13835134		G	A	
Scaffold-21	13835143		G	A	
Scaffold-21	14836288		A	C	
Scaffold-21	14836298		A	T	
Scaffold-21	14836308		C	T	
Scaffold-21	14836313		T	C	

Scaffold-21	15462383	G	A	
Scaffold-21	15462396	G	T	
Scaffold-21	15462400	A	G	
Scaffold-21	15462403	TACA	CACA	
Scaffold-21	15462409	C	T	
Scaffold-21	15462424	AGTCC		GGTCT
Scaffold-21	15462433	T	C	
Scaffold-21	15462442	T	C	
Scaffold-21	15462460	G	T	
Scaffold-21	15674760	T	C	
Scaffold-21	15674848	G	A	
Scaffold-21	17931857	G	A	
Scaffold-21	17931877	G	A	
Scaffold-21	17931886	C	T	
Scaffold-21	17931891	AGAC	CCAC	
Scaffold-21	17931900	GG	AA	
Scaffold-21	17931913	CAC	GAA	
Scaffold-21	17931921	G	T	
Scaffold-21	17931922	A	C	
Scaffold-21	17931925	T	A	
Scaffold-21	17931930	TTG	GCA	300
Scaffold-21	17931940	G	A	
Scaffold-21	18374554	G	T	
Scaffold-21	18374565	G	A	
Scaffold-21	18374584	CCACCACCAGC	CCACCACCACC,ACACCACCACC,CCACCAGCAGC,CCACCCCCACC,CCACCACCCCC	
Scaffold-21	18374604	A	G	
Scaffold-21	18374673	A	G	
Scaffold-21	19583690	A	G	
Scaffold-21	19583715	C	A	
Scaffold-21	19583725	C	T	
Scaffold-21	19583727	C	T	
Scaffold-21	19583756	C	T	
Scaffold-21	19583762	T	C	
Scaffold-21	19583794	T	C	
Scaffold-21	19583805	C	T	
Scaffold-21	19715905	A	C	
Scaffold-21	19715960	G	A	
Scaffold-21	19715981	T	G	
Scaffold-21	19715990	T	C	
Scaffold-21	21292446	C	T	
Scaffold-21	21292449	C	T	
Scaffold-21	21292465	T	A	

Scaffold-21	21292476	G	C	
Scaffold-21	21292491	C	T	
Scaffold-21	21292522	A	T	
Scaffold-21	21292538	A	T	
Scaffold-21	21292540	T	G	
Scaffold-21	21292561	C	T	
Scaffold-21	21292564	A	T	
Scaffold-21	21297398	G	T	
Scaffold-21	21297403	T	C	
Scaffold-21	21297438	G	A	
Scaffold-21	21297471	CTTTTTTC		CTTTTTC
Scaffold-21	21297499	A	T	
Scaffold-21	21297503	G	T	
Scaffold-21	21297509	T	G	
Scaffold-21	24186784	C	T	
Scaffold-21	24186827	ACAGA		ACAGG
Scaffold-21	24186832	A	C	
Scaffold-21	24186839	TGAC	CGAC,GGAC	
Scaffold-21	24186846	T	C	
Scaffold-21	24186848	G	A	
Scaffold-21	24186849	G	T	
Scaffold-21	24186851	C	G	
Scaffold-21	24186869	G	T	
Scaffold-21	24186882	A	T	
Scaffold-21	24186887	C	T	
Scaffold-21	24186891	GGTAGAGA		GGTCGAGA,AGTCGAGG,GGTCGAGG
Scaffold-21	25603770	T	A	
Scaffold-21	25603787	A	G	
Scaffold-21	25603820	C	G	
Scaffold-21	25603822	A	G	
Scaffold-21	25603836	G	A	
Scaffold-21	25603855	G	A	
Scaffold-21	25603863	T	A	
Scaffold-21	25603865	CTGAAAAGA		CTGAAAAGTGAAAAGA
Scaffold-21	25920369	GG	TT	
Scaffold-21	25920385	G	T	
Scaffold-21	32600945	AA	GT	
Scaffold-21	32600958	T	C	
Scaffold-21	38938766	G	A	
Scaffold-21	38938779	CTTTTTC		CTTTTC
Scaffold-21	38938845	G	A	
Scaffold-21	40253453	GGC	GGA	
Scaffold-21	40253489	A	G	
Scaffold-21	40253522	G	C	

Scaffold-21	40496044	G	A
Scaffold-21	41645531	A	G
Scaffold-21	41645537	A	G
Scaffold-21	41645546	GAA	GA
Scaffold-21	41645549	A	T
Scaffold-21	41864146	A	G
Scaffold-21	41864164	T	C
Scaffold-21	41864185	G	A
Scaffold-21	47929238	G	T
Scaffold-21	47929242	G	C
Scaffold-21	47929245	G	A
Scaffold-21	47929250	T	C
Scaffold-21	47929265	ACAT	GCAT
Scaffold-21	47929289	G	A
Scaffold-21	47929292	G	A
Scaffold-21	47929300	C	T
Scaffold-22	1356546	A	C
Scaffold-22	1356554	A	T
Scaffold-22	1356568	C	G
Scaffold-22	1356571	T	A
Scaffold-22	1356589	T	C
Scaffold-22	1356599	A	C
Scaffold-22	3565742	C	A
Scaffold-22	3565794	A	T
Scaffold-22	3565805	T	C
Scaffold-22	3565825	A	T
Scaffold-22	3565827	G	T
Scaffold-22	4715080	T	C
Scaffold-22	4715108	C	T
Scaffold-22	7226301	T	G
Scaffold-22	7226333	T	C
Scaffold-22	7226366	T	G
Scaffold-22	7226385	A	T
Scaffold-22	7434105	AAAGAAGA	AAAGA,AGAGAAGA
Scaffold-22	7434123	ACTTAT	ACTTATAT
Scaffold-22	7434152	A	G
Scaffold-22	7434177	C	T
Scaffold-22	7434186	GCG	GCC
Scaffold-22	10023489	C	A
Scaffold-22	10023513	C	G
Scaffold-22	10023519	T	C
Scaffold-22	10023546	C	T
Scaffold-22	10023553	T	C
Scaffold-22	10023554	G	C

Scaffold-22	10023555	C	G
Scaffold-22	10023576	A	G
Scaffold-22	10023580	GAAAAAAAC	GAAAAAAAAC
Scaffold-22	10023591	AGA	AA
Scaffold-22	10023594	G	C
Scaffold-22	10023600	A	G
Scaffold-22	10023610	A	G
Scaffold-22	11745884	A	G
Scaffold-22	11745897	G	A
Scaffold-22	11746008	C	T
Scaffold-22	14188284	T	A
Scaffold-22	14188289	A	G
Scaffold-22	14188298	A	G
Scaffold-22	14188322	G	T
Scaffold-22	17382223	C	A
Scaffold-22	17382230	CAT	AAT
Scaffold-22	17382291	C	T
Scaffold-22	21818639	C	T
Scaffold-22	21818660	CA	CC
Scaffold-22	21818673	A	G
Scaffold-22	21818682	G	A
Scaffold-22	21818710	G	A
Scaffold-22	21818716	C	T
Scaffold-22	23608196	A	G
Scaffold-22	23608202	G	A
Scaffold-22	23608218	G	A
Scaffold-22	23608229	T	C
Scaffold-22	23608271	C	A
Scaffold-22	23608286	G	A
Scaffold-22	26998622	G	A
Scaffold-22	26998700	G	A
Scaffold-22	27809860	C	T
Scaffold-22	27809864	A	G
Scaffold-22	27809896	A	G
Scaffold-22	27809910	A	T
Scaffold-22	27809915	C	G
Scaffold-22	27809945	G	T
Scaffold-22	27809950	C	T
Scaffold-22	27809953	A	G
Scaffold-22	27809955	ACA	ACG,GCG
Scaffold-22	27809959	C	A
Scaffold-22	27809966	T	C
Scaffold-22	29515929	A	G
Scaffold-22	29515943	A	G

Scaffold-22	29963301	G	A	
Scaffold-22	29963329	A	G	
Scaffold-22	29963330	G	A	
Scaffold-22	29963360	T	C	
Scaffold-22	34588718	G	A	
Scaffold-22	34588796	T	A	
Scaffold-22	38358766	A	G	
Scaffold-22	38358862	T	G	
Scaffold-22	43532311	A	G	
Scaffold-22	43532347	T	C	
Scaffold-22	43756245	C	T	
Scaffold-22	43756250	C	T	
Scaffold-22	43756269	C	G	
Scaffold-22	43756270	G	A	
Scaffold-22	43756293	T	A	
Scaffold-22	43756328	AGCTT		AGT
Scaffold-22	43756343	C	A	
Scaffold-22	46161245	T	C	
Scaffold-22	47359639	T	C	
Scaffold-22	47359642	GGCG	AGCT	
Scaffold-22	47359658	T	C,A	
Scaffold-22	47359687	C	T	
Scaffold-22	47359700	G	T	
Scaffold-22	47359720	G	T	
Scaffold-22	47359750	T	C	
Scaffold-22	47777237	G	A	
Scaffold-22	47777239	T	G	
Scaffold-22	47777258	T	G	
Scaffold-22	47777264	G	T,A	
Scaffold-22	47777280	G	A	
Scaffold-22	47777292	A	G	
Scaffold-22	47777308	C	T	
Scaffold-22	47999046	C	G	
Scaffold-22	47999069	G	A	
Scaffold-22	47999074	C	G	
Scaffold-22	47999075	C	A	
Scaffold-22	47999077	AGG	AG	
Scaffold-22	47999084	A	T	
Scaffold-22	47999103	C	T	
Scaffold-22	47999111	TGA	TGG	
Scaffold-22	47999122	T	A	
Scaffold-22	47999124	G	C	
Scaffold-22	47999139	A	T	
Scaffold-22	47999167	CTCA	CTCC	

Scaffold-22	47999171	GGG	GGT
Scaffold-22	47999177	C	T
Scaffold-23	268069	T	C
Scaffold-23	268088	G	C
Scaffold-23	268095	ACCTTGAGTGATTGTGGACC	ACCTTGAGTGATTGTAGACC,AC
Scaffold-23	268124	G	A
Scaffold-23	268139	G	C
Scaffold-23	268145	C	G
Scaffold-23	268155	T	C
Scaffold-23	268159	G	T
Scaffold-23	1051955	A	T
Scaffold-23	1051980	T	C
Scaffold-23	1052003	G	A
Scaffold-23	1580295	T	A
Scaffold-23	7206523	A	T
Scaffold-23	7206584	G	T
Scaffold-23	7206610	AACAA	GACAA,GACA
Scaffold-23	9819631	CAC	CAAAA
Scaffold-23	9819656	T	A
Scaffold-23	9819665	T	C
Scaffold-23	10933062	G	C
Scaffold-23	13816333	A	C
Scaffold-23	13816416	A	C
Scaffold-23	13816441	G	C
Scaffold-23	13816469	A	C
Scaffold-23	17033599	C	T
Scaffold-23	17033610	TT	AA
Scaffold-23	17033643	G	A
Scaffold-23	17033678	T	G
Scaffold-23	17033692	A	T
Scaffold-23	17033694	A	T
Scaffold-23	17033708	A	T
Scaffold-23	17033710	G	A
Scaffold-23	17127119	G	A
Scaffold-23	19786428	T	G
Scaffold-23	19786431	A	G
Scaffold-23	21847001	G	A
Scaffold-23	21847013	GCC	GC
Scaffold-23	21847046	C	A
Scaffold-23	21847121	A	G
Scaffold-23	23332301	T	A
Scaffold-23	23332323	A	G
Scaffold-23	26060341	G	A

Scaffold-23	26060354	G	C	
Scaffold-23	27598280	C	T	
Scaffold-23	27598588	A	C	
Scaffold-23	27598668	T	C	
Scaffold-23	27598686	G	A	
Scaffold-23	32568016	C	T	
Scaffold-23	32568043	G	T	
Scaffold-23	32568059	G	C	
Scaffold-23	32568065	G	A	
Scaffold-23	32568070	G	A	
Scaffold-23	32568086	C	G	
Scaffold-23	32568106	A	G	
Scaffold-23	32715013	T	G	
Scaffold-23	32715050	T	A	
Scaffold-23	32715054	T	C	
Scaffold-23	36150507	C	T	
Scaffold-23	36150525	A	G	
Scaffold-23	36150541	T	A	
Scaffold-23	38118431	A	T	
Scaffold-23	38118448	A	G	
Scaffold-23	38118467	T	G	
Scaffold-23	38118487	G	T	
Scaffold-23	38118544	G	T	
Scaffold-23	38118551	A	G	
Scaffold-23	38118562	C	T	
Scaffold-23	38450216	A	G	
Scaffold-23	38450221	G	A	
Scaffold-23	38450222	A	T	
Scaffold-23	38450228	T	C	
Scaffold-23	38450234	C	A	
Scaffold-23	38450238	T	C	
Scaffold-23	38450248	C	T	
Scaffold-23	38450254	G	A	
Scaffold-23	38450268	CCAC	CCAA	
Scaffold-23	38450272	T	C	
Scaffold-23	38450276	AATC	TATG	
Scaffold-23	38450283	G	A	
Scaffold-23	38450285	C	T	
Scaffold-23	38450288	C	A	
Scaffold-23	38450292	GAACTTG		GAACCTG,GACCCTA
Scaffold-23	38450299	TT	TA	
Scaffold-23	38450314	A	T	
Scaffold-23	38450318	CTT	CTTT,CTC	
Scaffold-23	38450324	A	T	

Scaffold-23	38450331	A	G
Scaffold-23	38450340	G	C
Scaffold-23	38450353	T	C
Scaffold-23	40926649	A	G
Scaffold-23	40926668	C	T
Scaffold-23	41390259	G	A
Scaffold-23	41390281	T	C
Scaffold-23	41390306	C	T
Scaffold-23	41390317	G	A
Scaffold-23	41390329	C	G
Scaffold-23	41390356	TCAT	TCATT,TCTT
Scaffold-23	41390367	G	T
Scaffold-23	44913636	T	G
Scaffold-23	44913653	C	A
Scaffold-23	47777956	C	T
Scaffold-23	47777964	T	C
Scaffold-23	47777984	A	G
Scaffold-23	47778039	G	A
Scaffold-23	48913643	G	A
Scaffold-23	48913698	A	G
Scaffold-23	48913749	ATT	ATTT
Scaffold-24	1882604	T	A
Scaffold-24	1882610	T	G
Scaffold-24	3151636	C	G
Scaffold-24	3151708	A	G
Scaffold-24	3151765	C	A
Scaffold-24	3695344	T	C
Scaffold-24	3695351	G	A
Scaffold-24	3695361	T	C
Scaffold-24	3695383	G	C
Scaffold-24	3695385	C	G
Scaffold-24	3695404	C	T
Scaffold-24	3695409	G	T
Scaffold-24	3695416	G	A
Scaffold-24	3695419	TGAC	CGAC,CGAA
Scaffold-24	5412098	T	C
Scaffold-24	8551725	C	T
Scaffold-24	8551744	G	A
Scaffold-24	8551746	C	T
Scaffold-24	8551756	C	A
Scaffold-24	8551776	G	C
Scaffold-24	9672227	G	A
Scaffold-24	9672246	A	C,G
Scaffold-24	9672251	G	A

Scaffold-24	9672267	T	G	
Scaffold-24	9672289	C	G	
Scaffold-24	9672311	A	G	
Scaffold-24	9849096	GG	AG	
Scaffold-24	9849108	C	A	
Scaffold-24	9849169	A	G	
Scaffold-24	10037249	C	T	
Scaffold-24	10037255	G	A	
Scaffold-24	10037316	C	T	
Scaffold-24	10037332	CTTTTTC		CTTTTC
Scaffold-24	10037344	C	G	
Scaffold-24	10037363	T	C	
Scaffold-24	10337466	C	T	
Scaffold-24	10337503	G	C	
Scaffold-24	15106697	A	G	
Scaffold-24	15106721	A	G	
Scaffold-24	15106739	T	A	
Scaffold-24	15106763	A	C	
Scaffold-24	15106772	C	G	
Scaffold-24	15106787	A	G	
Scaffold-24	15106802	A	G	
Scaffold-24	15107243	A	G	
Scaffold-24	15107282	G	A	
Scaffold-24	18539956	G	A	
Scaffold-24	18539957	C	G	
Scaffold-24	18539986	C	A	
Scaffold-24	18539997	ACAC	TCAT	
Scaffold-24	18540006	T	C	
Scaffold-24	18540007	AGG	ATGC	
Scaffold-24	18540054	C	A	
Scaffold-24	19664197	A	G	
Scaffold-24	19664214	G	T	
Scaffold-24	19664233	C	T	
Scaffold-24	19664262	A	G	
Scaffold-24	19664273	TCAA	CCAA	
Scaffold-24	19894403	T	C	
Scaffold-24	19894405	T	C	
Scaffold-24	19894443	T	G	
Scaffold-24	19894447	C	A	
Scaffold-24	19894451	T	C	
Scaffold-24	19894457	A	T	
Scaffold-24	19894476	G	C	
Scaffold-24	21354193	G	A	
Scaffold-24	21354209	G	T	

Scaffold-24	21354221	C	T
Scaffold-24	22449817	G	T
Scaffold-24	22449828	A	T
Scaffold-24	22449848	G	A
Scaffold-24	22449864	G	A
Scaffold-24	22449872	ACGG	CCGA,CTGA,AGGG
Scaffold-24	22449939	C	T
Scaffold-24	23182588	G	C
Scaffold-24	23182593	G	A
Scaffold-24	23182609	G	T
Scaffold-24	23182632	T	C
Scaffold-24	30153100	C	T
Scaffold-24	30153103	T	C
Scaffold-24	30153137	A	T
Scaffold-24	30153139	C	T
Scaffold-24	30153173	G	A
Scaffold-24	30669452	T	G
Scaffold-24	30669458	C	T
Scaffold-24	30669472	G	A
Scaffold-24	30669488	G	A
Scaffold-24	30669489	TACACAGAGAATACATTGAGATAGAAGCACTTTAAAGA	TACACAGAGAAAACATTGAGATAGAAGCACTTTAAAGA,GACACAGAGAATACATTGAGATAGAAGCACTTTAAAGA,TACACAGAGAAAACATTGAGATAGAAGCACTTTAAAAA,TACACAGAGAATACATTGAGATAGAAGCACTTTCAAGA
Scaffold-24	31192093	T	C
Scaffold-24	31192183	C	T
Scaffold-24	31514401	T	A
Scaffold-24	32288526	T	C,A
Scaffold-24	32288550	T	C
Scaffold-24	32288585	CAGAA	TAGAA,TAAAC
Scaffold-24	32288598	G	A
Scaffold-24	32517211	T	A
Scaffold-24	32517231	A	T
Scaffold-24	37203201	C	T
Scaffold-24	37203202	G	A
Scaffold-24	40384989	TTGCTCT	TTGTTCC,TTGCTCC
Scaffold-24	40385012	C	A,T
Scaffold-24	40385024	C	T
Scaffold-24	43275200	C	T
Scaffold-24	43275204	C	G
Scaffold-24	43275216	GGC	GGG
Scaffold-24	43275236	G	T
Scaffold-24	44295692	A	G
Scaffold-24	44295704	C	A

Scaffold-24	44295711	C	T	
Scaffold-24	44295764	A	G	
Scaffold-24	47258353	C	T	
Scaffold-24	49845142	AGGGGGA	AGGGGGAA,AGGGGGG	
Scaffold-24	49845152	A	G	
Scaffold-24	49845230	C	T	
Scaffold-24	49845232	G	A	
Scaffold-24	49847830	A	G	
Scaffold-25	6800259	TTT	ATT	
Scaffold-25	6800274	G	T	
Scaffold-25	6800286	A	T	
Scaffold-25	6800296	G	A	
Scaffold-25	6800300	T	C	
Scaffold-25	6800338	T	C	
Scaffold-25	6800339	T	C	
Scaffold-25	11203600	CTT	CT	
Scaffold-25	11203610	C	T	
Scaffold-25	11203622	CA	CTATATA	
Scaffold-25	11203624	A	C	
Scaffold-25	11203630	G	A	
Scaffold-25	11203633	A	G	
Scaffold-25	11203644	T	C	
Scaffold-25	11203665	T	C	
Scaffold-25	11203672	T	G	
Scaffold-25	11203677	T	A	
Scaffold-25	11203678	TTTAA	TTTAT	
Scaffold-25	11203690	A	T	
Scaffold-25	11203702	C	T	
Scaffold-25	16052442	G	C	
Scaffold-25	16052480	CTT	CT	
Scaffold-25	16052493	G	T	
Scaffold-25	16204449	T	A	
Scaffold-25	17319031	G	A	
Scaffold-25	17319043	T	A	
Scaffold-25	18255064	G	A	
Scaffold-25	18599648	A	T	
Scaffold-25	22195486	C	A	
Scaffold-25	22195556	C	T	
Scaffold-25	22195570	A	G	
Scaffold-25	24668695	T	C	
Scaffold-25	24668743	T	A	
Scaffold-25	26452209	C	A	
Scaffold-25	31146096	G	A	
Scaffold-25	31146131	C	T	

Scaffold-25	31146155	AGCTA	AGCTGCTA,AACTA
Scaffold-25	31146172	C	T
Scaffold-25	31146181	G	A
Scaffold-25	31146183	C	G
Scaffold-25	31146200	ACC	ACCC
Scaffold-25	31710156	T	G
Scaffold-25	31710195	G	T
Scaffold-25	32750854	T	C
Scaffold-25	32750866	C	T
Scaffold-25	32750900	A	G
Scaffold-25	32750907	TAAAAAAAAG	TAAAAAAAG,TATAAAAAAAG,TAAAAAAATG,TAAAAAACG,TAAAAAATAG
Scaffold-25	32750917	CGTAT	CGTAC
Scaffold-25	32750926	TT	TTCGAT
Scaffold-25	32750937	G	T
Scaffold-25	32750951	G	A
Scaffold-25	32750957	C	T
Scaffold-25	32750958	G	A,T
Scaffold-25	32750959	C	T
Scaffold-25	32750979	A	G
Scaffold-25	34131667	T	A
Scaffold-25	34131672	G	T
Scaffold-25	34131673	G	A
Scaffold-25	34131674	T	C
Scaffold-25	34131675	G	A
Scaffold-25	34131676	T	A
Scaffold-25	34131685	G	A,T
Scaffold-25	34131717	C	A
Scaffold-25	36923623	T	A
Scaffold-25	36923634	ATAT	ATAA
Scaffold-25	36923654	A	G
Scaffold-25	40307850	C	A
Scaffold-25	40307877	C	T
Scaffold-25	40307888	C	T
Scaffold-25	40307896	C	T
Scaffold-25	40307899	GTA	GTG,TTG
Scaffold-25	40307959	C	T
Scaffold-25	40598693	CCAGGA	CCAGGC,TCAGGC,CTAGGC,TCAGGT,CCAGAC
Scaffold-25	40598708	A	C
Scaffold-25	40598709	GATTA	GATTC
Scaffold-25	40598725	AACC	AACT
Scaffold-25	40598733	T	C
Scaffold-25	40598756	C	A

Scaffold-25	40598764	G	A
Scaffold-25	40598774	C	T
Scaffold-25	40598782	C	T
Scaffold-25	42514119	A	C
Scaffold-25	42514149	A	G
Scaffold-25	42514202	C	T
Scaffold-25	42514220	C	T
Scaffold-25	42514242	G	A
Scaffold-25	42514253	A	T
Scaffold-25	42514269	A	G,T
Scaffold-25	42514270	G	A
Scaffold-25	42514275	C	T
Scaffold-25	42514308	C	T
Scaffold-25	42514316	T	A
Scaffold-25	42514320	A	T
Scaffold-25	42514385	T	C
Scaffold-25	42514393	A	G
Scaffold-25	49178876	C	T
Scaffold-25	49178894	G	C
Scaffold-25	49446799	A	G
Scaffold-25	49446849	TG	AC
Scaffold-25	49446858	T	C
Scaffold-25	49446865	T	A
Scaffold-25	49446892	G	A
Scaffold-25	49446911	G	A
Scaffold-25	49446919	C	G,A
Scaffold-26	989504	A	G
Scaffold-26	1867813	T	C
Scaffold-26	1867855	C	A
Scaffold-26	1867866	T	C
Scaffold-26	1867877	C	T
Scaffold-26	1867882	C	G
Scaffold-26	1867907	C	T,A
Scaffold-26	1867910	T	C
Scaffold-26	5435305	T	A
Scaffold-26	5435319	C	T
Scaffold-26	5435365	G	A
Scaffold-26	5435379	A	G
Scaffold-26	5435401	C	G
Scaffold-26	5435403	A	G
Scaffold-26	7751052	A	G
Scaffold-26	7751067	C	T
Scaffold-26	7751070	G	A
Scaffold-26	7751087	G	A

Scaffold-26	7751099	G	A
Scaffold-26	7751128	C	T
Scaffold-26	7751138	TT	CA
Scaffold-26	7751155	T	A
Scaffold-26	20429763	C	A
Scaffold-26	20429767	C	T
Scaffold-26	20429800	C	A
Scaffold-26	20429818	G	T
Scaffold-26	20429833	AACCA	CACCA
Scaffold-26	21524385	GCCT	GCCC
Scaffold-26	21524391	G	A
Scaffold-26	21524393	ACG	ATG
Scaffold-26	21524399	AACAG	GACAG
Scaffold-26	21524422	CAC	CAG,CAT
Scaffold-26	21524425	ATTC	ACTC,ATTT
Scaffold-26	21524430	C	A
Scaffold-26	21524449	A	T
Scaffold-26	21524453	GTGTCAGG	GTTTCAGG
Scaffold-26	21524463	GCTTCTGCA	GCTTCTGCCA,TCTTTTGCCA
Scaffold-26	21524478	G	A
Scaffold-26	21524480	TGAC	TGAT
Scaffold-26	21524496	C	T
Scaffold-26	21524502	CCGGA	CCGGC,TCGGC
Scaffold-26	21525185	T	G
Scaffold-26	21525219	C	G
Scaffold-26	21525263	A	G
Scaffold-26	21525272	G	T
Scaffold-26	23403166	C	A
Scaffold-26	23403210	TG	CG,CA
Scaffold-26	25997032	G	A
Scaffold-26	25997039	G	A
Scaffold-26	25997059	G	A
Scaffold-26	25997074	CAT	CAG
Scaffold-26	25997079	G	T
Scaffold-26	25997089	A	G
Scaffold-26	25997090	C	A
Scaffold-26	25997115	G	A
Scaffold-26	25997127	G	A
Scaffold-26	25997128	A	G
Scaffold-26	30025426	TGGA	TGGC,TGGCGGC
Scaffold-26	30025433	G	A
Scaffold-26	30025489	G	A
Scaffold-26	33138865	G	A
Scaffold-26	33138872	G	T,A

Scaffold-26	33138881	A	G
Scaffold-26	33138913	C	G
Scaffold-26	35752394	T	G
Scaffold-26	35752403	C	G
Scaffold-26	35752414	TCA	GTA
Scaffold-26	35752426	TCG	TG
Scaffold-26	35752429	C	T
Scaffold-26	35752430	G	A
Scaffold-26	35752436	T	G
Scaffold-26	35752443	GAATT	GCATG,TCATG
Scaffold-26	35752463	TATAT	CATAT
Scaffold-26	35752491	G	T,C
Scaffold-26	35752515	G	A
Scaffold-26	37319125	C	T
Scaffold-26	37319159	T	G
Scaffold-26	37319198	T	C
Scaffold-26	38290896	TGAG	TGAGAG
Scaffold-26	38290909	A	C
Scaffold-26	38290949	T	C
Scaffold-26	39974915	A	G
Scaffold-26	39974945	GAATACT	GAATACAATACT
Scaffold-26	39974980	C	A
Scaffold-26	39975019	T	C
Scaffold-26	41679238	T	C
Scaffold-26	41679260	T	C
Scaffold-26	42999131	A	G
Scaffold-26	42999226	A	C
Scaffold-26	43194600	G	T
Scaffold-26	43194657	A	G
Scaffold-26	43194681	C	T
Scaffold-26	47307799	GGT	GGA,GAA
Scaffold-26	47307819	T	G
Scaffold-26	47307823	T	C
Scaffold-26	47307835	T	C
Scaffold-26	48856569	G	C
Scaffold-26	48856596	A	G
Scaffold-26	48856619	G	A
Scaffold-26	48856621	T	C
Scaffold-26	48856656	A	T
Scaffold-26	48856658	CGATGGAGATGGAGATGGAG	CGATGGAGATGGAGATGGAA,CGATGGAGATGGAGATGGAGATGGAG,CGATATAGATGGAGATGGAG,CGACGGAGATGGAGATGGAG
Scaffold-26	48856682	C	T
Scaffold-27	1833984	G	A

Scaffold-27	1833988	G	A
Scaffold-27	3580978	C	T
Scaffold-27	3580994	G	T
Scaffold-27	3581003	G	T
Scaffold-27	3581009	T	A
Scaffold-27	3581014	CACTT	CGCTC
Scaffold-27	3581039	G	A
Scaffold-27	3581051	G	T
Scaffold-27	3581058	T	C
Scaffold-27	3581070	C	T
Scaffold-27	6010208	CATAC	CATAATAC
Scaffold-27	6010238	C	T
Scaffold-27	6010249	T	C
Scaffold-27	6010250	T	C
Scaffold-27	6010284	G	C
Scaffold-27	6010297	T	C
Scaffold-27	6010300	G	T
Scaffold-27	6010324	T	C
Scaffold-27	10083130	T	G
Scaffold-27	10083136	C	T
Scaffold-27	10083146	T	C
Scaffold-27	11640696	G	A
Scaffold-27	16469836	C	T
Scaffold-27	16469886	T	G
Scaffold-27	17904256	G	C
Scaffold-27	17904286	A	G
Scaffold-27	17904306	C	T
Scaffold-27	17904318	A	T
Scaffold-27	17904366	C	A
Scaffold-27	27758375	G	T
Scaffold-27	27758376	C	A
Scaffold-27	27758382	G	T
Scaffold-27	27758425	TC	GC
Scaffold-27	27758436	TC	CA,CC
Scaffold-27	27758442	GGG	GGA,AGA
Scaffold-27	27758445	AGG	AGA
Scaffold-27	27758474	GGGC	AGGC
Scaffold-27	27758484	G	A
Scaffold-27	27758494	CG	TG
Scaffold-27	28466884	G	A
Scaffold-27	28466900	G	T
Scaffold-27	28466904	A	G
Scaffold-27	28466937	T	G
Scaffold-27	29865676	CGACGGT	TGACGGC,CGACGGC,CGAAGGT

Scaffold-27	29865727	C	T	
Scaffold-27	29865745	C	A,G	
Scaffold-27	29865760	T	C	
Scaffold-27	32513969	T	C	
Scaffold-27	35957667	A	G	
Scaffold-27	35957669	A	C	
Scaffold-27	35957700	A	G	
Scaffold-27	35957712	A	G	
Scaffold-27	38572769	C	A	
Scaffold-27	38572845	A	T	
Scaffold-27	38572854	T	A	
Scaffold-27	38572884	A	G	
Scaffold-27	39320373	G	A	
Scaffold-27	39320428	CCATCATCATCATC	CCATCATCATCATCATC	
Scaffold-27	40634769	C	T	
Scaffold-27	48632927	GT	GTAT	
Scaffold-27	48632931	GTGT	GTGG,GGGG,TTGG	400
Scaffold-27	48632964	T	G	
Scaffold-27	48633018	G	C	
Scaffold-27	48633019	G	A	
Scaffold-27	48633021	C	T	
Scaffold-27	48633036	T	A	
Scaffold-27	49320135	G	C	
Scaffold-28	5819203	C	G	
Scaffold-28	5819215	C	T	
Scaffold-28	5819219	T	C	
Scaffold-28	5819245	ACTC	GCTC	
Scaffold-28	6757987	A	G	
Scaffold-28	6757989	C	T	
Scaffold-28	6757990	G	A	
Scaffold-28	6757993	G	A	
Scaffold-28	6758041	G	T	
Scaffold-28	6758055	G	A	
Scaffold-28	7358058	T	C	
Scaffold-28	7358065	A	T	
Scaffold-28	7358070	T	A	
Scaffold-28	7358105	T	A	
Scaffold-28	9199540	C	A	
Scaffold-28	9199553	C	G	
Scaffold-28	9199558	ACCGG	GCCGG,GCCGT	
Scaffold-28	9199574	TGC	CGC,TGGC	
Scaffold-28	9199618	C	T	
Scaffold-28	9199630	C	T	
Scaffold-28	11044626	A	T	

Scaffold-28	11044659	TTTCCAT		TTTCCGT
Scaffold-28	11044684	T	A	
Scaffold-28	11044732	A	G	
Scaffold-28	11044735	C	T	
Scaffold-28	12009302	A	G	
Scaffold-28	12009333	T	C	
Scaffold-28	12009335	G	A	
Scaffold-28	12009337	G	T	
Scaffold-28	12009379	A	G	
Scaffold-28	12009382	A	G	
Scaffold-28	12009422	C	T	
Scaffold-28	12009432	T	C	
Scaffold-28	12908213	T	C	
Scaffold-28	15594471	G	A	
Scaffold-28	15594485	A	T	
Scaffold-28	15594495	G	A	
Scaffold-28	16386531	C	T	
Scaffold-28	16386543	A	G	
Scaffold-28	16386545	C	T	
Scaffold-28	16386552	A	G	
Scaffold-28	16386558	G	T	
Scaffold-28	16386562	A	G	
Scaffold-28	16386578	G	A	
Scaffold-28	16386590	C	T	
Scaffold-28	16386594	C	T	
Scaffold-28	16386596	G	A	
Scaffold-28	16386603	GCGCC		GC
Scaffold-28	16386611	G	A	
Scaffold-28	16386635	T	C	
Scaffold-28	19391438	C	T	
Scaffold-28	19391464	T	A	
Scaffold-28	19391477	C	T	
Scaffold-28	19391483	C	T	
Scaffold-28	19391528	T	A	
Scaffold-28	19391538	C	G	
Scaffold-28	19391544	C	T	
Scaffold-28	19954916	G	T	
Scaffold-28	19954940	T	C	
Scaffold-28	19954969	T	G	
Scaffold-28	19954982	C	A	
Scaffold-28	19955010	C	T	
Scaffold-28	20454109	CTG	TTG	
Scaffold-28	20454158	A	G	
Scaffold-28	20837772	T	C	

Scaffold-28	20837777	ATA	AA
Scaffold-28	20837790	G	A
Scaffold-28	20837795	G	A
Scaffold-28	20837799	G	T
Scaffold-28	20837804	T	C
Scaffold-28	20837809	A	T
Scaffold-28	20837841	T	A
Scaffold-28	20837865	G	A,C
Scaffold-28	20837868	G	A
Scaffold-28	20837881	T	C
Scaffold-28	20837902	C	T
Scaffold-28	24985806	A	C
Scaffold-28	24985835	A	G
Scaffold-28	27881153	A	T
Scaffold-28	29137610	T	C
Scaffold-28	29137727	G	A
Scaffold-28	32739108	C	T
Scaffold-28	32739170	T	A
Scaffold-28	32739174	T	C
Scaffold-28	32739179	A	C
Scaffold-28	32739191	C	T
Scaffold-28	33543686	AT	ATCAGAAGT
Scaffold-28	33543723	C	A
Scaffold-28	33543753	A	G
Scaffold-28	34029588	G	T
Scaffold-28	34029604	A	G
Scaffold-28	34029620	T	C
Scaffold-28	34029637	T	C
Scaffold-28	34029659	A	G
Scaffold-28	34029672	T	A
Scaffold-28	34029674	T	C
Scaffold-28	35021226	G	A
Scaffold-28	37366995	CG	CA
Scaffold-28	37367042	TCCGA	ACCGA
Scaffold-28	37367055	ACAG	GCAG,ACAA
Scaffold-28	37367088	TGAC	CGAC,CGAT
Scaffold-28	37367109	C	T
Scaffold-28	37367112	G	C
Scaffold-28	37367123	G	T
Scaffold-28	42700467	G	A
Scaffold-28	42700510	T	A
Scaffold-28	42849346	C	A
Scaffold-28	42849422	T	A
Scaffold-28	43109522	T	C

Scaffold-28	43109559	ATTTTTTTTA	ATTTTTTTTTA
Scaffold-28	43109571	T	A
Scaffold-28	43941894	C	T
Scaffold-28	43941895	A	G
Scaffold-28	43941911	G	A
Scaffold-28	43941937	A	T
Scaffold-28	43941978	G	T
Scaffold-28	44820434	A	T
Scaffold-28	44820437	A	C
Scaffold-28	44820445	A	C
Scaffold-28	44820458	T	A
Scaffold-28	44820463	T	C
Scaffold-28	44820513	C	T
Scaffold-28	44827855	C	T
Scaffold-28	44827864	A	G
Scaffold-28	44827908	A	G
Scaffold-28	44827913	A	T
Scaffold-28	44827945	C	T
Scaffold-28	44827969	T	C
Scaffold-28	44828011	C	T
Scaffold-28	46901046	T	G
Scaffold-28	46901084	T	C
Scaffold-28	46916937	C	G
Scaffold-28	46916942	C	G
Scaffold-28	46916948	C	T
Scaffold-28	46917009	T	A
Scaffold-28	46917016	A	T
Scaffold-28	46917034	T	G
Scaffold-28	46917037	T	A
Scaffold-28	46917061	TCT	TCC,TT
Scaffold-28	48494014	ATTTTTTTTC	ATTTTTTTTTC
Scaffold-28	48494029	C	A
Scaffold-28	48494063	TGG	TG
Scaffold-28	48494114	T	A
Scaffold-28	48494124	C	T
Scaffold-28	49424103	A	C
Scaffold-28	49424109	A	G
Scaffold-28	49424176	G	A
Scaffold-29	288919	GCACA	GCAA
Scaffold-29	288926	G	A
Scaffold-29	288934	G	A
Scaffold-29	288961	AAGCA	AGGTAGGGGTA,AGGTA
Scaffold-29	289007	G	A
Scaffold-29	4589989	C	T

Scaffold-29	4590001	T	A
Scaffold-29	4590034	T	G
Scaffold-29	4590052	C	T
Scaffold-29	4590078	T	A
Scaffold-29	4590091	G	A
Scaffold-29	5725118	A	G
Scaffold-29	5725125	G	A
Scaffold-29	5725127	G	T
Scaffold-29	5725167	A	T
Scaffold-29	5725182	C	T
Scaffold-29	5725255	A	G
Scaffold-29	5725277	C	G
Scaffold-29	5725347	A	G
Scaffold-29	5761781	T	C
Scaffold-29	5761799	C	G
Scaffold-29	5761826	TACG	GACG
Scaffold-29	5761841	C	T
Scaffold-29	5761865	T	C
Scaffold-29	5761871	C	A
Scaffold-29	7746337	G	T
Scaffold-29	12885582	G	T
Scaffold-29	12885583	CCA	CCC
Scaffold-29	12885600	AACTTC	AACTTT
Scaffold-29	12885620	A	T
Scaffold-29	12885636	AAAG	ACAG
Scaffold-29	12885657	G	A
Scaffold-29	12885693	TCAAT	GCAAT
Scaffold-29	12885698	C	T
Scaffold-29	12885702	G	A,C
Scaffold-29	13749124	G	C
Scaffold-29	13749137	G	C
Scaffold-29	13749158	T	G
Scaffold-29	13749219	G	C
Scaffold-29	14677972	T	G
Scaffold-29	14677989	C	T
Scaffold-29	14678047	C	A
Scaffold-29	14678049	TGG	TG,TGA
Scaffold-29	20665198	TAAAAAAAC	AAAAAAAAC,TAAGAAAAC,TAAAAAAC
Scaffold-29	20665240	A	G
Scaffold-29	20665252	A	T
Scaffold-29	20665270	G	A
Scaffold-29	21010930	GCCG	ACCG
Scaffold-29	21010937	G	T
Scaffold-29	21010938	C	A

Scaffold-29	21010947	T	G
Scaffold-29	21010975	CAGAG	CAGAA
Scaffold-29	21010987	G	T
Scaffold-29	21010990	A	G
Scaffold-29	21010993	T	C
Scaffold-29	21010997	G	T
Scaffold-29	21011022	G	A
Scaffold-29	21011028	GAGG	GAGA,GAG,GGGG
Scaffold-29	21011032	C	G
Scaffold-29	21011035	G	A
Scaffold-29	23479337	C	G
Scaffold-29	24988049	G	A
Scaffold-29	24988056	AAA	AGT,GGT
Scaffold-29	24988072	G	A
Scaffold-29	24988075	C	T
Scaffold-29	24988080	C	T
Scaffold-29	24988084	CT	CC,TC
Scaffold-29	24988098	G	A
Scaffold-29	24988105	C	T
Scaffold-29	24988117	G	A
Scaffold-29	24988130	C	T
Scaffold-29	24988143	G	A
Scaffold-29	24988149	TGGCA	CGGCA,CGGCG
Scaffold-29	25371738	A	G
Scaffold-29	25371756	T	G
Scaffold-29	25371771	C	G
Scaffold-29	25371779	A	T
Scaffold-29	25371812	T	G
Scaffold-29	25371817	A	G
Scaffold-29	25371822	C	A
Scaffold-29	25371855	G	A
Scaffold-29	29487507	A	G
Scaffold-29	29842799	G	A
Scaffold-29	29842831	T	C
Scaffold-29	29842840	A	T
Scaffold-29	29842869	A	T
Scaffold-29	29842881	C	G
Scaffold-29	29842890	C	T
Scaffold-29	29842899	TGA	CGA
Scaffold-29	29842925	TCG	TG
Scaffold-29	32615949	A	G
Scaffold-29	33505285	T	C
Scaffold-29	33505291	C	T
Scaffold-29	33505379	T	A

Scaffold-29	35354417	C	T
Scaffold-29	35354432	C	A
Scaffold-29	35354446	A	T
Scaffold-29	35354461	T	C
Scaffold-29	35354477	A	G
Scaffold-29	35354521	G	C
Scaffold-29	36779439	T	C
Scaffold-29	36779459	T	C
Scaffold-29	36779505	A	C
Scaffold-29	36779561	TT	TGT,TA
Scaffold-29	36779579	T	G
Scaffold-29	37274635	G	A
Scaffold-29	37274677	TAAAAAC	TAAAAC
Scaffold-29	37274701	A	G
Scaffold-29	37274714	C	G
Scaffold-29	37274726	A	G
Scaffold-29	38219987	C	T
Scaffold-29	38220017	G	A
Scaffold-29	38220091	C	G
Scaffold-29	38220098	A	G
Scaffold-29	38220101	G	A
Scaffold-29	40332610	G	A
Scaffold-29	40332620	A	T
Scaffold-29	40332626	T	G
Scaffold-29	40332636	C	T
Scaffold-29	40332641	T	C
Scaffold-29	40332675	A	G
Scaffold-29	40332681	TCG	CCG
Scaffold-29	40332689	G	T
Scaffold-29	40332703	G	A
Scaffold-29	40975579	TTTTT	TTTTG
Scaffold-29	40975595	A	C
Scaffold-29	43703387	G	A
Scaffold-29	43703423	T	C
Scaffold-29	43703429	G	A
Scaffold-29	43703450	G	C
Scaffold-29	43703459	A	G
Scaffold-29	43703468	T	G
Scaffold-29	43792530	A	G
Scaffold-29	43792575	ACTAA	ACTAC
Scaffold-29	43792580	TTTC	TTTT
Scaffold-29	43792598	T	G
Scaffold-29	43792602	T	C
Scaffold-29	43792606	C	A

Scaffold-29	43792616	G	T
Scaffold-29	43792630	T	C
Scaffold-29	43792638	C	A
Scaffold-29	43929164	G	A
Scaffold-29	43929179	G	A
Scaffold-29	43929194	C	T
Scaffold-29	43929237	T	G
Scaffold-29	43929250	AGG	AG
Scaffold-29	43929262	T	C
Scaffold-29	49600292	C	T
Scaffold-29	49600298	T	A
Scaffold-3	1958881	T	C
Scaffold-3	1958920	T	A
Scaffold-3	1958958	TT	TTAT
Scaffold-3	2109479	A	G
Scaffold-3	2109527	T	C
Scaffold-3	2109545	C	T
Scaffold-3	16963957	C	A
Scaffold-3	18688389	C	T
Scaffold-3	18688426	G	C
Scaffold-3	18688475	G	C
Scaffold-3	18688492	A	T
Scaffold-3	18688493	G	A
Scaffold-3	20674001	GGTGT	AGTGT,AGTGC
Scaffold-3	26654658	A	C
Scaffold-3	26654663	G	A
Scaffold-3	26654664	T	C
Scaffold-3	26654666	T	A
Scaffold-3	26654670	C	T
Scaffold-3	26654673	A	G
Scaffold-3	26654677	CG	TG,TA
Scaffold-3	26654693	ATT	ATTT
Scaffold-3	26654714	T	C
Scaffold-3	26654723	ATT	AT
Scaffold-3	26654731	G	A
Scaffold-3	26654732	T	C
Scaffold-3	26654734	A	C
Scaffold-3	26654743	A	G
Scaffold-3	26654767	G	A
Scaffold-3	33611811	T	A
Scaffold-3	33611816	TACACACACA	TACACACA
Scaffold-3	33611875	C	T
Scaffold-3	35617200	G	T
Scaffold-3	35617234	G	A

Scaffold-3	35617244	G	A
Scaffold-3	35617249	GA	GATATA
Scaffold-3	35617278	A	G
Scaffold-3	35617279	G	A
Scaffold-3	35617280	T	G
Scaffold-3	35617284	C	T
Scaffold-3	35617299	T	G
Scaffold-3	35617309	A	G
Scaffold-3	37725646	A	T
Scaffold-3	37725650	C	A
Scaffold-3	37725654	A	G
Scaffold-3	37725718	A	C
Scaffold-3	38010160	T	G
Scaffold-3	38010173	T	C
Scaffold-3	38010192	C	T
Scaffold-3	38010236	C	A
Scaffold-3	38010283	T	G
Scaffold-3	38010291	G	A
Scaffold-3	38313885	A	G
Scaffold-3	38313918	G	C
Scaffold-3	38313920	T	G
Scaffold-3	38313940	C	T
Scaffold-3	38313951	G	C
Scaffold-3	38859467	T	C
Scaffold-3	38859473	A	G
Scaffold-3	38859479	G	T
Scaffold-3	38859488	G	A
Scaffold-3	38859499	T	G
Scaffold-3	38859526	A	C
Scaffold-3	38859549	G	T
Scaffold-3	38859558	G	T
Scaffold-3	38859568	C	T
Scaffold-3	43515856	A	G
Scaffold-3	43515905	T	A
Scaffold-3	44496267	ATCC	TTCT
Scaffold-3	44496301	G	A
Scaffold-3	45320939	TGG	TGA
Scaffold-3	46490618	A	T
Scaffold-3	46490633	C	T
Scaffold-3	46490742	G	A
Scaffold-3	46490743	G	A
Scaffold-30	1351890	G	A
Scaffold-30	1351902	T	C
Scaffold-30	1351912	G	A

Scaffold-30	1351921	T	C	
Scaffold-30	1351939	G	A	
Scaffold-30	3176811	AGTG	AGTC	
Scaffold-30	3176815	CGAT	AGAT	
Scaffold-30	3176841	G	T	
Scaffold-30	3176870	C	T	
Scaffold-30	3176940	C	A	
Scaffold-30	3450887	A	C	
Scaffold-30	3450926	C	T	
Scaffold-30	3450944	G	A	
Scaffold-30	3450967	GTT	GTC,ATC	
Scaffold-30	3450994	CCATG		CCATT
Scaffold-30	3850495	T	C	
Scaffold-30	3850558	C	T	
Scaffold-30	3850582	G	A	
Scaffold-30	5576835	CGC	CGT	
Scaffold-30	5576838	CG	CA	
Scaffold-30	5576842	G	A	
Scaffold-30	5576863	C	T	
Scaffold-30	5576864	A	G	
Scaffold-30	5576876	G	C	
Scaffold-30	5576908	G	A	
Scaffold-30	6215023	A	C	
Scaffold-30	6215125	CGG	TGA	
Scaffold-30	6215135	C	G	
Scaffold-30	6817276	A	G	
Scaffold-30	6817335	A	G	
Scaffold-30	6817341	G	C	
Scaffold-30	6817379	A	G	
Scaffold-30	6817397	C	T	
Scaffold-30	16548708	C	G	
Scaffold-30	16548731	A	G	
Scaffold-30	17976837	A	C	
Scaffold-30	17976921	T	A	
Scaffold-30	17976956	C	A	
Scaffold-30	20231998	T	A	
Scaffold-30	20232011	G	C	
Scaffold-30	20232020	A	C	
Scaffold-30	20232031	C	A	
Scaffold-30	20232041	A	C	
Scaffold-30	20232047	A	C	
Scaffold-30	20232085	CG	GT	
Scaffold-30	20232092	ATA	ATG	
Scaffold-30	20232098	G	C	

Scaffold-30	23008801	CGTA	TGTA
Scaffold-30	23008906	T	C
Scaffold-30	27803651	T	C
Scaffold-30	27803683	G	A
Scaffold-30	27803685	A	T
Scaffold-30	27803711	TAA	TGA
Scaffold-30	28695366	G	T
Scaffold-30	28695378	C	A
Scaffold-30	28695381	C	T
Scaffold-30	28695416	G	T
Scaffold-30	28695448	C	G
Scaffold-30	31123685	GTTTTCAAACCTCCCACAAT	GTTTGCAAACCTCCCACAAT,GTTTTCAACCTCCCACAAT
Scaffold-30	31123760	G	A
Scaffold-30	33788452	AGGGCT	AT
Scaffold-30	33834057	G	T
Scaffold-30	33834080	A	G
Scaffold-30	33834081	G	A
Scaffold-30	33834098	C	A
Scaffold-30	33834133	G	A
Scaffold-30	39402912	C	T
Scaffold-30	39402931	C	T
Scaffold-30	39402932	G	A
Scaffold-30	39402964	A	G
Scaffold-30	39402974	G	C
Scaffold-30	39402985	TA	TG,CG
Scaffold-30	39403073	C	T
Scaffold-30	39403074	C	A
Scaffold-30	39403099	T	C
Scaffold-30	40722320	C	T
Scaffold-30	40722321	G	A
Scaffold-30	40722322	G	A
Scaffold-30	40722372	T	C
Scaffold-30	40722381	A	G
Scaffold-30	40722400	TCT	TCG
Scaffold-30	40722411	G	T
Scaffold-30	43511496	G	T
Scaffold-30	43511502	CAG	CAAG
Scaffold-30	43511543	G	A
Scaffold-30	43511619	G	C
Scaffold-30	43554662	C	A
Scaffold-30	43554677	C	A
Scaffold-30	43554710	T	A
Scaffold-30	43554728	C	A

Scaffold-30	48181305	T	C
Scaffold-30	48181314	A	G
Scaffold-30	48181322	C	T
Scaffold-30	48181333	A	G
Scaffold-30	48181338	T	G
Scaffold-31	9779089	GGAAA	CGAAA
Scaffold-31	9779094	G	T
Scaffold-31	9779124	TAAT	TAAA
Scaffold-31	9779154	G	C
Scaffold-31	17676619	C	T
Scaffold-31	17676624	T	C
Scaffold-31	17676639	A	C
Scaffold-31	17676678	C	T
Scaffold-31	18226733	A	G
Scaffold-31	18226755	A	G
Scaffold-31	18226765	G	A
Scaffold-31	18226792	CTCAATCAATCAATCAATCAATCAATCAATCAAT	CTCAATCCATCAATCAATCAATCAATCAAT,CTCAATCAATCAATCAATCAATCAATCAAT,CTCAATCCATCAATCAATCAATCAATCT,CTCAATCCATCAATCAATCAATCAATCAATCAAT
Scaffold-31	19406648	A	C
Scaffold-31	19406686	T	C
Scaffold-31	19406721	G	C,A
Scaffold-31	19406725	C	T
Scaffold-31	19406780	T	C
Scaffold-31	22096080	A	G
Scaffold-31	22096096	T	C
Scaffold-31	22096115	G	A
Scaffold-31	22096128	TTAAAAAAAAGTTTTAT	TTAAAAAAAAAGTTTTAT,TT
Scaffold-31	22158892	A	G
Scaffold-31	22158897	A	C
Scaffold-31	22158903	C	T
Scaffold-31	22158904	G	A
Scaffold-31	25702815	C	T
Scaffold-31	25702830	T	C
Scaffold-31	25702844	G	A
Scaffold-31	25702847	AGGTG	GGGTG,GGGTA
Scaffold-31	25702857	GT	AT
Scaffold-31	25702864	G	A
Scaffold-31	25702867	G	A
Scaffold-31	25702875	G	A
Scaffold-31	25702891	A	G
Scaffold-31	25702898	C	G

Scaffold-31	25702904	T	C	
Scaffold-31	25702936	G	A	
Scaffold-31	25702939	G	A	
Scaffold-31	26511799	A	C	
Scaffold-31	26511823	G	C	
Scaffold-31	26511834	T	C	
Scaffold-31	26511848	T	C	
Scaffold-31	26511886	C	T	
Scaffold-31	26511892	T	C	
Scaffold-31	30189078	C	T	
Scaffold-31	30189117	A	G	
Scaffold-31	30189153	C	T	
Scaffold-31	30189195	A	G	
Scaffold-31	31288142	A	T	
Scaffold-31	31288144	T	A	
Scaffold-31	31288161	C	A	
Scaffold-31	31288173	A	G	
Scaffold-31	31288190	A	G	
Scaffold-31	31288192	A	G	
Scaffold-31	32755506	C	T	
Scaffold-31	32755518	A	G	
Scaffold-31	32755528	C	T	
Scaffold-31	32755534	A	G	
Scaffold-31	32755536	A	C	
Scaffold-31	32755558	C	G	
Scaffold-31	32755578	T	C	
Scaffold-31	32755631	AAGGCAG		GAGGCAG,GAGG
Scaffold-31	33349423	GGAG	GGAA	
Scaffold-31	33349427	ATTA	ATTC	
Scaffold-31	33349431	C	G	
Scaffold-31	33349458	C	T	
Scaffold-31	33349484	GGTTC		CGTTC
Scaffold-31	33349490	GACA	GACC	
Scaffold-31	33349494	GTTG	ATTG	
Scaffold-31	33349498	G	T	
Scaffold-31	33349504	T	G	
Scaffold-31	33349505	CAT	TAC	
Scaffold-31	33349513	GGCCT		TGCCC
Scaffold-31	33349540	GC	AA	
Scaffold-31	33390689	T	C	
Scaffold-31	33390697	ATT	AT	
Scaffold-31	33390737	A	C	
Scaffold-31	35417495	A	G	
Scaffold-31	35417500	C	A	

Scaffold-31	35417514	A	C,T
Scaffold-31	35417546	GTCG	GTCT,GTCA
Scaffold-31	35417550	TA	TG
Scaffold-31	36223378	A	G
Scaffold-31	36223393	G	A
Scaffold-31	36223403	T	G
Scaffold-31	36223411	C	T
Scaffold-31	36223417	C	T
Scaffold-31	36223431	C	T
Scaffold-31	36223436	C	G
Scaffold-31	36223437	C	A
Scaffold-31	36223444	C	T
Scaffold-31	36447647	C	T
Scaffold-31	36447653	C	T
Scaffold-31	36447669	CAACC	TAACG,CAACT
Scaffold-31	36447685	T	G
Scaffold-31	36447697	C	T
Scaffold-31	36447706	T	A
Scaffold-31	36447732	C	T
Scaffold-31	37153443	A	G
Scaffold-31	37153447	C	T
Scaffold-31	37153456	G	A
Scaffold-31	37153477	T	A
Scaffold-31	37153495	G	A
Scaffold-31	37153540	A	G
Scaffold-31	37153571	C	T
Scaffold-31	37153576	G	T
Scaffold-31	37153579	G	T
Scaffold-31	39285734	A	G
Scaffold-31	39742767	G	A
Scaffold-31	39742785	G	A
Scaffold-31	39742795	A	G
Scaffold-31	39742830	G	T
Scaffold-31	39742841	C	T
Scaffold-31	39742846	T	C
Scaffold-31	39742867	T	A
Scaffold-31	39742874	GGGAA	GGGAAGAA,GGGAGGAA
Scaffold-31	39742890	A	G
Scaffold-31	39742898	G	A
Scaffold-31	41067670	G	A
Scaffold-31	41067672	G	C
Scaffold-31	41067677	G	A
Scaffold-31	41067768	T	G
Scaffold-31	41067772	G	A

Scaffold-31	42933772	AT	AC
Scaffold-31	42933835	A	T
Scaffold-31	42933856	ATATAA	ATAAAT,AGAAAA,GTAAAT
Scaffold-31	45278015	C	T
Scaffold-31	45278036	C	A
Scaffold-31	45278077	GCTTTTCTA	GCTTTTCTC
Scaffold-31	45278120	G	A
Scaffold-31	45278121	C	A
Scaffold-31	45278127	ACG	ATG
Scaffold-31	45294062	A	G
Scaffold-31	45294088	C	T
Scaffold-31	45294114	C	A
Scaffold-31	45294156	CTGT	CTTT,CT
Scaffold-31	45294189	T	C
Scaffold-31	47837190	C	T
Scaffold-31	47837229	TAAAAAAAAAC	TAAAAAAAAC,TAAAAAACAAC
Scaffold-31	49044593	C	T
Scaffold-31	49044598	G	A
Scaffold-31	49044605	C	G
Scaffold-31	49044634	G	T
Scaffold-31	49044643	G	T
Scaffold-32	914002	ATTTTTTTTA	ATTTTTTTA
Scaffold-32	914020	A	G
Scaffold-32	914076	AC	GC
Scaffold-32	914080	G	A
Scaffold-32	4439127	T	C
Scaffold-32	4439154	T	C
Scaffold-32	4439162	G	T
Scaffold-32	7692666	C	T
Scaffold-32	7692673	T	A
Scaffold-32	7692707	T	A
Scaffold-32	7692713	T	G
Scaffold-32	7692743	G	T
Scaffold-32	7692754	G	T
Scaffold-32	7692759	G	A
Scaffold-32	9856976	A	T
Scaffold-32	9857040	A	G
Scaffold-32	9857067	CTT	CT
Scaffold-32	11202633	C	A
Scaffold-32	11202635	C	A
Scaffold-32	11202678	T	C
Scaffold-32	14080250	G	A
Scaffold-32	18408289	A	G
Scaffold-32	18408299	C	G

Scaffold-32	18408324	C	G	
Scaffold-32	18408337	G	A	
Scaffold-32	18408350	CCTGGGT	CCTGGGC,CCTGAGC	
Scaffold-32	18408362	T	C	
Scaffold-32	18408375	G	T	
Scaffold-32	18408376	A	G	
Scaffold-32	18408386	GGAT	AGAT	
Scaffold-32	18408399	T	A	
Scaffold-32	18408406	A	C	
Scaffold-32	18408409	G	A	
Scaffold-32	18408410	T	C	
Scaffold-32	21201767	T	C	
Scaffold-32	21201768	A	G	
Scaffold-32	21201773	C	T	
Scaffold-32	21201852	T	C	
Scaffold-32	24280081	CCAGTCTTC	CCGGTCGTT	
Scaffold-32	24280096	A	G	
Scaffold-32	24280112	G	C	
Scaffold-32	24280117	A	C	
Scaffold-32	24280118	G	A	
Scaffold-32	24280123	C	T	
Scaffold-32	24280145	G	T,A	
Scaffold-32	24280163	C	A	
Scaffold-32	24280176	GTT	ATA	
Scaffold-32	24580443	T	C	
Scaffold-32	24580454	C	A	
Scaffold-32	24580487	G	T	
Scaffold-32	24580490	A	T	
Scaffold-32	27036794	G	A	
Scaffold-32	27036806	T	C	
Scaffold-32	27036846	A	G	
Scaffold-32	27056795	T	A	
Scaffold-32	27056799	TAA	TA	
Scaffold-32	27056802	CC	CT	
Scaffold-32	27056813	T	G	
Scaffold-32	27056816	T	C	
Scaffold-32	27056817	G	T	
Scaffold-32	27056851	A	G	
Scaffold-32	27056864	G	T	
Scaffold-32	28927879	T	G	
Scaffold-32	28927882	AACCGTTA	AACCGTTG,AACCATTG,AACCGTCA	500
Scaffold-32	28927899	C	T	
Scaffold-32	28927912	G	A	
Scaffold-32	28927913	A	T	

Scaffold-32	28927920	CGAAACCTGGGC	CGACACCTGGGC,CGATACCCGGGC,CGATACCCGGGA,CGACACCAGGGC,CAACACCCGGGC,CGGAACCTGGGC,CGATACCTGGGC
Scaffold-32	28927959	TTGGA	TTGGG,TTAGA
Scaffold-32	28927999	CG	CAG
Scaffold-32	29544315	C	A
Scaffold-32	29544317	CGG	CGT
Scaffold-32	29544337	C	A
Scaffold-32	29544340	C	T
Scaffold-32	29544362	GAC	CAC,CAG
Scaffold-32	29544396	A	C
Scaffold-32	34802814	T	A
Scaffold-32	37828526	G	A
Scaffold-32	37828537	G	C
Scaffold-32	37828574	T	C
Scaffold-32	37828619	G	A
Scaffold-32	37828659	T	C
Scaffold-32	41264764	CGCC	GGCC
Scaffold-32	41264792	CAAC	AAAC
Scaffold-32	48966592	A	T
Scaffold-32	48966634	G	A
Scaffold-32	48966662	C	T
Scaffold-32	49700017	T	G
Scaffold-33	1429151	T	A
Scaffold-33	1429235	ATTTTTTTTA	ATTTTTTTTTTA,ATTTTTTTTTA,ATTTTTTTTTTTA
Scaffold-33	1429246	A	G
Scaffold-33	1429257	C	A
Scaffold-33	1429281	T	C
Scaffold-33	1952734	T	C
Scaffold-33	1952735	G	T
Scaffold-33	1952773	A	G
Scaffold-33	2016302	G	A
Scaffold-33	2016310	T	C
Scaffold-33	2016312	TT	GT
Scaffold-33	2016319	A	G
Scaffold-33	2016362	CTGTTCCA	CTGTACCA,TTGTACCT
Scaffold-33	2016371	A	C
Scaffold-33	2016392	G	A,T
Scaffold-33	2016393	TGTATCTAC	TGTATTTAC,TGTATCTAGTATCTAC
Scaffold-33	2016402	CTG	CTA,GTA,TTA,CTT
Scaffold-33	2016430	T	A
Scaffold-33	5816192	A	T
Scaffold-33	5816211	A	G

Scaffold-33	5816262	C	T
Scaffold-33	6143955	A	T
Scaffold-33	7412010	C	A
Scaffold-33	7412030	T	C
Scaffold-33	7412067	C	T
Scaffold-33	9399488	TCTCC	TG
Scaffold-33	9399544	TA	TTTCACA,TT
Scaffold-33	9399546	ACAA	ACA
Scaffold-33	9399569	C	T
Scaffold-33	9399575	T	C
Scaffold-33	9399582	TAAAAAAAT	TAAAAAAAAT,TAAAAAAAC
Scaffold-33	15306934	C	G
Scaffold-33	15306964	C	T,G
Scaffold-33	15306965	T	C
Scaffold-33	15307029	TGG	TGA,TG
Scaffold-33	19763415	C	A
Scaffold-33	19763417	G	A
Scaffold-33	19763433	T	C,G
Scaffold-33	19763437	AAC	AAA,AGA
Scaffold-33	19763486	TT	CT,CG
Scaffold-33	19763553	CG	TG
Scaffold-33	26578202	GAA	GA
Scaffold-33	26578219	G	A
Scaffold-33	26578267	T	A
Scaffold-33	26578272	C	T
Scaffold-33	32731195	G	A
Scaffold-33	33367214	C	T
Scaffold-33	33367221	G	T
Scaffold-33	33367225	TAA	TA,TAG
Scaffold-33	33367263	GACTACTA	GACTA,GAATACTA
Scaffold-33	33367281	G	A
Scaffold-33	38024452	T	G
Scaffold-33	38024500	CAT	CAC
Scaffold-33	38024512	G	A
Scaffold-33	38024557	A	C
Scaffold-33	39806818	ATTTTTTTTA	ATTTTTTTA
Scaffold-33	39806863	A	G
Scaffold-33	42206639	T	A
Scaffold-33	42206664	G	T
Scaffold-33	43289072	C	A
Scaffold-33	43289079	G	A
Scaffold-33	43289086	T	A
Scaffold-33	43289087	C	G
Scaffold-33	43289095	G	A

Scaffold-33	43289125	C	T
Scaffold-33	43289140	T	G
Scaffold-33	43289146	GTCAG	ATCAT,GCCAG,GTTAG
Scaffold-33	46708292	G	T
Scaffold-33	46708302	G	C
Scaffold-33	46708320	AACA	AA
Scaffold-33	46708327	TCTTCTC	GTTTGTC
Scaffold-33	46708338	C	T
Scaffold-33	46708355	C	G
Scaffold-33	47208045	C	T,G
Scaffold-33	47208047	C	T
Scaffold-33	47208080	G	C
Scaffold-33	47208102	T	C
Scaffold-33	47208115	C	A
Scaffold-33	47208149	C	T
Scaffold-33	49295462	GCCC	TCCC
Scaffold-33	49295483	TTC	TTT
Scaffold-33	49295487	AGG	AG
Scaffold-33	49295490	C	A
Scaffold-33	49295498	T	C
Scaffold-33	49295499	CAG	AAG
Scaffold-33	49295506	CCAATC	CCAATG
Scaffold-33	49295512	GTC	GTT
Scaffold-33	49295518	A	G
Scaffold-33	49295521	T	A
Scaffold-33	49295558	T	A
Scaffold-33	49295559	G	A
Scaffold-33	49295562	G	C
Scaffold-33	49295567	A	G
Scaffold-33	49295591	C	T
Scaffold-34	1087506	A	C
Scaffold-34	1087536	C	T
Scaffold-34	1087568	A	T
Scaffold-34	1087575	CTTTTAT	CT,CTTTTCT
Scaffold-34	1087625	A	C
Scaffold-34	1087626	T	C
Scaffold-34	3480108	A	C
Scaffold-34	3480182	G	A
Scaffold-34	13339119	T	G
Scaffold-34	13339143	A	G
Scaffold-34	13339144	G	A
Scaffold-34	13339148	G	A
Scaffold-34	13339162	GTTTT	ATTTA
Scaffold-34	14374937	A	C

Scaffold-34	14374943	A	T
Scaffold-34	14374955	T	C
Scaffold-34	14374976	C	T
Scaffold-34	14374977	G	A
Scaffold-34	14375015	G	A
Scaffold-34	14375022	A	G
Scaffold-34	15108189	T	C
Scaffold-34	15108280	C	T
Scaffold-34	15108297	T	G
Scaffold-34	16218681	A	G
Scaffold-34	16218682	G	A
Scaffold-34	16218683	TCG	CCG
Scaffold-34	16218692	GG	AG
Scaffold-34	16218706	C	T
Scaffold-34	16218711	C	T
Scaffold-34	16218712	GTC	ATT
Scaffold-34	16218724	G	T
Scaffold-34	16218727	CAG	TAT
Scaffold-34	16218730	G	A
Scaffold-34	16218738	AGACA	CGACA,CGACT,GGACA
Scaffold-34	16218744	TTGGCCC	CTGGGCC,TTGGGCC,CTGGCCC,TTGGTAC,CTGGACC,CTGGTTT
Scaffold-34	16218753	C	T
Scaffold-34	16218755	T	C
Scaffold-34	16218767	A	G
Scaffold-34	16218768	G	T
Scaffold-34	16218794	TAGAT	GAGAT,AAGAT
Scaffold-34	16218818	A	T
Scaffold-34	17560805	TAA	TA
Scaffold-34	17560856	G	A
Scaffold-34	17560870	A	T
Scaffold-34	18244429	AA	AATA
Scaffold-34	18244469	T	A
Scaffold-34	18244477	G	C
Scaffold-34	19231202	ATT	AT
Scaffold-34	19231208	C	G
Scaffold-34	19231234	C	T
Scaffold-34	19231251	T	C
Scaffold-34	19231261	A	C
Scaffold-34	20562348	T	C
Scaffold-34	20562383	T	A
Scaffold-34	20562391	T	C
Scaffold-34	21352243	A	G
Scaffold-34	21352252	A	G

Scaffold-34	21352282	A	C
Scaffold-34	21352322	C	G
Scaffold-34	21352332	A	G
Scaffold-34	22602690	A	G
Scaffold-34	22602726	G	A
Scaffold-34	22602729	TAAAAAAAAT	TAAAAAAACT,TCAAAAAAAT
Scaffold-34	22602776	T	C
Scaffold-34	23087399	C	A
Scaffold-34	23813073	C	T
Scaffold-34	23813132	GTA	GTC
Scaffold-34	23813170	G	A
Scaffold-34	23813175	G	T
Scaffold-34	24872520	A	G
Scaffold-34	24872537	C	T
Scaffold-34	24872550	GCACAACAA	GCACAACCACAA,GCACAACAACAA
Scaffold-34	24872560	TCCAAAAAAAAAAAAC	TCCAAGAAAAAAAC,TCCAAGGAAAAAAAC,TCCAAGAAAAAAAAC,TCCAAAAAAAAAAAC
Scaffold-34	24872578	A	G
Scaffold-34	24872579	G	C
Scaffold-34	24872585	G	C
Scaffold-34	24872616	TG	GG
Scaffold-34	24872631	T	G
Scaffold-34	24872655	C	A
Scaffold-34	30294276	A	G
Scaffold-34	30294310	GGCTTTAATCCT	GGTTTTAATCCT,GT
Scaffold-34	30294325	T	A
Scaffold-34	31845059	C	T
Scaffold-34	31845085	A	G
Scaffold-34	32907515	T	C
Scaffold-34	32907539	C	T
Scaffold-34	32907552	G	A
Scaffold-34	32907576	G	A
Scaffold-34	33252484	C	T
Scaffold-34	33252487	C	G
Scaffold-34	33252595	C	T
Scaffold-34	33948778	TAAG	TAG,TGG
Scaffold-34	33948786	G	T
Scaffold-34	33948795	GA	GTA
Scaffold-34	35896490	T	A
Scaffold-34	35896508	TTT	TTA,ATA
Scaffold-34	35896558	C	T
Scaffold-34	41507978	A	G
Scaffold-34	41508033	G	A

Scaffold-34	41508048	T	C
Scaffold-34	41508057	G	T
Scaffold-34	41508069	A	G
Scaffold-34	41508077	A	C
Scaffold-34	43424507	G	C
Scaffold-34	43424572	A	G
Scaffold-34	43424596	G	T
Scaffold-34	43424668	C	A
Scaffold-34	43424673	G	A
Scaffold-34	43424756	C	T
Scaffold-34	43424758	T	A
Scaffold-34	45443923	G	T
Scaffold-34	45443931	G	T
Scaffold-34	46280441	C	T
Scaffold-34	46280468	T	A
Scaffold-34	46280498	G	T
Scaffold-34	46280503	GG	GC
Scaffold-34	46280511	G	A
Scaffold-34	46280540	GGGTGGTG	AGGTGGTG,GGGTG
Scaffold-34	46280551	G	A
Scaffold-34	46280554	G	A
Scaffold-34	46280564	C	G
Scaffold-34	49068176	T	G
Scaffold-34	49068201	G	C
Scaffold-34	49068221	T	C
Scaffold-34	49597766	C	T
Scaffold-34	49597793	C	T
Scaffold-34	49597808	T	C
Scaffold-35	244572	CTTGGTGG	GTTTGTGG,GTTTGTGC,CTTGTTGG
Scaffold-35	244586	CCG	TCG,TCA
Scaffold-35	244611	C	G
Scaffold-35	2348563	G	A
Scaffold-35	2348629	A	G
Scaffold-35	4943310	A	G
Scaffold-35	4943311	C	T
Scaffold-35	4943313	G	A
Scaffold-35	4943322	C	T
Scaffold-35	4943345	C	T
Scaffold-35	4943351	T	C
Scaffold-35	4943364	G	A
Scaffold-35	4943365	A	G
Scaffold-35	4943392	A	G
Scaffold-35	6644298	C	T
Scaffold-35	6644317	G	T

Scaffold-35	6644330	T	G
Scaffold-35	6644344	T	A
Scaffold-35	6644351	CCTC	CCTG
Scaffold-35	6644366	G	C
Scaffold-35	7277415	A	G
Scaffold-35	7277428	T	C
Scaffold-35	7277455	C	T
Scaffold-35	7277465	G	C
Scaffold-35	7277482	C	T
Scaffold-35	13386476	TTCA	TTCG,GTCG
Scaffold-35	13386492	G	C
Scaffold-35	13386521	A	G
Scaffold-35	13386551	A	G
Scaffold-35	13386575	A	C
Scaffold-35	13386590	G	A
Scaffold-35	13386608	T	C
Scaffold-35	14003426	A	T
Scaffold-35	14003506	T	G
Scaffold-35	14003509	C	T
Scaffold-35	15533323	C	A
Scaffold-35	15533394	A	T
Scaffold-35	18294182	CTTTTTTTTA	CTTTTTTTTTA
Scaffold-35	18294226	C	T
Scaffold-35	18294227	G	A
Scaffold-35	18685849	T	G
Scaffold-35	18685897	C	T
Scaffold-35	18685909	C	T
Scaffold-35	22070799	G	A,C
Scaffold-35	22070830	A	G
Scaffold-35	22070880	G	C
Scaffold-35	22070882	T	C
Scaffold-35	22070905	G	A
Scaffold-35	22070910	G	A
Scaffold-35	22070913	A	G,C
Scaffold-35	22070914	C	T
Scaffold-35	22070917	T	G
Scaffold-35	24231126	C	T
Scaffold-35	24231153	T	C
Scaffold-35	24231155	TTTT	CTTC,CTTT
Scaffold-35	24231167	TT	TGT
Scaffold-35	24231191	CAAAAAT	CAAAATT,CAATT
Scaffold-35	24231215	C	T
Scaffold-35	24231220	A	T
Scaffold-35	24231230	AAGG	AAGAGG

Scaffold-35	24512718	G	A
Scaffold-35	24512781	A	T
Scaffold-35	24512825	A	T
Scaffold-35	27491510	T	C
Scaffold-35	27491543	A	C
Scaffold-35	27491559	C	T
Scaffold-35	27491560	GTT	GT
Scaffold-35	27491625	A	G
Scaffold-35	28279513	G	A
Scaffold-35	29332362	C	T
Scaffold-35	29332400	A	T
Scaffold-35	29332412	C	T
Scaffold-35	29332422	C	T
Scaffold-35	29332434	G	A
Scaffold-35	29332456	T	C
Scaffold-35	29332458	T	G
Scaffold-35	29332478	TTGTT	CTGTC
Scaffold-35	29332486	C	T
Scaffold-35	29332487	G	A
Scaffold-35	29332504	A	G
Scaffold-35	29332505	G	T
Scaffold-35	29332506	A	T
Scaffold-35	33268539	C	T
Scaffold-35	33268563	C	T
Scaffold-35	33268600	A	T
Scaffold-35	33268624	G	A
Scaffold-35	33268656	G	T
Scaffold-35	33268660	C	T
Scaffold-35	35559962	A	C
Scaffold-35	35560011	CAA	CA
Scaffold-35	36009577	ATT	GTC
Scaffold-35	41668454	C	T
Scaffold-35	44638434	A	T
Scaffold-35	44638441	T	A,G
Scaffold-35	44638465	TATAGCTTCTTGAAGGAAAATAAATGTCTAGGCGA	TATAGCTCCTTGAAGGAAAATAAATGTCTAGGCGA
Scaffold-35	46493817	CAGG	GAGT
Scaffold-35	46493851	A	G
Scaffold-35	46493852	G	C
Scaffold-35	46493872	G	A
Scaffold-35	46493873	A	G
Scaffold-35	48014760	C	A
Scaffold-35	48014785	C	T
Scaffold-35	48014786	GACG	GACA,AACG

Scaffold-35	48014790	TATAGTAACA	TATA,TATAGTTACA
Scaffold-35	48014811	ATGAG	ATGAC
Scaffold-35	48014836	A	T
Scaffold-35	48014892	T	C
Scaffold-35	48014901	T	C
Scaffold-35	48014918	A	G
Scaffold-35	48014972	G	A
Scaffold-35	48015017	T	G
Scaffold-35	48015020	C	T
Scaffold-35	48015029	T	A
Scaffold-36	998560	T	C
Scaffold-36	998614	T	C
Scaffold-36	3746526	G	A
Scaffold-36	3746527	AATC	GATC
Scaffold-36	3746548	G	A
Scaffold-36	3746551	G	A
Scaffold-36	3746558	G	A
Scaffold-36	3746567	GGG	GGA
Scaffold-36	3746574	C	A,T
Scaffold-36	3746594	A	G
Scaffold-36	4248787	G	A
Scaffold-36	4248794	G	A
Scaffold-36	5264075	C	A
Scaffold-36	5264085	C	T
Scaffold-36	5264113	C	T
Scaffold-36	5264128	TTA	TTG
Scaffold-36	7251283	G	A
Scaffold-36	7251295	A	C
Scaffold-36	9034987	A	T
Scaffold-36	9035021	G	A
Scaffold-36	9035039	C	T
Scaffold-36	9035046	T	C
Scaffold-36	10981876	A	T
Scaffold-36	10981909	C	G
Scaffold-36	11767255	C	T
Scaffold-36	11767341	T	G
Scaffold-36	11767377	A	G
Scaffold-36	12040726	TGGGGGT	TGGAGGT,TGGGGT
Scaffold-36	12040746	C	T
Scaffold-36	12040747	CCTTCTTC	CCCTCTTC,TCTTCTTC
Scaffold-36	12040804	A	T
Scaffold-36	12040811	ATC	AC
Scaffold-36	12043468	AACC	AACT,CACT
Scaffold-36	12043499	G	A

Scaffold-36	12043511	G	T
Scaffold-36	13524876	CAAAAAAG	CAAAAGAG
Scaffold-36	13524905	T	A
Scaffold-36	13524918	T	C
Scaffold-36	13537208	C	G
Scaffold-36	13537270	G	A
Scaffold-36	13582459	C	T
Scaffold-36	13582464	A	G
Scaffold-36	13582507	A	C
Scaffold-36	13582520	C	T
Scaffold-36	13582560	T	C
Scaffold-36	13582584	G	A
Scaffold-36	16319152	G	A
Scaffold-36	16319156	A	C
Scaffold-36	16319208	C	A
Scaffold-36	16319217	A	G
Scaffold-36	16319236	T	A
Scaffold-36	17182577	G	A
Scaffold-36	17182620	C	A
Scaffold-36	17182639	A	G
Scaffold-36	17182646	G	T
Scaffold-36	17182651	T	C
Scaffold-36	17182676	C	T
Scaffold-36	19644943	G	A
Scaffold-36	19645009	A	G
Scaffold-36	23730366	G	A
Scaffold-36	23730367	C	A
Scaffold-36	23730374	GGATT	GGGTC
Scaffold-36	23730402	T	G
Scaffold-36	23730403	C	T
Scaffold-36	23730404	C	T
Scaffold-36	23730405	T	G
Scaffold-36	23730408	C	G
Scaffold-36	23730415	A	T
Scaffold-36	23730416	G	A
Scaffold-36	23730422	A	T,G
Scaffold-36	23730432	T	A
Scaffold-36	23730439	C	A
Scaffold-36	23730446	T	G
Scaffold-36	23730454	G	A
Scaffold-36	23730464	T	C
Scaffold-36	23730468	C	A
Scaffold-36	23730469	GT	AG
Scaffold-36	23730471	A	G

Scaffold-36	23730478	C	A
Scaffold-36	23921578	A	T
Scaffold-36	23921634	T	C
Scaffold-36	23921700	C	T
Scaffold-36	23921709	A	T
Scaffold-36	25259289	C	T
Scaffold-36	25259312	C	T
Scaffold-36	25971517	A	G
Scaffold-36	30728576	T	C
Scaffold-36	30728580	A	T
Scaffold-36	30728595	A	C
Scaffold-36	30728600	C	A
Scaffold-36	30728601	A	C
Scaffold-36	30728622	A	G
Scaffold-36	30728624	AAAATTATTACCCTTCGAGA	AAAATTATTATCCTTCGAGA,AA
Scaffold-36	30728658	G	A,C
Scaffold-36	30728678	A	T,C
Scaffold-36	30728681	T	A
Scaffold-36	30728685	T	A
Scaffold-36	32814282	C	G
Scaffold-36	32814296	C	T
Scaffold-36	32814314	A	G
Scaffold-36	32814331	A	G
Scaffold-36	32814332	A	T
Scaffold-36	33311016	G	A
Scaffold-36	33311053	G	A
Scaffold-36	33311062	CA	CG,TG
Scaffold-36	33311080	C	A
Scaffold-36	33311087	T	C
Scaffold-36	33311091	C	T
Scaffold-36	33311124	C	G
Scaffold-36	33311142	T	C
Scaffold-36	34852287	A	G
Scaffold-36	34852321	G	T
Scaffold-36	34852342	G	A
Scaffold-36	34852354	A	G
Scaffold-36	34852369	A	T
Scaffold-36	34852380	A	G
Scaffold-36	34852383	G	C
Scaffold-36	36376934	C	T
Scaffold-36	40674070	C	T
Scaffold-36	40674102	G	A
Scaffold-36	40674103	A	G

Scaffold-36	40674125	C	T
Scaffold-36	40674131	A	G
Scaffold-36	40674146	A	T
Scaffold-36	40674167	A	G
Scaffold-36	40674178	A	G
Scaffold-36	41573316	ACT	ACC
Scaffold-36	47567849	GAGAC	AAGAC
Scaffold-36	47567861	A	G
Scaffold-36	47567889	G	C
Scaffold-36	47567900	A	G
Scaffold-37	3144822	G	T,A
Scaffold-37	3144830	G	A
Scaffold-37	3144852	CCA	TCA,TCG
Scaffold-37	3144861	G	A
Scaffold-37	3144891	A	G
Scaffold-37	8364926	AATATATATATATATATATATA	AATATATATATA,AATATATATA,AATATATATATATA
Scaffold-37	8364952	A	G
Scaffold-37	10039593	C	T
Scaffold-37	10039649	CAGG	CAGC
Scaffold-37	10039668	G	A
Scaffold-37	10039679	G	A
Scaffold-37	10039700	C	T
Scaffold-37	10039706	A	C
Scaffold-37	21844152	ATA	GTG
Scaffold-37	21844189	A	T
Scaffold-37	23444077	G	A
Scaffold-37	23444095	A	G
Scaffold-37	23444100	T	C
Scaffold-37	23444101	C	T
Scaffold-37	23444115	TGGATAGCAGATTTTGG	TGGAATGCAGATTTTGG,TG 600
Scaffold-37	23444133	C	T
Scaffold-37	23444169	G	A
Scaffold-37	23444201	C	T
Scaffold-37	23479067	C	A
Scaffold-37	23479148	C	T
Scaffold-37	23479164	T	C
Scaffold-37	23479168	C	T
Scaffold-37	23674602	C	T
Scaffold-37	23674617	G	A
Scaffold-37	23674631	T	C
Scaffold-37	23674638	C	T
Scaffold-37	23674641	C	T

Scaffold-37	23674659	C	T	
Scaffold-37	23674677	A	G	
Scaffold-37	24675651	G	A	
Scaffold-37	24675670	C	T	
Scaffold-37	24675679	G	A	
Scaffold-37	24675683	G	T	
Scaffold-37	24675725	T	C	
Scaffold-37	26696580	A	G	
Scaffold-37	26696593	G	A	
Scaffold-37	26696620	G	A	
Scaffold-37	26696634	G	A	
Scaffold-37	26977851	A	T	
Scaffold-37	26977870	A	G	
Scaffold-37	26977908	G	A	
Scaffold-37	26977909	A	G	
Scaffold-37	26977927	G	A	
Scaffold-37	26977936	G	A	
Scaffold-37	26977945	C	T	
Scaffold-37	26977947	A	C	
Scaffold-37	26977954	C	A	
Scaffold-37	26977955	CCG	TCG,TCA	
Scaffold-37	26977973	G	A	
Scaffold-37	26977974	T	C	
Scaffold-37	26977981	C	T	
Scaffold-37	27225756	CCGTC		CCGTT
Scaffold-37	27225761	G	A	
Scaffold-37	27225766	G	A	
Scaffold-37	27225771	C	T	
Scaffold-37	27225777	T	A	
Scaffold-37	27225779	G	A	
Scaffold-37	27225786	G	A	
Scaffold-37	27225792	C	G	
Scaffold-37	27225793	G	A	
Scaffold-37	27225794	A	C	
Scaffold-37	27225822	G	C	
Scaffold-37	27225833	GGAT	AGAA	
Scaffold-37	27225840	C	T	
Scaffold-37	27225841	GG	AG	
Scaffold-37	27225847	C	T	
Scaffold-37	27225848	G	A	
Scaffold-37	27225860	C	T	
Scaffold-37	30371755	G	A	
Scaffold-37	30371815	C	T	
Scaffold-37	30371824	C	A	

Scaffold-37	30371840	A	T
Scaffold-37	37275256	G	T
Scaffold-37	37275295	A	G
Scaffold-37	37275349	C	T
Scaffold-37	40799283	T	A
Scaffold-37	40799332	A	G
Scaffold-37	42001398	T	G
Scaffold-37	42001426	C	A
Scaffold-37	42001441	A	T
Scaffold-37	42001442	T	A
Scaffold-37	42001454	T	C
Scaffold-37	42001485	T	C
Scaffold-37	42803313	T	A
Scaffold-37	42803321	A	T
Scaffold-37	42803322	G	T
Scaffold-37	42803361	ATTTCT	ATTTTG,TTTTTG,ATTCCT
Scaffold-37	42803376	A	C
Scaffold-37	42803388	G	T
Scaffold-37	42803403	G	A
Scaffold-37	42803405	G	A
Scaffold-37	42803417	AA	AT,AC
Scaffold-37	42803419	AGGTT	AAGTA,AGATT
Scaffold-37	42803426	C	T
Scaffold-37	42803427	G	A
Scaffold-37	44790124	T	A
Scaffold-37	44790137	G	T
Scaffold-37	44790148	CTCA	CTCC,TTCA
Scaffold-37	44790154	GGAA	GGAT,GCAT
Scaffold-37	44790205	GCTCTTA	GCTCCTG,GCTCCTA
Scaffold-37	44790218	T	A
Scaffold-37	45960369	C	A
Scaffold-37	45960374	T	C
Scaffold-37	45960393	T	G
Scaffold-37	45960408	A	C
Scaffold-37	45960432	G	A
Scaffold-37	45960457	AA	ATA
Scaffold-37	45960472	GTCTTCTCTA	ATCTTCTCTA,ATCA
Scaffold-37	45960488	C	T
Scaffold-38	2687389	T	C
Scaffold-38	7421186	TGCTGAG	AGCAGAA
Scaffold-38	7421216	CCTTT	ACTTC
Scaffold-38	7421228	A	C
Scaffold-38	7421240	CCCTGGA	ACCCGGG
Scaffold-38	7421355	A	G

Scaffold-38	7421359	C	T
Scaffold-38	7421361	A	G
Scaffold-38	7421365	G	C
Scaffold-38	7421400	A	G,T
Scaffold-38	7421419	AATA	AATT,GATT
Scaffold-38	7421423	A	T
Scaffold-38	7421459	G	A
Scaffold-38	10444972	A	G
Scaffold-38	10444974	T	C
Scaffold-38	10444986	C	G
Scaffold-38	10445040	A	C
Scaffold-38	11551985	A	G
Scaffold-38	11552007	A	G
Scaffold-38	11552020	C	G
Scaffold-38	12880397	C	T
Scaffold-38	12880398	C	T
Scaffold-38	12880400	G	A
Scaffold-38	12880403	G	T
Scaffold-38	12880404	A	G
Scaffold-38	12880412	G	A
Scaffold-38	12880419	C	T
Scaffold-38	12880422	CAT	CAC
Scaffold-38	12880436	A	G
Scaffold-38	12880437	G	C
Scaffold-38	12880445	AA	AC,GC
Scaffold-38	12880452	CTGG	TTGA,TTGG
Scaffold-38	12880458	C	A
Scaffold-38	12880461	C	T
Scaffold-38	12880470	C	T
Scaffold-38	12880487	G	A
Scaffold-38	12880489	A	G
Scaffold-38	12880494	CGA	CGG,CTA,TGG
Scaffold-38	12880497	AGGTT	AGGTA,AGGTC
Scaffold-38	12880509	C	T
Scaffold-38	15080785	A	T
Scaffold-38	15330213	A	G
Scaffold-38	15330224	T	G
Scaffold-38	15330230	T	C
Scaffold-38	15330254	A	C
Scaffold-38	15330271	A	T
Scaffold-38	15330302	G	T
Scaffold-38	15330310	C	T
Scaffold-38	15330317	A	G
Scaffold-38	15960406	T	C

Scaffold-38	15960436	G	A
Scaffold-38	15960441	G	A
Scaffold-38	15960457	AG	AA
Scaffold-38	15960467	T	G
Scaffold-38	15960511	G	A
Scaffold-38	17811041	CT	CC
Scaffold-38	17811070	GC	GT
Scaffold-38	17811096	ACGGT	ACGGG
Scaffold-38	17811119	A	G
Scaffold-38	17811120	G	A
Scaffold-38	17811124	G	A
Scaffold-38	17811164	G	C
Scaffold-38	20463057	CGACTTCC	CGACCTAC
Scaffold-38	20463069	A	G
Scaffold-38	20463108	GTGTGGA	GTGTGGT
Scaffold-38	20463159	C	T
Scaffold-38	24740223	ACT	CCT
Scaffold-38	25477424	C	T
Scaffold-38	25477503	T	C
Scaffold-38	25477509	G	A
Scaffold-38	25477510	A	C
Scaffold-38	28563019	G	A
Scaffold-38	28563025	G	A
Scaffold-38	28563047	A	G
Scaffold-38	28563068	G	A
Scaffold-38	28563111	C	T
Scaffold-38	28563135	C	T
Scaffold-38	31260002	C	T
Scaffold-38	31260021	GAAAA	GACAA
Scaffold-38	31260033	CTA	CTG
Scaffold-38	31260059	A	G
Scaffold-38	31260078	GGCC	GGCT
Scaffold-38	31260082	TG	TA
Scaffold-38	32821896	CCTA	CCTG
Scaffold-38	32821900	AC	AA
Scaffold-38	32821913	GGTTG	GGTTT,GATTT
Scaffold-38	32821933	T	G
Scaffold-38	32821948	G	A
Scaffold-38	38607305	G	A
Scaffold-38	38607347	A	G
Scaffold-38	38607387	C	T
Scaffold-38	38607391	T	C
Scaffold-38	38607406	T	G
Scaffold-38	38607410	G	A

Scaffold-38	42907111	A	G	
Scaffold-38	43126078	A	G	
Scaffold-38	43126093	CCTG	CG	
Scaffold-38	43126100	T	C	
Scaffold-38	43126131	C	T	
Scaffold-38	46160769	C	G	
Scaffold-38	46160774	T	A	
Scaffold-38	48732138	C	G	
Scaffold-38	48835054	C	G	
Scaffold-38	48835090	G	A	
Scaffold-38	48835096	T	G	
Scaffold-39	1742181	C	T	
Scaffold-39	1742191	C	T	
Scaffold-39	1742192	G	A	
Scaffold-39	1742201	C	G	
Scaffold-39	1742217	C	T	
Scaffold-39	1742218	G	C	
Scaffold-39	1742225	T	C	
Scaffold-39	1742236	G	T	
Scaffold-39	5152950	ACTT	GCTC	
Scaffold-39	5152988	A	G	
Scaffold-39	5152993	A	G	
Scaffold-39	5152999	A	G	
Scaffold-39	5153005	G	A	
Scaffold-39	5153028	A	G	
Scaffold-39	5153029	T	G	
Scaffold-39	5153047	A	G	
Scaffold-39	5153052	A	C	
Scaffold-39	10605074	A	G	
Scaffold-39	10605099	C	T	
Scaffold-39	10605107	A	G	
Scaffold-39	10605118	T	C	
Scaffold-39	10605127	A	C	
Scaffold-39	10605136	ATAA	GTAG,ATAG	
Scaffold-39	10605152	A	G	
Scaffold-39	10605165	T	C	
Scaffold-39	10605166	A	G	
Scaffold-39	10605185	T	C	
Scaffold-39	10605189	A	T	
Scaffold-39	10605195	CC	TA	
Scaffold-39	10605198	A	G	
Scaffold-39	11746853	TGTACG		GGTATG
Scaffold-39	11746863	CAGAC		CAAAA,CTGAC
Scaffold-39	11746875	C	G	

Scaffold-39	11746878	C	T	
Scaffold-39	11746884	C	A	
Scaffold-39	11746891	CCAAC		TCAAC
Scaffold-39	11746896	GG	GA	
Scaffold-39	11746903	ACCTTC		GCATTA,GCCTTC
Scaffold-39	11746913	A	G	
Scaffold-39	11746929	CTA	TTA	
Scaffold-39	11746932	T	A	
Scaffold-39	11746937	ATTTG		GTTTA
Scaffold-39	11746946	C	T	
Scaffold-39	11746959	C	A	
Scaffold-39	11746961	A	T	
Scaffold-39	11746965	C	T	
Scaffold-39	11746966	C	G	
Scaffold-39	16735346	G	A	
Scaffold-39	16735379	AG	GA,TA	
Scaffold-39	16735391	C	T	
Scaffold-39	16735437	T	G	
Scaffold-39	16748305	T	C	
Scaffold-39	16748310	T	G,C	
Scaffold-39	16748330	C	A	
Scaffold-39	16748353	GTA	GA	
Scaffold-39	17517701	T	A	
Scaffold-39	17517793	AA	TG	
Scaffold-39	19529835	C	T	
Scaffold-39	20036674	C	T	
Scaffold-39	20036676	C	T	
Scaffold-39	20036731	A	T	
Scaffold-39	20036736	C	G	
Scaffold-39	20036745	TT	GT,GA	
Scaffold-39	23315593	G	C	
Scaffold-39	23315616	G	C	
Scaffold-39	24408252	G	A	
Scaffold-39	24408261	C	A	
Scaffold-39	27007856	G	T	
Scaffold-39	27007873	G	A	
Scaffold-39	27007913	A	C	
Scaffold-39	27007915	A	T	
Scaffold-39	27007948	T	C	
Scaffold-39	28768590	C	A	
Scaffold-39	28768692	T	G	
Scaffold-39	36617303	T	C	
Scaffold-39	36617357	T	A	
Scaffold-39	36617367	A	G	

Scaffold-39	36617375	A	T
Scaffold-39	36617385	T	C
Scaffold-39	36873487	C	A,T
Scaffold-39	36873542	C	T
Scaffold-39	36873579	G	T
Scaffold-39	36873585	T	C
Scaffold-39	36873594	A	T
Scaffold-39	36923104	A	T
Scaffold-39	36940307	G	A
Scaffold-39	36940319	T	C
Scaffold-39	36940325	C	T
Scaffold-39	36940413	A	C
Scaffold-39	36987402	C	A
Scaffold-39	37222598	GATATATATATT	GATATATATACT
Scaffold-39	37222613	A	C
Scaffold-39	37222614	G	T
Scaffold-39	37222615	G	A
Scaffold-39	37222627	G	A
Scaffold-39	37222665	G	A
Scaffold-39	37323185	G	A
Scaffold-39	37450469	C	T
Scaffold-39	37450498	A	G
Scaffold-39	37457300	T	G
Scaffold-39	37457303	C	T
Scaffold-39	37536808	T	C
Scaffold-39	37536816	C	A
Scaffold-39	37536826	GATTC	AATTC,AATTA
Scaffold-39	37536841	C	T
Scaffold-39	37536852	G	A
Scaffold-39	37536859	G	A
Scaffold-39	37536919	CGAG	TGAG,TGAC
Scaffold-39	37536938	C	T
Scaffold-39	37618980	G	A
Scaffold-39	37619001	T	C
Scaffold-39	37619021	T	C
Scaffold-39	37619028	A	C
Scaffold-39	37619035	G	A
Scaffold-39	37619041	C	T
Scaffold-39	37619043	A	G
Scaffold-39	37619064	A	C
Scaffold-39	37619098	C	T
Scaffold-39	37619105	A	G
Scaffold-39	37619112	G	A
Scaffold-39	37858605	T	C

Scaffold-39	37858632	A	T
Scaffold-39	37858692	A	G
Scaffold-39	37940522	A	G
Scaffold-39	37940532	G	C
Scaffold-39	37940550	A	T
Scaffold-39	37940551	G	C
Scaffold-39	37940568	G	A
Scaffold-39	38296077	C	T
Scaffold-39	38296121	G	A
Scaffold-39	38563356	G	A
Scaffold-39	38563382	A	T
Scaffold-39	38563389	G	A
Scaffold-39	38563419	A	G
Scaffold-39	38563456	G	T
Scaffold-39	38563465	T	C
Scaffold-39	38563482	G	A
Scaffold-39	38743125	A	G
Scaffold-39	38743136	C	T
Scaffold-39	38743137	G	A
Scaffold-39	38743153	C	T
Scaffold-39	38743154	G	T
Scaffold-39	38743169	AT	AC
Scaffold-39	38743209	C	A
Scaffold-39	38743226	A	C
Scaffold-39	38743243	CAA	CAAA
Scaffold-39	39045719	G	C
Scaffold-39	39045735	G	A
Scaffold-39	39045736	G	A
Scaffold-39	39045742	A	G
Scaffold-39	39045757	T	C
Scaffold-39	39045762	A	G
Scaffold-39	39045775	G	T
Scaffold-39	39045781	A	C
Scaffold-39	39045783	G	A
Scaffold-39	39045835	T	A
Scaffold-39	39045851	T	C
Scaffold-39	39115426	G	A
Scaffold-39	39115437	T	A
Scaffold-39	39115507	G	A
Scaffold-39	39390186	C	A
Scaffold-39	39390213	G	A
Scaffold-39	39390237	G	A
Scaffold-39	39390240	C	T
Scaffold-39	39390252	C	T

Scaffold-39	39390253	G	A
Scaffold-39	39390269	A	G
Scaffold-39	39390317	C	T
Scaffold-39	39448804	G	T
Scaffold-39	39448850	G	A
Scaffold-39	39448922	A	G
Scaffold-39	39657843	CAAA	CAAAAA
Scaffold-39	39657863	G	A
Scaffold-39	39657874	A	G
Scaffold-39	39657896	C	G
Scaffold-39	39657902	C	T
Scaffold-39	39657906	A	T
Scaffold-39	39657919	GTT	GTC,GT
Scaffold-39	39657924	GG	GGAAATACTG
Scaffold-39	39657930	A	G
Scaffold-39	39719790	C	T
Scaffold-39	40107925	TACACACACACG	AACACACACACG
Scaffold-39	40110915	CTCTG	CTCTA,TTCTA
Scaffold-39	40110951	A	C
Scaffold-39	40110967	T	G
Scaffold-39	40184136	T	C
Scaffold-39	40184165	G	A
Scaffold-39	40184174	C	A
Scaffold-39	40184202	GCC	GC
Scaffold-39	40184215	T	C
Scaffold-39	40184232	T	G
Scaffold-39	40184237	TATTTTTGTCA	TCTTTTTGTCA
Scaffold-39	40201074	T	C
Scaffold-39	40201080	G	T
Scaffold-39	40201085	G	A
Scaffold-39	40201152	G	A
Scaffold-39	40283377	AGTG	AG
Scaffold-39	40283421	G	C
Scaffold-39	40540118	A	T
Scaffold-39	40540131	CG	CGTATAACTG
Scaffold-39	40540140	ACCCCCCT	ACCTCCCT,ACCCCCCCT
Scaffold-39	40540149	C	T
Scaffold-39	40540156	G	T
Scaffold-39	40540159	T	C
Scaffold-39	40540185	A	C
Scaffold-39	40556901	T	G
Scaffold-39	40974839	T	A
Scaffold-39	40974849	AGA	AAG
Scaffold-39	40974877	A	G

Scaffold-39	40974881	GTCA	ATA
Scaffold-39	40974891	T	A
Scaffold-39	40974925	T	A
Scaffold-39	41033357	GTTTTTC	GTTTTC,GTTTGTC
Scaffold-39	41033412	A	T
Scaffold-39	41033436	C	T
Scaffold-39	41033453	TAC	TAAC
Scaffold-39	41668370	C	T
Scaffold-39	41668376	GTT	GT,ATT
Scaffold-39	41668420	G	T
Scaffold-39	41668430	A	G
Scaffold-39	41668442	C	A
Scaffold-39	41913676	CTCAC	CTCAT
Scaffold-39	41913792	C	T
Scaffold-39	41983768	T	C
Scaffold-39	41983802	A	C
Scaffold-39	41983806	T	A
Scaffold-39	41983814	A	G
Scaffold-39	42007723	G	A
Scaffold-39	42007744	C	T
Scaffold-39	42007745	G	A
Scaffold-39	42007754	GTG	GTTG
Scaffold-39	42007759	T	C
Scaffold-39	42007778	T	C
Scaffold-39	42416174	GAA	GA,TAA
Scaffold-39	42416184	T	A
Scaffold-39	42416196	G	A
Scaffold-39	42416212	A	G
Scaffold-39	42416220	GTT	GTTT
Scaffold-39	42416233	T	A
Scaffold-39	42416265	A	G
Scaffold-39	42433816	C	T
Scaffold-39	42433837	A	G
Scaffold-39	42511420	G	T
Scaffold-39	42511431	TC	TTGT,TT
Scaffold-39	42511433	G	A
Scaffold-39	42511455	AAC	AAG,TAG
Scaffold-39	42511478	A	G
Scaffold-39	42511497	C	T
Scaffold-39	42511502	T	A
Scaffold-39	42511506	A	G
Scaffold-39	42511514	A	G
Scaffold-39	42511521	G	A
Scaffold-39	42569937	A	G

Scaffold-39	42569939	T	C
Scaffold-39	42569944	T	A,C
Scaffold-39	42569953	GT	AC,AT
Scaffold-39	42569974	A	G
Scaffold-39	42569980	CCTAATT	CCT,TCT
Scaffold-39	42569992	GC	AT
Scaffold-39	42570003	GAA	GGA,GA
Scaffold-39	42570006	GATTCT	GACTCA,GACTCT
Scaffold-39	42570012	G	A
Scaffold-39	42570020	A	C
Scaffold-39	42570022	A	G
Scaffold-39	42570030	C	T
Scaffold-39	42570041	A	T
Scaffold-39	42570045	T	G
Scaffold-39	42570047	A	G
Scaffold-39	42570050	T	A
Scaffold-39	42570053	A	G
Scaffold-39	42570061	G	C
Scaffold-39	42691766	T	A
Scaffold-39	42785236	AGGGGGT	AGGGGT
Scaffold-39	42785264	CTCTATCTAT	CTCTGTCTAT,CTCTAT
Scaffold-39	42785284	G	T
Scaffold-39	42785295	G	T
Scaffold-39	42785316	G	C
Scaffold-39	42785325	C	T
Scaffold-39	42785345	G	T,A
Scaffold-39	42971784	C	T
Scaffold-39	43124874	TCAG	TCAA
Scaffold-39	43124898	GGAC	GGAT,AGAT
Scaffold-39	43124916	A	G
Scaffold-39	43124940	A	T
Scaffold-39	43124970	A	C
Scaffold-39	43124984	CTGTG	CTCTA,CTCTG,GTCTA
Scaffold-39	43125027	T	C
Scaffold-39	43125043	G	A
Scaffold-39	43125051	A	G
Scaffold-39	43125092	G	C
Scaffold-39	43125102	T	A
Scaffold-39	43125150	AATCTCAA	TATGTCAG,TATCTCAA
Scaffold-39	43187184	T	G
Scaffold-39	43187262	G	A
Scaffold-39	43187273	T	C
Scaffold-39	43187295	C	A
Scaffold-39	43630256	A	G

Scaffold-39	43630271	GACA	GA
Scaffold-39	43630275	CA	GT
Scaffold-39	43630309	C	A
Scaffold-39	43630317	C	T
Scaffold-39	43630326	T	C
Scaffold-39	43630342	GCTT	GCTA,GCT,TCTA,GCTTT
Scaffold-39	43630373	C	T
Scaffold-39	43681543	A	T
Scaffold-39	43681604	C	T
Scaffold-39	43681626	A	G
Scaffold-39	43750743	C	T
Scaffold-39	43768425	A	G
Scaffold-39	43784085	C	T
Scaffold-39	43784100	T	C
Scaffold-39	43784117	T	C
Scaffold-39	43784127	CA	CG,TG
Scaffold-39	43784138	G	A
Scaffold-39	43784156	A	T
Scaffold-39	43784202	T	C
Scaffold-39	43939942	G	A
Scaffold-39	44045051	G	T
Scaffold-39	44045080	A	T
Scaffold-39	44045086	T	C,A
Scaffold-39	44320640	G	T
Scaffold-39	44320664	T	C
Scaffold-39	44371154	C	A
Scaffold-39	44371178	C	T,A
Scaffold-39	44371187	ATGCTGCTGCTGCTGCTGCT	ATGCTGCTGCTGCTGCT,ATGCTGCTGCTGCTGCTGCTGCT,ATGCTGCTGCT
Scaffold-39	44371959	CGG	TGC
Scaffold-39	44371969	G	A
Scaffold-39	44371981	G	A
Scaffold-39	44371997	C	T
Scaffold-39	44372000	A	T
Scaffold-39	44372009	C	G
Scaffold-39	44372014	G	A
Scaffold-39	44372020	T	C
Scaffold-39	44372031	T	C
Scaffold-39	44372036	C	T
Scaffold-39	44372038	A	G
Scaffold-39	44372040	G	A
Scaffold-39	44372055	T	A
Scaffold-39	44372056	C	T
Scaffold-39	44372075	C	T

Scaffold-39	44723260	T	A	
Scaffold-39	44723263	G	A	
Scaffold-39	44723291	A	G	
Scaffold-39	44723306	A	G	
Scaffold-39	44723323	T	C	
Scaffold-39	44824213	A	G	
Scaffold-39	44824216	A	T	
Scaffold-39	44824243	C	T	
Scaffold-39	44824246	C	A	
Scaffold-39	44824338	C	A	
Scaffold-39	44824378	C	T	
Scaffold-39	44824387	G	A	
Scaffold-39	44824391	CCCTCCTC	CCCTC	
Scaffold-39	44824437	T	C	
Scaffold-39	44824454	G	T	
Scaffold-39	44909199	G	A	
Scaffold-39	44909229	T	C	
Scaffold-39	44909247	G	A	
Scaffold-39	44909259	A	G	
Scaffold-39	44909268	T	C	
Scaffold-39	44909274	A	C	
Scaffold-39	44909298	T	C	
Scaffold-39	44909301	C	T	
Scaffold-39	45015081	C	A	
Scaffold-39	45015091	AATA	AA	
Scaffold-39	45015111	C	T	
Scaffold-39	45405965	G	A	
Scaffold-39	45406003	A	T	
Scaffold-39	45406017	G	T	
Scaffold-39	45406028	G	T	
Scaffold-39	45406055	G	T	
Scaffold-39	45476943	T	C	
Scaffold-39	45476957	T	C	
Scaffold-39	45476967	A	T	
Scaffold-39	45476974	A	G	
Scaffold-39	45477001	C	G	
Scaffold-39	45477034	A	G	
Scaffold-39	45490012	T	A	
Scaffold-39	45490041	C	A	
Scaffold-39	45490048	C	T	
Scaffold-39	45490086	A	T	
Scaffold-39	45490099	ATCG	GTCA,ATTG,GTCG	700
Scaffold-39	45490108	G	A	
Scaffold-39	45511489	T	A,C	

Scaffold-39	45603365	A	G
Scaffold-39	45605048	T	C
Scaffold-39	45605072	C	A
Scaffold-39	46072059	G	A
Scaffold-39	46103454	TG	TGGAAGGCG
Scaffold-39	46103476	A	G
Scaffold-39	46103482	T	C
Scaffold-39	46103483	A	G
Scaffold-39	46103487	T	C
Scaffold-39	46103490	T	C
Scaffold-39	46103498	TT	TGT
Scaffold-39	46103514	TC	TT
Scaffold-39	46103516	TT	TC
Scaffold-39	46103525	AC	AT,GT
Scaffold-39	46103544	T	C
Scaffold-39	46301418	A	T
Scaffold-39	46301506	C	T
Scaffold-39	46611826	C	G
Scaffold-39	46611861	T	C
Scaffold-39	46611897	G	T
Scaffold-39	46611903	C	T
Scaffold-39	46678650	C	T
Scaffold-39	47093546	C	T
Scaffold-39	47093575	T	A
Scaffold-39	47093581	C	T
Scaffold-39	47093593	G	A
Scaffold-39	47093601	T	C
Scaffold-39	47094222	T	C
Scaffold-39	47094231	G	A
Scaffold-39	47094251	C	T
Scaffold-39	47094310	A	T
Scaffold-39	47094319	C	T
Scaffold-39	47094323	A	G
Scaffold-39	47861099	C	T
Scaffold-39	47861120	G	A
Scaffold-39	47861147	C	T
Scaffold-39	47861153	C	T
Scaffold-39	47861178	T	C
Scaffold-39	47861192	T	C
Scaffold-39	47861196	G	T
Scaffold-39	47861204	G	A
Scaffold-39	47861207	G	A
Scaffold-39	47861217	G	A
Scaffold-39	48016109	A	G

Scaffold-39	48016135	G	T
Scaffold-39	48081110	A	C
Scaffold-39	48081152	G	A
Scaffold-39	48246816	G	A
Scaffold-39	48246910	G	A
Scaffold-39	48329282	C	T
Scaffold-39	48329283	G	T
Scaffold-39	48329291	G	A
Scaffold-39	48329331	T	C
Scaffold-39	48329335	GATT	GT
Scaffold-39	48329402	G	A
Scaffold-39	48448193	C	A
Scaffold-39	48448200	C	T
Scaffold-39	48448224	G	C
Scaffold-39	48448229	C	T
Scaffold-39	48448253	A	T
Scaffold-39	48448268	G	A
Scaffold-39	48473075	C	T
Scaffold-39	48473117	A	G
Scaffold-39	48679463	C	T
Scaffold-39	48679500	T	C
Scaffold-39	48726566	T	C
Scaffold-39	48726670	G	C
Scaffold-39	48886332	C	T
Scaffold-39	48886333	G	A
Scaffold-39	48886354	G	T
Scaffold-39	48886367	G	C
Scaffold-39	48886371	G	A
Scaffold-39	48886418	T	A
Scaffold-39	48886428	A	T
Scaffold-39	48886430	G	A
Scaffold-39	48886441	G	A
Scaffold-39	48937445	T	C
Scaffold-39	48937477	A	G
Scaffold-39	48937480	A	C
Scaffold-39	49000233	CCAGACAGACAGACAGAC	CCAGACAGACAGAC,CCAGACAGACAGACAGAGGAATT,CCAGACAGACAGACAGACAGAC,CCAGACAGACAGACGAATT,CCAGACAGACAGAGGAATT,CCAGACAGAGGAATT
Scaffold-39	49000262	C	A
Scaffold-39	49000274	A	G
Scaffold-39	49000288	A	G
Scaffold-39	49000296	A	G
Scaffold-39	49000313	T	C
Scaffold-39	49000318	AAT	AAG,TAG

Scaffold-39	49000349	G	A
Scaffold-39	49125827	A	T
Scaffold-39	49125834	T	C
Scaffold-39	49125835	C	T
Scaffold-39	49125887	A	G
Scaffold-39	49125907	A	T
Scaffold-39	49125914	A	G
Scaffold-39	49125917	G	A
Scaffold-39	49125919	C	T
Scaffold-39	49125920	GGTTGAACTATGTAAA	GAT
Scaffold-39	49125941	C	A
Scaffold-39	49125944	C	T
Scaffold-39	49190607	C	G
Scaffold-39	49190651	TATTATCAA	TATTATCAATTATCAA,TCTTATCAA,TATTATGAA
Scaffold-39	49190687	A	T
Scaffold-39	49190700	CC	CAC
Scaffold-39	49190704	T	C
Scaffold-39	49190715	G	A
Scaffold-39	49190726	G	C
Scaffold-39	49364621	T	C
Scaffold-39	49364656	GGCGA	TGCGA
Scaffold-39	49364691	T	A
Scaffold-39	49364698	C	T
Scaffold-39	49364712	A	T
Scaffold-39	49364733	C	T
Scaffold-39	49364755	G	T
Scaffold-39	49384914	T	G
Scaffold-39	49384952	TATGTCA	TA
Scaffold-39	49384982	GCCAA	ACCAA,ACCAG
Scaffold-39	49384996	T	C
Scaffold-39	49384997	G	A
Scaffold-39	49385001	C	T
Scaffold-39	49385007	G	A
Scaffold-39	49385015	T	A
Scaffold-39	49583647	AGACATTG	AGATATTG
Scaffold-39	49583667	C	G
Scaffold-39	49583686	G	A
Scaffold-39	49973487	C	T
Scaffold-39	49973492	T	C
Scaffold-39	49973504	C	T
Scaffold-39	49973539	A	T
Scaffold-39	49973567	A	T
Scaffold-39	49973589	G	A

Scaffold-39	49973606		T	C
Scaffold-39	49973639		C	T
Scaffold-39	49973723		G	A
Scaffold-4	97452	T	G	
Scaffold-4	97465	G	A	
Scaffold-4	97466	G	C	
Scaffold-4	97507	G	A	
Scaffold-4	97531	C	G	
Scaffold-4	97545	G	A	
Scaffold-4	97565	G	A	
Scaffold-4	2566487		T	G
Scaffold-4	2566507		T	A,C
Scaffold-4	2566508		G	A
Scaffold-4	2566542		A	T
Scaffold-4	2566555		T	C
Scaffold-4	2566573		T	A
Scaffold-4	2566588		CTCAT	CTCATCAT
Scaffold-4	4706779		C	T
Scaffold-4	4706780		T	G
Scaffold-4	11429273		T	C
Scaffold-4	14029647		C	A
Scaffold-4	14029656		G	A
Scaffold-4	14029659		G	A
Scaffold-4	14029663		GCGGA	GCGGC
Scaffold-4	14029668		T	C
Scaffold-4	14029691		T	G
Scaffold-4	14029698		A	T
Scaffold-4	14029734		T	C
Scaffold-4	14029746		G	A
Scaffold-4	14390483		G	A
Scaffold-4	16051275		A	C
Scaffold-4	16051287		G	T
Scaffold-4	16051323		TTCA	TTCC
Scaffold-4	16051332		T	C
Scaffold-4	16051366		T	C
Scaffold-4	17166317		AGT	AGC
Scaffold-4	17166320		C	A
Scaffold-4	17166321		G	T
Scaffold-4	17166325		CCTGG	CCCGG
Scaffold-4	17166339		C	T
Scaffold-4	17166340		GTC	ATC
Scaffold-4	17166360		AG	GG
Scaffold-4	17166365		G	A
Scaffold-4	17166367		A	G

Scaffold-4	17166379	C	T	
Scaffold-4	17166381	T	A	
Scaffold-4	17166384	GCC	CCT	
Scaffold-4	17166396	G	A	
Scaffold-4	17166422	CT	CG	
Scaffold-4	17166437	CA	CG	
Scaffold-4	17166445	GACAA		GACAG
Scaffold-4	17284170	T	A	
Scaffold-4	17284176	G	A	
Scaffold-4	17284203	A	G	
Scaffold-4	17284254	GTAC	ATAC	
Scaffold-4	17284301	A	C	
Scaffold-4	17505340	G	A	
Scaffold-4	17505347	G	C	
Scaffold-4	17505388	G	A	
Scaffold-4	20696931	A	G	
Scaffold-4	20696937	G	C	
Scaffold-4	20696941	T	C	
Scaffold-4	20696951	A	G	
Scaffold-4	20696957	CTGAT		CTGATGAT
Scaffold-4	20696974	T	G	
Scaffold-4	20697003	T	C	
Scaffold-4	20697031	C	G	
Scaffold-4	23184236	G	A	
Scaffold-4	23939138	A	C	
Scaffold-4	24457934	T	G	
Scaffold-4	24457939	T	A	
Scaffold-4	24457945	ATT	AT	
Scaffold-4	24457992	TTCG	ATCG,ATCA	
Scaffold-4	24458004	T	C	
Scaffold-4	24458016	A	G	
Scaffold-4	24458043	C	T	
Scaffold-4	30510710	G	A	
Scaffold-4	30510711	T	C	
Scaffold-4	32963720	G	A	
Scaffold-4	32963742	TG	TC	
Scaffold-4	32963761	C	A	
Scaffold-4	33724297	G	A	
Scaffold-4	33744011	T	A	
Scaffold-4	33744064	A	G	
Scaffold-4	33744070	A	G	
Scaffold-4	33744076	A	G	
Scaffold-4	33744083	C	A	
Scaffold-4	35982716	G	A	

Scaffold-4	40751571		A	C	
Scaffold-4	40751581		T	A	
Scaffold-4	40751598		C	T	
Scaffold-4	40751629		A	G	
Scaffold-4	40751638		A	C	
Scaffold-4	40751650		A	G	
Scaffold-4	40751656		G	A	
Scaffold-4	45342309		A	G	
Scaffold-4	45440956		G	C	
Scaffold-4	49297989		G	A	
Scaffold-4	49298039		ATTTG		ATTTGTTG
Scaffold-4	49298053		T	C	
Scaffold-4	49298083		C	T	
Scaffold-40	24303	A	G		
Scaffold-40	24322	A	C		
Scaffold-40	24396	A	G		
Scaffold-40	24403	A	G		
Scaffold-40	24405	T	G		
Scaffold-40	81032	G	C		
Scaffold-40	81043	G	A		
Scaffold-40	81050	C	T		
Scaffold-40	81051	G	A		
Scaffold-40	81067	C	T		
Scaffold-40	207119		G	A	
Scaffold-40	207143		C	G	
Scaffold-40	207153		A	G	
Scaffold-40	207158		A	T	
Scaffold-40	207164		TAAT	TT	
Scaffold-40	267418		C	A	
Scaffold-40	267442		T	G	
Scaffold-40	267520		T	C	
Scaffold-40	359267		G	A	
Scaffold-40	359281		A	G	
Scaffold-40	359287		C	A	
Scaffold-40	541684		G	T	
Scaffold-40	541693		C	A	
Scaffold-40	541717		G	A	
Scaffold-40	541746		G	A	
Scaffold-40	541821		T	C	
Scaffold-40	541834		C	A	
Scaffold-40	541850		G	A	
Scaffold-40	561712		G	A	
Scaffold-40	561742		A	C	
Scaffold-40	561762		A	T	

Scaffold-40	561777	A	T
Scaffold-40	561792	C	T
Scaffold-40	592481	G	T
Scaffold-40	592491	T	C
Scaffold-40	592509	A	C
Scaffold-40	592535	C	T
Scaffold-40	592578	C	G
Scaffold-40	592582	A	G
Scaffold-40	592587	T	A
Scaffold-40	592603	T	G
Scaffold-40	592617	A	G
Scaffold-40	948941	G	A
Scaffold-40	948971	T	A
Scaffold-40	948983	C	T
Scaffold-40	948999	TGG	TGGTTTGGCACTAGATTCAACACG,AGGTTTGGCACTAGATTCAACACG
Scaffold-40	949005	A	G
Scaffold-40	1365776	G	A
Scaffold-40	1365829	A	G
Scaffold-40	1365869	T	C
Scaffold-40	1873577	C	T
Scaffold-40	1873593	G	A
Scaffold-40	2337927	A	G
Scaffold-40	2337942	TTA	TTT,ATT
Scaffold-40	2337945	TT	TG
Scaffold-40	2337957	TAATGATTAGGGA	TAATGATTAGGGAATGATTAGGGA
Scaffold-40	2338013	C	T
Scaffold-40	2338023	C	T
Scaffold-40	2338033	C	A
Scaffold-40	2338062	C	T
Scaffold-40	2338124	GGATG	AGATA
Scaffold-40	2338134	G	A
Scaffold-40	2338160	T	A
Scaffold-40	2338184	G	A
Scaffold-40	2338195	A	T
Scaffold-40	2338203	T	G
Scaffold-40	2355579	G	A
Scaffold-40	2488280	T	C
Scaffold-40	2540913	GTT	GT
Scaffold-40	2556317	G	A
Scaffold-40	2566056	G	A
Scaffold-40	2566084	T	C
Scaffold-40	2566085	G	C

Scaffold-40	2597330	G	T	
Scaffold-40	2597334	G	A	
Scaffold-40	2597353	T	C	
Scaffold-40	2606513	A	G	
Scaffold-40	2606526	C	T	
Scaffold-40	2606534	A	C	
Scaffold-40	2616170	CT	AG,CG	
Scaffold-40	2616203	T	C	
Scaffold-40	2616214	T	A	
Scaffold-40	2754250	C	T	
Scaffold-40	2754285	G	A	
Scaffold-40	3042006	AATATATATATA		AATATATATATATA
Scaffold-40	3137983	CG	TT	
Scaffold-40	3138004	C	T	
Scaffold-40	3138053	C	T	
Scaffold-40	3138064	A	G	
Scaffold-40	3138079	A	T	
Scaffold-40	3184893	C	T	
Scaffold-40	3185008	G	A	
Scaffold-40	3394543	A	C	
Scaffold-40	3394577	ATT	ATTT	
Scaffold-40	3394600	C	T	
Scaffold-40	3394646	C	T	
Scaffold-40	3411619	T	C	
Scaffold-40	3411628	T	C	
Scaffold-40	3411637	A	G	
Scaffold-40	3411639	G	A	
Scaffold-40	3411640	T	C	
Scaffold-40	3411679	T	A	
Scaffold-40	3451066	C	T	
Scaffold-40	3451080	TTA	TTG	
Scaffold-40	3451124	C	T	
Scaffold-40	3451145	CAT	CAC	
Scaffold-40	3451166	G	A	
Scaffold-40	3632230	T	C	
Scaffold-40	3632240	T	C	
Scaffold-40	3632245	G	A	
Scaffold-40	3632248	GGA	TGG	
Scaffold-40	3632264	G	A	
Scaffold-40	3632275	T	C	
Scaffold-40	3632280	C	T	
Scaffold-40	3632287	G	A	
Scaffold-40	3632298	A	G	
Scaffold-40	3632317	C	G	

Scaffold-40	3815877	A	G
Scaffold-40	3815928	T	A
Scaffold-40	3815970	A	G
Scaffold-40	3815971	C	A
Scaffold-40	3819007	G	C
Scaffold-40	3819052	C	T
Scaffold-40	3869139	C	A
Scaffold-40	3869175	A	C
Scaffold-40	3869217	A	G
Scaffold-40	3869221	G	A
Scaffold-40	4033575	C	T
Scaffold-40	4033592	T	A
Scaffold-40	4033606	T	A
Scaffold-40	4353328	G	A
Scaffold-40	4479989	A	T
Scaffold-40	4479996	C	A
Scaffold-40	4480017	G	C
Scaffold-40	4480031	T	C
Scaffold-40	4480055	CAAAAAAG	CAAAAAAAG
Scaffold-40	4480063	A	G
Scaffold-40	4480084	T	C
Scaffold-40	4539575	C	T
Scaffold-40	4539604	G	C
Scaffold-40	4539615	TAG	TAAG
Scaffold-40	4753696	G	A
Scaffold-40	4753727	G	C
Scaffold-40	4753732	T	G
Scaffold-40	4753744	C	T
Scaffold-40	4801897	G	A
Scaffold-40	4801931	G	A
Scaffold-40	4801942	GCCC	ACCT,GCCT,GCGT
Scaffold-40	4801960	G	T
Scaffold-40	4801971	T	A
Scaffold-40	4801976	G	T
Scaffold-40	4801998	TAAG	TAAA,CAAA
Scaffold-40	4906178	C	T
Scaffold-40	4906185	G	T
Scaffold-40	4906194	A	G
Scaffold-40	5172558	A	G
Scaffold-40	5172580	T	C
Scaffold-40	5172621	G	T
Scaffold-40	5824452	C	T
Scaffold-40	5824511	G	A
Scaffold-40	5825081	GC	CA

Scaffold-40	5825103	A	G	
Scaffold-40	5825118	C	T	
Scaffold-40	5825162	A	T	
Scaffold-40	5825164	T	C	
Scaffold-40	5825176	A	G,T	
Scaffold-40	5825198	G	C	
Scaffold-40	5941739	G	A	
Scaffold-40	5941763	G	A	
Scaffold-40	5941785	C	T	
Scaffold-40	5941802	A	C	
Scaffold-40	5941810	G	A	
Scaffold-40	5941816	AGC	AC	
Scaffold-40	5941834	G	C	
Scaffold-40	5941858	G	T	
Scaffold-40	5941860	T	A	
Scaffold-40	6167934	CCTGT		CCTGC
Scaffold-40	6167944	T	C	
Scaffold-40	6167960	T	A	
Scaffold-40	6167974	AGA	AGT,TGT	
Scaffold-40	6167994	G	A	
Scaffold-40	6167996	GCAT	ACAT,ACAC	
Scaffold-40	6168007	AT	ATGAAACT	
Scaffold-40	6168013	T	C	
Scaffold-40	6168028	G	T	
Scaffold-40	6168037	G	A	
Scaffold-40	6168046	T	A	
Scaffold-40	6168052	A	G	
Scaffold-40	6168054	TC	CC,CT	
Scaffold-40	6168066	T	C	
Scaffold-40	6199180	C	A	
Scaffold-40	6199208	A	T	
Scaffold-40	6199235	A	C	
Scaffold-40	6199247	A	T	
Scaffold-40	6199285	G	A	
Scaffold-40	6199290	C	T	
Scaffold-40	6675401	A	G	
Scaffold-40	6675420	C	T	
Scaffold-40	6675437	A	G	
Scaffold-40	6675525	C	A	
Scaffold-40	6830719	T	C	
Scaffold-40	7116151	G	A	
Scaffold-40	7258801	A	C	
Scaffold-40	7258813	T	C	
Scaffold-40	7258834	T	C	

Scaffold-40	7291002	C	G
Scaffold-40	7291072	C	T
Scaffold-40	7291124	ATTG	ATTCG
Scaffold-40	7291135	T	C
Scaffold-40	7372656	A	T
Scaffold-40	7372668	T	C
Scaffold-40	7372735	G	A
Scaffold-40	7416351	A	C
Scaffold-40	7416356	T	C
Scaffold-40	7416363	A	G
Scaffold-40	7416381	T	G
Scaffold-40	7416387	TGG	TG
Scaffold-40	7416402	C	T
Scaffold-40	7416429	GACAA	GACA,GCCAAA,TACA,GATA
Scaffold-40	7416460	GACGTA	GACTTA,GA
Scaffold-40	7416471	G	A
Scaffold-40	7416474	G	T
Scaffold-40	7416493	C	T
Scaffold-40	8180230	C	G
Scaffold-40	8180231	A	T
Scaffold-40	8180236	G	A
Scaffold-40	8180248	C	T
Scaffold-40	8180261	A	G
Scaffold-40	8213725	C	T
Scaffold-40	8213737	C	T
Scaffold-40	8213743	C	T
Scaffold-40	8213772	C	A
Scaffold-40	8213788	C	T
Scaffold-40	8213821	GTT	GTTT
Scaffold-40	8213824	A	T
Scaffold-40	8397295	ACCAACTGCCAACTGC	ACCAACTGCTAACTGC,ACCAACTGC,ACCAGCTGCCAACTGC
Scaffold-40	8397330	G	C
Scaffold-40	8397341	T	C
Scaffold-40	8397355	GC	GTC
Scaffold-40	8397363	C	T
Scaffold-40	8397366	C	T
Scaffold-40	8397392	C	T,A
Scaffold-40	8397414	G	T
Scaffold-40	8581897	T	A
Scaffold-40	8581920	C	G,T
Scaffold-40	8581938	C	T
Scaffold-40	8581962	CGATA	TGATT,CGTTA
Scaffold-40	8581980	GGTG	GG

Scaffold-40	8581986	ATCTA	ATCTG,TTCTG
Scaffold-40	8582024	ATGG	ATGTGG
Scaffold-40	8737352	T	C
Scaffold-40	8737353	A	G
Scaffold-40	8737368	C	T
Scaffold-40	8737387	A	C
Scaffold-40	8737424	G	A
Scaffold-40	8818141	CATGATGATGA	CATGATGATGATGA
Scaffold-40	8916682	A	G
Scaffold-40	8916692	T	C
Scaffold-40	8939868	A	G
Scaffold-40	8939869	C	T
Scaffold-40	8939880	T	A
Scaffold-40	8939890	A	T
Scaffold-40	8939914	T	C
Scaffold-40	8939916	T	C
Scaffold-40	8939929	G	A
Scaffold-40	8974475	C	T
Scaffold-40	8974493	C	G
Scaffold-40	8974535	A	G
Scaffold-40	9082630	T	A
Scaffold-40	9082659	T	C
Scaffold-40	9082685	G	A
Scaffold-40	9082727	A	T
Scaffold-40	9156890	C	T
Scaffold-40	9156944	A	T
Scaffold-40	9375691	A	C
Scaffold-40	9375705	T	C
Scaffold-40	9375734	C	A
Scaffold-40	9375761	T	C
Scaffold-40	9375772	A	T
Scaffold-40	9375791	C	T
Scaffold-40	9375792	C	T
Scaffold-40	9375793	CG	CC
Scaffold-40	9596614	T	C
Scaffold-40	9596626	G	A
Scaffold-40	9596633	C	G
Scaffold-40	9596640	C	T
Scaffold-40	9596650	G	A
Scaffold-40	9596679	T	C
Scaffold-40	9596694	A	G
Scaffold-40	9659021	T	C
Scaffold-40	9659061	ATATT	AT
Scaffold-40	9659090	T	G

Scaffold-40	9659105	G	C,A
Scaffold-40	10052739	C	A
Scaffold-40	10052740	G	A
Scaffold-40	10052770	C	A
Scaffold-40	10052781	G	A
Scaffold-40	10052787	A	G
Scaffold-40	10052807	A	C
Scaffold-40	10052815	C	T
Scaffold-40	10088932	G	T
Scaffold-40	10088988	G	A
Scaffold-40	10089003	T	C
Scaffold-40	10089010	G	A
Scaffold-40	10089028	C	A
Scaffold-40	10089042	T	A
Scaffold-40	10482663	C	A
Scaffold-40	10482667	T	G
Scaffold-40	10482708	C	T
Scaffold-40	10482711	G	A
Scaffold-40	10482717	G	A
Scaffold-40	10482770	TTCT	CTCA
Scaffold-40	10482788	A	T
Scaffold-40	10567428	A	T
Scaffold-40	10567446	A	G
Scaffold-40	10567450	T	G
Scaffold-40	10567451	ACAATCC	ACAATCCAATCC
Scaffold-40	10567497	A	G
Scaffold-40	10567515	CATATGAA	CA
Scaffold-40	10567524	C	A
Scaffold-40	10669780	T	A
Scaffold-40	10799870	T	A
Scaffold-40	10799873	C	T
Scaffold-40	10799931	G	T
Scaffold-40	10799978	T	C
Scaffold-40	10926994	T	C
Scaffold-40	10928170	T	C
Scaffold-40	10928182	T	C
Scaffold-40	10929457	G	C
Scaffold-40	10929545	A	G
Scaffold-40	10993411	A	T
Scaffold-40	10993511	G	T
Scaffold-40	10993544	A	T
Scaffold-40	11075303	T	G
Scaffold-40	11126139	C	T
Scaffold-40	11126143	TGTCG	TG

Scaffold-40	11126170	GCT	GCCT
Scaffold-40	11168577	C	T
Scaffold-40	11168578	ATTTTTTA	ATTTTTA
Scaffold-40	11168587	C	A
Scaffold-40	11168606	A	G
Scaffold-40	11168624	A	G
Scaffold-40	11261003	T	C
Scaffold-40	11261014	T	C
Scaffold-40	11261036	C	A
Scaffold-40	11261056	T	C
Scaffold-40	11356226	AGT	GGT
Scaffold-40	11356237	C	T
Scaffold-40	11356240	G	A
Scaffold-40	11612782	C	T
Scaffold-40	11612838	T	A
Scaffold-40	11612849	T	A
Scaffold-40	11612881	TGTTCTTCTATAT	TGTTCTCCTATAT,CGTT
Scaffold-40	11749800	T	A
Scaffold-40	11749816	A	G
Scaffold-40	11791233	T	C
Scaffold-40	11791282	T	C
Scaffold-40	11791284	C	G
Scaffold-40	11791312	A	G
Scaffold-40	11791315	G	A,C
Scaffold-40	11791320	C	A
Scaffold-40	11803704	T	G
Scaffold-40	11803734	G	T
Scaffold-40	11803747	C	A
Scaffold-40	11803773	C	T
Scaffold-40	11803781	G	C
Scaffold-40	11803806	A	C
Scaffold-40	11803808	T	A
Scaffold-40	11803814	CAAAAAT	CAAAAT
Scaffold-40	11803822	A	G
Scaffold-40	11803824	T	C
Scaffold-40	11803832	AT	TA
Scaffold-40	11803845	G	T
Scaffold-40	11834325	A	G
Scaffold-40	11834340	A	G
Scaffold-40	11834346	C	T
Scaffold-40	11834360	ATATATTATA,TTATG	
Scaffold-40	11834368	A	G
Scaffold-40	11834370	T	C
Scaffold-40	11834395	A	G

Scaffold-40	11834408	T	C
Scaffold-40	11834421	G	A
Scaffold-40	11834425	A	C
Scaffold-40	12024231	A	G
Scaffold-40	12024241	G	A
Scaffold-40	12024243	A	G
Scaffold-40	12024251	T	G
Scaffold-40	12024280	G	T
Scaffold-40	12024363	G	A
Scaffold-40	12070316	A	T
Scaffold-40	12070365	G	T
Scaffold-40	12095130	T	C
Scaffold-40	12095133	C	T
Scaffold-40	12095168	C	T
Scaffold-40	12124774	C	T
Scaffold-40	12124799	T	C
Scaffold-40	12124816	A	G
Scaffold-40	12291374	T	A
Scaffold-40	12296983	A	G,T
Scaffold-40	12297020	A	G
Scaffold-40	12297037	G	A
Scaffold-40	12297064	G	T,C
Scaffold-40	12297065	G	T
Scaffold-40	12297115	A	T
Scaffold-40	12327282	T	C
Scaffold-40	12327312	G	T
Scaffold-40	12327322	A	G
Scaffold-40	12327373	G	A
Scaffold-40	12327374	G	A
Scaffold-40	12327383	T	A
Scaffold-40	12359432	T	A
Scaffold-40	12359451	CACATGAACATGAACATGAT	CACATGAACATGAACATGAACATGAT,CACATGAACATGACCATGAACATGAACATGAACATGAACATGAT,CACATGAACATGAACATGAACATGAACATGAACATGAT
Scaffold-40	12359471	A	C
Scaffold-40	12359530	G	A
Scaffold-40	12359544	A	G
Scaffold-40	12563712	G	A
Scaffold-40	12563784	T	C
Scaffold-40	12563789	A	C
Scaffold-40	12563792	A	C
Scaffold-40	12563812	T	C
Scaffold-40	12611080	G	C
Scaffold-40	12611084	CATG	CG

Scaffold-40	12611091	TTAATTGATGA	TA
Scaffold-40	12611121	T	C
Scaffold-40	12611133	ATTT	ATTC,CTTC
Scaffold-40	12611137	A	T
Scaffold-40	12611151	A	G
Scaffold-40	12723348	TC	CA
Scaffold-40	12723368	G	A
Scaffold-40	12723389	T	G
Scaffold-40	12723394	A	C
Scaffold-40	12723423	T	A
Scaffold-40	12723434	G	T
Scaffold-40	12723449	C	T
Scaffold-40	12723454	T	A
Scaffold-40	12723463	GTT	GTTT
Scaffold-40	12723475	G	A
Scaffold-40	12839639	G	A
Scaffold-40	12839684	T	A
Scaffold-40	12839731	TA	AT
Scaffold-40	12839737	C	T
Scaffold-40	12940279	T	C
Scaffold-40	12940302	C	G
Scaffold-40	13109610	T	A
Scaffold-40	13109615	C	T
Scaffold-40	13109621	C	T
Scaffold-40	13109633	A	T
Scaffold-40	13109642	G	A
Scaffold-40	13109648	A	G
Scaffold-40	13109650	C	G
Scaffold-40	13109710	T	C
Scaffold-40	13120746	C	T
Scaffold-40	13120844	G	T
Scaffold-40	13120845	G	A
Scaffold-40	13120867	A	G
Scaffold-40	13270416	A	G
Scaffold-40	13270485	A	T
Scaffold-40	13345889	A	T
Scaffold-40	13345950	T	C
Scaffold-40	13345964	C	A
Scaffold-40	13880398	A	G
Scaffold-40	13880413	G	C
Scaffold-40	13914378	T	G
Scaffold-40	13914380	CACA	CCC,CACAA
Scaffold-40	13914388	A	T
Scaffold-40	13914397	C	A

Scaffold-40	13914404	CAA	CA	
Scaffold-40	13914414	A	G	
Scaffold-40	13914415	AAG	GAA	
Scaffold-40	13915236	C	T	
Scaffold-40	13915253	T	C	
Scaffold-40	13915267	G	A	
Scaffold-40	13915306	C	A	
Scaffold-40	13915307	G	A	
Scaffold-40	13915328	G	A	
Scaffold-40	13915332	T	C	
Scaffold-40	13915360	C	T	
Scaffold-40	14155833	A	G	
Scaffold-40	14155901	T	G	
Scaffold-40	14155928	T	A	
Scaffold-40	14155931	C	T	
Scaffold-40	14193955	G	C	
Scaffold-40	14193961	TGAGG		TG
Scaffold-40	14193973	GTG	GG	
Scaffold-40	14193982	G	A	
Scaffold-40	14475193	C	T	
Scaffold-40	14475267	T	A	
Scaffold-40	14611583	TTG	GCA	
Scaffold-40	14611639	C	T	
Scaffold-40	14677492	T	A	
Scaffold-40	14769167	T	G	
Scaffold-40	14769217	C	T	
Scaffold-40	14769223	C	T	
Scaffold-40	14769261	T	C	
Scaffold-40	15037913	T	C	
Scaffold-40	15037937	T	C	
Scaffold-40	15037946	TAA	TAAA	
Scaffold-40	15037991	A	C,G	
Scaffold-40	15037993	T	C	
Scaffold-40	15200376	C	T	
Scaffold-40	15200438	G	A	
Scaffold-40	15200471	G	A	
Scaffold-40	15200475	G	A	
Scaffold-40	15200506	A	C,G	
Scaffold-40	15200556	A	G	
Scaffold-40	15200595	T	C	
Scaffold-40	15206234	G	C	
Scaffold-40	15206262	GC	AT	
Scaffold-40	15206265	GTC	GAA	
Scaffold-40	15206269	T	G	

Scaffold-40	15206317	TGC	TC
Scaffold-40	15206324	T	C
Scaffold-40	15320704	CTTGT	GTTGT
Scaffold-40	15320864	G	A
Scaffold-40	15320867	CAGTT	CT
Scaffold-40	15320874	G	A
Scaffold-40	15320886	ACCAA	GCCAA,GCCAT
Scaffold-40	15320896	GG	TT
Scaffold-40	15320910	C	T
Scaffold-40	15696811	T	A
Scaffold-40	15696815	C	A
Scaffold-40	15696837	T	C
Scaffold-40	15696868	A	G
Scaffold-40	15696877	G	A
Scaffold-40	15696908	T	C
Scaffold-40	15696934	T	A
Scaffold-40	15940606	G	A
Scaffold-40	16039765	T	G
Scaffold-40	16039790	T	C
Scaffold-40	16039799	T	C
Scaffold-40	16039824	A	G
Scaffold-40	16067309	T	C
Scaffold-40	16067328	G	C
Scaffold-40	16067332	C	T
Scaffold-40	16067337	T	A
Scaffold-40	16067339	TGTA	CGTG,CATG
Scaffold-40	16067344	G	A
Scaffold-40	16067360	C	T
Scaffold-40	16067375	A	C
Scaffold-40	16067388	G	T
Scaffold-40	16067391	C	G
Scaffold-40	16067393	C	T
Scaffold-40	16067408	T	C
Scaffold-40	16067425	T	C
Scaffold-40	16067432	C	T
Scaffold-40	16067447	T	C
Scaffold-40	16169077	C	T
Scaffold-40	16169104	T	A
Scaffold-40	16264523	G	T
Scaffold-40	16377766	C	T
Scaffold-40	16377786	A	G
Scaffold-40	16377791	C	T
Scaffold-40	16377815	A	G
Scaffold-40	16377837	A	C

Scaffold-40	16377850	G	C	
Scaffold-40	16459589	A	C	
Scaffold-40	16459601	G	A	
Scaffold-40	16459604	G	A	
Scaffold-40	16459607	C	T	
Scaffold-40	16459625	CAG	CAAG	
Scaffold-40	16508585	TAAAAAC		TAAAAC
Scaffold-40	16508597	T	C	
Scaffold-40	16508601	GTG	GTTG	800
Scaffold-40	16508611	G	A	
Scaffold-40	16508620	GC	GACC	
Scaffold-40	16508636	G	C	
Scaffold-40	16508639	GT	AC	
Scaffold-40	16508644	A	G	
Scaffold-40	16508645	G	T	
Scaffold-40	16508654	GATT	AATC	
Scaffold-40	16508659	G	C	
Scaffold-40	16508668	T	A	
Scaffold-40	16508669	T	A	
Scaffold-40	16508689	T	A	
Scaffold-40	16508690	A	T	
Scaffold-40	16560931	T	A	
Scaffold-40	16560964	A	G	
Scaffold-40	16560973	C	T	
Scaffold-40	16561036	G	A	
Scaffold-40	16561040	C	G	
Scaffold-40	16663238	A	G	
Scaffold-40	16663281	T	G	
Scaffold-40	16663302	C	T	
Scaffold-40	16923524	A	G	
Scaffold-40	16930503	T	C	
Scaffold-40	16930509	C	T	
Scaffold-40	16930528	T	A	
Scaffold-40	16930529	G	A	
Scaffold-40	16930532	A	C	
Scaffold-40	16930548	A	G	
Scaffold-40	17205047	T	G	
Scaffold-40	17205060	CT	AT,AC,CA,TT	
Scaffold-40	17205073	C	G	
Scaffold-40	17205077	A	T	
Scaffold-40	17205084	G	T	
Scaffold-40	17205092	G	A	
Scaffold-40	17205093	ATGC	TTGT	
Scaffold-40	17494877	A	G	

Scaffold-40	17494901	TCCGCCGCCGCCGC	TCCGCCGCCGCCGCCGCCGC,TTCTCCGCCGCCGC
Scaffold-40	17494946	A	C
Scaffold-40	17494950	G	A
Scaffold-40	17669855	A	C
Scaffold-40	17716167	G	A
Scaffold-40	17716170	A	G
Scaffold-40	17716227	C	T
Scaffold-40	17716233	A	T
Scaffold-40	17716303	G	T,A
Scaffold-40	17753187	C	T
Scaffold-40	17753191	A	G
Scaffold-40	17753215	A	C
Scaffold-40	17753219	T	A
Scaffold-40	17753229	A	G
Scaffold-40	17753261	T	G
Scaffold-40	17753268	A	G
Scaffold-40	17753284	G	C
Scaffold-40	17753300	G	A
Scaffold-40	17753304	C	T
Scaffold-40	17875006	A	C
Scaffold-40	17875016	A	G
Scaffold-40	17995917	G	T
Scaffold-40	17995928	G	A
Scaffold-40	17995930	A	T
Scaffold-40	17995934	G	A
Scaffold-40	17995938	G	A
Scaffold-40	17995954	C	A
Scaffold-40	17995964	T	G
Scaffold-40	17995983	G	A
Scaffold-40	17995992	C	A
Scaffold-40	17996008	G	A
Scaffold-40	17996023	T	C
Scaffold-40	17996042	G	C
Scaffold-40	18032325	A	G
Scaffold-40	18032343	T	A
Scaffold-40	18032397	G	A
Scaffold-40	18032418	T	C
Scaffold-40	18271013	T	A
Scaffold-40	18271025	CC	TC,TT
Scaffold-40	18271045	C	A
Scaffold-40	18271046	C	T
Scaffold-40	18271054	A	T
Scaffold-40	18271083	G	C

Scaffold-40	18271111	A	C	
Scaffold-40	18271114	C	G	
Scaffold-40	18271119	C	G	
Scaffold-40	18271123	G	T	
Scaffold-40	18271125	TT	TG	
Scaffold-40	18394048	T	C	
Scaffold-40	18394072	G	A	
Scaffold-40	18415690	A	G	
Scaffold-40	18415717	G	A	
Scaffold-40	18415774	C	A	
Scaffold-40	18415795	T	A	
Scaffold-40	18449901	T	A	
Scaffold-40	18450008	A	C	
Scaffold-40	18747816	C	T	
Scaffold-40	18747817	G	A	
Scaffold-40	18747823	A	G	
Scaffold-40	18747835	G	A	
Scaffold-40	18747863	A	C	
Scaffold-40	18747879	A	G	
Scaffold-40	18747888	A	C	
Scaffold-40	18747891	G	T	
Scaffold-40	18747926	A	G	
Scaffold-40	18747949	C	T	
Scaffold-40	18773245	C	A	
Scaffold-40	18773257	G	A	
Scaffold-40	18773280	G	A	
Scaffold-40	18773289	A	C	
Scaffold-40	18773295	A	G	
Scaffold-40	18773323	A	G	
Scaffold-40	18773365	G	T	
Scaffold-40	19121834	A	T	
Scaffold-40	19121848	C	T	
Scaffold-40	19121849	G	A	
Scaffold-40	19121876	G	C	
Scaffold-40	19121905	TAAAAAAG		TAAAAAAAG
Scaffold-40	19121923	T	G	
Scaffold-40	19121930	G	C	
Scaffold-40	19192426	C	G	
Scaffold-40	19192434	T	A	
Scaffold-40	19192440	G	T	
Scaffold-40	19192449	G	A	
Scaffold-40	19192467	G	A	
Scaffold-40	19192482	A	T	
Scaffold-40	19192573	C	T	

Scaffold-40	19192591	G	T
Scaffold-40	19192605	CCCAA	CCCAT,TCCAT
Scaffold-40	19192625	G	A
Scaffold-40	19192667	C	A
Scaffold-40	19282714	C	G
Scaffold-40	19282716	A	G
Scaffold-40	19282719	T	G
Scaffold-40	19282720	A	G
Scaffold-40	19282724	G	A
Scaffold-40	19282732	ATTTTTTTA	ATTGTTTTA,ATTTTTTA
Scaffold-40	19282751	G	A
Scaffold-40	19282795	T	C
Scaffold-40	19282819	G	A
Scaffold-40	19293482	T	G
Scaffold-40	19293493	G	A
Scaffold-40	19293503	G	A
Scaffold-40	19293507	T	C
Scaffold-40	19293529	A	T
Scaffold-40	19293532	C	A
Scaffold-40	19293559	A	C
Scaffold-40	19369852	T	C
Scaffold-40	19541289	A	G
Scaffold-40	19541319	T	C
Scaffold-40	19541345	A	G
Scaffold-40	19541354	CTTTTTTTA	CTTTTTTA
Scaffold-40	19713686	GGAGTT	GT
Scaffold-40	19713702	A	G
Scaffold-40	19713709	G	A
Scaffold-40	19713717	G	A
Scaffold-40	19713756	G	A
Scaffold-40	19713758	T	C
Scaffold-40	19713762	T	G
Scaffold-40	19713777	T	C
Scaffold-40	19747844	TATGGAGTCG	CATGGAGTCG
Scaffold-40	19747875	GAAAAAAAG	GAAAACAAG
Scaffold-40	19747884	T	C
Scaffold-40	19779609	C	T
Scaffold-40	19779613	A	G
Scaffold-40	19779634	G	A
Scaffold-40	19779639	G	A
Scaffold-40	19779656	A	G
Scaffold-40	19779657	C	T
Scaffold-40	19979965	G	A
Scaffold-40	19979969	C	T

Scaffold-40	19979970	G	A
Scaffold-40	19979973	A	G
Scaffold-40	19979974	C	G
Scaffold-40	19980002	CCCAG	TCCAG,TCCAA
Scaffold-40	19980012	T	A
Scaffold-40	19980035	ACA	GCA
Scaffold-40	19980049	A	G
Scaffold-40	19980074	A	G
Scaffold-40	20048315	A	G
Scaffold-40	20048333	A	G
Scaffold-40	20048353	G	A
Scaffold-40	20048361	AA	AATAGCCGCA
Scaffold-40	20048368	A	G
Scaffold-40	20048401	G	A
Scaffold-40	20048418	ATACTACT	ATACT
Scaffold-40	20048434	C	T
Scaffold-40	20048440	A	C
Scaffold-40	20048455	C	A
Scaffold-40	20061141	C	A
Scaffold-40	20061157	G	A
Scaffold-40	20061182	C	A
Scaffold-40	20061205	T	G
Scaffold-40	20061223	T	C
Scaffold-40	20061240	C	T
Scaffold-40	20144604	T	C
Scaffold-40	20144644	A	T
Scaffold-40	20144706	A	G
Scaffold-40	20145097	A	G
Scaffold-40	20145101	G	A
Scaffold-40	20145127	C	T
Scaffold-40	20145151	GAAAAAAT	GAAAGAAT
Scaffold-40	20180044	G	A
Scaffold-40	20180056	T	G
Scaffold-40	20180062	C	A
Scaffold-40	20180090	A	G
Scaffold-40	20180102	G	T
Scaffold-40	20662297	G	A
Scaffold-40	20662324	C	T
Scaffold-40	20662329	A	G
Scaffold-40	20662331	A	G
Scaffold-40	20662393	G	C
Scaffold-40	20799399	C	T
Scaffold-40	20799408	T	A
Scaffold-40	21215103	TTATTCT	TTACTCT

Scaffold-40	21215113	TGT	TT,TCT
Scaffold-40	21215119	GATACGATTGTTTACA	AATACGATTGTTTACA,GATACGACTGTTTACA
Scaffold-40	21215135	T	G
Scaffold-40	21215140	C	T
Scaffold-40	21215144	GTTTA	GTTTG
Scaffold-40	21215160	C	G,A
Scaffold-40	21215177	G	A
Scaffold-40	21215201	CTCTTAATTAAC	CTCTTTAAC,ATCTTAAC,CTCTTAAC
Scaffold-40	21318348	G	T
Scaffold-40	21318377	A	G
Scaffold-40	21318407	C	T
Scaffold-40	21443593	A	C
Scaffold-40	21443595	G	A
Scaffold-40	21443659	A	G
Scaffold-40	21443690	T	C
Scaffold-40	21443727	C	A
Scaffold-40	21443765	G	C
Scaffold-40	21443827	A	G
Scaffold-40	22086783	C	T
Scaffold-40	22086799	C	T
Scaffold-40	22086814	C	T
Scaffold-40	22086823	C	A
Scaffold-40	22609786	T	A
Scaffold-40	22609798	G	A
Scaffold-40	22609830	C	A
Scaffold-40	22609834	G	A
Scaffold-40	22609839	G	A
Scaffold-40	22609848	G	T
Scaffold-40	22609858	A	G
Scaffold-40	22609873	C	T
Scaffold-40	23116042	T	C
Scaffold-40	23116073	TTTCTTCT	TT
Scaffold-40	23116087	T	C
Scaffold-40	23286814	A	T
Scaffold-40	23286893	C	A
Scaffold-40	23286907	T	C
Scaffold-40	23286922	CATAC	TGTAG,CAAAG
Scaffold-40	23509487	G	A
Scaffold-40	23509510	G	A
Scaffold-40	23509512	C	A
Scaffold-40	23509518	C	T
Scaffold-40	23509548	A	G

Scaffold-40	23509574	G	A
Scaffold-40	23509585	T	C
Scaffold-40	23509599	T	C
Scaffold-40	23640554	T	A
Scaffold-40	23640593	G	A
Scaffold-40	23640651	G	A
Scaffold-40	23640661	CAAGGAAGGC	CATGGAAGGC,CAAGGCAGGC
Scaffold-40	23658519	T	A
Scaffold-40	23658524	TAAA	TAAC
Scaffold-40	23658538	C	A
Scaffold-40	23658549	C	T
Scaffold-40	23658555	CTA	CTT
Scaffold-40	23658578	G	A
Scaffold-40	23658585	GAAAA	GA
Scaffold-40	23658603	A	C
Scaffold-40	23658617	G	A
Scaffold-40	23658632	G	A
Scaffold-40	23675508	A	C
Scaffold-40	23675512	T	C
Scaffold-40	23675518	C	T
Scaffold-40	23739151	T	C
Scaffold-40	23739153	CATG	CATT,TATT
Scaffold-40	23739178	T	C
Scaffold-40	23739198	T	C
Scaffold-40	23739204	T	C
Scaffold-40	23739206	AGTGTGCTTAGTG	AG
Scaffold-40	23739229	C	T
Scaffold-40	23739239	G	T
Scaffold-40	23739254	T	C
Scaffold-40	23739261	C	G
Scaffold-40	23739265	A	T
Scaffold-40	23739273	A	T
Scaffold-40	23739284	G	A
Scaffold-40	23837952	G	A
Scaffold-40	23837966	G	T
Scaffold-40	23837995	G	A
Scaffold-40	23838001	C	T
Scaffold-40	23838041	C	A
Scaffold-40	23838073	T	A
Scaffold-40	23974525	C	T
Scaffold-40	23974546	G	A
Scaffold-40	23974570	T	C
Scaffold-40	24007305	A	G
Scaffold-40	24007310	G	T

Scaffold-40	24007380	G	A
Scaffold-40	24007402	CTTTTTG	CTTTTG
Scaffold-40	24196934	A	G
Scaffold-40	24196965	T	C
Scaffold-40	24197018	T	G
Scaffold-40	24197028	AG	GT
Scaffold-40	24197033	T	C
Scaffold-40	24197070	C	A
Scaffold-40	24197096	C	A
Scaffold-40	24197115	T	A
Scaffold-40	24197157	GAA	GA
Scaffold-40	24197192	AAAT	AAAA,TAAA
Scaffold-40	24197218	T	C
Scaffold-40	24228129	T	G
Scaffold-40	24228132	T	C
Scaffold-40	24228140	G	A
Scaffold-40	24228154	G	A
Scaffold-40	24228156	A	T
Scaffold-40	24228197	A	G
Scaffold-40	24315129	T	A
Scaffold-40	24315161	T	C
Scaffold-40	24315196	T	C
Scaffold-40	24315205	A	T
Scaffold-40	24315217	CAT	TAA
Scaffold-40	24409320	C	T
Scaffold-40	24409334	C	G
Scaffold-40	24409416	G	A
Scaffold-40	24409440	A	T
Scaffold-40	24409449	T	C
Scaffold-40	24781574	C	T
Scaffold-40	24781588	C	A
Scaffold-40	24781596	TAG	CAG,CAA
Scaffold-40	24781613	T	A
Scaffold-40	24781671	T	C
Scaffold-40	24795989	A	C
Scaffold-40	24796021	C	G
Scaffold-40	24796047	A	T
Scaffold-40	24796058	G	C
Scaffold-40	24796060	T	C
Scaffold-40	24796068	C	T
Scaffold-40	24796074	C	T
Scaffold-40	24796085	CGTAAG	TGTAAG,CCTAAG
Scaffold-40	25110582	A	G
Scaffold-40	25110591	C	G

Scaffold-40	25110605	A	G
Scaffold-40	25110706	T	C
Scaffold-40	25165305	A	T
Scaffold-40	25165341	G	A
Scaffold-40	25165342	C	A
Scaffold-40	25165371	C	T
Scaffold-40	25165377	T	A
Scaffold-40	25341861	G	A
Scaffold-40	25341870	TTC	TTT
Scaffold-40	25341924	G	A
Scaffold-40	25341944	A	G
Scaffold-40	25341957	T	C
Scaffold-40	25341994	C	T
Scaffold-40	25348507	G	C
Scaffold-40	25348520	G	A
Scaffold-40	25348556	A	G
Scaffold-40	25348578	CAA	CAC,CA
Scaffold-40	25348626	T	G
Scaffold-40	25348631	A	T
Scaffold-40	25348632	G	C
Scaffold-40	25381331	T	A
Scaffold-40	25381362	T	C
Scaffold-40	25381370	A	T
Scaffold-40	25755340	T	C
Scaffold-40	25755420	G	C
Scaffold-40	25755454	C	G
Scaffold-40	25755468	A	C
Scaffold-40	25945484	TGTTTGAAAT	TGTTTAAAAT
Scaffold-40	25945570	T	C
Scaffold-40	26223480	A	C
Scaffold-40	26223526	T	C
Scaffold-40	26379073	A	G
Scaffold-40	26379082	C	T
Scaffold-40	26379109	C	T
Scaffold-40	26379120	C	T
Scaffold-40	26379121	C	T
Scaffold-40	26379145	C	T
Scaffold-40	26379184	G	A
Scaffold-40	26379188	G	A
Scaffold-40	26410293	C	T
Scaffold-40	26410303	G	A
Scaffold-40	26410329	T	C
Scaffold-40	26410353	G	A
Scaffold-40	26410366	C	A

Scaffold-40	26410368	T	C
Scaffold-40	26410413	C	G
Scaffold-40	26447634	GAA	GAAA
Scaffold-40	26447653	G	A
Scaffold-40	26771076	G	T
Scaffold-40	26771082	T	A
Scaffold-40	26771110	C	G
Scaffold-40	26771137	T	C
Scaffold-40	26771165	T	A
Scaffold-40	26876314	A	G
Scaffold-40	26876383	C	A
Scaffold-40	26876423	T	A
Scaffold-40	27131787	T	C
Scaffold-40	27216488	G	A
Scaffold-40	27216668	G	A
Scaffold-40	27216696	T	C
Scaffold-40	27216713	T	C
Scaffold-40	27216725	A	G
Scaffold-40	27279322	T	G
Scaffold-40	27279349	C	G
Scaffold-40	27279354	A	C
Scaffold-40	27279366	T	G
Scaffold-40	27279379	C	A
Scaffold-40	27345071	C	T
Scaffold-40	27345103	G	A
Scaffold-40	27345116	ATATTTTTTTA	ATATTTTTTA
Scaffold-40	27345141	G	A
Scaffold-40	27345154	T	C
Scaffold-40	27345165	A	G
Scaffold-40	27420002	A	G
Scaffold-40	27420030	A	C
Scaffold-40	27420059	G	C
Scaffold-40	27612096	AGA	AA
Scaffold-40	27612113	C	T
Scaffold-40	27612114	AAGGG	GAGGG,GAGGA
Scaffold-40	27612119	A	G
Scaffold-40	27612153	TAAAAAG	TAAAAAAG
Scaffold-40	27612171	C	T
Scaffold-40	27736157	G	C
Scaffold-40	27736158	T	C
Scaffold-40	27736186	GTTTGGCTTCTTA	GA
Scaffold-40	27736201	T	G
Scaffold-40	27736246	T	C
Scaffold-40	27736287	T	C

Scaffold-40 27820439 A C
Scaffold-40 27820450 CTTATCTTATTAT
Scaffold-40 27820489 C T
Scaffold-40 27820490 C A
Scaffold-40 27820500 C A
Scaffold-40 27820509 ATCC TTCT
Scaffold-40 27820558 G A
Scaffold-40 27945873 AAC GAC,GAG
Scaffold-40 27945912 TTTACC TC
Scaffold-40 28062186 G A
Scaffold-40 28125335 TTGTGTGTGTGTGTGTGT
TTGTGTGTGTGT,TTGTGTGTGTGTGT
Scaffold-40 28125394 T C
Scaffold-40 28125416 T C
Scaffold-40 28125432 T G
Scaffold-40 28125510 AAATAATAATAATC
AAATAATAATAATAATAATC,AGATAATAATAATC
Scaffold-40 28125526 T C
Scaffold-40 28125562 GCTTCTTT GCTGCTTT,GCTTT
Scaffold-40 28125582 C T
Scaffold-40 28125599 C T
Scaffold-40 28125606 T A
Scaffold-40 28168936 C T
Scaffold-40 28168944 C T
Scaffold-40 28168953 T A
Scaffold-40 28168962 G A
Scaffold-40 28169012 CACC CACAC,GACAC
Scaffold-40 28169027 C A
Scaffold-40 28186962 A G
Scaffold-40 28187000 T A
Scaffold-40 28642784 T C
Scaffold-40 28642812 AATC TTTT
Scaffold-40 28642856 CG CTG
Scaffold-40 28642864 C A
Scaffold-40 28743107 T A
Scaffold-40 28743108 AATCA AATCG,TATCG
Scaffold-40 28743123 TGAA TGAT,AGAG
Scaffold-40 28743127 CAC CAT
Scaffold-40 28743139 G T,A
Scaffold-40 28743143 TTTACTTTGC,GTTGT
Scaffold-40 28743165 G C
Scaffold-40 28743166 T A
Scaffold-40 28743169 GTTTTGCAT
GTTTTGCGT,GT,GTTTCGCAT,ATTTTGCAT

Scaffold-40	28743184	A	T
Scaffold-40	28743199	GTGTTTGTTT	GTGATTGTTT
Scaffold-40	28743229	T	A
Scaffold-40	28743245	C	T
Scaffold-40	28759193	A	G
Scaffold-40	28759220	C	T
Scaffold-40	28759236	GGT	GGC,AGC
Scaffold-40	28759259	AGATC	AGAGC,AGATGATC
Scaffold-40	28759298	C	T
Scaffold-40	28912634	G	A
Scaffold-40	28912647	C	T
Scaffold-40	28912660	G	C
Scaffold-40	28941245	C	T
Scaffold-40	28941255	C	T
Scaffold-40	28941297	A	G
Scaffold-40	28941329	C	T
Scaffold-40	28941344	A	T,C
Scaffold-40	28941362	T	C
Scaffold-40	28941401	C	T
Scaffold-40	29036052	G	T
Scaffold-40	29036088	A	G
Scaffold-40	29036097	T	A
Scaffold-40	29086898	A	G
Scaffold-40	29086943	C	T
Scaffold-40	29086969	AGTG	AG,AATG
Scaffold-40	29125584	G	A
Scaffold-40	29125592	T	A
Scaffold-40	29125596	G	A
Scaffold-40	29125601	A	G
Scaffold-40	29125633	A	G
Scaffold-40	29125635	A	C
Scaffold-40	29125649	T	C
Scaffold-40	29125653	ATT	AT
Scaffold-40	29125659	CAA	CTA
Scaffold-40	29125677	CA	GA,GC
Scaffold-40	29125679	G	A
Scaffold-40	29125685	T	C
Scaffold-40	29125688	AAAAAATA	CAAACATT
Scaffold-40	29125709	T	C
Scaffold-40	29156411	G	A
Scaffold-40	29310587	AGG	AG,AGT
Scaffold-40	29310596	C	G
Scaffold-40	29310602	C	A
Scaffold-40	29310614	A	T

Scaffold-40	29310661	T	A	
Scaffold-40	29310662	A	G	
Scaffold-40	29310672	C	T	
Scaffold-40	29310680	C	A	
Scaffold-40	29310699	C	A	
Scaffold-40	29529152	G	T	
Scaffold-40	29529188	C	T	
Scaffold-40	29529196	ACTTGTCA		AA
Scaffold-40	29529228	C	T	
Scaffold-40	29594880	G	A	
Scaffold-40	29594885	A	G	
Scaffold-40	29594890	C	G	
Scaffold-40	29594924	GC	TA	
Scaffold-40	29594934	T	G	
Scaffold-40	29594944	TCCAG		TG
Scaffold-40	29653946	T	C	
Scaffold-40	29653994	C	T	
Scaffold-40	29654057	T	G	
Scaffold-40	29770159	G	A	
Scaffold-40	29770178	A	G	
Scaffold-40	29770192	C	T	
Scaffold-40	29796842	CAC	CAAC	
Scaffold-40	29796858	G	A	
Scaffold-40	29796861	T	A	
Scaffold-40	29796894	GAAG	AACG	
Scaffold-40	29796925	G	A	
Scaffold-40	29796933	A	C	
Scaffold-40	29796936	A	G	
Scaffold-40	29823328	TAG	TG	
Scaffold-40	29823350	C	T	
Scaffold-40	29823352	T	C	
Scaffold-40	29823361	T	C	
Scaffold-40	29823418	A	T	
Scaffold-40	29823433	T	C	
Scaffold-40	30049931	T	A	
Scaffold-40	30049937	G	T	
Scaffold-40	30049956	G	T	
Scaffold-40	30049969	T	A	
Scaffold-40	30049974	T	C	
Scaffold-40	30050026	G	C	
Scaffold-40	30422090	A	G	
Scaffold-40	30422101	T	A	
Scaffold-40	30516102	TAACA		TAACG,CAACG,TAACT
Scaffold-40	30516118	G	A	

Scaffold-40	30516123	A	T
Scaffold-40	30516177	C	G
Scaffold-40	30516182	G	T
Scaffold-40	30516191	C	G
Scaffold-40	30666547	A	T
Scaffold-40	30666554	GG	GA
Scaffold-40	30666569	A	T
Scaffold-40	30666582	GTTG	GTTC,ATTC
Scaffold-40	30666613	A	G
Scaffold-40	30666623	A	T
Scaffold-40	30666627	GTT	GTTT
Scaffold-40	30666654	C	G
Scaffold-40	30666673	G	T
Scaffold-40	31098073	TGA	TGG,TGAA
Scaffold-40	31098080	A	G
Scaffold-40	31098086	AAGA	AAGAGA
Scaffold-40	31098144	G	A
Scaffold-40	31360145	G	A
Scaffold-40	31360165	G	A
Scaffold-40	31399811	A	C
Scaffold-40	31399821	G	A
Scaffold-40	31399858	T	A
Scaffold-40	31399873	A	G
Scaffold-40	31399890	C	A
Scaffold-40	31469419	C	T
Scaffold-40	31625090	GC	CT,GT
Scaffold-40	31625104	T	C
Scaffold-40	31625131	A	G
Scaffold-40	31625167	G	A
Scaffold-40	31625173	A	T
Scaffold-40	31625175	G	A
Scaffold-40	31625186	GGTAC	TGTAC
Scaffold-40	31625210	T	A
Scaffold-40	31923161	G	A
Scaffold-40	31923221	C	T
Scaffold-40	31923267	TGG	TGA,TG
Scaffold-40	31923270	A	T
Scaffold-40	31982002	C	T
Scaffold-40	31982015	A	T
Scaffold-40	31982032	AGAC	AGAA,TGAA
Scaffold-40	31982090	CAAA	CA
Scaffold-40	31982129	C	T
Scaffold-40	32072380	AAACG	AAACT
Scaffold-40	32072411	T	C

Scaffold-40	32072419	T	G	
Scaffold-40	32072423	G	T	
Scaffold-40	32072426	T	C	
Scaffold-40	32072427	G	A	
Scaffold-40	32072433	T	C	
Scaffold-40	32072436	A	G	
Scaffold-40	32072444	G	A	
Scaffold-40	32072471	G	A	
Scaffold-40	32072476	G	T	
Scaffold-40	32072487	T	G	
Scaffold-40	32092927	A	T	
Scaffold-40	32092948	T	G	
Scaffold-40	32092978	G	A	
Scaffold-40	32092980	C	G	
Scaffold-40	32093007	T	A	
Scaffold-40	32352109	TTCTAT		TT
Scaffold-40	32352131	G	T	
Scaffold-40	32352154	T	C	
Scaffold-40	32352178	T	G	
Scaffold-40	32352186	T	A	
Scaffold-40	32430517	C	T	
Scaffold-40	32430541	T	C	
Scaffold-40	32430547	T	A	
Scaffold-40	32572324	C	T	
Scaffold-40	32572337	A	G	
Scaffold-40	32572374	C	G	
Scaffold-40	32572450	C	T	
Scaffold-40	32601944	T	C	
Scaffold-40	32602004	G	C	
Scaffold-40	32645424	A	C	
Scaffold-40	32645435	T	G	
Scaffold-40	32800651	T	C	
Scaffold-40	32800687	C	T	
Scaffold-40	33081249	G	C	
Scaffold-40	33081283	G	T	
Scaffold-40	33081352	T	C	
Scaffold-40	33124355	T	G	
Scaffold-40	33384540	GTT	ATG	
Scaffold-40	33384562	ACTC	AC	
Scaffold-40	33384579	GTTTTTG		GTTTTG
Scaffold-40	33384634	C	A	
Scaffold-40	33980672	CTT	CT	
Scaffold-40	33980738	A	T	
Scaffold-40	33980748	T	C	

Scaffold-40	33980758	T	C	
Scaffold-40	33980788	C	T	
Scaffold-40	33980793	G	T	
Scaffold-40	34421954	A	G	
Scaffold-40	34421961	A	G	
Scaffold-40	34422010	A	C	
Scaffold-40	34422031	T	A	
Scaffold-40	34422036	GCAAG		GCAAC,GCACC,GCGAC,TCACC
Scaffold-40	34422047	T	C	
Scaffold-40	34422048	T	C	
Scaffold-40	34422078	A	G	
Scaffold-40	34422086	G	T	
Scaffold-40	34747232	C	G	
Scaffold-40	34747271	A	T	
Scaffold-40	34747281	G	A	
Scaffold-40	34923885	A	G	
Scaffold-40	34923968	A	G	
Scaffold-40	34923974	C	T	
Scaffold-40	34923986	C	T	
Scaffold-40	34923987	G	A	
Scaffold-40	35107765	C	T	
Scaffold-40	35107789	CGG	CG	
Scaffold-40	35107795	A	G	
Scaffold-40	35107819	CTTAAGATT		TTTTAGATT,CT
Scaffold-40	35107835	T	G	
Scaffold-40	35107840	T	C	
Scaffold-40	35107852	T	C	
Scaffold-40	35107870	C	T	
Scaffold-40	35107876	A	G	
Scaffold-40	35107897	A	G	
Scaffold-40	35135865	C	A	
Scaffold-40	35135881	T	A	
Scaffold-40	35135900	G	A	
Scaffold-40	35135905	G	T	
Scaffold-40	35253171	A	T	
Scaffold-40	35253188	A	G	
Scaffold-40	35253218	G	T	
Scaffold-40	35253223	C	A	
Scaffold-40	35253248	A	G	
Scaffold-40	35253270	T	C	
Scaffold-40	35253281	GGT	GGC	
Scaffold-40	35427545	C	T	
Scaffold-40	35427554	GACA	AACG	
Scaffold-40	35427619	A	G	

Scaffold-40	35655122	C	A
Scaffold-40	35932420	A	G
Scaffold-40	35932451	A	T
Scaffold-40	35932458	G	C
Scaffold-40	35932460	T	A
Scaffold-40	35932463	A	T
Scaffold-40	35932471	T	C
Scaffold-40	35932478	G	C
Scaffold-40	35932497	T	A
Scaffold-40	35932510	T	A
Scaffold-40	36058527	G	A
Scaffold-40	36058541	A	G
Scaffold-40	36058557	C	T
Scaffold-40	36058641	G	A
Scaffold-40	36330295	T	C
Scaffold-40	36330418	A	G
Scaffold-40	36539709	T	G
Scaffold-40	36539711	C	G
Scaffold-40	36539729	G	A
Scaffold-40	36539741	A	C
Scaffold-40	36539742	CAAAAAG	CAAAAG,CAGAAAG
Scaffold-40	36539749	G	C
Scaffold-40	36539758	A	G
Scaffold-40	36539771	A	G
Scaffold-40	36539772	C	A
Scaffold-40	36539793	G	A
Scaffold-40	36539802	G	A
Scaffold-40	36539816	G	A
Scaffold-40	36539832	G	C
Scaffold-40	36664791	GTGGGATTACAATCT	GT,GTGGGATTACAATCC
Scaffold-40	36664807	T	C
Scaffold-40	36723333	C	A
Scaffold-40	36723341	CCCC	CCCA
Scaffold-40	36723359	AA	AC
Scaffold-40	36723369	A	G
Scaffold-40	36723390	T	G
Scaffold-40	37338877	T	G
Scaffold-40	37338881	AA	AACATA
Scaffold-40	37338906	G	A
Scaffold-40	37338980	C	T
Scaffold-40	37606548	T	C
Scaffold-40	37755091	C	A
Scaffold-40	37755098	A	C
Scaffold-40	37755136	CA	CG,TG

Scaffold-40	37755146	G	A
Scaffold-40	37755156	G	T
Scaffold-40	37755159	G	T
Scaffold-40	37755187	A	G
Scaffold-40	37755193	A	G
Scaffold-40	37755196	GG	GA,TA
Scaffold-40	37755203	A	G
Scaffold-40	37755215	A	T
Scaffold-40	37755219	A	G
Scaffold-40	37804259	C	T
Scaffold-40	37804282	A	T
Scaffold-40	37804303	C	T
Scaffold-40	37804309	C	T
Scaffold-40	37804322	C	T
Scaffold-40	37804342	A	T
Scaffold-40	37804357	A	T
Scaffold-40	37824466	G	T
Scaffold-40	37824482	T	C
Scaffold-40	37824492	T	C
Scaffold-40	37824505	C	T
Scaffold-40	37824525	C	G
Scaffold-40	37824557	C	T
Scaffold-40	37936029	C	T
Scaffold-40	37936050	C	G
Scaffold-40	37936063	CAA	CA
Scaffold-40	37936119	G	A
Scaffold-40	37936126	G	T
Scaffold-40	37936136	C	A
Scaffold-40	38329998	C	G
Scaffold-40	38330004	C	G
Scaffold-40	38330025	AATCA	GATCA
Scaffold-40	38330033	G	A
Scaffold-40	38330038	C	G
Scaffold-40	38330062	A	G
Scaffold-40	38330080	G	T
Scaffold-40	38330115	G	T
Scaffold-40	38459499	GAAAAAAAT	GAAAAAAAAT,GAAAAAAAAAT
Scaffold-40	38459514	C	T
Scaffold-40	38459524	T	C
Scaffold-40	38459548	T	C
Scaffold-40	38459576	C	T
Scaffold-40	38459580	A	C
Scaffold-40	38459586	G	A
Scaffold-40	38459588	A	T

Scaffold-40	38687015	C	T
Scaffold-40	39340082	C	A
Scaffold-40	39340096	CT	CTCTCTAGT
Scaffold-40	39340106	ACT	ACG
Scaffold-40	39340148	A	G
Scaffold-40	39340180	A	T
Scaffold-40	39381972	A	G
Scaffold-40	39381983	T	A
Scaffold-40	39381986	C	T
Scaffold-40	39381992	C	A
Scaffold-40	39382004	T	C
Scaffold-40	39382024	A	C
Scaffold-40	39382029	T	A
Scaffold-40	39401183	A	G
Scaffold-40	39401190	C	T
Scaffold-40	39401195	A	T
Scaffold-40	39401239	ATTTTTTA	ATTTTTA,ATTTTTTG
Scaffold-40	39401261	A	G
Scaffold-40	39401267	G	A
Scaffold-40	39401269	A	T
Scaffold-40	39402036	G	A
Scaffold-40	39402063	G	T
Scaffold-40	39402069	ATCACGCTCGTATGT	ATCGCGCTCGTATGT,ATTACGCTCGTATGT,AT
Scaffold-40	39402085	CTA	TTG
Scaffold-40	39402088	C	T
Scaffold-40	39402094	ATAATTTAAG	
Scaffold-40	39402112	C	T
Scaffold-40	39402135	G	A
Scaffold-40	39402151	CTCCAC	GACCAAC
Scaffold-40	39402159	G	T
Scaffold-40	39501118	A	G
Scaffold-40	39501132	TTG	TTC,CTC
Scaffold-40	39501162	A	C
Scaffold-40	39501187	C	T
Scaffold-40	39501194	C	T
Scaffold-40	39600671	C	T
Scaffold-40	39658864	C	G
Scaffold-40	39658871	C	T
Scaffold-40	39658880	G	T
Scaffold-40	39658888	C	T
Scaffold-40	39658950	A	C
Scaffold-40	39658951	CAA	CA
Scaffold-40	39658956	T	G

Scaffold-40	39658957	C	A
Scaffold-40	39658970	G	A
Scaffold-40	39658977	A	C
Scaffold-40	39872288	T	A
Scaffold-40	39872292	G	A
Scaffold-40	40090117	T	G
Scaffold-40	40090119	C	G
Scaffold-40	40090135	G	T
Scaffold-40	40090155	ACAAACAAATC	AC
Scaffold-40	40090205	C	G
Scaffold-40	40090226	G	A
Scaffold-40	40242291	GTTACT	GT
Scaffold-40	40242304	G	A
Scaffold-40	40242344	GAAC	GC
Scaffold-40	40242367	T	C
Scaffold-40	40269666	C	A
Scaffold-40	40269706	C	A
Scaffold-40	40269717	AACT	GACT
Scaffold-40	40269725	G	T
Scaffold-40	40269726	G	A
Scaffold-40	40269734	A	G
Scaffold-40	40269739	A	T
Scaffold-40	40269741	A	G
Scaffold-40	40269764	G	A
Scaffold-40	40269788	C	T
Scaffold-40	40316469	ACAACT	ACAACG
Scaffold-40	40445347	T	A
Scaffold-40	40465702	A	G
Scaffold-40	40730591	C	G
Scaffold-40	40730633	T	C
Scaffold-40	40730646	AGG	AG
Scaffold-40	40730677	A	G
Scaffold-40	40730679	TGTACTGAAAAGTGT	TT,TTTACTGAAAAGTGT
Scaffold-40	40730695	A	G
Scaffold-40	40730700	T	C
Scaffold-40	40730708	A	G
Scaffold-40	40746982	T	C
Scaffold-40	40747008	G	A
Scaffold-40	40747033	GAAAAAAAAAG	GAAAAAAAAG,GAAAAAAAAAAG
Scaffold-40	40747047	A	C
Scaffold-40	40747054	T	G
Scaffold-40	40747089	T	A
Scaffold-40	40994231	A	G,T
Scaffold-40	40994250	A	G

Scaffold-40	40994284	GAA	GAAA	
Scaffold-40	40994290	C	G	
Scaffold-40	40994305	T	C	
Scaffold-40	40994333	C	A	
Scaffold-40	41021378	G	A	
Scaffold-40	41021407	GGTAC		CGTAT,GGTAT
Scaffold-40	41021460	C	G	
Scaffold-40	41104451	C	T	
Scaffold-40	41104467	C	G	
Scaffold-40	41104515	A	G	
Scaffold-40	41104517	C	T	
Scaffold-40	41104518	G	T	
Scaffold-40	41124362	G	A	
Scaffold-40	41124388	A	T	
Scaffold-40	41124392	TAAAAAAT		TAAAAAAAT
Scaffold-40	41124417	T	C	
Scaffold-40	41142605	A	G	
Scaffold-40	41142607	A	G	
Scaffold-40	41142613	T	C	
Scaffold-40	41142644	C	A	
Scaffold-40	41148318	C	A	
Scaffold-40	41148347	T	C	
Scaffold-40	41148400	A	C	
Scaffold-40	41148407	G	A	
Scaffold-40	41148415	C	T	
Scaffold-40	41148429	T	C	
Scaffold-40	41148496	G	C	
Scaffold-40	41148573	T	C	
Scaffold-40	41166994	GTACTGGAACTATGAC		
GC,GTACTGGAACTATATATGAC,GTACTGGAACAATGAC				
Scaffold-40	41167012	TA	TAGGGCA	
Scaffold-40	41167021	G	A	
Scaffold-40	41167051	G	A	
Scaffold-40	41167052	T	A	
Scaffold-40	41167053	GGTTA		GGTTGTTA
Scaffold-40	41167089	T	C	
Scaffold-40	41167091	GC	GCAACAAATC	
Scaffold-40	41167099	T	G	
Scaffold-40	41167104	C	A	
Scaffold-40	41167105	A	G	
Scaffold-40	41199987	T	G	
Scaffold-40	41199997	A	G	
Scaffold-40	41200009	C	T	
Scaffold-40	41200061	G	A	

Scaffold-40	41228227	CC	CTT	
Scaffold-40	41228266	A	G	
Scaffold-40	41228279	C	G	
Scaffold-40	41228283	GC	AT	
Scaffold-40	41266575	A	G	
Scaffold-40	41693151	G	A	
Scaffold-40	41693187	C	T	
Scaffold-40	41693211	G	T	
Scaffold-40	41693213	T	C	
Scaffold-40	41693257	A	T	
Scaffold-40	41786942	C	G	
Scaffold-40	41786974	T	C,A	
Scaffold-40	41817965	A	G	
Scaffold-40	42049365	C	T	
Scaffold-40	42049366	G	A	
Scaffold-40	42049376	ACT	AT,AGT	
Scaffold-40	42049380	A	G	
Scaffold-40	42049400	G	A	
Scaffold-40	42049405	ATCT	TTCT,TTCA	
Scaffold-40	42049427	C	T	
Scaffold-40	42049428	A	T	
Scaffold-40	42049434	TGG	TG	
Scaffold-40	42049439	G	A	
Scaffold-40	42049444	C	G	
Scaffold-40	42049448	TCC	TC	
Scaffold-40	42049457	G	A	
Scaffold-40	42049466	G	A	
Scaffold-40	42049467	TAGAG		CAGAG
Scaffold-40	42049481	G	A	
Scaffold-40	42288425	A	C	
Scaffold-40	42288450	G	A	
Scaffold-40	42288476	A	G	
Scaffold-40	42335472	G	A	
Scaffold-40	42335473	C	A	
Scaffold-40	42335492	T	C	
Scaffold-40	42335537	A	T	
Scaffold-40	42335552	C	T	
Scaffold-40	42335561	A	G	
Scaffold-40	42359983	T	G,C	
Scaffold-40	42360026	T	C	
Scaffold-40	42360052	G	A	
Scaffold-40	42360067	A	G	
Scaffold-40	42360082	C	T	
Scaffold-40	42360083	T	C	

Scaffold-40	42701597	A	G
Scaffold-40	42922132	G	C
Scaffold-40	42922156	G	T
Scaffold-40	42922185	C	G
Scaffold-40	42922188	A	C
Scaffold-40	42922220	CTTTTTC	CTTTTTT
Scaffold-40	42922227	TCTC	TTTC,TCTT
Scaffold-40	42965851	T	A
Scaffold-40	42965930	T	C
Scaffold-40	42965942	G	T
Scaffold-40	42965972	A	G
Scaffold-40	42965979	G	A
Scaffold-40	42993460	CCAAAAAAAAAG	CCAAAAAAAAG,CCAAAAAAAAAAAG,CCAAAAAAAG,ACAAAAAAAAAG
Scaffold-40	42993477	A	C
Scaffold-40	42993499	C	T
Scaffold-40	42993505	GT	AT,AC
Scaffold-40	42993528	ACG	ATG
Scaffold-40	43020537	T	A
Scaffold-40	43020540	G	A
Scaffold-40	43020581	G	T
Scaffold-40	43021914	T	C
Scaffold-40	43021949	A	G
Scaffold-40	43021974	G	A
Scaffold-40	43022002	G	T
Scaffold-40	43022025	T	C
Scaffold-40	43091359	C	T
Scaffold-40	43091371	G	A
Scaffold-40	43091404	C	T
Scaffold-40	43091421	C	A
Scaffold-40	43091459	G	T
Scaffold-40	43217271	GTG	TTA
Scaffold-40	43217290	G	A
Scaffold-40	43217305	TTCCGAG	TTCCGATCCGAG,TTCTGAG
Scaffold-40	43217326	T	C
Scaffold-40	43217333	TTGT	TT
Scaffold-40	43217362	T	A
Scaffold-40	43217364	T	A
Scaffold-40	43217367	A	G
Scaffold-40	43217380	G	C
Scaffold-40	43545895	G	C
Scaffold-40	43545916	C	G
Scaffold-40	43545929	A	T
Scaffold-40	43545969	T	C

Scaffold-40	43660668	T	C
Scaffold-40	43660678	A	G
Scaffold-40	43660685	G	T
Scaffold-40	43660696	T	C
Scaffold-40	43660764	T	A
Scaffold-40	43660768	T	C
Scaffold-40	43660784	CGG	CAG,CGA
Scaffold-40	43660787	GG	GC
Scaffold-40	43680965	C	T
Scaffold-40	43680967	C	T
Scaffold-40	43680989	A	G
Scaffold-40	43681014	G	T
Scaffold-40	43681071	T	A
Scaffold-40	43681083	T	A
Scaffold-40	43681554	T	C
Scaffold-40	43681593	A	C
Scaffold-40	43820463	G	A
Scaffold-40	43820469	A	G
Scaffold-40	43820475	G	A
Scaffold-40	43820516	C	T
Scaffold-40	43820523	G	A
Scaffold-40	43820543	C	G
Scaffold-40	43820577	A	G
Scaffold-40	43900378	G	A
Scaffold-40	43900383	A	G
Scaffold-40	43900421	C	G
Scaffold-40	43900422	A	T
Scaffold-40	43981746	G	A
Scaffold-40	43981783	G	C
Scaffold-40	43981794	ATT	CTC,ATC
Scaffold-40	43981813	C	T
Scaffold-40	43981819	G	A
Scaffold-40	43981824	A	G
Scaffold-40	43981825	G	A
Scaffold-40	43981836	C	A
Scaffold-40	43981846	A	G
Scaffold-40	43981847	G	T
Scaffold-40	43981853	G	A
Scaffold-40	43996637	T	C
Scaffold-40	43996688	TTATACAT	TT
Scaffold-40	44175658	T	G
Scaffold-40	44175683	T	G
Scaffold-40	44175695	C	T
Scaffold-40	44175728	GGC	CGC,CGT

Scaffold-40	44175731	C	T
Scaffold-40	44175736	G	C
Scaffold-40	44175754	CCTG	GCTG
Scaffold-40	44175766	T	A
Scaffold-40	44290649	G	A
Scaffold-40	44290673	G	A
Scaffold-40	44290686	G	A
Scaffold-40	44290698	G	T
Scaffold-40	44290754	GA	GAATCTA
Scaffold-40	44381229	A	G
Scaffold-40	44381238	A	T
Scaffold-40	44381243	G	T
Scaffold-40	44381268	ATACCTAC	AGAG,AGAC
Scaffold-40	44381283	AT	TA,TT
Scaffold-40	44467450	C	T
Scaffold-40	44467453	C	T
Scaffold-40	44467473	C	A
Scaffold-40	44467531	G	A
Scaffold-40	44467606	T	G
Scaffold-40	44467638	T	C
Scaffold-40	44467693	T	C
Scaffold-40	44542637	C	T
Scaffold-40	44542647	C	A
Scaffold-40	44542675	T	A
Scaffold-40	44542677	T	C
Scaffold-40	44542683	T	A
Scaffold-40	44823557	T	G
Scaffold-40	44823560	G	A
Scaffold-40	44823562	T	C
Scaffold-40	44823622	CATTTATTTA	CATTTATTTATTA,CATTTATTTATTTA
Scaffold-40	45162663	C	A
Scaffold-40	45162705	T	C
Scaffold-40	45291798	C	T
Scaffold-40	45461072	G	A
Scaffold-40	45461080	T	C
Scaffold-40	45461143	A	G
Scaffold-40	45461165	T	C
Scaffold-40	45461167	AAAGT	TAAGT,TAAGC
Scaffold-40	45461174	T	C
Scaffold-40	45461177	T	G
Scaffold-40	45461183	G	A
Scaffold-40	45471546	G	C
Scaffold-40	45471580	G	A
Scaffold-40	45471589	C	T

Scaffold-40	45576719	C	A
Scaffold-40	45711248	T	C
Scaffold-40	45711296	C	T
Scaffold-40	45711308	C	T
Scaffold-40	45711348	G	A
Scaffold-40	45711371	A	C
Scaffold-40	45711372	T	C
Scaffold-40	45917201	C	T
Scaffold-40	45917248	C	T
Scaffold-40	45917256	C	G
Scaffold-40	45917260	ATT	AT,ATC
Scaffold-40	45917264	G	A
Scaffold-40	45917265	T	C
Scaffold-40	45917281	A	T
Scaffold-40	45917285	T	G
Scaffold-40	46066606	GCG	CCG
Scaffold-40	46066612	GTT	GTC,TTC,ATC
Scaffold-40	46066616	G	A
Scaffold-40	46066655	A	G
Scaffold-40	46066671	CTT	CT
Scaffold-40	46066679	ACTTG	ACTTA,GCTTA
Scaffold-40	46330677	T	C
Scaffold-40	46330704	C	G
Scaffold-40	46344155	T	A
Scaffold-40	46344160	G	A
Scaffold-40	46344210	T	A
Scaffold-40	46503561	C	T
Scaffold-40	46503584	A	T
Scaffold-40	46503599	C	G
Scaffold-40	46503628	G	A
Scaffold-40	46503642	A	T
Scaffold-40	46503667	G	T
Scaffold-40	46503688	G	A
Scaffold-40	46503690	T	G
Scaffold-40	46560652	T	G
Scaffold-40	46560685	TGG	TG
Scaffold-40	46560735	T	G
Scaffold-40	46560765	T	G
Scaffold-40	46589778	T	G
Scaffold-40	46693613	A	G
Scaffold-40	46693676	A	G
Scaffold-40	46693718	C	T
Scaffold-40	46921337	C	T
Scaffold-40	46921340	G	A

Scaffold-40	46921343	C	A
Scaffold-40	46921375	T	A
Scaffold-40	46921392	A	G
Scaffold-40	46921405	A	T
Scaffold-40	46921410	A	G
Scaffold-40	46921456	T	A
Scaffold-40	47109834	A	G
Scaffold-40	47109845	G	A
Scaffold-40	47109852	A	T
Scaffold-40	47109859	CAGG	TAGG,TAGA
Scaffold-40	47109871	CCATCGTCATCGTC	CCATCATCATCGTC,CTATCGTCATCGTC
Scaffold-40	47109907	CCTC	CC
Scaffold-40	47109915	T	A
Scaffold-40	47109932	T	C
Scaffold-40	47109939	A	G
Scaffold-40	47109953	A	C
Scaffold-40	47203393	G	C
Scaffold-40	47203446	T	A
Scaffold-40	47203455	T	C
Scaffold-40	47203506	C	T
Scaffold-40	47374579	ACC	CCC,AC
Scaffold-40	47374586	T	C
Scaffold-40	47374676	T	C
Scaffold-40	47374680	GG	TT
Scaffold-40	47377362	A	G
Scaffold-40	47377378	A	C
Scaffold-40	47377393	C	G
Scaffold-40	47478511	A	G
Scaffold-40	47478524	C	T
Scaffold-40	47478530	A	C
Scaffold-40	47478531	T	A
Scaffold-40	47478556	A	G
Scaffold-40	47478557	AGGTA	AA
Scaffold-40	47478565	G	A
Scaffold-40	47478613	ACC	ACCC
Scaffold-40	47478621	CCACCCACCAC	CCACCCAC
Scaffold-40	47478639	A	G
Scaffold-40	47478671	T	C
Scaffold-40	47478673	T	C
Scaffold-40	47478708	A	C
Scaffold-40	47478733	TTGCT	ATGCC
Scaffold-40	47478747	G	C
Scaffold-40	47478769	CAAACA	CAAACG,TAAAGG

Scaffold-40	47674984	TTTGG	GTTGA
Scaffold-40	47674994	CTTGGAG	TTTGGAG
Scaffold-40	47675020	AGATCCCCCG	GGATCCCCCG
Scaffold-40	47675032	GT	AC
Scaffold-40	47675041	G	T
Scaffold-40	47675043	GTG	GCG,GCGACGGACTC,GCGACGTACTC
Scaffold-40	47675053	T	G
Scaffold-40	47675065	ACC	ATC,AC
Scaffold-40	47675089	GG	GGCAGTGCCG
Scaffold-40	47675092	A	G
Scaffold-40	47675097	G	T
Scaffold-40	47675099	T	C
Scaffold-40	47675101	GCG	GG
Scaffold-40	47675105	A	G
Scaffold-40	47675111	G	A
Scaffold-40	47719058	A	T
Scaffold-40	47719072	GAGAC	AAGAT
Scaffold-40	47719095	T	A
Scaffold-40	47719113	A	C
Scaffold-40	47719162	C	G
Scaffold-40	47719174	AA	TT
Scaffold-40	47719183	T	C
Scaffold-40	47988181	G	A
Scaffold-40	48099712	T	A
Scaffold-40	48099764	C	T
Scaffold-40	48099777	T	A
Scaffold-40	48099821	A	T
Scaffold-40	48099828	ATT	TTC
Scaffold-40	48147884	C	G
Scaffold-40	48147898	T	A
Scaffold-40	48147899	A	G
Scaffold-40	48147909	C	A
Scaffold-40	48147927	C	T
Scaffold-40	48148000	C	T
Scaffold-40	48148001	G	A
Scaffold-40	48510336	T	C
Scaffold-40	48510367	ATC	GTC
Scaffold-40	48510393	T	C
Scaffold-40	48510413	G	A
Scaffold-40	48510435	A	G
Scaffold-40	48510442	A	T
Scaffold-40	48513845	A	C
Scaffold-40	48513919	G	T
Scaffold-40	48513920	T	C

Scaffold-40	48513948	A	G
Scaffold-40	48538431	TCTG	CCTG,CCTC
Scaffold-40	48538444	A	G
Scaffold-40	48538459	C	T
Scaffold-40	48538470	A	G
Scaffold-40	48538496	C	T
Scaffold-40	48538507	A	C
Scaffold-40	48538518	T	C
Scaffold-40	48538526	GCAATT	CTGATA,GCGATA
Scaffold-40	48538538	C	T
Scaffold-40	48632875	A	G
Scaffold-40	48660958	A	G
Scaffold-40	48661018	G	A
Scaffold-40	48661020	G	T
Scaffold-40	48661025	G	A
Scaffold-40	48661047	C	T
Scaffold-40	49057821	A	G
Scaffold-40	49057828	GGAG	GAACAAAG,GAACAAAA
Scaffold-40	49057852	A	G
Scaffold-40	49057855	G	C
Scaffold-40	49057863	ATTTGGTAATT	ATTTGGCAATT
Scaffold-40	49057878	T	A
Scaffold-40	49057907	A	G
Scaffold-40	49057911	T	C
Scaffold-40	49095940	G	T
Scaffold-40	49095944	T	C
Scaffold-40	49095946	A	C
Scaffold-40	49095954	G	A
Scaffold-40	49095965	TCC	TC
Scaffold-40	49095976	TGA	GGA,GCA
Scaffold-40	49095985	TAA	TAAA
Scaffold-40	49096002	A	T
Scaffold-40	49096003	T	C
Scaffold-40	49096006	G	T
Scaffold-40	49096007	G	C
Scaffold-40	49096014	CTATA	CTATT,TTATT
Scaffold-40	49096023	CAAG	CAAT,AAAT
Scaffold-40	49096031	T	G
Scaffold-40	49113876	C	T
Scaffold-40	49113901	T	G
Scaffold-40	49113941	G	T
Scaffold-40	49113948	C	T
Scaffold-40	49318043	C	A
Scaffold-40	49318056	G	C,A

Scaffold-40	49318058		T	A
Scaffold-40	49318065		T	G
Scaffold-40	49318067		A	G
Scaffold-40	49318081		G	A
Scaffold-40	49318134		TCACCG	ACGCCA,CCACCG
Scaffold-40	49318150		C	T
Scaffold-40	49318155		C	G
Scaffold-40	49318168		A	T
Scaffold-40	49318176		G	A
Scaffold-40	49338202		G	A
Scaffold-40	49338210		A	C
Scaffold-40	49338255		ATG	ATA,TTA
Scaffold-40	49338268		A	G
Scaffold-40	49338272		A	G
Scaffold-40	49434495		A	T
Scaffold-40	49527836		CTT	ATT
Scaffold-40	49527871		A	T
Scaffold-40	49527881		G	T
Scaffold-40	49527926		G	A
Scaffold-40	49570686		C	T
Scaffold-40	49570708		CAA	CA
Scaffold-40	49799751		A	T
Scaffold-40	49799758		T	A
Scaffold-40	49799774		G	A
Scaffold-40	49799785		C	T
Scaffold-40	49799805		TTATATAT	TTAT
Scaffold-40	49799860		C	A,T
Scaffold-41	74521	G	A	
Scaffold-41	74548	G	A	
Scaffold-41	74563	T	C	
Scaffold-41	75068	T	A	
Scaffold-41	75079	G	A	
Scaffold-41	75085	C	T	
Scaffold-41	75119	T	C	
Scaffold-41	75147	A	T	
Scaffold-41	75202	G	T	
Scaffold-41	230492		T	C
Scaffold-41	230539		G	T
Scaffold-41	230598		G	C
Scaffold-41	445010		T	C
Scaffold-41	445084		T	C
Scaffold-41	445102		A	G
Scaffold-41	445130		CTTTGT	GTTTGGT,GTTTGT,GTTTGG
Scaffold-41	540961		C	A

Scaffold-41	540997	C	A
Scaffold-41	541045	G	A
Scaffold-41	541083	C	T
Scaffold-41	743003	ATTTTTG	ATTTTG
Scaffold-41	743010	T	A
Scaffold-41	743025	A	G
Scaffold-41	743070	G	C
Scaffold-41	743113	T	C
Scaffold-41	909496	T	C
Scaffold-41	909514	T	A
Scaffold-41	909520	C	A
Scaffold-41	909535	T	A
Scaffold-41	909552	T	C
Scaffold-41	909558	GG	GGTTTCATTTG
Scaffold-41	909565	TGA	TGGA
Scaffold-41	909601	T	C
Scaffold-41	909624	G	T
Scaffold-41	942484	T	A
Scaffold-41	942492	T	A
Scaffold-41	942522	A	T
Scaffold-41	942525	C	T
Scaffold-41	942541	A	G
Scaffold-41	942573	A	T
Scaffold-41	1172148	CAAC	TAAC,TAAT
Scaffold-41	1172161	CAGTT	TAGTT,TAGTC
Scaffold-41	1172168	G	T
Scaffold-41	1172217	C	T
Scaffold-41	1613320	C	T
Scaffold-41	1613329	C	T
Scaffold-41	1613344	G	A
Scaffold-41	1613385	G	A
Scaffold-41	1613404	CTCG	GTCA,TTCG,CTCA
Scaffold-41	1613425	C	T
Scaffold-41	2140043	C	A
Scaffold-41	2140070	G	A
Scaffold-41	2140119	T	C
Scaffold-41	2188442	T	A
Scaffold-41	2188449	G	A
Scaffold-41	2192841	A	G
Scaffold-41	2192868	A	G
Scaffold-41	2504292	T	C
Scaffold-41	2504375	A	C
Scaffold-41	2504386	T	C
Scaffold-41	2504390	T	C

Scaffold-41	2504418	G	T
Scaffold-41	2509260	C	T
Scaffold-41	2509266	C	T
Scaffold-41	2509267	T	A
Scaffold-41	2509277	C	G
Scaffold-41	2509289	C	A
Scaffold-41	2509295	CG	CA,TA
Scaffold-41	2509317	G	C
Scaffold-41	2509325	A	G
Scaffold-41	2509340	C	G
Scaffold-41	2509373	G	A,C
Scaffold-41	2509380	C	T
Scaffold-41	2509389	G	A
Scaffold-41	2509391	T	G
Scaffold-41	2650295	T	C
Scaffold-41	2650304	A	T
Scaffold-41	2650378	T	G
Scaffold-41	2660193	G	T
Scaffold-41	2660227	A	G
Scaffold-41	2717921	C	A
Scaffold-41	2717951	A	G
Scaffold-41	2717969	CTCA	CTCG,GTCA,GTCG
Scaffold-41	2717979	ACC	AC
Scaffold-41	2718002	G	C
Scaffold-41	2718014	C	T
Scaffold-41	2718020	C	A
Scaffold-41	2718033	GCT	ACT,ACA
Scaffold-41	2810621	T	C
Scaffold-41	2810642	A	G,T
Scaffold-41	2810644	C	A
Scaffold-41	2810697	A	G
Scaffold-41	3038532	A	C
Scaffold-41	3038538	A	T
Scaffold-41	3038541	C	T
Scaffold-41	3038582	C	T
Scaffold-41	3180480	C	T
Scaffold-41	3180530	C	G
Scaffold-41	3180538	A	C
Scaffold-41	3180547	A	C
Scaffold-41	3569068	A	G
Scaffold-41	3569083	C	A
Scaffold-41	3569085	C	T
Scaffold-41	3569086	G	A
Scaffold-41	3569089	C	T

Scaffold-41	3569118	CAAAAAAAG	CAAAAAAAAG,CAAAAAACG
Scaffold-41	3569137	T	C
Scaffold-41	3569176	A	T
Scaffold-41	3721155	A	C
Scaffold-41	3732301	G	A
Scaffold-41	3732308	G	A
Scaffold-41	3732312	GCCCCCT	CCCCTCT,GCCCCCC,GCCCCCCT
Scaffold-41	3732337	C	G
Scaffold-41	3732343	C	T
Scaffold-41	3732348	A	T
Scaffold-41	3732391	G	T
Scaffold-41	3732401	A	G
Scaffold-41	3732417	A	C
Scaffold-41	3732440	T	C
Scaffold-41	3732444	G	A
Scaffold-41	3732484	T	C
Scaffold-41	3732512	G	A
Scaffold-41	3732525	C	A
Scaffold-41	3732542	C	T
Scaffold-41	3847093	C	T
Scaffold-41	3847168	C	T
Scaffold-41	3847199	C	T
Scaffold-41	4015251	GGTG	GGAA,CGAA
Scaffold-41	4015289	T	A
Scaffold-41	4218298	C	T
Scaffold-41	4218399	C	T
Scaffold-41	4218697	G	A
Scaffold-41	4218756	C	T
Scaffold-41	4496125	T	C
Scaffold-41	4496132	G	T
Scaffold-41	4496152	C	T
Scaffold-41	4496203	T	G
Scaffold-41	4496212	TC	TA
Scaffold-41	4496239	G	T
Scaffold-41	4496248	T	G
Scaffold-41	4496253	C	T
Scaffold-41	4767734	AATTTTTTTTC	GATTTTTTTC
Scaffold-41	4799823	ACCAAATCAGCACAAAAGC	AC
Scaffold-41	4799901	G	A
Scaffold-41	4799912	G	A
Scaffold-41	4800529	C	T
Scaffold-41	4800545	A	C
Scaffold-41	4800574	C	T
Scaffold-41	4800696	C	T

Scaffold-41	4800722	G	A
Scaffold-41	4800723	A	T
Scaffold-41	4800774	T	A
Scaffold-41	4883413	A	T
Scaffold-41	4883424	TAGCTG	AAACCA,GAGCTG
Scaffold-41	4883430	A	G
Scaffold-41	4883437	T	C
Scaffold-41	4883446	G	A
Scaffold-41	4883449	C	T
Scaffold-41	4883456	C	T
Scaffold-41	4883463	G	A
Scaffold-41	4883469	T	G
Scaffold-41	4883481	A	C
Scaffold-41	4883486	C	T
Scaffold-41	4883495	C	A
Scaffold-41	4883510	GTA	GCA,GTG,TTG
Scaffold-41	4883513	TC	TT
Scaffold-41	4883517	CTT	CTTT
Scaffold-41	4883520	C	T
Scaffold-41	4883531	A	G
Scaffold-41	4883536	CTGTTGTTGTT	CTGTTGTCGTT,CTGTTGTCATT,CTGTTGTT,CTGCTGTTGTT
Scaffold-41	4917892	ACTG	ACTC,GCTC
Scaffold-41	4917896	G	A
Scaffold-41	4917909	G	T
Scaffold-41	4917919	CAAA	TAAG
Scaffold-41	4917931	G	C
Scaffold-41	4917979	TGACGATCCG	GCACATTCCC,TCACATTCCC,TGACGATCCC
Scaffold-41	4917989	AAGG	AAGC
Scaffold-41	5184810	CGAAGAAGAAGAAA	TGAAGAAGAAGAAA
Scaffold-41	5184830	T	A
Scaffold-41	5184831	T	C
Scaffold-41	5184852	T	C
Scaffold-41	5184909	C	G
Scaffold-41	5435494	G	T
Scaffold-41	5435497	C	T
Scaffold-41	5435512	C	G
Scaffold-41	5435522	A	G
Scaffold-41	5510928	A	G
Scaffold-41	5590843	G	A
Scaffold-41	5590857	T	C
Scaffold-41	5590889	G	A
Scaffold-41	5590902	T	C

Scaffold-41	5679086	G	A	
Scaffold-41	5679091	T	G	
Scaffold-41	5679092	AC	AG,GG	
Scaffold-41	5705608	C	G	
Scaffold-41	5705644	G	A	
Scaffold-41	5705654	T	C	
Scaffold-41	5705668	A	T	
Scaffold-41	5709263	G	A	
Scaffold-41	5709304	T	C	
Scaffold-41	5709318	C	T	
Scaffold-41	5768226	A	G	
Scaffold-41	6187394	G	T	
Scaffold-41	6187410	G	A	
Scaffold-41	6187440	G	A	
Scaffold-41	6187443	TCT	TT	
Scaffold-41	6187447	G	A	
Scaffold-41	6187449	TTCTCTCTCTCA		TTCTCTCTCA
Scaffold-41	6187467	A	C	
Scaffold-41	6187476	T	C	
Scaffold-41	6187505	G	A	
Scaffold-41	6187508	C	G	
Scaffold-41	6289553	G	T	
Scaffold-41	6289558	T	G	
Scaffold-41	6289562	G	A	
Scaffold-41	6289627	A	T	
Scaffold-41	6514754	T	C	
Scaffold-41	6514765	C	T	
Scaffold-41	6514830	T	C	
Scaffold-41	6723429	C	A	
Scaffold-41	6723431	CC	CG	
Scaffold-41	6723441	C	G	
Scaffold-41	6723465	CCCT	CCCA,TCCA	
Scaffold-41	6723534	G	A	
Scaffold-41	6884153	G	T	
Scaffold-41	6884236	GAAA	GA	
Scaffold-41	6970043	T	C	
Scaffold-41	6970061	A	G	
Scaffold-41	6970129	C	T	
Scaffold-41	6970140	A	T	
Scaffold-41	7006638	C	T	
Scaffold-41	7006641	A	G	
Scaffold-41	7040706	A	C	
Scaffold-41	7040723	A	T	
Scaffold-41	7040724	A	C	

Scaffold-41	7040746	G	C
Scaffold-41	7040793	T	A
Scaffold-41	7040801	A	G
Scaffold-41	7040827	A	T
Scaffold-41	7040835	G	T
Scaffold-41	7294386	A	G
Scaffold-41	7294395	G	A
Scaffold-41	7294410	T	G
Scaffold-41	7294420	C	T
Scaffold-41	7294437	A	T
Scaffold-41	7294475	G	A
Scaffold-41	7294489	T	C
Scaffold-41	7294490	C	T
Scaffold-41	7294496	G	A
Scaffold-41	7331438	C	T
Scaffold-41	7331444	CT	CTCAT
Scaffold-41	7331450	A	T
Scaffold-41	7331459	A	C,T
Scaffold-41	7331465	A	G
Scaffold-41	7331469	A	C
Scaffold-41	7331486	C	T
Scaffold-41	7331494	T	A
Scaffold-41	7331510	C	T
Scaffold-41	7331530	G	A
Scaffold-41	7485398	A	G
Scaffold-41	7485527	T	A
Scaffold-41	7565950	T	C
Scaffold-41	7565973	T	C
Scaffold-41	7566022	A	G
Scaffold-41	7605030	T	C
Scaffold-41	7605066	T	C
Scaffold-41	7605079	GATATATG	GATATG
Scaffold-41	7605089	CG	TC,CC
Scaffold-41	7605097	GG	AG,AC
Scaffold-41	7605118	A	G
Scaffold-41	7605120	G	A
Scaffold-41	7605133	A	T
Scaffold-41	8434949	T	C
Scaffold-41	8434978	T	C
Scaffold-41	8434985	TTTCT	TTTTT,TT,TTTGT,TTT
Scaffold-41	8437806	A	C
Scaffold-41	8437812	A	G
Scaffold-41	8437846	C	T
Scaffold-41	8437863	C	A

Scaffold-41	8437872	G	A
Scaffold-41	8437896	T	G
Scaffold-41	8437905	T	A
Scaffold-41	8758092	A	G
Scaffold-41	8758109	G	A
Scaffold-41	8758115	C	A
Scaffold-41	8758118	T	C
Scaffold-41	8758130	C	T
Scaffold-41	8758133	A	G
Scaffold-41	8758165	G	C
Scaffold-41	8758172	T	C
Scaffold-41	9019556	T	A
Scaffold-41	9019567	C	T
Scaffold-41	9071798	C	T
Scaffold-41	9071810	G	A
Scaffold-41	9071834	C	T
Scaffold-41	9071840	G	A
Scaffold-41	9071890	G	A
Scaffold-41	10487124	A	G
Scaffold-41	10487129	C	T
Scaffold-41	10580711	T	C
Scaffold-41	10580747	G	C
Scaffold-41	10580756	T	C
Scaffold-41	10580774	T	G
Scaffold-41	10715243	A	C
Scaffold-41	10715250	A	G
Scaffold-41	10715307	C	T
Scaffold-41	10715319	A	C
Scaffold-41	10715344	GC	AG
Scaffold-41	10715358	T	A
Scaffold-41	10715368	TG	AG,AA
Scaffold-41	10715370	C	T
Scaffold-41	10921784	GTG	GTA,ATA
Scaffold-41	10921802	AC	TG,AG
Scaffold-41	10921823	A	G
Scaffold-41	10921850	C	G
Scaffold-41	10921882	TCC	CCT
Scaffold-41	10979976	G	A
Scaffold-41	10979977	C	T
Scaffold-41	10980001	A	G
Scaffold-41	11199611	AGCGGTGGCGGTGGCGGTGGCGGTGA	AGCGGTGGTGGTGGCGGTGGCGGTGA,AGCGGTGGCGGTGGCGGTGA,AGCGGTGGCGGTGGCGGTGGCGGTGGCGGTGA
Scaffold-41	11199660	C	A

Scaffold-41	11199677	C	T
Scaffold-41	11199719	A	G
Scaffold-41	11199733	C	A
Scaffold-41	11259297	G	T
Scaffold-41	11259301	GC	AA
Scaffold-41	11259318	AAT	CAC,AGT
Scaffold-41	11259324	CGAAAACG	CGAAAACT,CGAACACT
Scaffold-41	11259338	TGAAG	GGAAA
Scaffold-41	11259344	G	T
Scaffold-41	11259364	G	A
Scaffold-41	11259373	C	A
Scaffold-41	11259382	T	C
Scaffold-41	11259396	C	T
Scaffold-41	11259413	A	G,T
Scaffold-41	11259428	A	G
Scaffold-41	11396648	A	T
Scaffold-41	11396657	C	G
Scaffold-41	11396734	A	G
Scaffold-41	11396737	G	A
Scaffold-41	11397245	A	G
Scaffold-41	11397255	A	T
Scaffold-41	11397271	C	T
Scaffold-41	11538785	A	T
Scaffold-41	11538789	C	T
Scaffold-41	11538848	A	G
Scaffold-41	11538859	G	A
Scaffold-41	11538893	C	T
Scaffold-41	11538897	TCT	TCG,CCG
Scaffold-41	11538911	C	T
Scaffold-41	11538913	C	T
Scaffold-41	11538914	G	A
Scaffold-41	11538922	T	G
Scaffold-41	11538927	G	A
Scaffold-41	11540966	T	C
Scaffold-41	11540972	A	G
Scaffold-41	11540977	T	C
Scaffold-41	11540989	AATCAAAGTA	AA,AATAAAAGTA,AATCAAAGCA
Scaffold-41	11541018	CAT	CAAT
Scaffold-41	11541024	T	C
Scaffold-41	11541029	A	T
Scaffold-41	11541034	T	C
Scaffold-41	11541071	C	T
Scaffold-41	11541092	G	T
Scaffold-41	11957872	C	T

Scaffold-41	11957875	C	T
Scaffold-41	11957877	A	G
Scaffold-41	11957879	C	G
Scaffold-41	11957893	T	A
Scaffold-41	12190099	A	T
Scaffold-41	12190114	G	A
Scaffold-41	12190120	C	T
Scaffold-41	12190126	A	T
Scaffold-41	12190134	A	G
Scaffold-41	12190170	G	A
Scaffold-41	12190177	G	A
Scaffold-41	12190189	C	T
Scaffold-41	12190195	G	A
Scaffold-41	12190201	A	G
Scaffold-41	12208371	C	A
Scaffold-41	12208384	C	T
Scaffold-41	12208406	C	A
Scaffold-41	12208433	T	C
Scaffold-41	12208440	G	C
Scaffold-41	12208450	G	C
Scaffold-41	12231609	C	T
Scaffold-41	12231632	A	T
Scaffold-41	12231639	C	T,A
Scaffold-41	12231662	G	A
Scaffold-41	12231699	A	T
Scaffold-41	12275858	C	G
Scaffold-41	12275920	G	T
Scaffold-41	12275942	C	G
Scaffold-41	12275989	C	T
Scaffold-41	12374507	T	A
Scaffold-41	12374518	C	A
Scaffold-41	12374531	T	G
Scaffold-41	12374539	CAACCAA	CAAACAAACAGACATA,CAAACAAACAGGCATA
Scaffold-41	12374551	C	T
Scaffold-41	12374577	C	T
Scaffold-41	12374588	G	A
Scaffold-41	12374598	T	C
Scaffold-41	12374601	C	A
Scaffold-41	12374605	T	G
Scaffold-41	12565972	G	A
Scaffold-41	12565995	G	A
Scaffold-41	12566022	A	C
Scaffold-41	12566046	C	T

Scaffold-41	12566066	G	A
Scaffold-41	12566073	G	A
Scaffold-41	12636083	C	A
Scaffold-41	12636093	G	A
Scaffold-41	12636154	A	T
Scaffold-41	12636169	A	C
Scaffold-41	12636188	C	T
Scaffold-41	12723193	T	C
Scaffold-41	12723272	C	A
Scaffold-41	12723281	T	C
Scaffold-41	12747720	T	C
Scaffold-41	12747735	T	G
Scaffold-41	12747745	ATT	AT
Scaffold-41	12747752	A	C
Scaffold-41	12747785	T	C
Scaffold-41	12747801	C	T
Scaffold-41	12774378	C	G
Scaffold-41	12774399	T	A
Scaffold-41	12774418	C	G
Scaffold-41	12774450	C	A
Scaffold-41	12774457	AGG	ACG,AG
Scaffold-41	12774470	ATATCT	ATATCTATCT
Scaffold-41	12774478	GCATAT	GTATAT,GT
Scaffold-41	12774495	C	T
Scaffold-41	12774509	T	G
Scaffold-41	12861941	T	C
Scaffold-41	12861956	TTATATATAGTTACATATAG,TTATATAG	
Scaffold-41	12861983	G	A
Scaffold-41	12861984	C	A
Scaffold-41	12862004	T	C
Scaffold-41	12862103	G	A
Scaffold-41	12862155	G	A
Scaffold-41	12862203	A	G
Scaffold-41	13250048	G	A
Scaffold-41	13250094	T	C
Scaffold-41	13298382	GGCGTCCGC	GGCGTCCGA
Scaffold-41	13298391	C	A
Scaffold-41	13298392	G	T
Scaffold-41	13298407	A	G
Scaffold-41	13298422	G	A
Scaffold-41	13298448	C	T
Scaffold-41	13298460	A	G
Scaffold-41	13298465	A	G
Scaffold-41	13298468	C	T

Scaffold-41	13410860	C	G
Scaffold-41	13410896	T	C
Scaffold-41	13410897	C	T
Scaffold-41	13410986	A	G
Scaffold-41	13843579	GTTTG	GTTTT
Scaffold-41	13843586	G	A
Scaffold-41	13843649	T	A
Scaffold-41	13843666	G	A,T
Scaffold-41	14000354	T	A
Scaffold-41	14000357	AG	TA
Scaffold-41	14005736	T	C
Scaffold-41	14005743	A	G
Scaffold-41	14005776	G	C
Scaffold-41	14005781	A	T
Scaffold-41	14005853	G	C
Scaffold-41	14111854	C	T
Scaffold-41	14111890	G	A
Scaffold-41	14125111	TTACAGA	TTACACACA,TTACACAGA,TTACAGAGA
Scaffold-41	14132374	T	A
Scaffold-41	14132384	T	G
Scaffold-41	14132391	ACTTAACTT	TCTTGACTG,ACTTTAACTT,TCTTGACTT
Scaffold-41	14132427	C	T
Scaffold-41	14132434	A	T
Scaffold-41	14132457	G	A
Scaffold-41	14132473	C	A
Scaffold-41	14132485	C	T
Scaffold-41	14132496	T	C
Scaffold-41	14132501	T	A
Scaffold-41	14182341	A	G
Scaffold-41	14182366	ACTAGC	GCTAGC
Scaffold-41	14602468	CC	TT
Scaffold-41	14602488	T	C
Scaffold-41	14602513	G	A
Scaffold-41	14602543	C	T
Scaffold-41	14602583	A	T
Scaffold-41	14602617	A	G
Scaffold-41	14602638	A	G
Scaffold-41	14602639	CAAGCTACTTAGAAGCTTAAA	CAAGCTACTTAGAAGCTCAAA,CAAGCTACTTAGAAGCTCTAA,CATGCTACTTAGAAGCTTAAA,CAAGCTATTTAGAAGCTTAAA
Scaffold-41	14602668	G	C
Scaffold-41	14602689	TT	TTTTTCAAT
Scaffold-41	14762896	A	G
Scaffold-41	14762900	C	T

Scaffold-41	14762931	A	G	
Scaffold-41	14953673	ATTTTTTTTTA		
ATTTTTTTTTAA,ATTTTTTTTA,ATTTTCTTTCA,ATTTTTTTA				
Scaffold-41	14953702	A	T	
Scaffold-41	14957039	T	C	
Scaffold-41	14957064	A	G	
Scaffold-41	14990085	A	G	
Scaffold-41	14990093	C	T	
Scaffold-41	14990095	CT	CGT,CC	
Scaffold-41	14990101	AAGAGTTCCCC		AG,AAGGGTTCCCC
Scaffold-41	14990118	ATGACAGATT		AT
Scaffold-41	14990133	C	T	
Scaffold-41	14990140	A	G	
Scaffold-41	14990147	A	T	
Scaffold-41	14990160	T	G	
Scaffold-41	14990172	A	G	
Scaffold-41	14990184	G	A	
Scaffold-41	14990193	T	C	
Scaffold-41	14990200	G	T	
Scaffold-41	14990215	G	A	
Scaffold-41	15126225	AAT	AAC	
Scaffold-41	15126255	CTA	CTG	
Scaffold-41	15126258	TTCCCG	TTCCCA,TTGCCA	
Scaffold-41	15126269	G	A	
Scaffold-41	15126270	C	A	
Scaffold-41	15126274	G	A	
Scaffold-41	15126280	TAAAAAAAAC		TAAAAAAAAAC
Scaffold-41	15126290	CGT	CAT,TGT	
Scaffold-41	15126295	C	G	
Scaffold-41	15126301	G	A	
Scaffold-41	15126307	C	T	
Scaffold-41	15126311	CTA	TTA,TTT	
Scaffold-41	15126325	C	T	
Scaffold-41	15433803	A	G	
Scaffold-41	15433828	T	A	
Scaffold-41	15544648	C	A	
Scaffold-41	15544669	G	A	
Scaffold-41	15544687	C	T	
Scaffold-41	15544723	G	C	
Scaffold-41	15712500	TCC	TC	
Scaffold-41	15712512	C	T	
Scaffold-41	15712529	C	T	
Scaffold-41	15712540	G	T	
Scaffold-41	15712580	T	C	

Scaffold-41	15712583	T	C
Scaffold-41	15712610	T	C
Scaffold-41	15712613	A	T
Scaffold-41	15849119	T	G
Scaffold-41	15849133	TA	TG,TGT
Scaffold-41	15902286	C	T
Scaffold-41	16420545	G	A
Scaffold-41	16420590	C	A
Scaffold-41	16420609	C	A
Scaffold-41	16565515	ATTTTTA	ATTTAAA,ATTTAA,ATTTA
Scaffold-41	16565531	C	G
Scaffold-41	16565594	G	A
Scaffold-41	16565609	CTT	CT
Scaffold-41	16565615	A	T
Scaffold-41	16565623	A	G
Scaffold-41	16641789	CGC	AGT,CGT
Scaffold-41	16641858	C	T
Scaffold-41	16641878	TG	CG,CT,CA
Scaffold-41	16641887	GG	GT,TT
Scaffold-41	16641899	T	C
Scaffold-41	16641902	A	T
Scaffold-41	16641904	C	A
Scaffold-41	16690972	A	C
Scaffold-41	16690983	T	C
Scaffold-41	16690984	T	C
Scaffold-41	16691003	C	T,G
Scaffold-41	16691567	G	T
Scaffold-41	16691584	G	A
Scaffold-41	16691635	T	G
Scaffold-41	16691681	A	C
Scaffold-41	16691690	G	A
Scaffold-41	16856699	A	C
Scaffold-41	16959720	G	A
Scaffold-41	16959752	G	C
Scaffold-41	16959755	T	G
Scaffold-41	16959774	G	A
Scaffold-41	16959784	CTGATGGCG	TTGATGGCG,TTGATAGCG,TTTATAGCG,TTTATAGCA
Scaffold-41	16959793	T	C
Scaffold-41	16959799	G	A
Scaffold-41	16959810	G	A
Scaffold-41	16959818	C	T
Scaffold-41	16959819	G	C,A
Scaffold-41	16959833	C	T

Scaffold-41	16959834	G	A	
Scaffold-41	16959846	G	A,C	
Scaffold-41	17039416	T	C	
Scaffold-41	17039485	T	C	
Scaffold-41	17039495	T	C	
Scaffold-41	17039506	A	G	
Scaffold-41	17141342	G	A	
Scaffold-41	17141358	T	A	
Scaffold-41	17308500	G	A	
Scaffold-41	17312514	A	G	
Scaffold-41	17312623	C	A	
Scaffold-41	17350183	G	A	
Scaffold-41	17350184	A	G	
Scaffold-41	17350248	A	T	
Scaffold-41	17350261	G	A	
Scaffold-41	17350279	T	G	
Scaffold-41	17350319	A	G	
Scaffold-41	17350339	G	A	
Scaffold-41	17350340	G	C	
Scaffold-41	17350366	G	A	
Scaffold-41	17350371	G	A	
Scaffold-41	17350425	G	T	
Scaffold-41	17350428	A	G	
Scaffold-41	17351005	T	G	
Scaffold-41	17351009	T	C	
Scaffold-41	17351033	GAACA		GAACC
Scaffold-41	17479516	C	T	
Scaffold-41	17479554	G	T	
Scaffold-41	17479586	T	C	
Scaffold-41	17479600	G	A	
Scaffold-41	17479605	C	T	
Scaffold-41	17479614	CTTT	CT	
Scaffold-41	17479634	A	G	
Scaffold-41	17479640	TCA	TCCA	
Scaffold-41	17479645	A	G	
Scaffold-41	17594028	T	A,C	
Scaffold-41	17710218	A	G	
Scaffold-41	17710276	A	G	
Scaffold-41	17710277	C	A	
Scaffold-41	17710289	T	A	
Scaffold-41	17710326	A	G	
Scaffold-41	17710341	T	G	
Scaffold-41	17721447	G	C	
Scaffold-41	17796044	A	G	

Scaffold-41	17796070	T	C
Scaffold-41	17796102	C	T
Scaffold-41	17796154	A	G
Scaffold-41	17820680	C	A
Scaffold-41	17820718	T	A
Scaffold-41	17820727	A	G
Scaffold-41	17820734	C	T
Scaffold-41	17845833	G	T
Scaffold-41	17845845	T	C
Scaffold-41	18004163	G	A
Scaffold-41	18316081	C	T
Scaffold-41	18464910	T	C
Scaffold-41	18702581	GATATATATATATG	GATATATATATATATG
Scaffold-41	18702629	G	T
Scaffold-41	18702640	TAA	TA
Scaffold-41	18832581	A	G
Scaffold-41	18832600	G	T
Scaffold-41	18832605	C	G
Scaffold-41	18832618	T	G
Scaffold-41	18832689	A	G
Scaffold-41	18872810	T	C
Scaffold-41	18872831	T	G
Scaffold-41	18872856	G	A
Scaffold-41	18872895	GT	AC
Scaffold-41	18936737	G	C
Scaffold-41	18936787	T	C
Scaffold-41	18936819	T	G
Scaffold-41	18936829	T	C
Scaffold-41	19063372	G	T
Scaffold-41	19063427	G	C
Scaffold-41	19063478	G	T
Scaffold-41	19243765	C	G
Scaffold-41	19243804	C	T
Scaffold-41	19243819	C	T
Scaffold-41	19243868	A	G
Scaffold-41	19262023	T	G
Scaffold-41	19262028	C	T
Scaffold-41	19262034	A	G
Scaffold-41	19262053	T	C
Scaffold-41	19269757	G	C
Scaffold-41	19269803	T	A
Scaffold-41	19362350	T	A
Scaffold-41	19362356	T	A
Scaffold-41	19362376	A	G

Scaffold-41	19362379	A	T
Scaffold-41	19362421	G	C
Scaffold-41	19362429	C	A
Scaffold-41	19362455	T	C
Scaffold-41	19362470	C	T
Scaffold-41	19457078	T	A
Scaffold-41	19457120	G	A
Scaffold-41	19457156	A	C
Scaffold-41	19461034	T	C
Scaffold-41	19461056	A	G
Scaffold-41	19461070	A	G
Scaffold-41	19461082	A	T
Scaffold-41	19461124	G	A
Scaffold-41	19461127	C	T,A
Scaffold-41	19461130	ACACC	AC
Scaffold-41	19489581	G	C
Scaffold-41	19489589	C	T
Scaffold-41	19489610	T	C
Scaffold-41	19679729	T	G
Scaffold-41	19679754	A	G
Scaffold-41	19679767	G	A
Scaffold-41	19963439	A	T
Scaffold-41	19963463	C	T
Scaffold-41	19963472	T	C
Scaffold-41	19963491	C	G
Scaffold-41	20105367	C	A
Scaffold-41	20105403	T	A
Scaffold-41	20105445	G	C
Scaffold-41	20132478	A	T
Scaffold-41	20132484	C	T
Scaffold-41	20147716	G	A
Scaffold-41	20147717	A	G
Scaffold-41	20147745	T	G
Scaffold-41	20147757	G	A
Scaffold-41	20147760	C	A
Scaffold-41	20147773	A	G
Scaffold-41	20156126	A	G
Scaffold-41	20156130	GAAAAAAAAG	GAAAAAAAAAG
Scaffold-41	20156140	C	G
Scaffold-41	20269685	A	T
Scaffold-41	20269689	T	C
Scaffold-41	20269696	TCCGT	TCTGT,TCCGG
Scaffold-41	20314800	C	T
Scaffold-41	20314811	TCA	CCG,CCT

Scaffold-41	20314829	A	G
Scaffold-41	20503078	A	T
Scaffold-41	20503120	CTGGCCTGCTGCCATGTTGGATTTTT	CTTGCCTGCTGCCATGTTGGATTTTT,CTGGCTTGCTGCCATGTTGGATTTTT
Scaffold-41	20503159	C	T
Scaffold-41	20503173	G	A
Scaffold-41	20503180	A	G
Scaffold-41	20503188	A	G
Scaffold-41	20645003	T	G
Scaffold-41	20645093	G	C
Scaffold-41	20702436	C	A
Scaffold-41	20702489	T	C
Scaffold-41	20702542	T	C
Scaffold-41	20827855	T	G
Scaffold-41	20827889	A	T
Scaffold-41	20827909	A	T
Scaffold-41	20827915	C	G
Scaffold-41	21005106	T	A
Scaffold-41	21005115	A	G
Scaffold-41	21005162	C	G
Scaffold-41	21027829	GTA	GA
Scaffold-41	21119327	T	C
Scaffold-41	21119355	ACC	ACT,ACG
Scaffold-41	21119428	T	C
Scaffold-41	21119444	C	T
Scaffold-41	21119447	C	T
Scaffold-41	21119484	G	A
Scaffold-41	21119489	G	A
Scaffold-41	21119507	A	G
Scaffold-41	21119550	C	T
Scaffold-41	21119580	CGCAA	TGCAA,CACAA
Scaffold-41	21386797	A	G
Scaffold-41	21386873	A	G
Scaffold-41	21386891	A	G
Scaffold-41	21430123	CAA	CAAA
Scaffold-41	21430127	ATA	AAA,AA
Scaffold-41	21430135	TAAAAAAAT	TTAAAAAAT
Scaffold-41	21430231	TCCACCAT	TCCAGCAT
Scaffold-41	21687079	GCCTGTGTTTTTA	GA,GCCTGGGTTTTTA
Scaffold-41	21687343	C	T
Scaffold-41	21687368	T	G
Scaffold-41	21687389	A	T
Scaffold-41	21789275	T	C
Scaffold-41	21789299	A	G

Scaffold-41	21789400	T	G
Scaffold-41	21918263	A	T
Scaffold-41	21918321	TAACAACAACAACAACAACAACA	TAAAAACAACAACAACAACAACA
Scaffold-41	21918357	G	A
Scaffold-41	22385692	T	A
Scaffold-41	22385731	TGGTG	CGGTG,TGATG
Scaffold-41	22404430	TCTTTCT	TT
Scaffold-41	22404464	TATAG	AATAC
Scaffold-41	22404473	C	T
Scaffold-41	22404479	G	A
Scaffold-41	22404532	C	T
Scaffold-41	22404536	C	G
Scaffold-41	22404573	C	T
Scaffold-41	22404576	ACA	GCA
Scaffold-41	22404579	A	T
Scaffold-41	22404702	GTGACC	GTGAC
Scaffold-41	22404719	A	G
Scaffold-41	22404723	T	C
Scaffold-41	22481173	A	C
Scaffold-41	22481190	A	G
Scaffold-41	22481193	C	T
Scaffold-41	22481197	T	A
Scaffold-41	22481209	A	T
Scaffold-41	22481215	C	G
Scaffold-41	22481224	C	T
Scaffold-41	22481242	ATTATGATGTT	AT
Scaffold-41	22481257	AT	ATGCT
Scaffold-41	22481264	TC	TGACC,CC,TGATGACC,TT
Scaffold-41	22512444	TAC	TAT
Scaffold-41	22512492	C	T
Scaffold-41	22512534	TTTGTTGTTGTTGA	TTTGTTGTTGA
Scaffold-41	22513280	C	T
Scaffold-41	22537646	T	A
Scaffold-41	22537658	C	T
Scaffold-41	22537684	TAGGC	CAGGC,CAGGG,GAGGC
Scaffold-41	22537705	T	C
Scaffold-41	22537724	G	T,A,C
Scaffold-41	22884805	C	T
Scaffold-41	22884829	ATA	AGA,AG
Scaffold-41	22884840	TTTTC	TTTTT,CTTTT
Scaffold-41	22884845	CA	CG
Scaffold-41	22884860	G	T
Scaffold-41	22884868	C	T

Scaffold-41	22884873	T	A
Scaffold-41	22899573	CCCG	CCCA
Scaffold-41	22899663	G	A
Scaffold-41	22902898	A	G
Scaffold-41	22902960	AATATTA	AA
Scaffold-41	22904158	A	G
Scaffold-41	22904256	C	G
Scaffold-41	22904273	C	T
Scaffold-41	22912822	T	C
Scaffold-41	22912888	A	G
Scaffold-41	22912904	C	A
Scaffold-41	22912922	A	G
Scaffold-41	22990295	A	T
Scaffold-41	23277819	A	C
Scaffold-41	23277922	A	G
Scaffold-41	23316778	G	C
Scaffold-41	23316820	A	T
Scaffold-41	23316907	A	G
Scaffold-41	23369778	T	G
Scaffold-41	23369793	CAAA	TAAA,TAAT
Scaffold-41	23369817	A	G
Scaffold-41	23369837	A	T
Scaffold-41	23369853	A	T
Scaffold-41	23382485	ATGT	AT
Scaffold-41	23456289	C	T
Scaffold-41	23456306	G	A
Scaffold-41	23456345	T	G
Scaffold-41	23456681	T	C
Scaffold-41	23498831	C	T
Scaffold-41	23498881	T	A
Scaffold-41	23598572	TG	TA
Scaffold-41	23598603	G	A,T
Scaffold-41	23598634	T	A
Scaffold-41	24025875	G	C
Scaffold-41	24025943	AACGA	AA
Scaffold-41	24026011	G	A
Scaffold-41	24026055	G	A
Scaffold-41	24026060	A	T
Scaffold-41	24038149	C	T
Scaffold-41	24038175	T	A
Scaffold-41	24170328	G	A
Scaffold-41	24170409	G	A
Scaffold-41	24439204	G	A
Scaffold-41	24439210	CGGT	TGGT

Scaffold-41	24439225	T	C
Scaffold-41	24439309	C	T
Scaffold-41	24578031	T	C
Scaffold-41	24578052	G	A
Scaffold-41	24578082	C	T
Scaffold-41	24598431	C	G
Scaffold-41	24598439	A	C
Scaffold-41	24598476	CGCC	AGCT,CGCT
Scaffold-41	24615804	G	A
Scaffold-41	24615828	TAACGAT	TAATGAC
Scaffold-41	24615857	A	G
Scaffold-41	24615873	A	G
Scaffold-41	24615935	A	G
Scaffold-41	24796254	T	C
Scaffold-41	24796288	G	A
Scaffold-41	24808623	C	T
Scaffold-41	24808635	A	G
Scaffold-41	24808659	AGCTGCC	TGCGGCT
Scaffold-41	24808677	GTCG	ATCA
Scaffold-41	24808686	TGCC	TGCA,TGCG,CGCA
Scaffold-41	24894214	C	A
Scaffold-41	24894223	A	G
Scaffold-41	24894228	T	C
Scaffold-41	24894246	A	C
Scaffold-41	24894282	A	G
Scaffold-41	24894316	CAC	CAT
Scaffold-41	25120518	C	G
Scaffold-41	25240790	C	T
Scaffold-41	25240850	G	T
Scaffold-41	25240890	T	A
Scaffold-41	25240911	AT	TA
Scaffold-41	25269631	A	T
Scaffold-41	25277293	C	T
Scaffold-41	25277336	G	A
Scaffold-41	25277399	T	C
Scaffold-41	25374621	T	C
Scaffold-41	25385925	A	G
Scaffold-41	25385970	G	A
Scaffold-41	25385972	T	C
Scaffold-41	25385998	TTG	ATG,ATC
Scaffold-41	25386005	ACAAGTTTTTTTATAGTACAATGCCCCA	ACAAGTTTTTTTATACTACAATGCCCCA,ACAAGTTTTTGTATACTACAATGCCCCA,ACAAGTTTTTTAATACTACAATGCCCCA,ACAAGTTTTTGTATGCTACAATGCCCCA,AAAAGTTTTTTTATACTACAATGCCCCA,ACAAGTTTTTTCATACTACAATGCCCCA

Scaffold-41	25433533	G	A
Scaffold-41	25433538	C	T
Scaffold-41	25433563	C	T
Scaffold-41	25433593	C	T
Scaffold-41	25433603	A	G
Scaffold-41	25449164	G	A
Scaffold-41	25449191	G	A
Scaffold-41	25449216	T	A
Scaffold-41	25557450	T	C
Scaffold-41	25557477	CCG	CCA,ACA
Scaffold-41	25557480	G	A
Scaffold-41	25557492	A	G
Scaffold-41	25557503	G	A
Scaffold-41	25557528	C	T
Scaffold-41	25557543	C	A
Scaffold-41	25557548	T	C
Scaffold-41	25557557	A	C
Scaffold-41	25557563	T	C
Scaffold-41	25575752	C	T
Scaffold-41	25575754	A	G
Scaffold-41	25575761	A	T
Scaffold-41	25575818	T	C
Scaffold-41	25575824	T	G
Scaffold-41	25575845	C	T
Scaffold-41	25645506	G	A
Scaffold-41	25645507	A	G
Scaffold-41	25645540	CAAAAAAC	CAAAC
Scaffold-41	25645552	AACA	AAAA
Scaffold-41	25645600	C	T
Scaffold-41	25830270	A	G
Scaffold-41	25830329	GTG	GTA
Scaffold-41	26240534	C	T
Scaffold-41	26430839	G	C
Scaffold-41	26430850	C	T
Scaffold-41	26430870	C	T
Scaffold-41	26430913	C	A
Scaffold-41	26430947	A	G
Scaffold-41	26525869	T	C
Scaffold-41	26525883	C	T
Scaffold-41	26525924	A	G
Scaffold-41	26525942	T	C
Scaffold-41	26525944	T	A
Scaffold-41	26525968	A	G
Scaffold-41	26525971	T	C

Scaffold-41	26582843	A	G	
Scaffold-41	26582902	A	G	
Scaffold-41	26582940	T	G	
Scaffold-41	26583412	C	T	
Scaffold-41	26583440	G	T	
Scaffold-41	26583481	T	C	
Scaffold-41	26583489	T	G	
Scaffold-41	26583514	A	G	
Scaffold-41	26583516	A	G	
Scaffold-41	26700547	G	C	
Scaffold-41	26700567	T	C	
Scaffold-41	26753646	T	A	
Scaffold-41	26753656	C	A	
Scaffold-41	26753678	A	G	
Scaffold-41	26753694	CATGA		GATGG
Scaffold-41	26753710	AA	ACA	
Scaffold-41	26753721	T	C	
Scaffold-41	26753744	T	C	
Scaffold-41	26753753	C	T	
Scaffold-41	26753757	A	C	
Scaffold-41	26817945	A	T	
Scaffold-41	26817955	G	C	
Scaffold-41	26818048	T	C	
Scaffold-41	26818071	C	A	
Scaffold-41	26845232	A	G	
Scaffold-41	26845253	G	A	
Scaffold-41	26845280	A	G	
Scaffold-41	26845336	G	A	
Scaffold-41	26845354	T	C	
Scaffold-41	26871833	CTC	GTC	
Scaffold-41	26871847	G	A	
Scaffold-41	26871858	A	C	
Scaffold-41	26871868	G	T	
Scaffold-41	26871873	T	A	
Scaffold-41	26871884	A	G	
Scaffold-41	26871891	A	G	
Scaffold-41	26871900	C	T	
Scaffold-41	26871906	T	G	
Scaffold-41	27062677	C	A	
Scaffold-41	27232213	G	A	
Scaffold-41	27338615	A	G	
Scaffold-41	27338635	A	G	
Scaffold-41	27338653	CTTTGTATGAG		TTTCCTATAAA,TTTTCTATAAA
Scaffold-41	27338677	T	C	

Scaffold-41	27338696	A	G
Scaffold-41	27338720	A	G
Scaffold-41	27338731	T	A,G
Scaffold-41	27338752	G	A
Scaffold-41	27342518	T	A
Scaffold-41	27342536	T	C
Scaffold-41	27342547	G	A
Scaffold-41	27342592	TGT	TTT,TT
Scaffold-41	27342595	TTTTT	TTTAA,TTTTA
Scaffold-41	27342600	AA	TA,GA
Scaffold-41	27368051	A	C
Scaffold-41	27368062	G	A
Scaffold-41	27368066	A	G
Scaffold-41	27368096	C	T
Scaffold-41	27368135	C	G
Scaffold-41	27368137	T	A
Scaffold-41	27368154	T	C
Scaffold-41	27370111	C	T
Scaffold-41	27370154	A	T
Scaffold-41	27370171	C	T
Scaffold-41	27370192	G	A
Scaffold-41	27370197	A	T
Scaffold-41	27370204	G	C
Scaffold-41	27438171	C	T
Scaffold-41	27438229	A	G
Scaffold-41	27438238	T	A
Scaffold-41	27438254	G	C
Scaffold-41	27438274	C	G
Scaffold-41	27438287	T	C
Scaffold-41	27467428	A	T
Scaffold-41	27467455	G	A
Scaffold-41	27467458	G	C
Scaffold-41	27467482	T	A
Scaffold-41	27467509	C	T
Scaffold-41	27523314	C	G
Scaffold-41	27523400	G	T
Scaffold-41	27523404	A	T
Scaffold-41	27523405	C	T
Scaffold-41	27523408	G	A
Scaffold-41	27523426	GGTGC	GGTGGC,TGTGGC
Scaffold-41	27593824	T	C
Scaffold-41	27593838	G	A
Scaffold-41	27593852	CCAGG	ACAGT
Scaffold-41	27593916	A	T

Scaffold-41	27687818	G	A	
Scaffold-41	27687836	GGTC	GGAC	
Scaffold-41	27687840	TAG	TAA	
Scaffold-41	27687852	TT	CC	
Scaffold-41	27687861	ATTGA		CTTA,CTTGA
Scaffold-41	27687895	T	A	
Scaffold-41	27687909	A	G	
Scaffold-41	27706634	C	T	
Scaffold-41	27816341	G	T	
Scaffold-41	27816357	A	G	
Scaffold-41	27816374	G	A	
Scaffold-41	27822041	A	G	
Scaffold-41	27822062	G	A	
Scaffold-41	27822111	A	G	
Scaffold-41	27822113	C	T	
Scaffold-41	27822117	TCA	TCG	
Scaffold-41	27822139	C	A	
Scaffold-41	27836171	G	A	
Scaffold-41	27881381	ACACCACT		ACACT,ACACTACT
Scaffold-41	27881398	G	T	
Scaffold-41	27881455	A	G	
Scaffold-41	27881461	T	A,G	
Scaffold-41	28056489	C	A	
Scaffold-41	28099354	GCCG	GCCC,ACCC	
Scaffold-41	28232267	T	A	
Scaffold-41	28232275	C	A	
Scaffold-41	28232299	TCAAC		TG
Scaffold-41	28232312	A	C,G	
Scaffold-41	28232350	G	T	
Scaffold-41	28232361	G	T	
Scaffold-41	28458226	C	G	
Scaffold-41	28458233	A	C	
Scaffold-41	28458256	A	T	
Scaffold-41	28458270	A	T	
Scaffold-41	28458277	T	C	
Scaffold-41	28458307	C	T	
Scaffold-41	28458325	GAG	GAAT	
Scaffold-41	28458343	C	A,T	
Scaffold-41	28458347	G	A	
Scaffold-41	28458350	G	A,T	
Scaffold-41	28491076	T	A	
Scaffold-41	28491084	AGC	AC	
Scaffold-41	28491101	TACA	AGCG,AGCA	
Scaffold-41	28491112	A	T	

Scaffold-41	28491129	C	T	
Scaffold-41	28491149	T	G	
Scaffold-41	28588596	A	T	
Scaffold-41	28588608	T	C	
Scaffold-41	28588650	T	C	
Scaffold-41	28624095	A	G	
Scaffold-41	28624098	G	C	
Scaffold-41	28624117	C	T	
Scaffold-41	28624123	TG	TGCGTTTGCG	
Scaffold-41	28624125	T	C	
Scaffold-41	28624172	T	C	
Scaffold-41	28624174	TCG	TG	
Scaffold-41	28729146	C	G	
Scaffold-41	28729157	T	C	
Scaffold-41	28729190	A	G	
Scaffold-41	28729193	CA	TG	
Scaffold-41	28729207	T	G	
Scaffold-41	28729220	TTTGG		GTTGG,GTTGA
Scaffold-41	28729238	C	A	
Scaffold-41	28741663	T	A	
Scaffold-41	28741673	G	T	
Scaffold-41	28945745	T	C	
Scaffold-41	28993811	T	C	
Scaffold-41	28993862	C	G	
Scaffold-41	29060516	C	A	
Scaffold-41	29060557	A	C	
Scaffold-41	29060600	T	G	
Scaffold-41	29564154	C	T	
Scaffold-41	29564158	A	G	
Scaffold-41	29564159	GG	GTG	
Scaffold-41	29564174	C	G	
Scaffold-41	29564230	T	A	
Scaffold-41	29564241	G	A	
Scaffold-41	29564258	G	A	
Scaffold-41	29565939	TGTAA		AGTAA
Scaffold-41	29566001	G	A	
Scaffold-41	29566026	G	C	
Scaffold-41	29566029	C	T	
Scaffold-41	29566045	G	A	
Scaffold-41	29566054	G	T	
Scaffold-41	29577913	T	A	
Scaffold-41	29577941	T	A	
Scaffold-41	29577994	G	A	
Scaffold-41	29578010	GTCTA		GA

Scaffold-41	29578017	C	T	
Scaffold-41	29578029	ATG	CTG	
Scaffold-41	29588369	C	T	
Scaffold-41	29588400	T	C	
Scaffold-41	29588426	A	G	
Scaffold-41	29588444	T	C	
Scaffold-41	29588452	C	T	
Scaffold-41	29665193	C	T	
Scaffold-41	29665202	T	C	
Scaffold-41	29665215	T	C	
Scaffold-41	29832898	T	C	
Scaffold-41	29832925	G	A	
Scaffold-41	29832934	T	A	
Scaffold-41	29859261	G	C	
Scaffold-41	29859312	G	T	
Scaffold-41	29859320	GTTTC		ATTTT
Scaffold-41	29859337	G	C	
Scaffold-41	29859352	T	A	
Scaffold-41	29859378	T	C	
Scaffold-41	29890389	A	G	
Scaffold-41	29890470	G	A	
Scaffold-41	29890499	C	T	
Scaffold-41	29890501	T	C	
Scaffold-41	29987512	T	G	
Scaffold-41	29987521	T	A	
Scaffold-41	29987559	C	A	
Scaffold-41	29987589	G	A	
Scaffold-41	29987614	T	C	
Scaffold-41	29987626	G	A	
Scaffold-41	29987635	G	A	
Scaffold-41	30047493	T	A	
Scaffold-41	30058822	G	A	
Scaffold-41	30058837	G	A	
Scaffold-41	30058849	C	T	
Scaffold-41	30058855	C	T	
Scaffold-41	30058861	T	C	
Scaffold-41	30058862	C	T	
Scaffold-41	30058870	T	A	
Scaffold-41	30058881	C	T	
Scaffold-41	30058890	C	T	
Scaffold-41	30058916	C	G	
Scaffold-41	30058928	C	T	
Scaffold-41	30058937	G	A	
Scaffold-41	30058944	C	T	

Scaffold-41	30059995	C	A
Scaffold-41	30060002	T	A
Scaffold-41	30060004	ATGA	ATGT,GTGT
Scaffold-41	30060049	G	C
Scaffold-41	30060063	ACA	ACCA,GCA
Scaffold-41	30060075	AGCAGT	AGCACT
Scaffold-41	30060107	T	C
Scaffold-41	30060120	T	C
Scaffold-41	30060131	A	G
Scaffold-41	30187471	AATAC	ATTAC,CATAT
Scaffold-41	30187549	A	G
Scaffold-41	30187569	T	C
Scaffold-41	30187597	G	A
Scaffold-41	30187603	G	A
Scaffold-41	30470263	C	T
Scaffold-41	30470278	A	T
Scaffold-41	30470332	C	T
Scaffold-41	30470343	A	G
Scaffold-41	30595398	G	A
Scaffold-41	30631375	G	C
Scaffold-41	30631439	G	A
Scaffold-41	30668092	C	A
Scaffold-41	30724170	T	C
Scaffold-41	30724235	A	G
Scaffold-41	30724239	C	T
Scaffold-41	30724255	T	A
Scaffold-41	30724256	G	A
Scaffold-41	30724275	A	G
Scaffold-41	30724297	T	G
Scaffold-41	30724301	T	G
Scaffold-41	30724306	A	G
Scaffold-41	30724308	G	A
Scaffold-41	30881787	T	C
Scaffold-41	30881802	C	A
Scaffold-41	30881862	A	G
Scaffold-41	30881877	CTAGACG	GTAGACG,GTGGACA
Scaffold-41	30882984	A	G
Scaffold-41	30882994	A	G
Scaffold-41	30883004	T	G
Scaffold-41	30910167	T	C
Scaffold-41	31006443	G	A
Scaffold-41	31051439	G	C
Scaffold-41	31051445	A	T
Scaffold-41	31051491	T	A

Scaffold-41	31051523	T	A
Scaffold-41	31087191	A	T
Scaffold-41	31087213	G	A
Scaffold-41	31087242	G	T
Scaffold-41	31287028	G	A
Scaffold-41	31287032	G	C
Scaffold-41	31287053	GAAA	GAAAAA
Scaffold-41	31287057	C	T
Scaffold-41	31287069	T	C
Scaffold-41	31287072	TAA	TTA
Scaffold-41	31366598	A	T
Scaffold-41	31649759	A	G
Scaffold-41	31649783	G	A
Scaffold-41	31657049	C	A
Scaffold-41	31657064	G	A
Scaffold-41	31657074	TTC	CTT
Scaffold-41	31657143	A	G
Scaffold-41	31736512	G	A
Scaffold-41	31736553	T	C
Scaffold-41	31736573	T	A
Scaffold-41	31736577	C	T
Scaffold-41	31736580	TT	AC
Scaffold-41	31736587	T	C
Scaffold-41	31736636	G	A
Scaffold-41	31736641	C	T
Scaffold-41	31831337	AGA	AA
Scaffold-41	31831344	CC	TG,TC
Scaffold-41	31831380	C	A
Scaffold-41	31831388	T	A
Scaffold-41	31831401	GGCTAACT	AGCGAACC,GGCTAACC
Scaffold-41	31831432	C	T
Scaffold-41	31831446	C	T
Scaffold-41	31890230	G	T
Scaffold-41	31890238	G	C
Scaffold-41	31890242	C	A
Scaffold-41	31890257	G	A
Scaffold-41	31890266	G	A
Scaffold-41	31890292	TG	TA
Scaffold-41	31890294	TC	TA
Scaffold-41	31890314	C	T
Scaffold-41	31890316	T	A
Scaffold-41	31890329	T	C
Scaffold-41	31929024	A	T
Scaffold-41	31929040	ACGTTTTCGTGGAATTCACACAACCATTCACC	

			ACGTTTTAGTGGAATTCACACAACCATTCACC
Scaffold-41	31929077	GGGT	TGGT
Scaffold-41	31929102	A	C
Scaffold-41	32137758	G	C
Scaffold-41	32137765	C	T
Scaffold-41	32137778	C	T
Scaffold-41	32291817	T	A
Scaffold-41	32291900	A	G
Scaffold-41	32291921	T	G
Scaffold-41	32291925	A	C
Scaffold-41	32291932	T	C
Scaffold-41	32367024	TA	AT
Scaffold-41	32367082	C	T
Scaffold-41	32367085	A	T
Scaffold-41	32367106	T	C
Scaffold-41	32609340	T	C
Scaffold-41	32609393	C	G
Scaffold-41	32626149	G	C
Scaffold-41	32626172	CCATCATCATC	CCATCATC,CCATCATCATCATC,CCATCGTCATC
Scaffold-41	32703534	T	G
Scaffold-41	32703540	A	T
Scaffold-41	32703568	T	C
Scaffold-41	32703578	T	C
Scaffold-41	32703586	G	A
Scaffold-41	32719463	A	G
Scaffold-41	32719500	A	G
Scaffold-41	32719526	A	G
Scaffold-41	32785902	A	T
Scaffold-41	32785938	G	C
Scaffold-41	32785976	G	A
Scaffold-41	32786018	T	C
Scaffold-41	32828124	C	T
Scaffold-41	32828151	A	G
Scaffold-41	32828214	C	T
Scaffold-41	32829040	GCC	GC
Scaffold-41	32829107	A	T
Scaffold-41	32829112	C	T
Scaffold-41	32859069	C	A
Scaffold-41	32859086	T	C
Scaffold-41	32859089	G	T,C
Scaffold-41	32902779	C	T
Scaffold-41	32902823	G	A
Scaffold-41	32902836	C	T

Scaffold-41	32902837	A	G
Scaffold-41	32902878	GGTGTGTG	GGTGTG
Scaffold-41	32902905	G	C
Scaffold-41	32964852	G	A
Scaffold-41	32964936	T	A
Scaffold-41	33090203	T	A
Scaffold-41	33090263	C	T
Scaffold-41	33090302	C	A
Scaffold-41	33293907	T	C
Scaffold-41	33319323	G	T
Scaffold-41	33319351	C	A
Scaffold-41	33319356	A	G
Scaffold-41	33426168	G	A
Scaffold-41	33426229	T	C
Scaffold-41	33434133	G	A
Scaffold-41	33434142	A	T
Scaffold-41	33434176	G	T
Scaffold-41	33434194	G	A
Scaffold-41	33434203	G	A
Scaffold-41	33434220	C	T
Scaffold-41	33437222	G	A
Scaffold-41	33437243	G	T
Scaffold-41	33437246	T	C
Scaffold-41	33437255	A	T
Scaffold-41	33437258	G	T
Scaffold-41	33437297	A	G
Scaffold-41	33437502	C	T
Scaffold-41	33437531	C	T
Scaffold-41	33437533	C	T
Scaffold-41	33450532	T	C
Scaffold-41	33450551	A	C
Scaffold-41	33450573	C	T
Scaffold-41	33450575	ATA	AA
Scaffold-41	33450579	CTAAATG	CTAAATTAAATG,CTAACTG
Scaffold-41	33450603	G	A
Scaffold-41	33450630	G	C
Scaffold-41	33450723	C	T
Scaffold-41	33653941	T	C
Scaffold-41	33653996	T	G
Scaffold-41	33696594	T	C
Scaffold-41	33696637	T	G
Scaffold-41	33696728	C	A
Scaffold-41	33992287	T	C
Scaffold-41	33992290	GGGATAAAG	GG

Scaffold-41	33992366	G	A	
Scaffold-41	33992371	T	A	
Scaffold-41	34363923	C	G	
Scaffold-41	34420631	C	G	
Scaffold-41	34420636	C	A	
Scaffold-41	34420669	A	G	
Scaffold-41	34420685	C	T	
Scaffold-41	34420724	CGTC	TGTC	
Scaffold-41	34421753	ATT	ATC	
Scaffold-41	34421765	GT	AC	
Scaffold-41	34421768	A	G	
Scaffold-41	34421788	T	C	
Scaffold-41	34421795	G	A	
Scaffold-41	34421805	A	G	
Scaffold-41	34421812	T	A	
Scaffold-41	34421828	G	T	
Scaffold-41	34421839	T	C	
Scaffold-41	34421848	G	A	
Scaffold-41	34762386	CAAGAA		CATGAA
Scaffold-41	34762398	T	C	
Scaffold-41	34762439	CA	AG	
Scaffold-41	34762443	A	G	
Scaffold-41	34762452	C	T	
Scaffold-41	34762480	G	A	
Scaffold-41	34796650	T	C	
Scaffold-41	34796655	A	T	
Scaffold-41	34796746	C	T	
Scaffold-41	34873098	C	G	
Scaffold-41	34873125	T	C	
Scaffold-41	34873131	T	C	
Scaffold-41	34873140	C	T	
Scaffold-41	34873195	G	A	
Scaffold-41	34873409	G	A	
Scaffold-41	34873424	G	A	
Scaffold-41	34873427	G	A	
Scaffold-41	34873487	A	G	
Scaffold-41	34873493	A	G	
Scaffold-41	34873497	CAC	AAG	
Scaffold-41	34873570	C	A	
Scaffold-41	34873575	G	A	
Scaffold-41	34873576	T	C	
Scaffold-41	34873583	G	A	
Scaffold-41	34873610	C	T	
Scaffold-41	34873622	T	G	

Scaffold-41	34873628	TTTG	ATTG,ATTT
Scaffold-41	34873638	A	G
Scaffold-41	34956508	T	C
Scaffold-41	34956521	C	A
Scaffold-41	34956535	CAAAAAAAAC	CAAAAAAAAAC,CAAAAAAAC,CAAAAAAAACAAAAAAACAAAAAAC
Scaffold-41	34956550	A	G
Scaffold-41	34956552	CAA	CA
Scaffold-41	34956575	A	G
Scaffold-41	34956589	C	T
Scaffold-41	34956606	G	A
Scaffold-41	35001848	C	T
Scaffold-41	35001923	A	T
Scaffold-41	35149810	T	C
Scaffold-41	35149816	AA	AATA
Scaffold-41	35149823	C	G
Scaffold-41	35149828	T	G
Scaffold-41	35149829	A	G
Scaffold-41	35149900	C	T
Scaffold-41	35149921	C	G
Scaffold-41	35149925	T	A
Scaffold-41	35161468	A	G
Scaffold-41	35161580	G	T
Scaffold-41	35170984	C	T
Scaffold-41	35170986	A	G
Scaffold-41	35170989	TCCTAACCCTAACCCTAACC	TCCTAACCCTAACCCTAACCCTAACC,TCCTAATCCTAACCCTAACC,TCCTAACCCTAACC
Scaffold-41	35171024	T	G
Scaffold-41	35171055	T	G
Scaffold-41	35203380	C	T
Scaffold-41	35203386	C	G
Scaffold-41	35203416	G	C
Scaffold-41	35203417	G	A
Scaffold-41	35245286	G	A
Scaffold-41	35245359	G	C
Scaffold-41	35245376	GGG	AGA
Scaffold-41	35245399	T	A
Scaffold-41	35622421	T	C
Scaffold-41	35622458	T	A
Scaffold-41	35622486	T	C
Scaffold-41	35630320	C	T
Scaffold-41	35630325	A	C
Scaffold-41	35630347	G	A

Scaffold-41	35630428	C	G
Scaffold-41	35745626	T	A
Scaffold-41	35745634	G	A
Scaffold-41	35745666	C	T,A
Scaffold-41	35745697	T	A
Scaffold-41	35745703	C	T
Scaffold-41	35745729	C	T
Scaffold-41	35745740	C	G
Scaffold-41	35745745	ACTTCTTC	ACTTC
Scaffold-41	35937047	C	T
Scaffold-41	35937086	G	C
Scaffold-41	36003460	T	C
Scaffold-41	36043585	C	T
Scaffold-41	36043661	A	C
Scaffold-41	36043687	C	A
Scaffold-41	36043704	G	A
Scaffold-41	36060463	CTT	CTTT
Scaffold-41	36060513	TAAAAAAAAAAAAG	TACAAAAAAAAAG
Scaffold-41	36096034	C	T
Scaffold-41	36096125	T	C
Scaffold-41	36097964	A	G
Scaffold-41	36097970	G	A
Scaffold-41	36098024	A	G
Scaffold-41	36098042	C	G
Scaffold-41	36098069	G	C
Scaffold-41	36102138	T	A,G
Scaffold-41	36102157	C	T
Scaffold-41	36102159	A	G
Scaffold-41	36117530	C	T
Scaffold-41	36117563	A	G
Scaffold-41	36117586	G	C
Scaffold-41	36117589	A	G
Scaffold-41	36117640	A	G
Scaffold-41	36117738	G	A
Scaffold-41	36451047	A	G
Scaffold-41	36653664	A	C
Scaffold-41	36653686	G	C
Scaffold-41	36653736	C	T
Scaffold-41	36653745	G	T
Scaffold-41	36653769	CAACACATACTGTTTA	CAGCACATACTGTTTA,CAACACATACTGTTTAACACATACTGTTTA
Scaffold-41	36653790	C	T
Scaffold-41	36657363	A	G
Scaffold-41	36657395	CAA	CAAA

Scaffold-41	36657409	C	G
Scaffold-41	36657419	C	T
Scaffold-41	36732225	TAA	TA
Scaffold-41	36732232	T	C
Scaffold-41	36732239	G	A
Scaffold-41	36732246	C	T
Scaffold-41	36732277	A	C
Scaffold-41	36732309	G	A
Scaffold-41	36732311	G	C
Scaffold-41	36732321	G	T
Scaffold-41	36987796	G	A
Scaffold-41	36987814	T	G
Scaffold-41	36987835	C	T
Scaffold-41	36987856	G	A
Scaffold-41	36987892	C	A
Scaffold-41	36987896	A	G
Scaffold-41	37126846	T	C
Scaffold-41	37126860	T	C
Scaffold-41	37126864	CAAAAAAC	CAAAAAAAC
Scaffold-41	37126902	GTCTCAT	ATCTTAT,ATCTTAC
Scaffold-41	37126940	CA	GT
Scaffold-41	37126954	A	G
Scaffold-41	37126961	G	T
Scaffold-41	37710247	T	C
Scaffold-41	37710505	A	G
Scaffold-41	37710509	G	C
Scaffold-41	37710529	G	A
Scaffold-41	37710568	A	G
Scaffold-41	37754706	T	C
Scaffold-41	37754711	G	A
Scaffold-41	37754727	T	C
Scaffold-41	37754731	G	C
Scaffold-41	37754792	A	T
Scaffold-41	37754795	A	G
Scaffold-41	37841593	GCG	GCC
Scaffold-41	37841596	TCT	TCC
Scaffold-41	37841600	C	T
Scaffold-41	37841615	G	A
Scaffold-41	37841624	G	C
Scaffold-41	37841666	C	G
Scaffold-41	37860976	C	T
Scaffold-41	37861012	C	G
Scaffold-41	37861037	T	G
Scaffold-41	38041231	TAAAAAAAAAG	TAAAAAAAAAAG

Scaffold-41	38041244	CAT	TAC,TAT,TGC
Scaffold-41	38041278	G	T
Scaffold-41	38095694	A	C
Scaffold-41	38095709	A	G
Scaffold-41	38095754	C	A
Scaffold-41	38095763	A	G
Scaffold-41	38168385	G	A
Scaffold-41	38168389	G	A
Scaffold-41	38168407	GAAGAA	AAAGAA,ACAGAA
Scaffold-41	38168418	T	C
Scaffold-41	38168422	A	G
Scaffold-41	38168433	C	A
Scaffold-41	38168441	T	G
Scaffold-41	38168458	C	T
Scaffold-41	38168485	C	A
Scaffold-41	38168494	GGA	TGA
Scaffold-41	38168510	ATG	GTG
Scaffold-41	38199259	G	A
Scaffold-41	38199304	C	T
Scaffold-41	38201537	C	G
Scaffold-41	38201589	T	C
Scaffold-41	38201596	TGT	TTT,TGTT,TGTTT,TGTTTT
Scaffold-41	38201601	TT	TTTTGT,TCTTGT
Scaffold-41	38201678	C	T
Scaffold-41	38201698	G	C
Scaffold-41	38201710	A	C
Scaffold-41	38201714	T	C
Scaffold-41	38201780	C	T
Scaffold-41	38201782	CATCACCAAGCTCAATA	CATCATCAAGCTCAATA
Scaffold-41	38201814	T	C
Scaffold-41	38322036	A	T
Scaffold-41	38322068	T	C
Scaffold-41	38322080	G	A
Scaffold-41	38322118	T	G
Scaffold-41	38322129	C	T
Scaffold-41	38579286	T	C,G
Scaffold-41	38666774	T	G
Scaffold-41	38666782	C	T
Scaffold-41	38748709	C	G
Scaffold-41	38748800	C	A
Scaffold-41	38984143	A	T
Scaffold-41	38984156	C	T
Scaffold-41	38984161	G	A
Scaffold-41	38984198	T	C

Scaffold-41	38984204	T	C
Scaffold-41	39076928	TAA	TA
Scaffold-41	39076933	A	G
Scaffold-41	39076947	CTAAACAACTAAT	CTATACAACTAAT,CT
Scaffold-41	39076989	G	A
Scaffold-41	39077007	T	G
Scaffold-41	39103347	T	C
Scaffold-41	39103355	AAAGAAGAAGT	AAAGAAGT,AAAGAAGTAAGT
Scaffold-41	39103407	C	G
Scaffold-41	39103436	C	T
Scaffold-41	39167215	A	G
Scaffold-41	39167235	T	C
Scaffold-41	39462167	C	T
Scaffold-41	39462197	A	G
Scaffold-41	39550487	T	C
Scaffold-41	39550497	C	T
Scaffold-41	39550503	T	C
Scaffold-41	39550506	AAA	CAA
Scaffold-41	39550520	GGAATA	CGAATA
Scaffold-41	39550529	TAGGC	TAGGG
Scaffold-41	39550564	T	C
Scaffold-41	39550581	C	T
Scaffold-41	39550620	G	T
Scaffold-41	39550649	AATTCA	AA
Scaffold-41	39550716	C	T
Scaffold-41	39573416	T	A
Scaffold-41	39573450	C	A
Scaffold-41	39573460	C	G
Scaffold-41	39573479	T	C
Scaffold-41	39573493	C	G,T
Scaffold-41	39573538	A	G
Scaffold-41	39573553	G	A
Scaffold-41	39759867	G	A
Scaffold-41	39759891	A	G
Scaffold-41	39759951	C	T
Scaffold-41	39939129	C	G
Scaffold-41	39939130	T	G
Scaffold-41	39939133	A	G
Scaffold-41	39939152	TC	CT
Scaffold-41	39939166	C	A
Scaffold-41	39939175	AT	ACTGA
Scaffold-41	39939207	GAA	GA
Scaffold-41	39939219	G	A
Scaffold-41	39939247	T	C

Scaffold-41	40151341	G	A
Scaffold-41	40151381	A	G
Scaffold-41	40203403	ATACAAAATT	AT
Scaffold-41	40203415	A	G
Scaffold-41	40216325	C	T
Scaffold-41	40216340	G	A
Scaffold-41	40216382	T	A
Scaffold-41	40216406	T	C
Scaffold-41	40300086	A	G
Scaffold-41	40300090	C	T
Scaffold-41	40300094	T	C
Scaffold-41	40300107	A	G
Scaffold-41	40300156	C	T
Scaffold-41	40443859	A	G
Scaffold-41	40443902	A	T
Scaffold-41	40443934	G	T
Scaffold-41	40443941	G	A
Scaffold-41	40612421	GT	GTTCAACACT
Scaffold-41	40612446	G	T
Scaffold-41	40635792	C	G
Scaffold-41	40635804	G	C
Scaffold-41	40679383	CGTTG	CGTCG,CG
Scaffold-41	40679397	CAA	CA
Scaffold-41	40679468	AACCA	AACCG,GACCG
Scaffold-41	40698729	A	C
Scaffold-41	40698808	T	C
Scaffold-41	40698841	TTGTGTGTGTGTGG	TTGTGTGTGTGCGG,TTGTGTGTGTGG
Scaffold-41	40788561	T	C
Scaffold-41	40788594	T	A
Scaffold-41	40788632	G	T
Scaffold-41	41063670	C	A
Scaffold-41	41063678	C	A
Scaffold-41	41063685	C	A
Scaffold-41	41063689	C	T
Scaffold-41	41063699	G	A
Scaffold-41	41063708	A	C
Scaffold-41	41063709	C	A
Scaffold-41	41063717	TCA	CCA,CCG
Scaffold-41	41063723	G	A
Scaffold-41	41063736	T	C
Scaffold-41	41063757	C	T
Scaffold-41	41063768	G	T
Scaffold-41	41063785	G	C

Scaffold-41	41183083	C	T
Scaffold-41	41183108	TGC	TC
Scaffold-41	41183188	A	C
Scaffold-41	41406414	G	A
Scaffold-41	41406476	C	T
Scaffold-41	41406528	C	G
Scaffold-41	41406759	T	G
Scaffold-41	41406789	C	T
Scaffold-41	41406816	G	A
Scaffold-41	41406877	C	G
Scaffold-41	41406894	A	T
Scaffold-41	41491194	G	T
Scaffold-41	41491266	T	C
Scaffold-41	41551237	A	G
Scaffold-41	41551240	C	T
Scaffold-41	41551280	TCC	TC
Scaffold-41	41551289	A	T
Scaffold-41	41551294	A	G
Scaffold-41	41551304	C	T
Scaffold-41	41551315	T	C
Scaffold-41	41551329	CC	AA
Scaffold-41	41551343	G	A
Scaffold-41	41637358	G	A
Scaffold-41	41637366	T	C
Scaffold-41	41713075	A	G
Scaffold-41	41713178	A	T
Scaffold-41	41719635	T	G
Scaffold-41	41719639	A	T
Scaffold-41	41719645	T	C
Scaffold-41	41719654	A	C
Scaffold-41	41719659	T	G
Scaffold-41	41719671	ACC	AC
Scaffold-41	41989429	T	C
Scaffold-41	41989444	C	T
Scaffold-41	41989463	T	A
Scaffold-41	41989477	T	C
Scaffold-41	41989479	T	G
Scaffold-41	42024290	A	T
Scaffold-41	42024311	C	G
Scaffold-41	42125983	C	T
Scaffold-41	42125990	A	G
Scaffold-41	42126010	T	C
Scaffold-41	42126011	G	A
Scaffold-41	42126023	GTT	GT

Scaffold-41	42126048	TG	TGGCAG
Scaffold-41	42126051	T	C
Scaffold-41	42126053	C	T
Scaffold-41	42126066	G	T
Scaffold-41	42126097	C	T
Scaffold-41	42511717	CAA	CAAA
Scaffold-41	42511798	C	A
Scaffold-41	42958224	T	C
Scaffold-41	42958329	T	C
Scaffold-41	42958331	T	G
Scaffold-41	43044439	G	A
Scaffold-41	43044518	T	C
Scaffold-41	43044534	AGGG	CGGG,CGGA,AGGA
Scaffold-41	43076016	A	G
Scaffold-41	43076025	T	A
Scaffold-41	43076040	G	A
Scaffold-41	43129004	T	G
Scaffold-41	43129061	A	G
Scaffold-41	43129573	C	T
Scaffold-41	43129601	A	G
Scaffold-41	43129620	T	C
Scaffold-41	43129667	C	A
Scaffold-41	43326504	TTC	CTT
Scaffold-41	43326515	C	A
Scaffold-41	43326527	A	G
Scaffold-41	43326563	A	G
Scaffold-41	43326569	ACT	AT
Scaffold-41	43326576	C	A
Scaffold-41	43365820	G	T
Scaffold-41	43365840	C	G
Scaffold-41	43365848	C	G
Scaffold-41	43365863	CCACT	CCACC,GCACC
Scaffold-41	43365886	T	G
Scaffold-41	43365890	T	C
Scaffold-41	43365901	GCCCCCG	GCCCCG
Scaffold-41	43365934	T	G
Scaffold-41	43365948	C	G
Scaffold-41	43407445	A	T
Scaffold-41	43407456	G	A
Scaffold-41	43407458	GCAA	ACAG
Scaffold-41	43407466	T	C
Scaffold-41	43407472	C	A
Scaffold-41	43407483	T	C
Scaffold-41	43407502	GC	GG

Scaffold-41	43407507	T	A
Scaffold-41	43407521	C	A
Scaffold-41	43407535	ACA	ACG,GCG
Scaffold-41	43447141	A	T
Scaffold-41	43447149	C	T
Scaffold-41	43447163	A	C
Scaffold-41	43447182	GTT	ATC,ATT
Scaffold-41	43447191	G	A
Scaffold-41	43447202	A	T
Scaffold-41	43447204	G	A
Scaffold-41	43447211	A	C
Scaffold-41	43447217	G	C
Scaffold-41	43447235	T	C
Scaffold-41	43447241	C	T
Scaffold-41	43447246	T	A
Scaffold-41	43447278	A	G
Scaffold-41	43447283	A	C
Scaffold-41	43502450	C	T
Scaffold-41	43502547	AGTAGACGC	GGTGGACGA
Scaffold-41	43575039	C	A
Scaffold-41	43575044	T	C
Scaffold-41	43575049	G	A
Scaffold-41	43575075	T	C
Scaffold-41	43575078	A	G
Scaffold-41	43575100	C	G
Scaffold-41	43607655	A	T
Scaffold-41	43607677	G	C
Scaffold-41	43607680	GAATTAATTG	GAATTAATTAATTG
Scaffold-41	43607703	T	C
Scaffold-41	43607706	A	T
Scaffold-41	43607709	G	A
Scaffold-41	43607718	A	G
Scaffold-41	43607742	A	G
Scaffold-41	43624455	C	T
Scaffold-41	43624473	A	C
Scaffold-41	43624495	T	G
Scaffold-41	43624520	G	A
Scaffold-41	43624551	A	G
Scaffold-41	43788359	T	C
Scaffold-41	43788366	C	A
Scaffold-41	43788391	T	C
Scaffold-41	43788417	A	C
Scaffold-41	43788431	C	T
Scaffold-41	43788461	G	A

Scaffold-41	43801185	T	C
Scaffold-41	43801189	A	T
Scaffold-41	43801230	C	G
Scaffold-41	43801256	G	A
Scaffold-41	43801263	CCAAATGTGATTGC	CCAAATGTGATTGCAAATGTGATTGC,CCAAACGTGATTGC
Scaffold-41	43802526	A	G
Scaffold-41	43802551	C	T
Scaffold-41	43802561	C	T
Scaffold-41	43802564	A	T
Scaffold-41	43802627	T	C
Scaffold-41	43802634	G	C
Scaffold-41	43802649	G	A
Scaffold-41	43802656	A	G
Scaffold-41	43846429	G	A
Scaffold-41	43846520	C	T
Scaffold-41	43846530	C	T
Scaffold-41	43881477	A	G
Scaffold-41	43881499	CT	CTTCGT
Scaffold-41	43881513	T	A
Scaffold-41	43881517	G	A
Scaffold-41	43881518	G	T
Scaffold-41	43881532	CTT	CTTT
Scaffold-41	43881548	A	T
Scaffold-41	43881549	T	G
Scaffold-41	43881563	TCG	TG
Scaffold-41	44151100	C	T
Scaffold-41	44151116	TAA	TAAA
Scaffold-41	44151132	AATG	AATATG
Scaffold-41	44151140	A	T
Scaffold-41	44161568	G	A
Scaffold-41	44161600	A	T
Scaffold-41	44161605	G	T
Scaffold-41	44161652	G	A
Scaffold-41	44161684	T	C
Scaffold-41	44161685	G	T
Scaffold-41	44495890	G	A,C
Scaffold-41	44540653	T	A
Scaffold-41	44664389	A	G
Scaffold-41	44664425	T	A
Scaffold-41	44664430	ATT	AT
Scaffold-41	44714144	ATAGG	TTAGGG
Scaffold-41	44714151	C	G
Scaffold-41	44714159	A	T

Scaffold-41	44714161	G	T
Scaffold-41	44714171	C	A
Scaffold-41	44714174	C	T
Scaffold-41	44714191	ACCAGG	TTCAGA,ACTAGG
Scaffold-41	44714210	A	G
Scaffold-41	44714215	G	T
Scaffold-41	44714224	T	C
Scaffold-41	44714226	T	C
Scaffold-41	44714241	T	C
Scaffold-41	44715596	A	G
Scaffold-41	44715615	G	A
Scaffold-41	44715640	TC	AT,AC
Scaffold-41	44715648	C	A
Scaffold-41	44715678	TAAAAAAAAT	TAAAAAAAAAAT,TAAAAAAAAAAAT
Scaffold-41	44715688	C	A
Scaffold-41	44715693	G	A
Scaffold-41	44715701	A	G
Scaffold-41	44715735	C	A
Scaffold-41	44871416	C	T
Scaffold-41	44871447	ACCA	ACCG,GCCG
Scaffold-41	44871525	T	A
Scaffold-41	44871540	A	G
Scaffold-41	44999496	A	G
Scaffold-41	44999499	T	C
Scaffold-41	44999544	G	A
Scaffold-41	44999555	C	G
Scaffold-41	45314590	G	A
Scaffold-41	45314592	G	A
Scaffold-41	45314625	AT	ACA,AC
Scaffold-41	45314632	CATA	CA
Scaffold-41	45314636	A	G
Scaffold-41	45314647	G	A
Scaffold-41	45314692	T	C
Scaffold-41	45333369	C	T
Scaffold-41	45333393	A	C
Scaffold-41	45346419	T	G
Scaffold-41	45346428	ATTT	AT
Scaffold-41	45346458	T	A
Scaffold-41	45346463	G	C
Scaffold-41	45346467	G	A
Scaffold-41	45385026	C	T
Scaffold-41	45385032	T	G
Scaffold-41	45385051	A	G

Scaffold-41	45385062	C	T
Scaffold-41	45454909	T	A,C
Scaffold-41	45454919	C	T
Scaffold-41	45454928	T	C
Scaffold-41	45454933	T	G
Scaffold-41	45454944	T	C
Scaffold-41	45454962	T	C
Scaffold-41	45454968	T	A
Scaffold-41	45454973	G	A
Scaffold-41	45454978	CCTGT	CCTGC,CATGC,GCTGC
Scaffold-41	45454991	CA	CG
Scaffold-41	45454996	C	T
Scaffold-41	45454997	C	T
Scaffold-41	45454998	GT	GTACT
Scaffold-41	45455003	G	A
Scaffold-41	45455011	A	G
Scaffold-41	45455014	G	A
Scaffold-41	45455015	G	A
Scaffold-41	45455021	A	T
Scaffold-41	45455026	A	T
Scaffold-41	45468823	T	C
Scaffold-41	45544992	CGAAT	TGAAC
Scaffold-41	45544998	A	C
Scaffold-41	45545001	A	T
Scaffold-41	45545006	GCTATT	TCTATT,TCTATA
Scaffold-41	45545036	CA	AT
Scaffold-41	45545039	A	T
Scaffold-41	45545040	C	A
Scaffold-41	45545047	G	T
Scaffold-41	45545048	T	C
Scaffold-41	45545076	C	A
Scaffold-41	45545080	C	T
Scaffold-41	45545084	A	C
Scaffold-41	45545090	GGA	GTT
Scaffold-41	45545104	C	A
Scaffold-41	45545110	T	C
Scaffold-41	45890011	A	T
Scaffold-41	45890017	A	G
Scaffold-41	45890029	A	G
Scaffold-41	45890084	T	C
Scaffold-41	45890088	C	T
Scaffold-41	45890092	G	A
Scaffold-41	46040486	T	C
Scaffold-41	46040493	T	A

Scaffold-41	46040494	GGAAATTTC	GGAAATTTGAAATTTC,GGAAAATTC
Scaffold-41	46040540	CCTAACG	CCGAACG,CCGAACA,CGCGAACA,TCGAACG
Scaffold-41	46040558	C	G
Scaffold-41	46194649	T	A
Scaffold-41	46194659	G	A,T
Scaffold-41	46194718	CTT	CT
Scaffold-41	46194738	A	G
Scaffold-41	46534061	T	C
Scaffold-41	46534082	G	A
Scaffold-41	46555521	T	A
Scaffold-41	46555539	C	T
Scaffold-41	46555545	G	C
Scaffold-41	46555584	A	G
Scaffold-41	46555607	T	G
Scaffold-41	46579301	C	A
Scaffold-41	46579308	A	G
Scaffold-41	46579328	T	C
Scaffold-41	46579356	T	G
Scaffold-41	46579369	G	C
Scaffold-41	46579374	G	A
Scaffold-41	46579424	A	G
Scaffold-41	46579427	T	C
Scaffold-41	46588089	C	T
Scaffold-41	46588097	T	C
Scaffold-41	46588105	G	T
Scaffold-41	46588136	T	C
Scaffold-41	46588190	A	G
Scaffold-41	46723623	A	T
Scaffold-41	46723648	A	T
Scaffold-41	46723680	A	T
Scaffold-41	46826081	C	T
Scaffold-41	46826153	T	C
Scaffold-41	46826158	ACA	AA
Scaffold-41	46828150	TA	CT
Scaffold-41	46828162	C	T
Scaffold-41	47101839	C	T
Scaffold-41	47101893	A	T
Scaffold-41	47140921	T	A
Scaffold-41	47140948	T	C
Scaffold-41	47290640	T	C
Scaffold-41	47290657	GTG	GCA,TCA
Scaffold-41	47290664	TGGCCATAGTTA	CGGACATGGTTC
Scaffold-41	47290688	T	C

Scaffold-41	47290708	C	A
Scaffold-41	47290742	G	T
Scaffold-41	47387056	C	A
Scaffold-41	47387063	TT	CC,CT
Scaffold-41	47387077	C	T
Scaffold-41	47387078	G	A
Scaffold-41	47387119	A	C
Scaffold-41	47472001	A	C
Scaffold-41	47472014	A	T,G
Scaffold-41	47472025	CAT	CAC,GAC
Scaffold-41	47472035	A	T
Scaffold-41	47472045	A	T
Scaffold-41	47472064	T	G
Scaffold-41	47472086	T	A
Scaffold-41	47472096	G	T
Scaffold-41	47472099	G	A
Scaffold-41	47472105	G	T
Scaffold-41	47472115	A	T
Scaffold-41	47562135	T	C
Scaffold-41	47562169	C	T
Scaffold-41	47562236	A	G
Scaffold-41	47562243	C	T
Scaffold-41	47570492	A	T
Scaffold-41	47570553	G	C
Scaffold-41	47570596	A	T
Scaffold-41	47642350	T	A
Scaffold-41	47642356	C	T
Scaffold-41	47642383	A	G
Scaffold-41	47642397	C	G
Scaffold-41	47642444	C	A
Scaffold-41	47642458	G	A
Scaffold-41	47642459	A	C
Scaffold-41	47642466	G	A
Scaffold-41	47642474	T	C
Scaffold-41	47968166	C	T
Scaffold-41	47968215	T	A
Scaffold-41	47968232	T	A
Scaffold-41	48483184	G	A
Scaffold-41	48483235	T	C
Scaffold-41	48483250	C	T
Scaffold-41	48503145	A	G
Scaffold-41	48503170	A	T
Scaffold-41	48503186	TG	CG
Scaffold-41	48547745	C	T

Scaffold-41	48547753	A	C
Scaffold-41	48592043	C	T
Scaffold-41	48592060	G	C
Scaffold-41	48592085	C	T
Scaffold-41	48592109	A	C
Scaffold-41	48592117	A	G
Scaffold-41	48592122	A	G
Scaffold-41	48592144	T	C
Scaffold-41	48592147	CGATC	CGATT,TGATT
Scaffold-41	48592166	C	G
Scaffold-41	48592167	G	A
Scaffold-41	48610283	G	A
Scaffold-41	48610324	G	T
Scaffold-41	48610332	T	C
Scaffold-41	48610347	T	A
Scaffold-41	48610365	T	G
Scaffold-41	48610384	C	T
Scaffold-41	48612331	C	T
Scaffold-41	48612362	A	T
Scaffold-41	48630152	A	T
Scaffold-41	48630154	T	C
Scaffold-41	48630155	A	G
Scaffold-41	48630202	G	A
Scaffold-41	48630225	A	G
Scaffold-41	48668530	C	T
Scaffold-41	48826690	A	G
Scaffold-41	48826733	A	T
Scaffold-41	48899696	G	A
Scaffold-41	48899735	T	G
Scaffold-41	48899747	G	A
Scaffold-41	48899769	A	G
Scaffold-41	48899771	C	G
Scaffold-41	48973961	A	G
Scaffold-41	48973964	T	G
Scaffold-41	48973999	C	T
Scaffold-41	48974015	G	A
Scaffold-41	48974039	G	C
Scaffold-41	48974048	T	C
Scaffold-41	48974060	A	G
Scaffold-41	49198701	T	C
Scaffold-41	49198748	T	C
Scaffold-41	49198791	ATACTTTTTTTTTTA	ATACTTTTTTTTTGA
Scaffold-41	49198811	A	G
Scaffold-41	49233920	G	A

Scaffold-41	49233930	G	T
Scaffold-41	49233937	A	C
Scaffold-41	49234005	C	T
Scaffold-41	49280348	A	G
Scaffold-41	49373974	G	C
Scaffold-41	49373992	C	T
Scaffold-41	49373998	C	A
Scaffold-41	49374011	A	G
Scaffold-41	49374018	A	G
Scaffold-41	49374039	C	T
Scaffold-41	49374043	A	C
Scaffold-41	49374047	C	T
Scaffold-41	49374060	T	C
Scaffold-41	49374079	C	T
Scaffold-41	49374092	G	A
Scaffold-41	49421576	G	A
Scaffold-41	49421592	TCC	TCA,AAA
Scaffold-41	49601452	T	C
Scaffold-41	49601486	G	T
Scaffold-41	49601513	C	G
Scaffold-41	49614548	TAAAC	TATAT,TAAAT,AATAT
Scaffold-41	49614580	TTTA	GTTT,TTTT
Scaffold-41	49614623	A	G
Scaffold-41	49621107	G	A
Scaffold-41	49646334	T	G
Scaffold-41	49646344	G	C
Scaffold-41	49646345	G	C
Scaffold-41	49646350	A	T
Scaffold-41	49646377	G	A
Scaffold-41	49646383	C	T
Scaffold-41	49693790	TTGTGTGTGT	TTGTGTGTGTGT
Scaffold-41	49693832	G	T
Scaffold-41	49693875	TTGGTGGA	TTGGA
Scaffold-41	49693891	C	G
Scaffold-41	49726424	C	T
Scaffold-41	49726489	C	T
Scaffold-41	49726526	T	C
Scaffold-41	49860811	G	A
Scaffold-41	49860945	C	A
Scaffold-41	49977347	AGA	AGGA,AGAA
Scaffold-41	49977358	C	G
Scaffold-41	49977369	T	G
Scaffold-41	49977378	A	G
Scaffold-41	49977383	T	C

Scaffold-41	49977403		T	C
Scaffold-41	49977405		C	A
Scaffold-41	49999989		G	C
Scaffold-42	48388	C	G	
Scaffold-42	48392	CG	CT	
Scaffold-42	48426	A	G	
Scaffold-42	48464	A	C	
Scaffold-42	48501	A	G	
Scaffold-42	160310		C	T
Scaffold-42	160348		G	A
Scaffold-42	160349		C	A
Scaffold-42	160370		T	C
Scaffold-42	160373		C	A
Scaffold-42	160374		G	A
Scaffold-42	160402		T	C
Scaffold-42	160405		A	G
Scaffold-42	299889		G	A
Scaffold-42	299890		C	T
Scaffold-42	299900		C	G
Scaffold-42	299934		G	A
Scaffold-42	299968		G	T
Scaffold-42	299972		A	G
Scaffold-42	299977		T	C
Scaffold-42	534337		A	G
Scaffold-42	534347		T	G
Scaffold-42	534360		C	G
Scaffold-42	534367		AT	TA
Scaffold-42	534375		CTGTGTGT	CTGTGT
Scaffold-42	534401		A	G
Scaffold-42	534418		A	G
Scaffold-42	534423		T	C
Scaffold-42	534436		C	T
Scaffold-42	534445		A	G
Scaffold-42	534471		G	C
Scaffold-42	583147		T	C
Scaffold-42	583168		T	C
Scaffold-42	583195		TGG	AGG,AGC
Scaffold-42	583207		C	A
Scaffold-42	583215		A	G
Scaffold-42	627687		C	A
Scaffold-42	627717		A	G
Scaffold-42	627721		A	G
Scaffold-42	627744		G	C
Scaffold-42	707831		T	G

Scaffold-42	707873	C	T
Scaffold-42	726769	T	A
Scaffold-42	726780	GTCC	GTCG,TTCC
Scaffold-42	726804	G	A
Scaffold-42	726812	C	G
Scaffold-42	726825	C	T
Scaffold-42	726843	C	T
Scaffold-42	726857	G	A
Scaffold-42	804922	T	C
Scaffold-42	804948	G	A
Scaffold-42	804962	CG	CTG
Scaffold-42	805014	G	A
Scaffold-42	973726	T	C
Scaffold-42	973745	G	A
Scaffold-42	1060861	C	T
Scaffold-42	1060882	C	T
Scaffold-42	1060912	TTGA	TTGC,CTGC,TTGT
Scaffold-42	1060930	T	C
Scaffold-42	1060942	A	G
Scaffold-42	1060951	CG	AA
Scaffold-42	1060972	T	A
Scaffold-42	1060974	T	A
Scaffold-42	1060988	T	C
Scaffold-42	1061013	C	T
Scaffold-42	1061021	T	C
Scaffold-42	1061030	A	G
Scaffold-42	1061047	C	G
Scaffold-42	1061050	G	A
Scaffold-42	1061052	C	T
Scaffold-42	1061088	T	C
Scaffold-42	1061098	C	T
Scaffold-42	1061100	C	T
Scaffold-42	1243198	A	T
Scaffold-42	1243199	A	G
Scaffold-42	1243828	A	G
Scaffold-42	1243844	AGG	AG
Scaffold-42	1243854	AATTGCAGAATGTACAAGATCTA	AA
Scaffold-42	1243886	T	G
Scaffold-42	1243895	GTA	ATA,GA
Scaffold-42	1243911	GA	GGTTTA
Scaffold-42	1243933	C	A
Scaffold-42	1243942	GAA	GA
Scaffold-42	1243946	A	C
Scaffold-42	1243965	TAGT	CAGT,AAGG,TAAT

Scaffold-42	1243987	AA	ACT,ATT
Scaffold-42	1244048	TCC	TC
Scaffold-42	1244056	C	T
Scaffold-42	1244083	A	G
Scaffold-42	1423877	G	C
Scaffold-42	1423959	C	T
Scaffold-42	1423960	G	T
Scaffold-42	1491681	G	T
Scaffold-42	1491693	T	C
Scaffold-42	1491739	GTATAAACT	GT
Scaffold-42	1491749	T	C
Scaffold-42	1491807	C	T
Scaffold-42	1554550	C	A
Scaffold-42	1554616	A	G
Scaffold-42	1554636	G	A
Scaffold-42	1758000	T	C
Scaffold-42	1758016	A	C
Scaffold-42	1758098	C	G
Scaffold-42	1758125	C	T
Scaffold-42	1762403	T	C
Scaffold-42	1762478	G	T
Scaffold-42	1793052	C	T
Scaffold-42	1793088	G	T
Scaffold-42	1793094	GA	AC
Scaffold-42	1793104	T	C
Scaffold-42	1793116	G	T
Scaffold-42	1793128	G	A
Scaffold-42	1793170	C	T
Scaffold-42	1809871	C	T
Scaffold-42	1920986	T	C
Scaffold-42	1921026	C	T
Scaffold-42	1921053	T	G
Scaffold-42	1921056	C	T
Scaffold-42	1921064	TCT	TCC,TT
Scaffold-42	1921067	TTGT	TTGCT
Scaffold-42	1921106	A	G
Scaffold-42	1974355	T	A
Scaffold-42	1974366	T	A
Scaffold-42	1974369	TCCA	CCCA,CCCC
Scaffold-42	1974381	T	A
Scaffold-42	1974393	T	A
Scaffold-42	1974424	T	A
Scaffold-42	1974436	C	T
Scaffold-42	1974470	G	C

Scaffold-42	2000748	C	T
Scaffold-42	2000835	C	T
Scaffold-42	2018238	T	C
Scaffold-42	2018283	C	G
Scaffold-42	2018284	A	C
Scaffold-42	2018311	A	T
Scaffold-42	2156294	C	T
Scaffold-42	2156326	G	A
Scaffold-42	2288458	A	G
Scaffold-42	2288483	T	C
Scaffold-42	2288494	CTT	CT
Scaffold-42	2288499	AAC	CAC,CAG
Scaffold-42	2288528	A	T
Scaffold-42	2288557	T	G
Scaffold-42	2288587	C	T
Scaffold-42	2337278	T	A
Scaffold-42	2337315	C	T
Scaffold-42	2337329	T	C
Scaffold-42	2380666	CGGGG	TGGGG,TGGGA
Scaffold-42	2380705	T	C
Scaffold-42	2380718	C	T
Scaffold-42	2449758	T	C
Scaffold-42	2449817	C	G
Scaffold-42	2498924	GT	AA
Scaffold-42	2498950	G	T
Scaffold-42	2564666	G	T
Scaffold-42	2564687	G	A
Scaffold-42	2564712	G	T
Scaffold-42	2564739	G	A
Scaffold-42	2564775	C	G
Scaffold-42	2594430	G	A
Scaffold-42	2594656	A	G
Scaffold-42	2594670	TAAAAAAAAAAAT	TAAAAAAATAAAAAT
Scaffold-42	2616620	T	G
Scaffold-42	2616693	A	G
Scaffold-42	2616709	G	T
Scaffold-42	2641640	G	A
Scaffold-42	2641666	A	C
Scaffold-42	2641707	A	T
Scaffold-42	3070631	C	A
Scaffold-42	3070657	C	G
Scaffold-42	3070659	T	G
Scaffold-42	3200640	C	T
Scaffold-42	3200663	T	C

Scaffold-42	3265051	A	G
Scaffold-42	3265065	GTT	GT
Scaffold-42	3265080	T	C
Scaffold-42	3265122	G	A
Scaffold-42	3265124	T	C
Scaffold-42	3333617	G	A
Scaffold-42	3359012	TCGG	TCGC
Scaffold-42	3359025	C	T
Scaffold-42	3581962	C	T
Scaffold-42	3581966	A	G
Scaffold-42	3581970	A	G
Scaffold-42	3581986	G	T
Scaffold-42	3582003	T	G
Scaffold-42	3582033	G	A
Scaffold-42	3582072	A	G
Scaffold-42	3582077	A	G
Scaffold-42	3582085	A	G
Scaffold-42	3582101	T	A
Scaffold-42	3607846	T	G
Scaffold-42	3607860	G	T
Scaffold-42	3607863	A	C
Scaffold-42	3607875	G	T
Scaffold-42	3607880	G	A
Scaffold-42	3607893	CGCAGCCTTCCGGTAAACCGTTCTCAG	CGCAGCCTTCCGGTAAACCGTTCACAG,CGCAGCCTTCAGGTAAACCGTTCTCAG,CG,CGCAGCCTTCTGGTAAACCGTTCACAG,CGCAGCCCTCCGGTAAACCGTTCTCAG
Scaffold-42	3607929	A	T
Scaffold-42	3607945	A	C
Scaffold-42	3607965	C	A
Scaffold-42	3607987	G	C
Scaffold-42	3650326	C	G
Scaffold-42	3650339	G	A
Scaffold-42	3810504	A	G
Scaffold-42	3810780	G	T
Scaffold-42	3870402	A	G
Scaffold-42	3870437	G	A
Scaffold-42	3927675	C	T
Scaffold-42	3927717	C	T
Scaffold-42	3927759	T	A
Scaffold-42	3927781	G	A
Scaffold-42	3927790	A	G
Scaffold-42	3927791	C	A
Scaffold-42	4037624	C	A
Scaffold-42	4037637	T	C

Scaffold-42	4037664	G	A
Scaffold-42	4037789	A	G
Scaffold-42	4037823	G	A
Scaffold-42	4037856	C	G
Scaffold-42	4066471	C	T
Scaffold-42	4066495	C	T
Scaffold-42	4158970	T	C
Scaffold-42	4159099	A	T
Scaffold-42	4296118	A	G
Scaffold-42	4296248	G	A
Scaffold-42	4296278	T	A
Scaffold-42	4296292	T	C
Scaffold-42	4296312	G	C
Scaffold-42	4297729	C	T
Scaffold-42	4297734	C	T
Scaffold-42	4297750	C	T
Scaffold-42	4297786	T	C
Scaffold-42	4297835	G	A
Scaffold-42	4298383	A	C
Scaffold-42	4298394	A	G
Scaffold-42	4298407	T	C
Scaffold-42	4298455	T	C
Scaffold-42	4472809	G	A
Scaffold-42	4472846	T	G
Scaffold-42	4472889	G	T
Scaffold-42	4472933	G	C
Scaffold-42	4472943	G	A
Scaffold-42	4531057	T	C
Scaffold-42	4531085	T	C
Scaffold-42	4531106	A	G
Scaffold-42	4531375	A	G
Scaffold-42	4531401	C	T
Scaffold-42	4629628	T	A
Scaffold-42	4629634	C	A
Scaffold-42	4629700	G	A
Scaffold-42	4771118	A	G
Scaffold-42	4876391	T	C
Scaffold-42	4876429	C	G
Scaffold-42	4876461	T	C
Scaffold-42	4876492	C	T
Scaffold-42	4900663	G	C
Scaffold-42	4913831	G	T
Scaffold-42	4913835	A	C
Scaffold-42	4913852	G	T

Scaffold-42	4913902	A	T
Scaffold-42	4936011	T	G
Scaffold-42	4936069	A	T
Scaffold-42	4943196	A	T
Scaffold-42	4943199	A	G
Scaffold-42	4943230	T	A
Scaffold-42	4943236	T	G
Scaffold-42	4943258	ACG	ACA,TCG
Scaffold-42	4943277	T	A
Scaffold-42	4943303	T	C
Scaffold-42	4943328	A	G
Scaffold-42	4950933	T	G
Scaffold-42	4950999	T	G
Scaffold-42	4951006	G	A
Scaffold-42	4951855	GATT	AATAA
Scaffold-42	4951869	A	G
Scaffold-42	4951916	A	T
Scaffold-42	4951923	C	T
Scaffold-42	4981394	C	T
Scaffold-42	4981511	A	G
Scaffold-42	5000742	C	T
Scaffold-42	5000751	C	T
Scaffold-42	5000841	G	A
Scaffold-42	5010276	TA	AC
Scaffold-42	5062541	C	T
Scaffold-42	5062555	A	G
Scaffold-42	5062558	A	G
Scaffold-42	5062567	C	T
Scaffold-42	5062595	G	A
Scaffold-42	5062600	C	T
Scaffold-42	5062610	A	G
Scaffold-42	5062645	C	T
Scaffold-42	5062666	G	A
Scaffold-42	5126236	T	C
Scaffold-42	5138356	G	T
Scaffold-42	5138360	A	C
Scaffold-42	5512786	G	A
Scaffold-42	5512794	C	A
Scaffold-42	5512801	A	T
Scaffold-42	5515453	GA	TA
Scaffold-42	5606273	T	C
Scaffold-42	5606321	T	G
Scaffold-42	5606382	C	G
Scaffold-42	5676045	C	T

Scaffold-42	5676101	AGCGGGTA	AA
Scaffold-42	5676124	T	C
Scaffold-42	5676132	G	A
Scaffold-42	5739828	CTAG	CTATAG,CTGG
Scaffold-42	5739846	T	C
Scaffold-42	5739853	G	A
Scaffold-42	5739881	C	T
Scaffold-42	5739947	T	C
Scaffold-42	5756934	ATT	ATC,CTC
Scaffold-42	5756959	G	A
Scaffold-42	5756995	C	A
Scaffold-42	5756996	G	T
Scaffold-42	5757016	C	G
Scaffold-42	5757021	G	A
Scaffold-42	5762338	T	C
Scaffold-42	5762398	T	G
Scaffold-42	5762444	T	C
Scaffold-42	5860735	T	G
Scaffold-42	5860739	T	A
Scaffold-42	5860744	A	T
Scaffold-42	5860747	CTC	TTT
Scaffold-42	5860750	A	G
Scaffold-42	5860781	A	T
Scaffold-42	5860783	T	C
Scaffold-42	5860789	T	A
Scaffold-42	5860807	C	A
Scaffold-42	5860824	C	T
Scaffold-42	5860843	G	A
Scaffold-42	5907158	T	G
Scaffold-42	5907188	G	C
Scaffold-42	5907194	T	C
Scaffold-42	5907207	T	G
Scaffold-42	5907227	C	G
Scaffold-42	5907248	A	T
Scaffold-42	5907281	A	G
Scaffold-42	5995107	T	C
Scaffold-42	5995131	T	C
Scaffold-42	5995134	G	C
Scaffold-42	5995149	A	G
Scaffold-42	5995152	T	C
Scaffold-42	5995170	T	C
Scaffold-42	5995175	T	C
Scaffold-42	5995206	C	T
Scaffold-42	6077589	C	G

Scaffold-42	6077642	T	C
Scaffold-42	6077659	A	T
Scaffold-42	6102539	TC	TAC
Scaffold-42	6102562	G	T
Scaffold-42	6102572	TAATGAATGA	TAATGA
Scaffold-42	6102600	T	C
Scaffold-42	6102620	T	C
Scaffold-42	6126428	C	T
Scaffold-42	6223977	G	A
Scaffold-42	6223987	T	G
Scaffold-42	6224009	A	T
Scaffold-42	6224012	ACTTCTTC	ACTTC
Scaffold-42	6378898	G	A
Scaffold-42	6378928	GAGC	GAGA,TAGA
Scaffold-42	6378946	T	A
Scaffold-42	6378971	G	T
Scaffold-42	6452768	A	G
Scaffold-42	6452803	C	A
Scaffold-42	6452827	C	G
Scaffold-42	6480230	T	C
Scaffold-42	6480308	T	C
Scaffold-42	6480309	G	T
Scaffold-42	6480323	T	C
Scaffold-42	6582295	T	G
Scaffold-42	6582308	G	T
Scaffold-42	6582316	TTCA	CTCA
Scaffold-42	6582328	T	A
Scaffold-42	6583358	A	C
Scaffold-42	6583453	GA	CG
Scaffold-42	6585301	C	A
Scaffold-42	6585341	T	G
Scaffold-42	6585419	G	A
Scaffold-42	6585434	T	C
Scaffold-42	6620582	C	G
Scaffold-42	6620597	T	G
Scaffold-42	6620599	T	A
Scaffold-42	6620604	G	C
Scaffold-42	6620620	T	C
Scaffold-42	6620622	T	C
Scaffold-42	6673656	T	G
Scaffold-42	6673690	T	C
Scaffold-42	6673718	G	A
Scaffold-42	6673732	A	T
Scaffold-42	6716289	T	G

Scaffold-42	6895776	A	T	
Scaffold-42	6895888	G	A	
Scaffold-42	6977679	T	C	
Scaffold-42	6977681	G	A	
Scaffold-42	6977714	A	C	
Scaffold-42	6977750	C	T	
Scaffold-42	6977756	C	T	
Scaffold-42	6977765	C	A	
Scaffold-42	7009132	C	T	
Scaffold-42	7009198	C	T	
Scaffold-42	7011218	A	C	
Scaffold-42	7011220	T	C	
Scaffold-42	7011251	C	G	
Scaffold-42	7011285	A	G	
Scaffold-42	7011324	T	C	
Scaffold-42	7027840	G	A	
Scaffold-42	7027845	G	A	
Scaffold-42	7027852	C	T	
Scaffold-42	7135031	TCT	TTT,TT	
Scaffold-42	7191790	A	C	
Scaffold-42	7191866	A	G	
Scaffold-42	7191893	G	A	
Scaffold-42	7191919	A	C	
Scaffold-42	7191921	GGTTG	GG	
Scaffold-42	7277902	CGT	TGG,CGG	
Scaffold-42	7277971	A	G	
Scaffold-42	7420321	T	A	
Scaffold-42	7420358	A	C	
Scaffold-42	7420369	G	A	
Scaffold-42	7425507	C	T	
Scaffold-42	7425508	G	T	
Scaffold-42	7425526	A	G	
Scaffold-42	7425541	T	C	
Scaffold-42	7428796	G	A	
Scaffold-42	7474472	C	A	
Scaffold-42	7474485	G	A	
Scaffold-42	7474487	A	G	
Scaffold-42	7474570	T	A	
Scaffold-42	7514894	T	G	
Scaffold-42	7642260	GTATAAGAAC		TTATTCGAAA
Scaffold-42	7718514	C	T	
Scaffold-42	7828634	A	G	
Scaffold-42	7935047	T	G	
Scaffold-42	8199560	A	G	

Scaffold-42	8199566	G	T
Scaffold-42	8199607	CAA	CA
Scaffold-42	8199618	G	T
Scaffold-42	8199630	T	C
Scaffold-42	8199637	AAGTATTGTCATCAAGTGTAATGA	AAGTATTGTCATCAAATGTAATGA
Scaffold-42	8199671	T	A
Scaffold-42	8199680	T	C
Scaffold-42	8232634	C	T
Scaffold-42	8232650	T	C
Scaffold-42	8232651	A	G
Scaffold-42	8232712	ATAC	CTAT
Scaffold-42	8294000	A	G
Scaffold-42	8294051	CAAGGGGAACA	
Scaffold-42	8294084	G	T
Scaffold-42	8294093	T	G
Scaffold-42	8294099	A	G
Scaffold-42	8334635	G	A
Scaffold-42	8334645	C	T
Scaffold-42	8334711	G	A
Scaffold-42	8350475	A	G
Scaffold-42	8350490	C	T
Scaffold-42	8350538	TA	TAAA
Scaffold-42	8381642	G	A
Scaffold-42	8381680	A	C
Scaffold-42	8381694	A	G
Scaffold-42	8381696	A	C
Scaffold-42	8381700	G	T
Scaffold-42	8381709	T	G
Scaffold-42	8381729	C	T
Scaffold-42	8381741	T	C
Scaffold-42	8381748	C	T
Scaffold-42	8381755	C	T
Scaffold-42	8381758	G	A
Scaffold-42	8432874	T	C
Scaffold-42	8432877	ATCG	ATCTCG
Scaffold-42	8432884	C	T
Scaffold-42	8432897	A	G
Scaffold-42	8432928	C	T
Scaffold-42	8432935	G	C
Scaffold-42	8432974	G	A
Scaffold-42	8432981	C	T
Scaffold-42	8464700	G	T
Scaffold-42	8464706	TAG	AAG

Scaffold-42	8464736	T	A
Scaffold-42	8578366	G	A
Scaffold-42	8578369	G	T
Scaffold-42	8578395	G	A
Scaffold-42	8578401	C	A
Scaffold-42	8578415	A	G
Scaffold-42	8578450	TAAAAAAT	TAAAAAAAT
Scaffold-42	8654309	C	T
Scaffold-42	8654335	G	T
Scaffold-42	8654345	G	T
Scaffold-42	8654424	C	G
Scaffold-42	8654435	A	G
Scaffold-42	8871844	T	C
Scaffold-42	8871929	G	C
Scaffold-42	8871946	C	T
Scaffold-42	8918615	C	T
Scaffold-42	8953197	A	T
Scaffold-42	8953199	A	G
Scaffold-42	8953203	A	G
Scaffold-42	8953216	TATAATA	
Scaffold-42	8953223	C	T
Scaffold-42	8953232	A	G
Scaffold-42	8953247	C	T
Scaffold-42	8953255	A	G
Scaffold-42	8953259	T	A
Scaffold-42	8953284	T	A
Scaffold-42	8953309	T	C
Scaffold-42	9047564	A	G
Scaffold-42	9047606	G	T
Scaffold-42	9102336	A	T
Scaffold-42	9102366	T	C
Scaffold-42	9102390	A	G
Scaffold-42	9102409	TGA	TA
Scaffold-42	9102463	AT	ATATT
Scaffold-42	9162340	G	A
Scaffold-42	9162343	C	T
Scaffold-42	9162349	G	C
Scaffold-42	9162381	G	A
Scaffold-42	9162421	T	C
Scaffold-42	9162432	G	A
Scaffold-42	9162441	T	A
Scaffold-42	9188454	A	G
Scaffold-42	9341040	C	A
Scaffold-42	9341114	C	T

Scaffold-42	9343746	CTTTTTTTTC	CTTTTTTTTTC,CTTATTTTTC
Scaffold-42	9408534	T	A
Scaffold-42	9456596	T	C
Scaffold-42	9456667	C	A
Scaffold-42	9600434	C	A
Scaffold-42	9600470	T	G
Scaffold-42	9600479	C	G
Scaffold-42	9600497	A	T
Scaffold-42	9600532	T	G
Scaffold-42	9600534	G	T
Scaffold-42	9600539	AGG	GGG,GGT,AGT
Scaffold-42	9745572	T	C
Scaffold-42	9745583	A	G
Scaffold-42	9745639	G	C
Scaffold-42	9748343	C	G
Scaffold-42	9872375	T	C
Scaffold-42	9872464	C	G
Scaffold-42	9872504	A	T
Scaffold-42	9872506	C	G
Scaffold-42	9872518	G	A
Scaffold-42	9973509	C	A
Scaffold-42	9973531	CAA	CA
Scaffold-42	9994178	T	C
Scaffold-42	9994261	C	G
Scaffold-42	9994272	CTT	CTTT
Scaffold-42	9994275	A	G
Scaffold-42	10008961	G	A
Scaffold-42	10009038	T	C
Scaffold-42	10009039	A	T
Scaffold-42	10009040	CTTTTTTTC	CTTTTTTC
Scaffold-42	10019903	C	G
Scaffold-42	10019986	G	A
Scaffold-42	10020012	A	G
Scaffold-42	10020019	C	T
Scaffold-42	10040017	GA	GCA
Scaffold-42	10040039	C	T
Scaffold-42	10040045	A	C
Scaffold-42	10040100	CAA	CA
Scaffold-42	10040111	C	T
Scaffold-42	10040124	G	A
Scaffold-42	10040126	T	C
Scaffold-42	10040148	AGC	AC
Scaffold-42	10058535	A	G
Scaffold-42	10075002	T	C

Scaffold-42	10075109	GAAAAAAAT	GAAAAAAT
Scaffold-42	10078583	TTGGACTTGGT	TTGAACTTGGT
Scaffold-42	10078693	G	C
Scaffold-42	10172733	GTCTGCCTCTGGGAGAGGGAGGGTCATTGACTAAATTT	GTCTGCCTCTGGGAGAGGGAGGGTCATTGACTAAGTTT,GTCTTCCTCTGGGAGAGGGAGGGTCATTGACTAAGTTT
Scaffold-42	10172786	G	C
Scaffold-42	10172863	C	T
Scaffold-42	10179770	A	C
Scaffold-42	10179785	T	C
Scaffold-42	10179885	C	T
Scaffold-42	10182644	C	T
Scaffold-42	10182674	GAAG	GAAAAG
Scaffold-42	10182679	G	T
Scaffold-42	10182694	C	T
Scaffold-42	10182696	A	T
Scaffold-42	10182704	TAACA	TAATG,TA
Scaffold-42	10182709	AG	AA,AT
Scaffold-42	10182716	G	A
Scaffold-42	10252704	C	T
Scaffold-42	10327152	C	T
Scaffold-42	10327167	G	A
Scaffold-42	10327205	T	G
Scaffold-42	10327220	G	A
Scaffold-42	10444397	G	A
Scaffold-42	10444441	G	A
Scaffold-42	10444446	A	G
Scaffold-42	10444454	G	A
Scaffold-42	10444469	A	T
Scaffold-42	10578971	C	T
Scaffold-42	10578997	C	T
Scaffold-42	10579014	G	A
Scaffold-42	10579022	A	G
Scaffold-42	10579024	C	A
Scaffold-42	10579033	A	G
Scaffold-42	10716596	TAA	TAAA
Scaffold-42	10716602	C	T
Scaffold-42	10716608	TGT	CGT
Scaffold-42	10716627	A	G
Scaffold-42	11068473	C	T
Scaffold-42	11068488	C	T
Scaffold-42	11068530	A	G
Scaffold-42	11068546	G	A

Scaffold-42	11089079	A	C	
Scaffold-42	11225772	A	G	
Scaffold-42	11425668	A	G	
Scaffold-42	11438961	T	A	
Scaffold-42	11439017	C	T	
Scaffold-42	11501768	AGG	AG	
Scaffold-42	11501778	C	G	
Scaffold-42	11501779	C	T	
Scaffold-42	11501799	G	A	
Scaffold-42	11501800	G	A	
Scaffold-42	11501813	C	T	
Scaffold-42	11716972	ATTTG		TTTTG,TTTTC
Scaffold-42	11716996	G	A	
Scaffold-42	11717018	T	C	
Scaffold-42	11717022	C	T	
Scaffold-42	11717034	T	C	
Scaffold-42	11717044	C	T	
Scaffold-42	11717051	G	T	
Scaffold-42	11733396	ATT	AT	
Scaffold-42	11733428	T	A	
Scaffold-42	11733548	C	T	
Scaffold-42	11733555	G	A	
Scaffold-42	11753788	TCG	TCT	
Scaffold-42	11753827	CGGCG		TGGCA,CCGCG,CGGTG
Scaffold-42	11753909	G	A	
Scaffold-42	11876746	A	G	
Scaffold-42	11876780	G	A	
Scaffold-42	11876873	G	A	
Scaffold-42	12108145	C	T	
Scaffold-42	12108169	C	T	
Scaffold-42	12108184	A	T	
Scaffold-42	12108195	T	C	
Scaffold-42	12108201	A	T	
Scaffold-42	12108243	T	A	
Scaffold-42	12115158	G	T	
Scaffold-42	12115183	A	T	
Scaffold-42	12115201	G	A	
Scaffold-42	12115251	A	G	
Scaffold-42	12115269	T	C	
Scaffold-42	12115284	T	G	
Scaffold-42	12379810	A	C	
Scaffold-42	12379915	A	G	
Scaffold-42	12566911	G	C	
Scaffold-42	12566915	G	T	

Scaffold-42	12566923	C	A	
Scaffold-42	12566999	C	G,T	
Scaffold-42	12697721	GAA	AAA,AAG	
Scaffold-42	12697734	G	T	
Scaffold-42	12697739	GCAC	ACAT	
Scaffold-42	12697749	C	T	
Scaffold-42	12697818	A	C	
Scaffold-42	12697827	G	T	
Scaffold-42	12724524	A	G	
Scaffold-42	12724546	G	C	
Scaffold-42	12724575	A	G	
Scaffold-42	12875505	G	A	
Scaffold-42	12875530	CATGA		CGA
Scaffold-42	12875537	TCAA	TTAA	
Scaffold-42	12881784	G	T	
Scaffold-42	12881814	G	A	
Scaffold-42	12881872	G	A	
Scaffold-42	12881873	GC	GT,AT	
Scaffold-42	12901054	C	T	
Scaffold-42	12901087	C	A	
Scaffold-42	12901088	C	G	
Scaffold-42	12901108	GTTCT		ATTCT
Scaffold-42	12912252	A	G	
Scaffold-42	12912300	C	T	
Scaffold-42	12922001	C	G	
Scaffold-42	12922019	C	T	
Scaffold-42	12922056	A	G	
Scaffold-42	13045896	T	A	
Scaffold-42	13045902	C	G	
Scaffold-42	13045918	G	A	
Scaffold-42	13045928	G	A	
Scaffold-42	13045996	C	T	
Scaffold-42	13349452	C	G	
Scaffold-42	13349458	G	A	
Scaffold-42	13349472	C	T	
Scaffold-42	13558901	T	G	
Scaffold-42	13558921	T	C	
Scaffold-42	13558927	A	G	
Scaffold-42	13558934	T	A	
Scaffold-42	13558938	G	A	
Scaffold-42	13558968	GGTCG		GG,GGTCA
Scaffold-42	13558977	T	C	
Scaffold-42	13559012	G	A	
Scaffold-42	13559020	G	A	

Scaffold-42	13559034	A	G
Scaffold-42	13626814	T	A
Scaffold-42	13626826	G	A
Scaffold-42	13626845	T	G
Scaffold-42	13626858	GC	AA
Scaffold-42	13901124	C	T
Scaffold-42	13901141	T	C
Scaffold-42	13920428	G	A
Scaffold-42	13920445	A	G
Scaffold-42	13920466	G	C
Scaffold-42	13920484	A	G
Scaffold-42	13920503	G	A
Scaffold-42	13920526	A	G
Scaffold-42	13920531	A	G
Scaffold-42	13920553	C	T
Scaffold-42	13956343	T	C
Scaffold-42	14019241	A	G
Scaffold-42	14020830	A	T
Scaffold-42	14020837	A	T
Scaffold-42	14020845	G	T
Scaffold-42	14020878	A	G
Scaffold-42	14020928	T	C
Scaffold-42	14020946	G	A
Scaffold-42	14035607	A	G
Scaffold-42	14035615	A	T
Scaffold-42	14035712	C	G
Scaffold-42	14316535	TACCG	CACCA,CTCCA
Scaffold-42	14316567	GA	GG
Scaffold-42	14527409	GGCA	AGCA,AGCG,ACCA
Scaffold-42	14527424	C	A
Scaffold-42	14527427	C	T
Scaffold-42	14527476	A	T
Scaffold-42	14527507	A	G
Scaffold-42	14527523	C	T
Scaffold-42	14527530	T	C
Scaffold-42	14670998	T	C
Scaffold-42	14671044	GACA	GACACA
Scaffold-42	14671063	TAA	AAC,AAA
Scaffold-42	14695365	GAAAAAAAG	GAAAAAAAAG,GAAAAAAAAT
Scaffold-42	14695381	G	A
Scaffold-42	14695386	G	C
Scaffold-42	14773705	T	A
Scaffold-42	14773763	G	A
Scaffold-42	14773792	A	G

Scaffold-42	14773814	T	C
Scaffold-42	14773833	CTCTG	CTCTC,GTCTC
Scaffold-42	14779418	T	C
Scaffold-42	14779424	AGGG	ACGG,AG
Scaffold-42	14779428	G	T
Scaffold-42	14779438	T	A
Scaffold-42	14779442	T	C
Scaffold-42	14779451	G	T
Scaffold-42	14779458	T	C
Scaffold-42	14779465	A	T
Scaffold-42	14779499	A	G
Scaffold-42	15044102	G	A
Scaffold-42	15044166	T	G
Scaffold-42	15200886	A	G
Scaffold-42	15200908	GAGCAT	GAGCAA,TGGCAA
Scaffold-42	15200935	T	G
Scaffold-42	15200987	A	G
Scaffold-42	15235572	G	C
Scaffold-42	15235584	G	A
Scaffold-42	15235597	G	A
Scaffold-42	15235606	C	T
Scaffold-42	15235607	G	A
Scaffold-42	15235608	AGTTGAAACGG	AGTTGAAACAG,AGTTGAAATGG,AG
Scaffold-42	15235628	C	T
Scaffold-42	15235639	G	A
Scaffold-42	15235643	C	T
Scaffold-42	15235648	C	T
Scaffold-42	15235653	C	A
Scaffold-42	15235664	C	A
Scaffold-42	15235665	C	T
Scaffold-42	15235679	G	T
Scaffold-42	15235688	C	A
Scaffold-42	15235706	A	T
Scaffold-42	15235712	A	T
Scaffold-42	15322174	C	T
Scaffold-42	15322229	C	T
Scaffold-42	15339449	GGCTT	GGCTC
Scaffold-42	15339490	AG	TA,TG
Scaffold-42	15339534	A	G
Scaffold-42	15339542	C	A
Scaffold-42	15477902	TAAACACAAACACA	TAAACACAATCACA,TAAACACAAACACAAACACA
Scaffold-42	15477920	C	A

Scaffold-42	15477972	A	G
Scaffold-42	15477973	T	G
Scaffold-42	15477979	G	C
Scaffold-42	15636828	C	T
Scaffold-42	15636914	G	T
Scaffold-42	15681175	C	G
Scaffold-42	15681177	G	A
Scaffold-42	15681206	A	C
Scaffold-42	15681218	T	A
Scaffold-42	15681221	T	A
Scaffold-42	15681230	T	C
Scaffold-42	15681245	A	G
Scaffold-42	15681254	GCCA	CCCA,CCCG
Scaffold-42	15826267	G	A
Scaffold-42	15826319	G	A
Scaffold-42	15826369	T	G
Scaffold-42	15826384	T	A
Scaffold-42	15847776	C	A
Scaffold-42	15847825	A	T
Scaffold-42	15847830	C	G
Scaffold-42	15847897	T	C
Scaffold-42	15929965	C	G
Scaffold-42	15966177	T	C
Scaffold-42	15966187	A	C
Scaffold-42	15966241	T	C
Scaffold-42	15977022	A	T
Scaffold-42	16309171	A	T
Scaffold-42	16309179	G	C
Scaffold-42	16309197	GC	CT
Scaffold-42	16309215	C	T
Scaffold-42	16309741	G	A
Scaffold-42	16309744	A	C
Scaffold-42	16309765	ATC	GTA,GTC
Scaffold-42	16309771	A	G
Scaffold-42	16309775	T	C
Scaffold-42	16569883	A	G
Scaffold-42	16693119	C	T
Scaffold-42	16693137	C	A
Scaffold-42	16693142	C	A
Scaffold-42	16693159	G	T
Scaffold-42	16693162	T	C
Scaffold-42	16693189	C	T
Scaffold-42	16693221	A	C,T
Scaffold-42	16693225	C	A

Scaffold-42	16814701	A	G
Scaffold-42	16814738	A	G
Scaffold-42	16948089	A	T
Scaffold-42	16948090	C	T
Scaffold-42	16948101	C	G
Scaffold-42	16948132	G	C
Scaffold-42	16948147	C	G
Scaffold-42	16948158	A	G
Scaffold-42	16948190	T	C
Scaffold-42	16983852	T	C
Scaffold-42	16983871	A	T
Scaffold-42	16983879	GT	GTTATAACT
Scaffold-42	16983906	G	A
Scaffold-42	16983917	A	G
Scaffold-42	16983919	CA	AT
Scaffold-42	16990616	C	T
Scaffold-42	16990629	A	G
Scaffold-42	16990671	G	T
Scaffold-42	16990673	A	G
Scaffold-42	16990702	G	C
Scaffold-42	16990704	A	G
Scaffold-42	17039965	A	G
Scaffold-42	17039983	G	T
Scaffold-42	17040074	G	A
Scaffold-42	17040088	G	T
Scaffold-42	17045148	G	A
Scaffold-42	17045187	A	G
Scaffold-42	17145899	A	G
Scaffold-42	17145902	G	A
Scaffold-42	17145921	G	A
Scaffold-42	17168963	C	T
Scaffold-42	17254227	C	A
Scaffold-42	17254248	T	C
Scaffold-42	17254260	T	C
Scaffold-42	17254266	TGAC	CGAC
Scaffold-42	17280398	T	G
Scaffold-42	17280405	T	C
Scaffold-42	17280422	C	G
Scaffold-42	17280452	T	C
Scaffold-42	17280486	C	A
Scaffold-42	17280498	T	C
Scaffold-42	17721818	T	C
Scaffold-42	17770082	G	A
Scaffold-42	17770165	A	T

Scaffold-42	17770190	A	G
Scaffold-42	17770197	C	G
Scaffold-42	17770198	T	A
Scaffold-42	17828492	C	A
Scaffold-42	17828558	T	A
Scaffold-42	17934396	G	A
Scaffold-42	17934482	A	G
Scaffold-42	17934500	A	C
Scaffold-42	17934515	G	A
Scaffold-42	18034029	C	T
Scaffold-42	18034062	G	T
Scaffold-42	18123054	C	A
Scaffold-42	18123065	C	G
Scaffold-42	18123092	G	A
Scaffold-42	18123098	C	T
Scaffold-42	18123163	GTCA	GA
Scaffold-42	18123171	G	T
Scaffold-42	18123184	G	A
Scaffold-42	18130656	T	A
Scaffold-42	18758705	C	T
Scaffold-42	18758719	A	G
Scaffold-42	18841836	G	C
Scaffold-42	18841868	A	T
Scaffold-42	18841914	T	C
Scaffold-42	18845197	C	T
Scaffold-42	18845296	G	C
Scaffold-42	18846078	G	T
Scaffold-42	18846101	A	C
Scaffold-42	18846103	A	C
Scaffold-42	18954732	C	T
Scaffold-42	18961478	G	C
Scaffold-42	18961500	T	C
Scaffold-42	18961528	C	A
Scaffold-42	18965461	G	A
Scaffold-42	18965499	G	A
Scaffold-42	18965514	T	C
Scaffold-42	18965580	C	T
Scaffold-42	19043672	G	C
Scaffold-42	19043688	T	C
Scaffold-42	19043694	G	T
Scaffold-42	19043702	T	G
Scaffold-42	19043716	T	C
Scaffold-42	19043745	C	G
Scaffold-42	19043762	A	C

Scaffold-42	19043769	G	A
Scaffold-42	19144848	G	A
Scaffold-42	19144875	C	A
Scaffold-42	19144884	T	C
Scaffold-42	19144888	G	A
Scaffold-42	19144890	A	G
Scaffold-42	19165231	A	G
Scaffold-42	19165246	G	C
Scaffold-42	19165333	G	A
Scaffold-42	19267366	G	A
Scaffold-42	19267381	G	T
Scaffold-42	19267398	G	A,C
Scaffold-42	19267428	C	T
Scaffold-42	19267468	G	T
Scaffold-42	19337509	T	A
Scaffold-42	19337533	GC	AA
Scaffold-42	19337566	C	A
Scaffold-42	19337590	T	C
Scaffold-42	19337594	C	A
Scaffold-42	19337632	G	A
Scaffold-42	19337633	G	C
Scaffold-42	19525398	T	C
Scaffold-42	19525411	T	A
Scaffold-42	19525456	T	C
Scaffold-42	19525464	TG	CG,CA
Scaffold-42	19525526	AT	ATAATGTT
Scaffold-42	19551861	A	G
Scaffold-42	19551875	A	G
Scaffold-42	19551878	G	C
Scaffold-42	19551883	T	C
Scaffold-42	19551906	G	C
Scaffold-42	19551941	C	T
Scaffold-42	19551952	G	A
Scaffold-42	19576392	C	T
Scaffold-42	19576420	T	C
Scaffold-42	19576492	G	A
Scaffold-42	19673705	C	A
Scaffold-42	19673719	A	G
Scaffold-42	19673728	A	G
Scaffold-42	19673750	G	A
Scaffold-42	19673754	A	G
Scaffold-42	19673777	C	A
Scaffold-42	19673797	A	T
Scaffold-42	19673801	TTGTC	ATGTT,TTGTT

Scaffold-42	19673813	AATG	AGTG
Scaffold-42	19673817	TT	TC
Scaffold-42	19673838	G	A
Scaffold-42	19785988	G	C
Scaffold-42	19785995	A	G
Scaffold-42	19785998	C	T
Scaffold-42	19786002	TGCAACTAAAAGCG	TTCAACTAAAAGCG
Scaffold-42	19786030	T	C
Scaffold-42	19786063	A	G
Scaffold-42	19786072	C	T
Scaffold-42	19786080	T	C
Scaffold-42	19786086	A	T
Scaffold-42	19841460	CCCG	CCCA,GCCA
Scaffold-42	19841498	C	G
Scaffold-42	19956144	T	C
Scaffold-42	19956170	C	T
Scaffold-42	19956182	T	C
Scaffold-42	19956213	G	C
Scaffold-42	19956236	G	A
Scaffold-42	19956252	C	T
Scaffold-42	20027034	A	G
Scaffold-42	20027054	GAAAAAAAAAAG	GAAAAAAAAAG,GGAAAAAAAAAG,GAAAAAAAAAAAG
Scaffold-42	20027068	ATTA	AA
Scaffold-42	20027073	A	C
Scaffold-42	20027077	A	T
Scaffold-42	20027114	A	G
Scaffold-42	20047697	T	G
Scaffold-42	20047726	G	A
Scaffold-42	20047749	T	A
Scaffold-42	20047824	G	A
Scaffold-42	20106344	C	T
Scaffold-42	20106398	C	A
Scaffold-42	20106437	G	T
Scaffold-42	20106463	C	T
Scaffold-42	20106524	G	A
Scaffold-42	20106575	C	A
Scaffold-42	20106584	G	A
Scaffold-42	20106593	T	C
Scaffold-42	20124773	T	A
Scaffold-42	20209898	C	G
Scaffold-42	20209990	G	T
Scaffold-42	20210026	G	T
Scaffold-42	20242639	AGG	AG

Scaffold-42	20242689	T	C
Scaffold-42	20296098	C	T
Scaffold-42	20296125	TTATAACAACAACA	TTATAACAACA
Scaffold-42	20296150	G	A
Scaffold-42	20332695	A	T
Scaffold-42	20332705	T	G
Scaffold-42	20332727	C	A
Scaffold-42	20332751	T	A
Scaffold-42	20593670	A	G
Scaffold-42	20618590	T	G
Scaffold-42	20618626	T	C
Scaffold-42	20618652	T	G
Scaffold-42	20618663	G	T
Scaffold-42	20618674	C	T
Scaffold-42	20618677	A	G
Scaffold-42	20618693	C	T
Scaffold-42	20618724	C	T
Scaffold-42	20632804	C	G
Scaffold-42	20632806	T	C
Scaffold-42	20632811	C	A
Scaffold-42	20632845	G	C
Scaffold-42	20632852	C	G
Scaffold-42	20789319	C	A
Scaffold-42	20789367	A	T
Scaffold-42	20789370	G	A
Scaffold-42	20789380	A	T
Scaffold-42	20789399	C	G
Scaffold-42	20789426	ATT	AT
Scaffold-42	20884777	T	C
Scaffold-42	20884810	T	C
Scaffold-42	20884817	C	G
Scaffold-42	20884852	A	G
Scaffold-42	20893753	A	T
Scaffold-42	20893772	G	A
Scaffold-42	20893776	C	T
Scaffold-42	20893786	C	T
Scaffold-42	20893811	A	T
Scaffold-42	20893826	T	G
Scaffold-42	20893827	TAA	TA,GAA
Scaffold-42	20893844	C	T
Scaffold-42	20893889	G	T
Scaffold-42	20912692	G	A
Scaffold-42	20912710	A	T
Scaffold-42	20912750	G	A

Scaffold-42	21114478	G	A
Scaffold-42	21114488	G	A
Scaffold-42	21114490	C	T
Scaffold-42	21114491	G	A
Scaffold-42	21114502	A	T
Scaffold-42	21114509	C	A
Scaffold-42	21114527	G	A
Scaffold-42	21114550	T	C
Scaffold-42	21114573	CAA	CAAA,CGA
Scaffold-42	21114581	AGAC	CGAG,CGAC
Scaffold-42	21114586	C	T
Scaffold-42	21158561	A	G
Scaffold-42	21158568	G	A
Scaffold-42	21158584	T	G
Scaffold-42	21182473	C	T
Scaffold-42	21272843	G	A
Scaffold-42	21272880	T	C
Scaffold-42	21376054	C	T
Scaffold-42	21376158	CGACA	CGACG,TGACG
Scaffold-42	21470486	C	G
Scaffold-42	21470487	G	A
Scaffold-42	21470498	T	G,A
Scaffold-42	21470513	C	T
Scaffold-42	21470518	A	C
Scaffold-42	21470523	G	T
Scaffold-42	21470532	G	A
Scaffold-42	21470537	G	T
Scaffold-42	21470538	G	A
Scaffold-42	21470542	T	G
Scaffold-42	21470547	T	G
Scaffold-42	21470569	T	C
Scaffold-42	21470575	T	A
Scaffold-42	21470607	AAACAACA	AAATAACA
Scaffold-42	21589348	T	C
Scaffold-42	21589359	G	A
Scaffold-42	21589401	C	T
Scaffold-42	21589428	C	T
Scaffold-42	21656780	A	G
Scaffold-42	21656804	C	T
Scaffold-42	21778248	C	A
Scaffold-42	21778298	G	A
Scaffold-42	21778328	CTTAGT	CTTACT,CT,CTTGCT
Scaffold-42	21778349	C	A
Scaffold-42	21778357	TAAG	CAAG

Scaffold-42	21778369	T	C
Scaffold-42	21808383	T	C
Scaffold-42	21808385	TATAATA	
Scaffold-42	21808395	ATAT	GTAT
Scaffold-42	21808409	T	A
Scaffold-42	21808412	T	C
Scaffold-42	21808428	GA	AT
Scaffold-42	21808440	A	G
Scaffold-42	21808453	G	A
Scaffold-42	21820906	G	A
Scaffold-42	21821030	G	A
Scaffold-42	21835485	T	C
Scaffold-42	21835499	A	C
Scaffold-42	21835511	T	A
Scaffold-42	21835518	T	A
Scaffold-42	21835528	CGG	CG
Scaffold-42	21835549	G	A
Scaffold-42	21835556	A	G
Scaffold-42	21835990	C	T
Scaffold-42	21836000	A	T
Scaffold-42	21836007	C	G
Scaffold-42	21836025	C	T
Scaffold-42	21836058	A	G
Scaffold-42	21836074	C	T
Scaffold-42	21836097	G	A
Scaffold-42	21836105	A	C
Scaffold-42	21836120	A	G
Scaffold-42	21964555	C	T
Scaffold-42	21964573	G	T
Scaffold-42	21964594	C	A
Scaffold-42	21964606	T	G
Scaffold-42	21964637	G	T
Scaffold-42	21964639	A	C
Scaffold-42	22245787	C	T
Scaffold-42	22245830	C	T
Scaffold-42	22245840	G	A
Scaffold-42	22407400	T	A
Scaffold-42	22407409	G	T
Scaffold-42	22407459	T	A
Scaffold-42	22407464	C	T
Scaffold-42	22407470	C	T
Scaffold-42	22407513	A	C
Scaffold-42	22441991	T	C
Scaffold-42	22442044	A	G

Scaffold-42	22442052	G	A
Scaffold-42	22442058	G	T
Scaffold-42	22442059	C	G
Scaffold-42	22442079	A	G
Scaffold-42	22442106	C	T
Scaffold-42	22669993	GAAAAAT	GAAAAAAT
Scaffold-42	22670031	A	T
Scaffold-42	22753580	A	G
Scaffold-42	22753628	C	G
Scaffold-42	22783819	T	C
Scaffold-42	22783885	G	A
Scaffold-42	22783893	A	C
Scaffold-42	22783903	A	G
Scaffold-42	22783909	C	G
Scaffold-42	22806475	GTTTTTA	GTTTTTTA
Scaffold-42	22806498	T	A
Scaffold-42	22887376	A	G
Scaffold-42	22887389	CAA	AAA
Scaffold-42	22887427	C	A
Scaffold-42	22887445	G	T
Scaffold-42	23034077	A	G
Scaffold-42	23034141	T	C
Scaffold-42	23034147	T	C
Scaffold-42	23034151	ATGA	ATGG,GTGG
Scaffold-42	23034164	C	G
Scaffold-42	23034167	G	C
Scaffold-42	23171237	A	C
Scaffold-42	23171244	C	T
Scaffold-42	23171311	CA	GC,GA
Scaffold-42	23171345	G	A
Scaffold-42	23171366	A	G
Scaffold-42	23171373	A	T
Scaffold-42	23186101	C	A,G
Scaffold-42	23186117	G	C
Scaffold-42	23186132	G	C
Scaffold-42	23186141	T	G
Scaffold-42	23248610	CC	CCCACGTCCAGGTC,CCCATGTC
Scaffold-42	23248615	T	G
Scaffold-42	23248619	AG	AGTTCCGGTTCCGG,AGTTCCGG
Scaffold-42	23248631	G	T
Scaffold-42	23248638	TTTTTTC	CTTTCTT
Scaffold-42	23248694	AGCT	GGCT,GGCG
Scaffold-42	23299390	G	A
Scaffold-42	23299435	G	A

Scaffold-42	23299461	A	G	
Scaffold-42	23299498	C	T	
Scaffold-42	23305759	T	G	
Scaffold-42	23305785	C	A	
Scaffold-42	23305789	A	G	
Scaffold-42	23305795	T	G	
Scaffold-42	23305820	A	G	
Scaffold-42	23384626	G	C	
Scaffold-42	23384637	T	C	
Scaffold-42	23384652	AAT	AAC,TAC	
Scaffold-42	23479273	T	C	
Scaffold-42	23479280	C	T	
Scaffold-42	23479321	GAACAACA		GAACA
Scaffold-42	23479348	G	C	
Scaffold-42	23483992	G	A	
Scaffold-42	23484007	G	T	
Scaffold-42	23484025	G	T	
Scaffold-42	23484028	A	C	
Scaffold-42	23484052	G	A	
Scaffold-42	23492452	TGTCT		TT
Scaffold-42	23492457	TCC	TC	
Scaffold-42	23492490	C	T	
Scaffold-42	23492522	A	T	
Scaffold-42	23492525	C	A	
Scaffold-42	23492556	C	A	
Scaffold-42	23492571	C	G	
Scaffold-42	23492574	A	T	
Scaffold-42	23528611	A	G	
Scaffold-42	23528613	C	A	
Scaffold-42	23528617	C	T	
Scaffold-42	23528623	CTGA	TTGG	
Scaffold-42	23528632	T	C	
Scaffold-42	23528644	C	T	
Scaffold-42	23528663	C	A	
Scaffold-42	23528669	T	A	
Scaffold-42	23528678	T	C	
Scaffold-42	23528683	T	C	
Scaffold-42	23528693	G	A	
Scaffold-42	23528697	C	T	
Scaffold-42	23528728	G	A	
Scaffold-42	23528731	G	A	
Scaffold-42	23704491	G	A	
Scaffold-42	23704515	A	G	
Scaffold-42	23704525	T	G	

Scaffold-42	23723410	G	A	
Scaffold-42	23723412	C	T	
Scaffold-42	23723435	C	G	
Scaffold-42	23755871	C	A	
Scaffold-42	23755919	T	C	
Scaffold-42	23755997	T	G	
Scaffold-42	23840138	CAA	CAAA	
Scaffold-42	23840169	G	A	
Scaffold-42	23840171	G	T	
Scaffold-42	23840182	T	C	
Scaffold-42	23840215	G	A	
Scaffold-42	23840227	GCGAT		GCGAC,CCGAC
Scaffold-42	23840252	A	C	
Scaffold-42	23840271	C	G	
Scaffold-42	23891380	T	A	
Scaffold-42	23972171	G	A	
Scaffold-42	23972206	C	T	
Scaffold-42	23972211	TTGTC		GTATC,GTGTC
Scaffold-42	23972226	TAA	TA	
Scaffold-42	23979998	C	A	
Scaffold-42	23980050	T	C	
Scaffold-42	23980101	C	T	
Scaffold-42	24067827	A	T	
Scaffold-42	24067834	T	C	
Scaffold-42	24067836	A	G	
Scaffold-42	24067866	C	A	
Scaffold-42	24067868	G	A	
Scaffold-42	24067932	G	A	
Scaffold-42	24476031	TG	AA	
Scaffold-42	24476043	G	T	
Scaffold-42	24476074	G	A	
Scaffold-42	24540503	ACC	GCC	
Scaffold-42	24540524	TG	AG	
Scaffold-42	24560947	G	T	
Scaffold-42	24561076	C	T	
Scaffold-42	24561078	C	T	
Scaffold-42	24561080	C	A	
Scaffold-42	24561319	T	C	
Scaffold-42	24561398	T	C	
Scaffold-42	24576014	G	T	
Scaffold-42	24576062	TAAAAAAAAAAG		TTAAAAAAAAAG
Scaffold-42	24689023	A	C	
Scaffold-42	24689027	T	G	
Scaffold-42	24689041	T	A	

Scaffold-42	24689044	T	C
Scaffold-42	24689100	C	G
Scaffold-42	24822581	T	G
Scaffold-42	24822594	C	G
Scaffold-42	24822605	ACTC	AC
Scaffold-42	24928073	A	G
Scaffold-42	24928108	T	C
Scaffold-42	24928136	G	A
Scaffold-42	24950228	T	C
Scaffold-42	24951900	C	A
Scaffold-42	24951908	T	C
Scaffold-42	25048178	G	A
Scaffold-42	25048183	G	C
Scaffold-42	25048186	G	A
Scaffold-42	25048217	GT	GTTTGTTCACT
Scaffold-42	25052851	G	A
Scaffold-42	25204323	C	A
Scaffold-42	25204386	C	A
Scaffold-42	25204431	A	C
Scaffold-42	25204448	C	T
Scaffold-42	25280493	C	A
Scaffold-42	25280584	C	T
Scaffold-42	25338834	G	A
Scaffold-42	25338882	A	G
Scaffold-42	25406527	T	C
Scaffold-42	25406547	C	T
Scaffold-42	25406550	C	T
Scaffold-42	25406559	TTCGG	TTTGG,GTTGA
Scaffold-42	25406568	A	G
Scaffold-42	25406640	C	T
Scaffold-42	25406642	A	G
Scaffold-42	25406647	C	T
Scaffold-42	25414230	T	C
Scaffold-42	25414252	G	A
Scaffold-42	25414269	GGCA	GGCC,AGCC
Scaffold-42	25414281	G	A
Scaffold-42	25414310	A	T
Scaffold-42	25419296	C	T
Scaffold-42	25419320	A	C
Scaffold-42	25419360	T	C
Scaffold-42	25419405	G	T
Scaffold-42	25443990	G	T
Scaffold-42	25443994	T	A
Scaffold-42	25444022	A	G

Scaffold-42	25444068	C	T
Scaffold-42	25444099	T	G
Scaffold-42	25449600	A	C
Scaffold-42	25449639	C	T,A
Scaffold-42	25449642	A	G
Scaffold-42	25449646	T	C
Scaffold-42	25449678	T	A
Scaffold-42	25449706	GAG	GAATAC
Scaffold-42	25449713	C	T
Scaffold-42	25449719	A	G
Scaffold-42	25761863	T	G
Scaffold-42	25836360	G	A
Scaffold-42	25836366	G	A
Scaffold-42	25836417	G	A
Scaffold-42	25836461	T	C
Scaffold-42	26091375	G	A
Scaffold-42	26091430	A	G
Scaffold-42	26091431	A	G
Scaffold-42	26091445	T	G
Scaffold-42	26091479	GTTT	GT
Scaffold-42	26091490	C	A
Scaffold-42	26091509	T	G
Scaffold-42	26158791	G	T
Scaffold-42	26158799	T	C
Scaffold-42	26158866	C	T
Scaffold-42	26158879	G	A
Scaffold-42	26194699	A	G
Scaffold-42	26585895	G	A
Scaffold-42	26585927	G	A
Scaffold-42	26747647	C	T
Scaffold-42	26747673	ATT	ATTT
Scaffold-42	26747679	T	C
Scaffold-42	26747693	AGT	AGGT
Scaffold-42	26785786	C	T
Scaffold-42	26785810	G	C
Scaffold-42	26785814	C	A
Scaffold-42	26785833	G	T
Scaffold-42	26785835	C	T
Scaffold-42	26785842	A	G
Scaffold-42	27008633	G	A
Scaffold-42	27008707	A	T
Scaffold-42	27090302	T	A
Scaffold-42	27243864	C	A
Scaffold-42	27243931	T	G

Scaffold-42	27425088	T	A	
Scaffold-42	27471898	C	A	
Scaffold-42	27471923	A	T	
Scaffold-42	27471939	A	C	
Scaffold-42	27471949	A	C	
Scaffold-42	27477720	G	A	
Scaffold-42	27477724	T	C	
Scaffold-42	27477738	T	C	
Scaffold-42	27477799	GTAT	GT	
Scaffold-42	27477814	C	T	
Scaffold-42	27477820	G	T,C	
Scaffold-42	27477825	G	A	
Scaffold-42	27518938	C	T	
Scaffold-42	27518952	TCCCGAGC		TCCCAAGT
Scaffold-42	27518970	CCGAT		CCGAC,TCGAC
Scaffold-42	27518977	G	A	
Scaffold-42	27518984	C	T	
Scaffold-42	27519002	T	G	
Scaffold-42	27519012	A	G	
Scaffold-42	27519016	A	T	
Scaffold-42	27519022	T	C	
Scaffold-42	27519031	T	C	
Scaffold-42	27519040	T	C	
Scaffold-42	27519048	A	G	
Scaffold-42	27519076	G	A	
Scaffold-42	27747246	T	C	
Scaffold-42	27747299	T	A	
Scaffold-42	27747320	T	G	
Scaffold-42	27747327	G	C	
Scaffold-42	27747348	C	T	
Scaffold-42	27747366	A	G	
Scaffold-42	27747375	T	A	
Scaffold-42	27763480	TATAG		CATAC
Scaffold-42	27763487	C	A	
Scaffold-42	27763488	G	C	
Scaffold-42	27763489	GCG	TCG,TTG,CCA	
Scaffold-42	27763507	C	G	
Scaffold-42	27763538	T	C	
Scaffold-42	27763547	T	G	
Scaffold-42	27763579	TAA	CAG,TAG	
Scaffold-42	27763611	AT	GC,AC	
Scaffold-42	27836114	A	G	
Scaffold-42	27836144	A	G	
Scaffold-42	27836204	T	C	

Scaffold-42	27839971	A	C,T
Scaffold-42	27839975	C	T
Scaffold-42	27839981	T	C
Scaffold-42	27960625	C	T
Scaffold-42	27960647	A	T
Scaffold-42	27960650	A	C
Scaffold-42	27960656	G	C
Scaffold-42	27960682	TAA	TAAA
Scaffold-42	27960692	TTCCAGT	TT,TTCTAGT
Scaffold-42	27961125	G	A
Scaffold-42	27961161	A	G
Scaffold-42	27963741	C	A
Scaffold-42	27963742	G	A
Scaffold-42	27963766	T	G
Scaffold-42	27963799	T	C
Scaffold-42	28125676	A	G
Scaffold-42	28125698	C	T
Scaffold-42	28125716	A	C
Scaffold-42	28125720	T	C
Scaffold-42	28125723	C	A
Scaffold-42	28125747	G	A
Scaffold-42	28130114	G	A
Scaffold-42	28130132	A	G
Scaffold-42	28130157	T	C
Scaffold-42	28130161	A	C
Scaffold-42	28130170	G	C
Scaffold-42	28130173	CAAAG	AAAAA
Scaffold-42	28130215	A	T
Scaffold-42	28309303	A	T
Scaffold-42	28309322	T	C
Scaffold-42	28309342	ATAT	ATTC,GTTC
Scaffold-42	28309351	T	G
Scaffold-42	28536062	ATTTTTTTTG	ATTTTTGGTG,ATTTTTTTCG
Scaffold-42	28536072	TG	TA
Scaffold-42	28536096	T	C
Scaffold-42	28536116	CAGA	CACA
Scaffold-42	28536134	A	G
Scaffold-42	28536136	C	G
Scaffold-42	28536150	C	T
Scaffold-42	28724594	A	C,G
Scaffold-42	28724607	AAA	AAG,CAG
Scaffold-42	28724611	T	C
Scaffold-42	28724614	G	T
Scaffold-42	28724626	A	T

Scaffold-42	28724639	T	C	
Scaffold-42	28724654	C	G	
Scaffold-42	28724670	T	G	
Scaffold-42	28725526	A	T,C	
Scaffold-42	28725544	G	A	
Scaffold-42	28725553	C	T	
Scaffold-42	28725577	A	T	
Scaffold-42	28725586	G	A	
Scaffold-42	28725592	G	A	
Scaffold-42	28725613	G	T	
Scaffold-42	28725623	G	A	
Scaffold-42	28725650	A	G	
Scaffold-42	28729381	A	T	
Scaffold-42	28729441	A	T	
Scaffold-42	28729497	G	A	
Scaffold-42	28729502	G	C	
Scaffold-42	28955590	C	T	
Scaffold-42	28959373	G	A	
Scaffold-42	28959379	G	T	
Scaffold-42	28959396	A	T	
Scaffold-42	28959398	C	T	
Scaffold-42	28959414	G	T	
Scaffold-42	29057360	T	C	
Scaffold-42	29057398	C	A	
Scaffold-42	29057416	A	G	
Scaffold-42	29057427	T	G	
Scaffold-42	29265567	C	T	
Scaffold-42	29265579	T	C	
Scaffold-42	29337560	C	A	
Scaffold-42	29337599	T	C	
Scaffold-42	29337628	A	G	
Scaffold-42	29337669	A	G	
Scaffold-42	29652415	A	G	
Scaffold-42	29652421	TGA	TGGA	
Scaffold-42	29652425	C	T	
Scaffold-42	29652502	G	C	
Scaffold-42	29657652	G	A	
Scaffold-42	29657691	G	A	
Scaffold-42	29657757	C	T	
Scaffold-42	29709106	G	A	
Scaffold-42	29721037	A	G	
Scaffold-42	29721061	A	C	
Scaffold-42	29721092	ACCCCCA		ACCCCA
Scaffold-42	29721112	A	T	

Scaffold-42	29721114	CTT	CT
Scaffold-42	29721130	A	G
Scaffold-42	29721152	CTTTATAGT	CTCTATAGT,CTTTATAGTTTATAGT
Scaffold-42	29837002	G	T
Scaffold-42	29837011	C	T
Scaffold-42	29837083	G	T
Scaffold-42	29868004	ATATTATTATA	ATATTATA
Scaffold-42	29868041	A	G
Scaffold-42	29868047	G	A
Scaffold-42	29868093	TA	TGCA
Scaffold-42	29884879	A	T
Scaffold-42	29884926	TGT	TGC,GGC
Scaffold-42	29884941	A	T
Scaffold-42	29992876	T	C
Scaffold-42	29992889	TGT	TT
Scaffold-42	29992915	C	T
Scaffold-42	29992920	T	C
Scaffold-42	29992926	A	G
Scaffold-42	29992934	TTTAGCT	TT
Scaffold-42	29992960	G	A
Scaffold-42	29992966	T	A
Scaffold-42	30198019	C	T
Scaffold-42	30198056	T	C
Scaffold-42	30213324	A	G
Scaffold-42	30213325	T	C
Scaffold-42	30213378	G	T
Scaffold-42	30213400	A	G
Scaffold-42	30213437	G	T
Scaffold-42	30231195	C	A
Scaffold-42	30231197	C	G
Scaffold-42	30231273	AATA	AA
Scaffold-42	30231284	G	A
Scaffold-42	30231333	G	A
Scaffold-42	30299501	C	T
Scaffold-42	30299514	TT	TCT
Scaffold-42	30299541	G	A,T
Scaffold-42	30299551	T	G
Scaffold-42	30299569	A	G
Scaffold-42	30299578	GCA	GA
Scaffold-42	30299589	C	G
Scaffold-42	30299594	TAAAT	TAAAG,CAAAG
Scaffold-42	30299608	C	T
Scaffold-42	30299618	T	A
Scaffold-42	30332544	CAAG	AAAT,AAAG

Scaffold-42	30332559	AATAGTAAGACCAAATCTTAT	AATAGTAAGACCAAATCTTAC,AAAAGTAAGACCAAATCTTAC
Scaffold-42	30332608	G	T
Scaffold-42	30332612	T	A
Scaffold-42	30332623	A	C
Scaffold-42	30332633	G	A
Scaffold-42	30332661	C	T
Scaffold-42	30332675	A	T
Scaffold-42	30538917	G	A
Scaffold-42	30765694	A	G
Scaffold-42	30765727	A	G
Scaffold-42	30765762	C	T
Scaffold-42	30765810	A	G
Scaffold-42	30803188	G	A
Scaffold-42	30803232	A	G
Scaffold-42	30803235	T	A
Scaffold-42	30803271	A	G
Scaffold-42	30803293	A	G
Scaffold-42	30803308	AGG	AG
Scaffold-42	30803316	C	T
Scaffold-42	30803788	C	T
Scaffold-42	30803864	G	A
Scaffold-42	30803873	T	C
Scaffold-42	30803876	TTAATTTTGG	TG
Scaffold-42	30803889	A	G
Scaffold-42	30803892	A	G
Scaffold-42	30803916	T	C
Scaffold-42	30998667	T	C
Scaffold-42	30998677	A	C
Scaffold-42	30998727	T	C
Scaffold-42	30998779	C	T
Scaffold-42	31141218	A	G
Scaffold-42	31479888	T	C
Scaffold-42	31479916	C	T
Scaffold-42	31479917	G	A
Scaffold-42	31479945	C	T
Scaffold-42	31480003	A	G
Scaffold-42	31596377	T	G,C
Scaffold-42	31596461	A	G
Scaffold-42	31596488	G	A
Scaffold-42	31600839	A	G
Scaffold-42	31600864	T	A
Scaffold-42	31600883	TCCGCCCCTCCTCCCTCTCC	TCTGCTCCTCCTCTCTCTCC,TCCGCCCCTCCCCCCTCTCC,TCCGCTCCTCCTCCCT

CTCC,TCCGCCCCTCCTCCCTCTTC

Scaffold-42	31600905	CAC	CC	
Scaffold-42	31600908	C	T	
Scaffold-42	31600913	G	A	
Scaffold-42	31600940	T	A	
Scaffold-42	31600944	T	A	
Scaffold-42	31600949	G	T	
Scaffold-42	31600964	A	G	
Scaffold-42	31600968	G	C	
Scaffold-42	31600969	C	T	
Scaffold-42	31730160	T	C	
Scaffold-42	31730209	A	C	
Scaffold-42	31730221	T	G	
Scaffold-42	31730224	A	G	
Scaffold-42	31730272	G	A	
Scaffold-42	31730290	C	T	
Scaffold-42	31730860	G	T	
Scaffold-42	31768191	CA	CG	
Scaffold-42	31768214	G	A	
Scaffold-42	31768291	TCC	TC	
Scaffold-42	31795211	T	C	
Scaffold-42	31795224	T	G	
Scaffold-42	31795246	GTATATAT		GTATATATAT
Scaffold-42	31904503	C	A	
Scaffold-42	31977742	T	A	
Scaffold-42	31977752	C	T	
Scaffold-42	31977753	T	C	
Scaffold-42	31977779	A	G	
Scaffold-42	32034705	T	C	
Scaffold-42	32034706	A	G	
Scaffold-42	32350351	CTTGT		CTTGTT
Scaffold-42	32350378	A	G	
Scaffold-42	32467648	A	G	
Scaffold-42	32474631	T	C	
Scaffold-42	32474652	T	C	
Scaffold-42	32589243	C	T	
Scaffold-42	32589325	A	G	
Scaffold-42	32751176	G	C	
Scaffold-42	32751228	T	C	
Scaffold-42	32929248	T	A	
Scaffold-42	32929254	T	G	
Scaffold-42	32929264	T	A	
Scaffold-42	32929271	CATA	TATT	
Scaffold-42	32929285	T	A	

Scaffold-42	32929296	ATAAACAATTCAAGAAATCA	CTAAACAATTCAAGAAATCA,AA,ATAAACAATTCAAGAGATCA
Scaffold-42	32929331	A	G
Scaffold-42	32929363	G	A
Scaffold-42	32942433	G	T
Scaffold-42	32942466	T	A
Scaffold-42	32942475	ACTTCC	AC
Scaffold-42	32942496	C	G
Scaffold-42	32970930	G	T
Scaffold-42	32970971	A	G
Scaffold-42	32970983	C	A
Scaffold-42	32970997	C	G
Scaffold-42	32971021	T	G
Scaffold-42	32971032	AATA	AA
Scaffold-42	32971057	G	A
Scaffold-42	32975753	A	T
Scaffold-42	32975792	A	G
Scaffold-42	33005159	CAAAAAAAG	CAAAAAAAAG,CAAAAAAAAAG
Scaffold-42	33005186	AG	AC
Scaffold-42	33005205	A	G
Scaffold-42	33005206	G	C
Scaffold-42	33057821	T	C
Scaffold-42	33068441	C	T
Scaffold-42	33068474	G	C
Scaffold-42	33144853	G	A
Scaffold-42	33144868	A	G
Scaffold-42	33144888	G	A
Scaffold-42	33144897	A	T
Scaffold-42	33144907	C	A
Scaffold-42	33144910	G	A
Scaffold-42	33144948	T	C
Scaffold-42	33144969	G	T
Scaffold-42	33144991	G	A
Scaffold-42	33235972	G	T,A
Scaffold-42	33412733	A	G
Scaffold-42	33412738	A	G
Scaffold-42	33412744	A	T
Scaffold-42	33412762	CAT	CT
Scaffold-42	33412783	C	T
Scaffold-42	33412789	G	C
Scaffold-42	33412805	T	C
Scaffold-42	33412817	ATT	AT
Scaffold-42	33412829	A	G
Scaffold-42	33455079	T	C

Scaffold-42	33455112	C	T
Scaffold-42	33563052	G	A
Scaffold-42	33908074	T	G
Scaffold-42	33908079	C	A
Scaffold-42	33908106	C	T
Scaffold-42	33908114	GC	GT,AT
Scaffold-42	33908163	C	T
Scaffold-42	33908166	G	A
Scaffold-42	33971431	T	C
Scaffold-42	33971471	A	G
Scaffold-42	34095579	C	T
Scaffold-42	34095580	G	A
Scaffold-42	34095584	C	T
Scaffold-42	34095610	G	A
Scaffold-42	34095614	G	C
Scaffold-42	34095628	G	A
Scaffold-42	34095630	T	G
Scaffold-42	34095656	C	T
Scaffold-42	34095672	A	G
Scaffold-42	34106788	C	T
Scaffold-42	34147051	T	A
Scaffold-42	34147054	C	A
Scaffold-42	34147055	TG	CA
Scaffold-42	34147107	AGCCCGTTG	AG,AGCCTGTTG,AGCCCATTG
Scaffold-42	34147121	A	C
Scaffold-42	34147138	C	T
Scaffold-42	34147157	G	A
Scaffold-42	34147182	C	T
Scaffold-42	34159645	G	C
Scaffold-42	34159661	G	A
Scaffold-42	34159673	T	C
Scaffold-42	34159680	CGGTGATGGTGGTG	CGGTAATGGTGGTG,CGGTGATGTTGGTG
Scaffold-42	34159710	T	A
Scaffold-42	34159727	G	C
Scaffold-42	34159730	GTA	GTT,TTT
Scaffold-42	34159765	G	A
Scaffold-42	34159770	G	A
Scaffold-42	34177419	T	C
Scaffold-42	34177464	T	C
Scaffold-42	34177475	CATCC	TATCC
Scaffold-42	34205825	GA	TG
Scaffold-42	34205908	C	T
Scaffold-42	34205911	C	T,G

Scaffold-42	34465414	A	G
Scaffold-42	34465445	C	T
Scaffold-42	34465511	G	T
Scaffold-42	34523203	A	G
Scaffold-42	34523270	G	A
Scaffold-42	34593565	TC	AA
Scaffold-42	34593588	A	C
Scaffold-42	34593592	A	C
Scaffold-42	34593593	T	A
Scaffold-42	34593612	C	T
Scaffold-42	34713200	G	A
Scaffold-42	34713215	A	C
Scaffold-42	34713229	GC	AT
Scaffold-42	34713242	A	G
Scaffold-42	34713248	A	G
Scaffold-42	34713254	G	A
Scaffold-42	34713262	GTTCAACT	CTTCCACC,CTTCGACC
Scaffold-42	34713275	C	T
Scaffold-42	34713284	C	A
Scaffold-42	34750707	G	T
Scaffold-42	34750775	C	T
Scaffold-42	34906065	G	A
Scaffold-42	34906074	A	G
Scaffold-42	34906086	CC	CG
Scaffold-42	34906131	TGG	TGGG
Scaffold-42	34906135	C	A
Scaffold-42	34906151	T	A
Scaffold-42	34906162	C	T
Scaffold-42	34906163	C	A
Scaffold-42	34906175	T	G
Scaffold-42	34906189	G	A
Scaffold-42	34906191	T	C
Scaffold-42	35009207	A	G
Scaffold-42	35009231	A	G
Scaffold-42	35009292	A	C
Scaffold-42	35117728	C	G
Scaffold-42	35134446	G	T
Scaffold-42	35134460	CAA	CAAA
Scaffold-42	35134477	A	G
Scaffold-42	35134560	T	A
Scaffold-42	35134564	G	A
Scaffold-42	35134568	A	G
Scaffold-42	35166499	T	C
Scaffold-42	35166536	A	G

Scaffold-42	35372537	G	C
Scaffold-42	35734563	T	A
Scaffold-42	35734583	T	G
Scaffold-42	35734596	T	C
Scaffold-42	35734619	T	C
Scaffold-42	35734625	G	A
Scaffold-42	35734657	A	G
Scaffold-42	35775161	T	C
Scaffold-42	35775179	T	C
Scaffold-42	35775212	GATTACA	GA
Scaffold-42	35775258	A	G
Scaffold-42	35775275	CAGCTAGCTAGCTA	CAGCTAGCTA
Scaffold-42	35775294	T	A
Scaffold-42	35786181	T	A
Scaffold-42	35786206	T	C
Scaffold-42	35786257	A	G
Scaffold-42	35786260	G	A
Scaffold-42	35786417	C	T
Scaffold-42	35786443	C	T
Scaffold-42	35786497	G	C
Scaffold-42	35878386	T	A
Scaffold-42	35878408	T	A
Scaffold-42	35878430	A	G
Scaffold-42	35878476	A	T
Scaffold-42	35878484	T	C
Scaffold-42	35878516	A	G,T
Scaffold-42	35878521	C	G
Scaffold-42	36065375	T	A
Scaffold-42	36065488	A	T
Scaffold-42	36150951	G	C
Scaffold-42	36150987	G	T
Scaffold-42	36550553	T	A
Scaffold-42	36550587	A	G,C
Scaffold-42	36550595	A	G
Scaffold-42	36550602	C	G
Scaffold-42	36550632	G	A
Scaffold-42	36550641	A	G
Scaffold-42	36550651	A	G
Scaffold-42	36579904	A	T
Scaffold-42	36579911	C	T
Scaffold-42	36579970	A	G
Scaffold-42	36579971	C	T
Scaffold-42	36579977	A	G
Scaffold-42	36579982	A	G

Scaffold-42	36580010	C	A
Scaffold-42	36580020	ATA	GTG
Scaffold-42	36580028	A	G
Scaffold-42	36643603	AACAGGTTGTG	AACAGGTGGTG
Scaffold-42	36643627	G	T
Scaffold-42	36643634	C	G
Scaffold-42	36643693	G	A
Scaffold-42	36643701	T	C
Scaffold-42	36643702	A	G
Scaffold-42	36643705	ATCTCTCTCTCTCA	ATCTCTCTCTCTCT,ATCTCTCTCTCACA,ATCTCTCTCTCACG
Scaffold-42	36643719	CACA	CTCA,CTCT
Scaffold-42	36643728	A	T
Scaffold-42	36643730	A	T
Scaffold-42	36647445	C	A
Scaffold-42	36647475	A	G
Scaffold-42	36647476	A	C
Scaffold-42	36647485	AA	AC,GT
Scaffold-42	36713930	T	G,C
Scaffold-42	36713945	TC	TG,AG
Scaffold-42	36713960	G	A
Scaffold-42	36713988	AGC	TGT,AGT
Scaffold-42	36714013	C	A
Scaffold-42	36714037	A	G
Scaffold-42	36759025	A	G
Scaffold-42	36759047	CAAAAAAAAT	CAATAAAAAT,CAAAAAAAAAT
Scaffold-42	36759092	T	C
Scaffold-42	36783605	GAAAAAAG	GAAAAAAGG
Scaffold-42	36783648	G	A
Scaffold-42	36794003	G	A
Scaffold-42	36794013	C	T
Scaffold-42	36813898	A	G
Scaffold-42	36813904	G	A
Scaffold-42	36813929	G	T
Scaffold-42	36813960	G	T
Scaffold-42	36813971	ATT	ATTT
Scaffold-42	36813975	A	T
Scaffold-42	36813980	T	C
Scaffold-42	36860386	T	A
Scaffold-42	36860411	T	C
Scaffold-42	36936583	T	C
Scaffold-42	36936622	G	A
Scaffold-42	36961747	T	C
Scaffold-42	36961775	C	A

Scaffold-42	36961777	TACAACAACAACAA		
TACAACAACAAGAA,TACAACAACAA				
Scaffold-42	36961804	T	C	
Scaffold-42	37021692	T	G	
Scaffold-42	37021702	T	A	
Scaffold-42	37021712	C	A	
Scaffold-42	37021717	C	A	
Scaffold-42	37021741	T	A	
Scaffold-42	37021755	C	T	
Scaffold-42	37025983	C	A	
Scaffold-42	37025985	C	G	
Scaffold-42	37025990	G	T	
Scaffold-42	37025992	G	A	
Scaffold-42	37026011	C	T	
Scaffold-42	37026066	A	G	
Scaffold-42	37026072	C	T	
Scaffold-42	37026075	TG	CG,CC	
Scaffold-42	37026080	C	T	
Scaffold-42	37082690	TGAGC		CGAGG
Scaffold-42	37082700	T	A	
Scaffold-42	37082719	C	T	
Scaffold-42	37082739	CAAT	TAAC	
Scaffold-42	37082757	T	C	
Scaffold-42	37082769	G	A	
Scaffold-42	37082775	T	C	
Scaffold-42	37082780	TAGTATG		GAATATA
Scaffold-42	37082792	A	G	
Scaffold-42	37082801	A	G	
Scaffold-42	37082805	A	G	
Scaffold-42	37082806	T	C	
Scaffold-42	37095996	C	G	
Scaffold-42	37096002	C	T	
Scaffold-42	37096040	G	C	
Scaffold-42	37096075	T	C	
Scaffold-42	37121351	A	G	
Scaffold-42	37121363	G	A	
Scaffold-42	37121423	C	A,T	
Scaffold-42	37121447	A	G	
Scaffold-42	37121448	C	A	
Scaffold-42	37175850	A	G	
Scaffold-42	37175853	T	C	
Scaffold-42	37175872	C	T	
Scaffold-42	37175909	CCTTCTTA		CCTTA
Scaffold-42	37181295	G	T	

Scaffold-42	37181364	G	C
Scaffold-42	37181399	C	T
Scaffold-42	37181431	G	A
Scaffold-42	37248601	A	C
Scaffold-42	37248604	TT	TTGTTGCCT
Scaffold-42	37248610	G	A
Scaffold-42	37267070	CATTG	CG
Scaffold-42	37267076	A	G
Scaffold-42	37267093	C	A,G
Scaffold-42	37267103	G	A
Scaffold-42	37267161	C	T
Scaffold-42	37267167	T	C
Scaffold-42	37267198	G	C
Scaffold-42	37267215	G	T
Scaffold-42	37267247	C	G
Scaffold-42	37267255	G	A
Scaffold-42	37267258	C	G
Scaffold-42	37438861	CT	TC,GT
Scaffold-42	37438885	C	T
Scaffold-42	37438893	G	A
Scaffold-42	37438899	T	C
Scaffold-42	37438913	C	T
Scaffold-42	37438932	T	A
Scaffold-42	37438934	T	G,C
Scaffold-42	37438936	A	C
Scaffold-42	37438946	G	T
Scaffold-42	37438949	G	C,A
Scaffold-42	37438956	A	G
Scaffold-42	37438982	C	T
Scaffold-42	37438985	G	A
Scaffold-42	37489230	C	A
Scaffold-42	37546183	G	C
Scaffold-42	37546213	G	A
Scaffold-42	37546270	T	G
Scaffold-42	37546274	G	C
Scaffold-42	37703630	G	A
Scaffold-42	37703653	T	A
Scaffold-42	37703731	T	A
Scaffold-42	37703733	T	C
Scaffold-42	37703762	T	C
Scaffold-42	38008869	C	T
Scaffold-42	38008897	CAAT	TAAT
Scaffold-42	38008909	G	A
Scaffold-42	38008965	G	A

Scaffold-42	38016755	C	T
Scaffold-42	38016785	T	C
Scaffold-42	38016803	C	T
Scaffold-42	38016812	G	C
Scaffold-42	38016818	A	G
Scaffold-42	38016843	G	A
Scaffold-42	38048317	G	A
Scaffold-42	38048335	A	G
Scaffold-42	38048408	G	A
Scaffold-42	38048429	ACCA	ACCG
Scaffold-42	38076067	A	G
Scaffold-42	38076089	G	A
Scaffold-42	38076098	A	G
Scaffold-42	38076138	A	C
Scaffold-42	38076151	A	G
Scaffold-42	38076157	A	C
Scaffold-42	38218875	A	C
Scaffold-42	38218887	T	G
Scaffold-42	38443267	A	G
Scaffold-42	38443273	G	A
Scaffold-42	38443324	T	C
Scaffold-42	38443368	A	G
Scaffold-42	38443405	T	C
Scaffold-42	38636207	T	A
Scaffold-42	38722635	G	C
Scaffold-42	38725171	T	C
Scaffold-42	38725173	G	T
Scaffold-42	38725200	G	A
Scaffold-42	38725233	AGTGTGTGTGTGTA	AGTGTGTGTGTA,AGTGTGTGTGTATA,AGAGTGTGTGTGTA
Scaffold-42	38820163	G	C
Scaffold-42	38820168	G	A
Scaffold-42	38820190	C	A
Scaffold-42	38975811	A	C
Scaffold-42	38975812	A	G
Scaffold-42	38975896	G	A
Scaffold-42	38975915	A	G
Scaffold-42	38975933	C	G
Scaffold-42	38975941	C	T
Scaffold-42	38980264	A	C
Scaffold-42	38980278	G	A
Scaffold-42	38980303	C	T
Scaffold-42	38980327	T	C
Scaffold-42	38980346	G	A

Scaffold-42	38980356	C	T
Scaffold-42	38980380	A	G
Scaffold-42	38980381	C	G
Scaffold-42	38980382	T	C
Scaffold-42	39017810	C	G
Scaffold-42	39017823	G	A
Scaffold-42	39173230	C	T
Scaffold-42	39251744	A	G
Scaffold-42	39251847	A	G
Scaffold-42	39290773	C	A
Scaffold-42	39290873	G	C
Scaffold-42	39318902	A	G
Scaffold-42	39318936	GAAAGTCAAAGTCA	GAAAGTCAAAGTCAAAGTCA,GAAAGTCAAAGA
Scaffold-42	39318973	ATACTACA	ATACA
Scaffold-42	39319029	GCA	GTA
Scaffold-42	39386685	T	A
Scaffold-42	39391571	T	C
Scaffold-42	39391619	G	C
Scaffold-42	39391623	ACC	ACCC
Scaffold-42	39391631	C	G
Scaffold-42	39391644	C	T
Scaffold-42	39391670	TCT	TCC
Scaffold-42	39391679	G	T
Scaffold-42	39391698	C	T
Scaffold-42	39425632	C	T
Scaffold-42	39425719	TC	CC,CG
Scaffold-42	39425724	A	G
Scaffold-42	39425731	C	T
Scaffold-42	39478357	T	C
Scaffold-42	39478368	T	C
Scaffold-42	39478374	A	G
Scaffold-42	39478384	A	T
Scaffold-42	39478397	C	A
Scaffold-42	39478450	TAAAAAC	TAAAC,TCAAAAC
Scaffold-42	39553788	T	A
Scaffold-42	39553811	A	C
Scaffold-42	39553820	C	A
Scaffold-42	39553873	C	T
Scaffold-42	39553891	C	G
Scaffold-42	39630723	G	A
Scaffold-42	39630733	C	T
Scaffold-42	39630774	T	C
Scaffold-42	39630810	C	T

Scaffold-42	39667273	C	T
Scaffold-42	39667317	T	C
Scaffold-42	39667323	C	A
Scaffold-42	39667337	A	G
Scaffold-42	39667352	T	G
Scaffold-42	39667361	T	C
Scaffold-42	39698070	CA	TA
Scaffold-42	39698187	C	T
Scaffold-42	39840940	A	T
Scaffold-42	39840972	T	C
Scaffold-42	39840992	TAGAA	TAGAAGAA
Scaffold-42	39840998	T	A
Scaffold-42	39841015	G	C
Scaffold-42	39841036	GA	AA,AT
Scaffold-42	40115381	T	C
Scaffold-42	40115388	CT	CC,AC
Scaffold-42	40115455	A	T
Scaffold-42	40143956	C	T
Scaffold-42	40144003	C	T
Scaffold-42	40144057	T	G
Scaffold-42	40183437	G	A
Scaffold-42	40183471	G	C
Scaffold-42	40198161	C	T
Scaffold-42	40243929	A	G
Scaffold-42	40243939	G	T
Scaffold-42	40243980	G	T
Scaffold-42	40244000	G	A
Scaffold-42	40244055	T	C
Scaffold-42	40307894	T	C
Scaffold-42	40307931	TGGGGGT	TGGGGT
Scaffold-42	40307960	A	T
Scaffold-42	40307970	G	A
Scaffold-42	40310860	C	T
Scaffold-42	40310922	TGCA	TGCGCA
Scaffold-42	40310940	GTTACT	GT
Scaffold-42	40310950	T	C
Scaffold-42	40310965	C	A
Scaffold-42	40380262	C	T
Scaffold-42	40380283	T	C
Scaffold-42	40380308	G	C
Scaffold-42	40380337	T	C
Scaffold-42	40380372	C	T
Scaffold-42	40380376	A	G
Scaffold-42	40396366	T	C

Scaffold-42	40396379	T	C
Scaffold-42	40396401	T	C
Scaffold-42	40493346	A	G
Scaffold-42	40493367	TGAA	AGAA,AGAG
Scaffold-42	40493455	G	T
Scaffold-42	40493461	G	A
Scaffold-42	40493466	A	G
Scaffold-42	40493472	A	C
Scaffold-42	40599555	C	T
Scaffold-42	40599579	T	C
Scaffold-42	40599588	G	A
Scaffold-42	40599619	G	T
Scaffold-42	40599630	T	C
Scaffold-42	40599632	A	T
Scaffold-42	40599657	C	A
Scaffold-42	40599659	C	A
Scaffold-42	40599669	A	C
Scaffold-42	40709140	T	C
Scaffold-42	40709224	C	T
Scaffold-42	40709601	C	A
Scaffold-42	40709634	T	G
Scaffold-42	40709638	CAA	CA,CAAA
Scaffold-42	40709643	T	G
Scaffold-42	40709662	A	T
Scaffold-42	40709681	C	A
Scaffold-42	40709696	C	G
Scaffold-42	40964095	A	G
Scaffold-42	40966209	GTG	GTTG
Scaffold-42	40966244	T	G
Scaffold-42	40966248	G	T
Scaffold-42	40966281	A	C
Scaffold-42	40966285	GAAAAAC	GAAAAC
Scaffold-42	41035427	A	T
Scaffold-42	41035465	ATGT	GTGT,GTGC
Scaffold-42	41035505	T	C
Scaffold-42	41035513	T	C
Scaffold-42	41094898	C	T
Scaffold-42	41094929	A	G
Scaffold-42	41094965	C	T
Scaffold-42	41304726	A	C
Scaffold-42	41304734	C	T
Scaffold-42	41304745	T	A
Scaffold-42	41304770	TACAAACAAA	TACAAA
Scaffold-42	41308995	C	T

Scaffold-42	41435193	C	T
Scaffold-42	41435236	C	T
Scaffold-42	41632301	G	A
Scaffold-42	41632367	G	C
Scaffold-42	41845028	G	A
Scaffold-42	41845062	C	A
Scaffold-42	41845094	TACACACACC	TACACACC
Scaffold-42	41845144	G	A
Scaffold-42	41876767	G	A
Scaffold-42	41876843	T	C
Scaffold-42	41880645	T	C
Scaffold-42	41880653	G	A
Scaffold-42	41880688	C	T
Scaffold-42	41880724	A	G
Scaffold-42	41945841	C	T
Scaffold-42	41945893	C	T
Scaffold-42	41945910	ACC	AC
Scaffold-42	41945929	A	T
Scaffold-42	41945945	T	G
Scaffold-42	41945954	A	G
Scaffold-42	41945981	G	A
Scaffold-42	42071752	G	C
Scaffold-42	42071775	G	C
Scaffold-42	42071780	T	A
Scaffold-42	42093419	CTC	CTTC
Scaffold-42	42093422	A	T
Scaffold-42	42093433	A	T
Scaffold-42	42093466	C	T
Scaffold-42	42093509	A	C
Scaffold-42	42093520	C	T
Scaffold-42	42263460	G	C
Scaffold-42	42263527	G	A
Scaffold-42	42263533	C	A
Scaffold-42	42396015	C	T
Scaffold-42	42396020	ATTTTTTTA	ATTTTTTTTA,ATTTTTTTTTA
Scaffold-42	42396043	A	C
Scaffold-42	42396072	A	C
Scaffold-42	42396095	T	C
Scaffold-42	42396104	C	T
Scaffold-42	42396105	A	T
Scaffold-42	42396106	C	T
Scaffold-42	42396112	A	G
Scaffold-42	42571338	G	A
Scaffold-42	42571364	T	C

Scaffold-42	42571467	C	G
Scaffold-42	42743985	C	T
Scaffold-42	42744799	ATA	AA
Scaffold-42	42769614	C	G
Scaffold-42	42769638	A	T
Scaffold-42	42769639	C	T
Scaffold-42	42769677	C	T
Scaffold-42	42769678	A	T
Scaffold-42	42937804	CAT	AAT
Scaffold-42	42937817	AATATGT	TATTTGT
Scaffold-42	42937844	C	T
Scaffold-42	42937857	GAG	GAA,AAA
Scaffold-42	42937889	A	C
Scaffold-42	42937895	T	G
Scaffold-42	42937912	T	G
Scaffold-42	42937915	T	C
Scaffold-42	42937925	AACTTA	AATTTA,AA
Scaffold-42	42937935	T	G
Scaffold-42	43032693	C	T
Scaffold-42	43042630	TT	CA
Scaffold-42	43042689	AGG	AG
Scaffold-42	43042692	GTTTTTTTA	GTTTTTTTG
Scaffold-42	43042715	C	T
Scaffold-42	43042741	GTT	GTTT
Scaffold-42	43042745	G	T
Scaffold-42	43059946	G	A
Scaffold-42	43059955	C	T
Scaffold-42	43279457	A	C
Scaffold-42	43279462	C	T
Scaffold-42	43279502	G	A
Scaffold-42	43279528	C	T
Scaffold-42	43279535	C	A
Scaffold-42	43279545	ATTATGT	AT
Scaffold-42	43279559	A	G
Scaffold-42	43279569	T	C
Scaffold-42	43305598	A	G
Scaffold-42	43305618	G	T
Scaffold-42	43305675	C	T
Scaffold-42	43447534	G	A
Scaffold-42	43447545	C	A
Scaffold-42	43447546	T	C
Scaffold-42	43447565	T	C
Scaffold-42	43447608	C	T
Scaffold-42	43447640	T	C

Scaffold-42	43447646	A	G
Scaffold-42	43447651	G	A
Scaffold-42	43447669	C	A
Scaffold-42	43679602	C	T
Scaffold-42	43912825	A	G
Scaffold-42	43912826	T	A
Scaffold-42	43912827	C	A
Scaffold-42	43912844	C	A
Scaffold-42	43912846	G	A
Scaffold-42	43912856	A	T
Scaffold-42	43912867	C	T
Scaffold-42	43912874	G	A
Scaffold-42	43912941	T	C
Scaffold-42	44061738	C	A
Scaffold-42	44061748	G	A
Scaffold-42	44061757	ACCCC	ACCCA
Scaffold-42	44061775	C	T
Scaffold-42	44061779	A	G
Scaffold-42	44061791	G	A
Scaffold-42	44061813	C	G,A
Scaffold-42	44061816	C	G
Scaffold-42	44061823	C	G
Scaffold-42	44061849	T	A
Scaffold-42	44061862	AACCA	AACCC
Scaffold-42	44257679	T	C
Scaffold-42	44257710	A	G
Scaffold-42	44257721	T	C
Scaffold-42	44307797	A	G
Scaffold-42	44312441	T	C
Scaffold-42	44312449	C	T
Scaffold-42	44312492	A	G
Scaffold-42	44313221	C	T
Scaffold-42	44313233	T	A
Scaffold-42	44392024	C	T
Scaffold-42	44392087	A	G
Scaffold-42	44503865	T	C
Scaffold-42	44503878	CAAAAAAAT	CAAAAAAAAT
Scaffold-42	44503902	A	C
Scaffold-42	44503967	CAAT	GAAT,GAAA
Scaffold-42	44503979	T	A
Scaffold-42	44565555	A	C
Scaffold-42	44565596	G	A
Scaffold-42	44771318	A	T
Scaffold-42	44989304	C	T

Scaffold-42	44989869	G	A
Scaffold-42	45020389	TCC	TC
Scaffold-42	45020402	C	A
Scaffold-42	45020445	A	G
Scaffold-42	45020499	T	C
Scaffold-42	45020500	T	A
Scaffold-42	45204632	A	G
Scaffold-42	45204673	C	T
Scaffold-42	45204674	A	G
Scaffold-42	45204685	T	C
Scaffold-42	45278742	T	C
Scaffold-42	45278754	A	G
Scaffold-42	45278818	C	A
Scaffold-42	45289933	C	T
Scaffold-42	45290002	TCG	TCA,ACA,TAG
Scaffold-42	45312802	G	A
Scaffold-42	45312832	G	A
Scaffold-42	45312891	G	A,T
Scaffold-42	45312950	C	T
Scaffold-42	45312964	C	T
Scaffold-42	45313018	G	T
Scaffold-42	45414380	G	T
Scaffold-42	45414405	C	G
Scaffold-42	45453353	A	C
Scaffold-42	45453374	G	C
Scaffold-42	45453375	A	C
Scaffold-42	45748891	G	T
Scaffold-42	45748971	A	G
Scaffold-42	45748982	A	G
Scaffold-42	45748987	TCT	ACT,ACG
Scaffold-42	45796514	C	G
Scaffold-42	45796515	C	G
Scaffold-42	45796526	C	T
Scaffold-42	45796556	A	G
Scaffold-42	45796560	CATTT	CATTC,TATTG,CATTG
Scaffold-42	45796569	T	G
Scaffold-42	45797910	A	T
Scaffold-42	45797925	A	G
Scaffold-42	45797964	T	C
Scaffold-42	45797985	G	T
Scaffold-42	45841593	G	C
Scaffold-42	45841594	T	A
Scaffold-42	45841617	C	G
Scaffold-42	45841632	C	T

Scaffold-42	45841652	C	T	
Scaffold-42	45841662	T	C	
Scaffold-42	45841671	T	G	
Scaffold-42	45841730	G	A	
Scaffold-42	45870590	C	T	
Scaffold-42	45870607	C	A	
Scaffold-42	45870618	A	G	
Scaffold-42	45870638	T	C	
Scaffold-42	45870653	T	C	
Scaffold-42	45870671	C	T	
Scaffold-42	45904847	T	C	
Scaffold-42	45904861	G	A	
Scaffold-42	45904880	G	A	
Scaffold-42	45923566	T	C	
Scaffold-42	46017190	GCTTCAAACA		ACTTCAAACA,GCTTCAGACA
Scaffold-42	46017206	T	A	
Scaffold-42	46028869	C	T,A	
Scaffold-42	46028875	C	T	
Scaffold-42	46028876	GAAT	AAAC,AAAT	
Scaffold-42	46028884	G	A	
Scaffold-42	46028910	G	A	
Scaffold-42	46028916	G	A	
Scaffold-42	46028935	T	C	
Scaffold-42	46028941	G	A	
Scaffold-42	46028961	TTA	ATC,TTC	
Scaffold-42	46028972	G	T	
Scaffold-42	46028973	T	C,G	
Scaffold-42	46118305	G	C	
Scaffold-42	46118311	T	C	
Scaffold-42	46204921	C	T	
Scaffold-42	46204977	C	T	
Scaffold-42	46207289	T	G	
Scaffold-42	46207296	T	G	
Scaffold-42	46207309	A	T	
Scaffold-42	46207321	G	T	
Scaffold-42	46207392	C	G	
Scaffold-42	46207394	A	G	
Scaffold-42	46343374	G	A	
Scaffold-42	46343465	A	G	
Scaffold-42	46400405	C	T	
Scaffold-42	46400417	C	T	
Scaffold-42	46400493	A	T	
Scaffold-42	46400511	A	G	
Scaffold-42	46400544	C	T	

Scaffold-42	46507433	C	T
Scaffold-42	46507515	C	A
Scaffold-42	46507559	A	G
Scaffold-42	46507562	G	A
Scaffold-42	46507569	A	T
Scaffold-42	46579291	ACA	ACC
Scaffold-42	46608305	A	C
Scaffold-42	46608315	C	T
Scaffold-42	46608413	G	T
Scaffold-42	46608438	G	A
Scaffold-42	46608440	C	A,T
Scaffold-42	46628562	C	T
Scaffold-42	46628609	G	A
Scaffold-42	46628655	A	T
Scaffold-42	46628663	C	T
Scaffold-42	46628676	T	C
Scaffold-42	46628685	A	G
Scaffold-42	46632022	G	A
Scaffold-42	46632024	T	C
Scaffold-42	46632041	T	C
Scaffold-42	46632067	C	G
Scaffold-42	46632074	C	T
Scaffold-42	46632141	A	T
Scaffold-42	46651475	C	A
Scaffold-42	46651479	AA	AAGAATACTA
Scaffold-42	46651490	TCAA	TA
Scaffold-42	46651511	T	C
Scaffold-42	46651588	G	C
Scaffold-42	46651605	C	T
Scaffold-42	46659328	G	T
Scaffold-42	46659345	C	T
Scaffold-42	46659362	G	A
Scaffold-42	46665404	G	A
Scaffold-42	46665422	GA	TC
Scaffold-42	46665471	T	C
Scaffold-42	46800902	A	T
Scaffold-42	46800905	G	A
Scaffold-42	46800914	A	T
Scaffold-42	46800979	C	T
Scaffold-42	46800980	C	G
Scaffold-42	46800999	G	C
Scaffold-42	46955181	C	T
Scaffold-42	46955206	C	G
Scaffold-42	46955257	C	G

Scaffold-42	46978486	T	A	
Scaffold-42	47000987	C	A	
Scaffold-42	47000992	C	G	
Scaffold-42	47001001	G	T	
Scaffold-42	47001006	C	T	
Scaffold-42	47001016	G	A	
Scaffold-42	47001019	G	T	
Scaffold-42	47001020	TA	GA,AT	
Scaffold-42	47001026	C	A	
Scaffold-42	47001042	A	G	
Scaffold-42	47001057	G	A	
Scaffold-42	47001059	G	A	
Scaffold-42	47137656	G	A	
Scaffold-42	47137698	A	G	
Scaffold-42	47137743	A	G	
Scaffold-42	47137764	T	C	
Scaffold-42	47160523	TTCT	TTCC	
Scaffold-42	47225326	C	T	
Scaffold-42	47225351	C	T	
Scaffold-42	47225400	A	G	
Scaffold-42	47227386	T	C	
Scaffold-42	47304297	G	T	
Scaffold-42	47304372	A	G	
Scaffold-42	47359151	ATTA	TTTA,TTTT	
Scaffold-42	47359186	CCAA	CCGA,TCAG	
Scaffold-42	47359236	A	G	
Scaffold-42	47359243	T	G	
Scaffold-42	47491394	C	T	
Scaffold-42	47491447	T	C	
Scaffold-42	47491481	A	C	
Scaffold-42	47491517	T	A	
Scaffold-42	47491662	GATAA		AATAA
Scaffold-42	47491672	A	T	
Scaffold-42	47492450	A	G	
Scaffold-42	47492460	T	C	
Scaffold-42	47541608	C	A	
Scaffold-42	47541622	C	T	
Scaffold-42	47541632	C	T	
Scaffold-42	47541641	A	G	
Scaffold-42	47633804	T	G	
Scaffold-42	47633853	A	T	
Scaffold-42	47660133	T	G	
Scaffold-42	47660170	TAA	TAAA	
Scaffold-42	47660182	G	A,T	

Scaffold-42	47660196	A	G
Scaffold-42	47660231	C	A
Scaffold-42	47660244	A	T
Scaffold-42	47759331	G	A
Scaffold-42	47759415	C	T
Scaffold-42	47759436	A	G
Scaffold-42	47759450	C	T
Scaffold-42	47763126	G	T
Scaffold-42	47763226	A	G
Scaffold-42	47770808	G	A
Scaffold-42	47770810	T	C
Scaffold-42	47770836	AAACAACA	AAACACCA,AAACA
Scaffold-42	47770869	G	T
Scaffold-42	47846060	A	G
Scaffold-42	47984220	C	T
Scaffold-42	47984228	A	G
Scaffold-42	47984238	G	A
Scaffold-42	47984273	C	T
Scaffold-42	47984294	A	G,T
Scaffold-42	47984328	T	C
Scaffold-42	47984339	T	C
Scaffold-42	47984342	G	A
Scaffold-42	48026062	G	A
Scaffold-42	48026083	T	C
Scaffold-42	48026088	T	G
Scaffold-42	48026104	TGCGG	TG
Scaffold-42	48053184	T	G
Scaffold-42	48053201	T	C
Scaffold-42	48053236	C	A
Scaffold-42	48053259	G	A
Scaffold-42	48411340	T	C
Scaffold-42	48411389	A	G
Scaffold-42	48485852	T	C
Scaffold-42	48485933	T	C
Scaffold-42	48485934	G	A
Scaffold-42	48507351	TGTG	TGTA,CGTA
Scaffold-42	48507384	C	T
Scaffold-42	48507423	C	T
Scaffold-42	48507459	G	A
Scaffold-42	48507467	G	C
Scaffold-42	48539307	G	A
Scaffold-42	48563244	AAAC	GAAT
Scaffold-42	48563252	G	A
Scaffold-42	48563274	T	C

Scaffold-42	48563289	G	T
Scaffold-42	48563295	A	G
Scaffold-42	48617988	T	C
Scaffold-42	48618010	T	C
Scaffold-42	48618017	AT	TG
Scaffold-42	48618020	TAACCAACCAACCACCCAAGCTAATGAAATTAA	TA,TAACCAAAAAACCACCCAAGCTAATGAAATTAA
Scaffold-42	48618063	GC	GG
Scaffold-42	48618114	G	T
Scaffold-42	48618118	CGTCA	AGTCA
Scaffold-42	48642668	C	T
Scaffold-42	48902747	A	G
Scaffold-42	48968273	T	C
Scaffold-42	48968318	T	A
Scaffold-42	49058489	A	T
Scaffold-42	49058535	C	A
Scaffold-42	49058550	C	G
Scaffold-42	49058564	T	G
Scaffold-42	49058565	C	T
Scaffold-42	49058567	G	A
Scaffold-42	49058573	C	T
Scaffold-42	49058576	C	A
Scaffold-42	49058578	G	T
Scaffold-42	49058584	A	G
Scaffold-42	49058588	G	C
Scaffold-42	49078714	C	T
Scaffold-42	49078735	A	G
Scaffold-42	49078776	C	T
Scaffold-42	49291177	C	G
Scaffold-42	49291186	C	T
Scaffold-42	49291189	A	T
Scaffold-42	49291210	C	A
Scaffold-42	49291255	C	T
Scaffold-42	49291265	C	T
Scaffold-42	49291284	GTAACC	GC
Scaffold-42	49335074	TG	AA
Scaffold-42	49335084	T	C
Scaffold-42	49335100	GGCCGTTAATTTGGCACA	GGTCGTTAATTTGGCACA,GA
Scaffold-42	49335122	G	T
Scaffold-42	49335147	G	C
Scaffold-42	49336101	G	C
Scaffold-42	49336123	G	A
Scaffold-42	49336126	A	G

Scaffold-42	49336137	T	C
Scaffold-42	49336192	T	A
Scaffold-42	49336215	CTTTTAGATATTAT	CTATAGATATTAT,CT
Scaffold-42	49336232	A	G
Scaffold-42	49427190	G	A
Scaffold-42	49427234	G	A
Scaffold-42	49427291	G	C
Scaffold-42	49525777	T	C
Scaffold-42	49525789	C	T
Scaffold-42	49525806	T	C
Scaffold-42	49525822	T	A
Scaffold-42	49525828	G	A
Scaffold-42	49525849	G	A
Scaffold-42	49525861	A	G
Scaffold-42	49659339	A	G
Scaffold-42	49659412	C	A
Scaffold-42	49659421	C	T
Scaffold-42	49659476	C	T
Scaffold-42	49703395	A	G
Scaffold-42	49703403	A	G
Scaffold-42	49718848	G	A
Scaffold-42	49718890	G	C
Scaffold-42	49718917	G	T
Scaffold-42	49718921	T	A
Scaffold-42	49718926	GATAATTTTTTA	AACAATTTTTA
Scaffold-42	49738594	TACAA	TA
Scaffold-42	49738604	C	T
Scaffold-42	49738641	C	A
Scaffold-42	49738644	C	A
Scaffold-42	49738647	G	A
Scaffold-42	49738671	G	A
Scaffold-42	49739132	G	A
Scaffold-42	49739555	T	G
Scaffold-42	49750161	C	T
Scaffold-42	49750202	G	A
Scaffold-42	49750217	G	A
Scaffold-42	49829839	G	A
Scaffold-42	49829852	A	G
Scaffold-42	49829854	A	C
Scaffold-42	49829872	C	T
Scaffold-42	49829905	ACC	ACCATGC
Scaffold-42	49829943	TCTTGC	TTTTGC,TC
Scaffold-5	780229	C	T
Scaffold-5	780240	T	C

Scaffold-5	780242	G	A
Scaffold-5	780286	G	A
Scaffold-5	780314	G	T
Scaffold-5	1291092	T	A
Scaffold-5	1291159	A	C
Scaffold-5	1291163	T	C
Scaffold-5	1291196	T	C
Scaffold-5	1291219	C	A
Scaffold-5	1291223	A	G
Scaffold-5	1327929	ATGCT	ATGCTGCT
Scaffold-5	1327983	GTGG	CTGG,CTGC
Scaffold-5	3507976	G	A
Scaffold-5	3508012	G	A
Scaffold-5	3508071	T	C
Scaffold-5	4107077	GTAAT	TTAAT,TTAAA,TTGAT
Scaffold-5	4107130	C	T
Scaffold-5	4107158	C	A
Scaffold-5	4107164	C	T
Scaffold-5	4107179	C	A
Scaffold-5	6294112	C	T
Scaffold-5	6294143	AG	AT
Scaffold-5	6294166	GTCTAAAT	GTCTAAAA
Scaffold-5	6294178	GGT	GGC,GAC
Scaffold-5	6294190	G	T,A
Scaffold-5	6294216	C	A
Scaffold-5	6294224	C	A
Scaffold-5	7592708	C	G
Scaffold-5	7592713	A	T
Scaffold-5	7592714	C	A
Scaffold-5	7592740	G	A
Scaffold-5	7592749	G	A
Scaffold-5	7592823	C	A
Scaffold-5	9718200	C	T
Scaffold-5	9718209	G	T
Scaffold-5	9718261	A	G
Scaffold-5	11531493	G	A
Scaffold-5	11531506	TCCAG	TG
Scaffold-5	11531516	T	C
Scaffold-5	11531563	G	T
Scaffold-5	11531593	G	C,A
Scaffold-5	11531615	C	T
Scaffold-5	12161547	G	A
Scaffold-5	12161579	G	A
Scaffold-5	12619874	TG	TA,GA

Scaffold-5	12619922	T	A
Scaffold-5	14684737	A	T
Scaffold-5	14684739	G	C
Scaffold-5	14684752	A	T
Scaffold-5	14684765	T	G
Scaffold-5	14684776	T	C
Scaffold-5	14684784	TC	TAC
Scaffold-5	14684818	G	A
Scaffold-5	16348115	A	G
Scaffold-5	16348123	C	A
Scaffold-5	16348136	A	G
Scaffold-5	16348151	A	G
Scaffold-5	16348157	C	G
Scaffold-5	16348169	C	T
Scaffold-5	16348179	C	G
Scaffold-5	20740607	T	A
Scaffold-5	20740620	CGTTGTTT	CGTTT,CGTCGTTT
Scaffold-5	20867992	C	T
Scaffold-5	20868019	TTA	GCT,GAT,CGT,GTA,TAT
Scaffold-5	20868027	G	A
Scaffold-5	20868046	G	A
Scaffold-5	20868069	G	T
Scaffold-5	20868080	T	C
Scaffold-5	21561956	G	T
Scaffold-5	21562000	TTTTC	ATTTA
Scaffold-5	21562030	G	C
Scaffold-5	21562044	T	A
Scaffold-5	21565936	T	A
Scaffold-5	21565937	C	A
Scaffold-5	21565939	G	T
Scaffold-5	21565940	GCC	ACC
Scaffold-5	21565946	T	A,C
Scaffold-5	21565959	C	T
Scaffold-5	21565962	G	C
Scaffold-5	21565968	T	G
Scaffold-5	21565970	C	T
Scaffold-5	21565971	G	T
Scaffold-5	21565972	C	T
Scaffold-5	21565976	C	A
Scaffold-5	21565989	C	A
Scaffold-5	21565999	A	T
Scaffold-5	22224999	T	A
Scaffold-5	22225007	C	T
Scaffold-5	22225008	G	A

Scaffold-5	22225028	C	T
Scaffold-5	22225047	G	A
Scaffold-5	22225053	A	G
Scaffold-5	22225054	C	T
Scaffold-5	22225060	GGATGGT	GGACGGT
Scaffold-5	22225067	C	T
Scaffold-5	22225084	C	T
Scaffold-5	22225088	G	A
Scaffold-5	22225089	G	A
Scaffold-5	22225091	GGTT	GGTC
Scaffold-5	22225095	G	T
Scaffold-5	22225103	G	A
Scaffold-5	22225106	G	T
Scaffold-5	23151263	CCACG	TCACG,GCACG,GCACA
Scaffold-5	23151270	G	A
Scaffold-5	23151288	TCCCTCA	TCCCTCT,TCTCTCT
Scaffold-5	26498353	T	C
Scaffold-5	26498412	C	A
Scaffold-5	26498418	G	C
Scaffold-5	33775919	A	T
Scaffold-5	33775921	A	C
Scaffold-5	33775945	ATTTTTG	AGTTTTG,ATTTTTTG,ATTTG,ATTTTTA
Scaffold-5	33775990	G	C
Scaffold-5	33776012	A	T
Scaffold-5	33776026	C	T
Scaffold-5	33776046	T	A
Scaffold-5	34940081	GGTTTG	GG
Scaffold-5	34940096	C	T
Scaffold-5	34940108	TGGCG	CGGCG,CGGCC
Scaffold-5	34940113	AC	GG
Scaffold-5	34940139	GCAAC	ACAAA
Scaffold-5	34940174	A	C
Scaffold-5	34940178	G	A
Scaffold-5	34940180	GG	GC,AC
Scaffold-5	34940188	G	A
Scaffold-5	36636493	ATCTTCTG	ATCTTCTTCTG
Scaffold-5	36636504	C	A
Scaffold-5	36636523	AAAT	AAAG
Scaffold-5	36636527	TTGTTA	TTGCTA,TTGATA
Scaffold-5	36636558	G	A
Scaffold-5	36636569	A	G
Scaffold-5	39455064	A	C
Scaffold-5	42882718	G	T
Scaffold-5	42882758	C	A

Scaffold-5	44877243	C	T
Scaffold-5	44877261	G	T,A
Scaffold-5	44877275	C	T
Scaffold-5	44877284	A	G
Scaffold-5	44877288	TTTA	TTTG
Scaffold-5	44877299	G	A
Scaffold-5	44877300	AAATC	TAATC
Scaffold-5	44877316	C	A
Scaffold-5	44877320	G	A
Scaffold-5	44877331	T	C
Scaffold-5	44877336	C	A
Scaffold-5	44967233	T	C
Scaffold-5	44967248	C	G
Scaffold-5	44967251	T	G
Scaffold-5	44967260	A	T
Scaffold-5	48891006	TT	TG
Scaffold-5	48891008	GTTC	GTTA
Scaffold-5	48891015	G	A
Scaffold-5	48891020	T	G
Scaffold-5	48891056	G	A
Scaffold-5	48891102	A	T
Scaffold-5	49417469	C	T
Scaffold-5	49417502	C	T
Scaffold-5	49417616	A	G
Scaffold-5	49417673	T	C
Scaffold-6	1455857	T	C
Scaffold-6	4006239	T	A
Scaffold-6	4006259	T	C
Scaffold-6	4006265	G	A
Scaffold-6	4883225	T	C
Scaffold-6	4883233	GCTGCC	GCTGCCC
Scaffold-6	4883239	CTT	CTA,CTTT
Scaffold-6	4883286	G	A
Scaffold-6	4883292	CA	GA
Scaffold-6	4883296	C	T
Scaffold-6	4883299	C	T
Scaffold-6	4883310	C	T
Scaffold-6	4883312	A	G
Scaffold-6	4883325	ACGAC	ACGAT,AGGAC
Scaffold-6	4883330	TC	GA
Scaffold-6	4883360	G	T
Scaffold-6	4883363	A	C
Scaffold-6	5493974	C	A
Scaffold-6	5494028	C	T

Scaffold-6	5494047	T	C
Scaffold-6	5494095	T	A
Scaffold-6	5494097	G	A
Scaffold-6	10540907	A	T
Scaffold-6	11557720	T	G
Scaffold-6	11557751	G	T
Scaffold-6	11557762	CC	CT,AT
Scaffold-6	11557777	C	T
Scaffold-6	11557801	A	T
Scaffold-6	11557804	C	G
Scaffold-6	11557826	A	T
Scaffold-6	11557828	C	T
Scaffold-6	11557838	T	A
Scaffold-6	11557842	G	A
Scaffold-6	14128734	G	A
Scaffold-6	14128737	A	C
Scaffold-6	16045040	A	C
Scaffold-6	16045049	A	G
Scaffold-6	16045085	TCT	TTT,TT
Scaffold-6	16903793	A	G
Scaffold-6	17354487	AACACGA	AACACAA
Scaffold-6	17354499	C	T
Scaffold-6	17354512	TATATATCG	TATATATAG
Scaffold-6	17354576	T	C
Scaffold-6	18966321	A	G
Scaffold-6	18966328	A	T
Scaffold-6	18966335	CATC	TATA
Scaffold-6	18966351	A	C
Scaffold-6	18966369	T	A
Scaffold-6	18966379	G	A
Scaffold-6	18966389	C	A
Scaffold-6	18966396	C	T
Scaffold-6	20114305	T	A
Scaffold-6	20114323	T	A
Scaffold-6	20947883	G	A
Scaffold-6	21905888	CCTAA	TCTAA
Scaffold-6	22002427	G	A
Scaffold-6	22003078	T	C
Scaffold-6	22003150	A	G
Scaffold-6	22510080	C	T
Scaffold-6	22510092	C	T
Scaffold-6	22510093	G	A
Scaffold-6	22510173	A	G
Scaffold-6	25363234	A	T

Scaffold-6	25363248	T	G
Scaffold-6	25363264	C	T
Scaffold-6	25363274	C	T
Scaffold-6	25363298	C	T
Scaffold-6	25492857	GGTTGTTA	GGTGGTTA
Scaffold-6	27892579	A	T
Scaffold-6	27892597	AATGCA	ACTGCG
Scaffold-6	27892671	ACCCCCA	ACCCCA
Scaffold-6	30030941	AGGGCT	AT
Scaffold-6	32366548	G	C
Scaffold-6	32366593	G	T
Scaffold-6	32366604	T	C
Scaffold-6	32366626	C	G
Scaffold-6	32366628	C	T
Scaffold-6	36143467	A	T
Scaffold-6	36143488	G	A
Scaffold-6	36143522	C	T
Scaffold-6	39138120	C	G
Scaffold-6	39138121	T	G
Scaffold-6	39138126	A	G
Scaffold-6	39138132	T	G
Scaffold-6	39138173	T	C
Scaffold-6	39138179	G	C
Scaffold-6	39138183	C	T
Scaffold-6	39138209	C	T
Scaffold-6	39138212	C	T
Scaffold-6	39138218	G	C
Scaffold-6	39138232	C	T
Scaffold-6	39138234	ATG	GTG
Scaffold-6	40498530	G	T
Scaffold-6	40498574	A	G
Scaffold-6	40498586	C	T
Scaffold-6	40498589	CTT	CT
Scaffold-6	40498617	GTGC	ATGC
Scaffold-6	40498650	A	T
Scaffold-6	45751500	GAA	GGA,AAA
Scaffold-6	45751504	G	A
Scaffold-6	45751522	T	C
Scaffold-6	45751527	A	T
Scaffold-6	45751532	TTT	TTG,GTC
Scaffold-6	45751535	A	G
Scaffold-6	45751537	T	C
Scaffold-6	45751545	ATG	TTG,TTC
Scaffold-6	45751551	T	A

Scaffold-6	45751562	T	G
Scaffold-6	45751589	A	T
Scaffold-6	45751596	A	G
Scaffold-7	1166119	A	G
Scaffold-7	1166141	C	T
Scaffold-7	1227137	C	T
Scaffold-7	1227153	T	G
Scaffold-7	1245872	TTTT	CTTT,CTTA
Scaffold-7	1245896	A	T
Scaffold-7	1245917	A	T
Scaffold-7	1245919	T	A
Scaffold-7	5950672	C	T
Scaffold-7	13130855	G	A
Scaffold-7	13130867	ACT	AT
Scaffold-7	20762379	C	T
Scaffold-7	20762385	T	G
Scaffold-7	20762416	TAAGAAGAAGAAGG	TAAGAAGAAGG,TAAGAAGAAGAAAG
Scaffold-7	20762470	GGGTT	GGCTT,AGGTT
Scaffold-7	23407111	C	G
Scaffold-7	23407134	T	G
Scaffold-7	23407168	A	G
Scaffold-7	23407177	G	A
Scaffold-7	23407179	TTG	TTA,GTA,TTT
Scaffold-7	23407182	A	T
Scaffold-7	23407189	C	G
Scaffold-7	23407196	C	A
Scaffold-7	23505202	G	T
Scaffold-7	23505260	G	A
Scaffold-7	23866608	A	G
Scaffold-7	23866632	T	C
Scaffold-7	23866672	A	G
Scaffold-7	23867060	A	G
Scaffold-7	23867061	A	G
Scaffold-7	26695100	G	T
Scaffold-7	26695125	G	A
Scaffold-7	26695138	C	T
Scaffold-7	29009989	C	T
Scaffold-7	29010004	G	T
Scaffold-7	29010076	G	A
Scaffold-7	30455561	A	T
Scaffold-7	30455574	C	G
Scaffold-7	30455580	C	A
Scaffold-7	30455588	TC	CC,CT

Scaffold-7	30455591	T	A	
Scaffold-7	30455603	T	A	
Scaffold-7	30455625	TT	CA	
Scaffold-7	30455628	C	T	
Scaffold-7	30455632	T	C	
Scaffold-7	30455654	C	T	
Scaffold-7	30455671	C	T	
Scaffold-7	30455672	G	T	
Scaffold-7	33465259	CTA	CTTA	
Scaffold-7	33465270	ATT	ATTT	
Scaffold-7	33465279	TTACT	TTACTACT	
Scaffold-7	33465287	C	G	
Scaffold-7	33465294	A	C	
Scaffold-7	33465335	GAGCATACGTA		GA
Scaffold-7	33465371	C	T	
Scaffold-7	33683421	T	A,C	
Scaffold-7	33683473	C	A	
Scaffold-7	33843671	G	T	
Scaffold-7	33843674	C	T	
Scaffold-7	33843732	G	T	
Scaffold-7	33843759	C	G	
Scaffold-7	34726634	T	A	
Scaffold-7	34726668	A	G	
Scaffold-7	38032864	C	T	
Scaffold-7	38032871	C	G	
Scaffold-7	38032874	G	A	
Scaffold-7	38032885	C	T	
Scaffold-7	38032900	C	T	
Scaffold-7	39207368	A	G	
Scaffold-7	39207396	A	G	
Scaffold-7	40499221	T	C	
Scaffold-7	40499224	T	C	
Scaffold-7	40499237	C	G	
Scaffold-7	40499238	A	C	
Scaffold-7	40499246	T	C	
Scaffold-7	40499258	T	A	
Scaffold-7	40499259	A	T	
Scaffold-7	40499272	T	C	
Scaffold-7	40499290	C	G	
Scaffold-7	40499313	T	C	
Scaffold-7	40499318	C	A	
Scaffold-7	40499322	T	C	
Scaffold-7	40499325	CTA	CGA,TTA	
Scaffold-7	40499334	A	C	

Scaffold-7	42244614	GGTGA	GGTGG
Scaffold-7	42244656	CATG	CATC
Scaffold-7	42244664	T	A
Scaffold-7	42244713	CTCA	CTCT,ATCA
Scaffold-7	42945325	C	T
Scaffold-7	42945347	C	T
Scaffold-7	42949444	T	C
Scaffold-7	42949452	A	G
Scaffold-7	42949497	T	C
Scaffold-7	42949522	G	A
Scaffold-7	42949536	A	T
Scaffold-8	679592	T	C
Scaffold-8	679623	C	A
Scaffold-8	679624	G	A
Scaffold-8	679626	G	T
Scaffold-8	679628	ATCC	GTCC
Scaffold-8	679633	A	T
Scaffold-8	679637	A	C
Scaffold-8	679642	C	T
Scaffold-8	679704	G	A
Scaffold-8	3628299	G	C
Scaffold-8	3628305	G	C
Scaffold-8	3628316	G	A
Scaffold-8	3628351	GA	TC
Scaffold-8	3628377	A	C
Scaffold-8	3804267	A	G
Scaffold-8	3804344	A	G
Scaffold-8	5603203	T	G
Scaffold-8	5603252	T	C
Scaffold-8	9664062	T	C
Scaffold-8	9664064	G	T
Scaffold-8	9664071	A	G
Scaffold-8	15061334	G	T
Scaffold-8	15061342	G	A
Scaffold-8	15061347	C	T
Scaffold-8	15061362	G	A
Scaffold-8	15061367	ACT	ACG,ACA,ATG,ATT
Scaffold-8	15061378	G	A
Scaffold-8	15061390	G	C
Scaffold-8	15061397	A	G
Scaffold-8	15061421	G	T
Scaffold-8	16246269	T	C
Scaffold-8	16246376	C	T
Scaffold-8	16246388	A	G

Scaffold-8	18927700	G	A
Scaffold-8	18927764	T	C
Scaffold-8	24289612	G	A
Scaffold-8	24290137	G	A
Scaffold-8	24290218	C	T
Scaffold-8	24290242	T	C
Scaffold-8	25114450	A	G
Scaffold-8	25114467	TCCCCCCA	TGCCCCCCA
Scaffold-8	25114546	G	C
Scaffold-8	29947831	A	C
Scaffold-8	29947843	CCCACCACCACCACCGTAGTTTCCTC	CC
Scaffold-8	29947906	C	A
Scaffold-8	29947919	C	A
Scaffold-8	31757798	A	T
Scaffold-8	31757831	C	G
Scaffold-8	34784151	A	C
Scaffold-8	34784214	T	A
Scaffold-8	40809125	T	C
Scaffold-8	40809153	ACC	AC,ATC
Scaffold-8	40809159	G	A
Scaffold-8	40809196	T	C
Scaffold-8	43272809	TTGGT	TTGCT
Scaffold-8	43272822	CCTA	CCTG,TCTG
Scaffold-8	43272841	T	C
Scaffold-8	43272896	C	A
Scaffold-8	44515880	T	C
Scaffold-8	45311037	T	C
Scaffold-8	45311098	T	A
Scaffold-8	46209538	C	G
Scaffold-8	46209587	C	T
Scaffold-8	46209598	A	T
Scaffold-8	46209636	C	A
Scaffold-8	48358310	C	T
Scaffold-9	233896	T	A
Scaffold-9	233942	C	A
Scaffold-9	233949	G	A
Scaffold-9	325313	T	G
Scaffold-9	325320	C	A
Scaffold-9	325329	T	C
Scaffold-9	325334	A	C
Scaffold-9	325342	G	T
Scaffold-9	325355	C	G
Scaffold-9	325357	C	T
Scaffold-9	325381	G	T

Scaffold-9	325404	G	T	
Scaffold-9	325721	T	G	
Scaffold-9	325727	G	A	
Scaffold-9	325764	T	A	
Scaffold-9	325781	GA	AT	
Scaffold-9	325794	G	A	
Scaffold-9	325834	T	C	
Scaffold-9	325837	A	T	
Scaffold-9	2161237	A	G	
Scaffold-9	2161275	CGG	AGG,CGA	
Scaffold-9	2161282	A	G	
Scaffold-9	2161293	C	T	
Scaffold-9	2161298	G	A	
Scaffold-9	2161306	C	T	
Scaffold-9	2161311	A	T	
Scaffold-9	2161322	AT	CA	
Scaffold-9	2161333	C	T	
Scaffold-9	6078675	G	A	
Scaffold-9	6078725	GAA	GAAA	
Scaffold-9	6666730	T	A	
Scaffold-9	6666774	G	A	
Scaffold-9	7473281	T	C	
Scaffold-9	7473323	G	C	
Scaffold-9	7473342	C	A	
Scaffold-9	7473383	A	C	
Scaffold-9	7473386	C	T,A	
Scaffold-9	8615953	G	A	
Scaffold-9	8615959	G	T	
Scaffold-9	8615996	C	T	
Scaffold-9	8616035	A	C	
Scaffold-9	8616046	T	A	
Scaffold-9	8616047	GG	AA	
Scaffold-9	8616051	G	A	
Scaffold-9	8616052	T	C	
Scaffold-9	8616065	C	T	
Scaffold-9	8616067	G	T	
Scaffold-9	12437784	A	G	
Scaffold-9	12437806	G	A	
Scaffold-9	12437819	CA	GG	
Scaffold-9	12437848	TCTTCCA		ACTTTCC
Scaffold-9	14286483	A	G	
Scaffold-9	14286546	G	A	
Scaffold-9	14286548	T	C	
Scaffold-9	15042075	G	A	

Scaffold-9	15042113	GCA	GCCA
Scaffold-9	15042149	T	G
Scaffold-9	15042151	ACTC	ACTCTC,AC,ACTG
Scaffold-9	15042166	GT	TG
Scaffold-9	15042172	G	C
Scaffold-9	15471004	A	T
Scaffold-9	15471022	TAAAAAAG	TAAAAAAC
Scaffold-9	15471040	G	A
Scaffold-9	15471053	G	T
Scaffold-9	15956187	T	A
Scaffold-9	15956293	T	A
Scaffold-9	20800701	C	T
Scaffold-9	20800721	A	C
Scaffold-9	20800734	C	G,A
Scaffold-9	23779911	G	A
Scaffold-9	23779920	T	C
Scaffold-9	23779925	G	C
Scaffold-9	23779990	C	A
Scaffold-9	23779991	A	G
Scaffold-9	26689744	G	T
Scaffold-9	26689747	GGAA	GGAG
Scaffold-9	26689767	G	A
Scaffold-9	26689771	C	G
Scaffold-9	26689776	T	G
Scaffold-9	26689781	GT	GC
Scaffold-9	26689793	TGAC	TGAA
Scaffold-9	26689798	C	A
Scaffold-9	26689804	TAAAGG	GGAAGG,GAAAGG
Scaffold-9	26689823	G	A
Scaffold-9	26689842	GGAAA	GGAAG,GAAAA
Scaffold-9	26689857	G	A
Scaffold-9	29380429	A	C
Scaffold-9	29380441	C	T
Scaffold-9	32031465	C	A
Scaffold-9	32031470	G	A
Scaffold-9	32031471	C	T
Scaffold-9	32031488	CACCA	CACCG
Scaffold-9	32031511	TTGAC	TTGAT,CTGAT
Scaffold-9	32031516	A	T
Scaffold-9	32031524	CCTA	CCTG,CCTC
Scaffold-9	32031533	T	G
Scaffold-9	32031538	G	A
Scaffold-9	32031541	G	T
Scaffold-9	32031558	A	G

Scaffold-9	32031576	G	A
Scaffold-9	32031582	T	C
Scaffold-9	32031593	C	T
Scaffold-9	32031596	G	A
Scaffold-9	32031597	G	A
Scaffold-9	32031598	C	A
Scaffold-9	37137692	G	T
Scaffold-9	37137723	C	T
Scaffold-9	37137734	C	T
Scaffold-9	37137736	C	T
Scaffold-9	37137761	C	T
Scaffold-9	37137764	C	G
Scaffold-9	37137800	G	A
Scaffold-9	37137810	G	A
Scaffold-9	37137811	T	G
Scaffold-9	37137822	G	A,C
Scaffold-9	37481707	CCT	CCG
Scaffold-9	39293726	A	G
Scaffold-9	39293747	G	A
Scaffold-9	39293752	TTGGGCAAG	TCGGGCAAG,TA
Scaffold-9	39293785	C	G
Scaffold-9	39293787	G	A
Scaffold-9	39293795	T	G
Scaffold-9	39293798	CAG	CAAG
Scaffold-9	40652906	T	A
Scaffold-9	40652958	C	T
Scaffold-9	40854425	C	T
Scaffold-9	40854443	A	G
Scaffold-9	40854523	ACCC	ACCT
Scaffold-9	40971973	T	C
Scaffold-9	40971974	C	T
Scaffold-9	40972013	T	C
Scaffold-9	40972045	C	T
Scaffold-9	41442599	T	G
Scaffold-9	41442622	T	A
Scaffold-9	41442626	C	G
Scaffold-9	41442627	A	G
Scaffold-9	41442637	TTGGAT	TT
Scaffold-9	41442658	A	T
Scaffold-9	41442667	T	A
Scaffold-9	41442676	G	T
Scaffold-9	42967956	G	A
Scaffold-9	42968000	G	T
Scaffold-9	46478578	G	A

Scaffold-9	46478605	G	A	
Scaffold-9	46478607	A	C	
Scaffold-9	46478631	GTTCTTCT		GTTCTTTT,GTTCT,GGTCTTCT
Scaffold-9	46478650	A	T	
Scaffold-9	46478656	T	A	
Scaffold-9	46478659	A	G	
Scaffold-9	46478670	C	T	
Scaffold-9	48074905	C	T	
Scaffold-9	48074931	CG	TA	
Scaffold-9	48074960	C	T	
Scaffold-9	48074967	T	C	
Scaffold-9	48074997	C	T	
Scaffold-9	48075019	A	C	
Scaffold-9	48238086	C	G	
Scaffold-9	48238124	G	C	
Scaffold-9	48238139	G	A	
Scaffold-9	48238141	C	A	
Scaffold-9	48238144	G	C	
Scaffold-9	48238145	G	A	
Scaffold-9	48238168	G	A	
Scaffold-9	48238171	G	A	
Scaffold-9	48238172	G	A	
Scaffold-9	48238193	C	G	
